# A New Assessment of Thioester-Containing Proteins Diversity of the Freshwater Snail *Biomphalaria glabrata*

**DOI:** 10.3390/genes11010069

**Published:** 2020-01-07

**Authors:** David Duval, Remi Pichon, Damien Lassalle, Maud Laffitte, Benjamin Gourbal, Richard Galinier

**Affiliations:** IHPE, Université de. Montpellier, CNRS, Ifremer, Université de Perpignan Via Domitia, 66860 Perpignan, France; david.duval@univ-perp.fr (D.D.); remi.pichon@univ-perp.fr (R.P.); damien.lassalle@univ-perp.fr (D.L.); maud.laffitte01@etu.umontpellier.fr (M.L.); benjamin.gourbal@univ-perp.fr (B.G.)

**Keywords:** *Biomphalaria glabrata*, Thioester-containing protein, alternative splicing, immunity, Schistosome

## Abstract

Thioester-containing proteins (TEPs) superfamily is known to play important innate immune functions in a wide range of animal phyla. TEPs are involved in recognition, and in the direct or mediated killing of several invading organisms or pathogens. While several TEPs have been identified in many invertebrates, only one TEP (named BgTEP) has been previously characterized in the freshwater snail, *Biomphalaria glabrata*. As the presence of a single member *of that* family is particularly intriguing, transcriptomic data and the recently published genome were used to explore the presence of other BgTEP related genes in *B. glabrata*. Ten other TEP members have been reported and classified into different subfamilies: Three complement-like factors (BgC3-1 to BgC3-3), one α-2-macroblobulin (BgA2M), two macroglobulin complement-related proteins (BgMCR1, BgMCR2), one CD109 (BgCD109), and three insect TEP (BgTEP2 to BgTEP4) in addition to the previously characterized BgTEP that we renamed BgTEP1. This is the first report on such a level of TEP diversity and of the presence of macroglobulin complement-related proteins (MCR) in mollusks. Gene structure analysis revealed alternative splicing in the highly variable region of three members (BgA2M, BgCD109, and BgTEP2) with a particularly unexpected diversity for BgTEP2. Finally, different gene expression profiles tend to indicate specific functions for such novel family members.

## 1. Introduction

Thioester-containing proteins (TEP) are large secreted proteins playing central roles in the innate immune response [1,2]. TEP are characterized by the presence of a unique intrachain β-cysteinyl-γ-glutamyl thioester bond (CGEQ) originally discovered in the human protease inhibitor, α-2-macroglobulin (A2M), and complement C3 and C4 [3]. A2M are pan-protease suicide inhibitors that encapsulate targets after protease cleavage activation, leading to neutralization of targeted protease activity [4,5,6,7,8]; whereas complement factors deposit on target surfaces following activation, enabling the subsequent elimination or killing of the pathogens [9,10,11].

Phylogenetic analyses classified TEP proteins in two distinct groups, the complement factor and A2M depending on the presence of two domains, anaphylotoxin and C345C. The complement factor group is composed of vertebrate C3, C4, and C5 components and their orthologues (complement-like factors) in invertebrates. The A2M group comprises several closely related molecules: α-2-macroglobulin (A2M), macroglobulin complement-related protein (MCR), pregnancy zone protein (PZP), C3/PZP-like/A2M domain containing 8 (CPAMD8), antigen CD109 (CD109), and insect TEP (iTEP) [12].

Since the increasing availability of molecular databases for Metazoa (from Cnidarians to Mammalians), the thioester-encoding regions were found in many genes, that help in a better definition of the thioester-containing protein family. This ancestral family of molecules is present in all animal phyla except Porifera (sponges) [12]. However, each TEP type is not always recovered in all phyla. For example, seven members of this family are encoded in the human genome: Three complement factors (C3, C4, and C5), A2M, PZP, CD109, and CPAMD8. At the opposite, *Drosophila melanogaster* encodes solely six members, five iTEP, and one MCR [13,14,15]. The question of the origin and evolution of TEP was raised by several studies [1,12]. Indeed, iTEP was only found for Protostomia, MCR was, until now, only reported for arthropods, whereas complement factor TEP was never found in available insect genomes while present in all other arthropod groups. If vertebrate genomes are relatively well documented, data from invertebrates are too fragmentary to have a clear view of TEP gene distribution among them. Thus, additional data of TEP family member characterizations from organisms belonging to seldom documented phyla would be mandatory for a better understanding of TEP origin and evolution.

Among invertebrates, TEPs were most deeply studied in insect group, especially in flies and mosquitos, with *Anopheles gambiae* TEP1 (AgTEP1) being the only TEP for which a crystal structure was resolved [16]. Several TEPs were also characterized in other arthropod species, like in the Tick *Ixodes ricinus*, for which most of the TEP classes, including C3-like complement factors, A2M, MCR, and iTEP were described [17]. In *D. melanogaster,* iTEP and MCR are induced upon bacterial, fungal, and parasitic challenges [1,14,15,18,19,20]. In mosquito, AgTEP1 was shown to be able to directly bind malaria parasite and to promote their elimination [21,22], while *Aedes aegypti* iTEP have been shown to regulate *Flavivirus* infection [23,24]. Finally, the Mollusca phylum has been seldom explored, CD109 molecule was characterized in the cephalopod *Euprymna scolopes* [25], two bivalve species were reported to contain A2M, iTEP, and C3-like complement factor [26,27,28], and more recently five TEP members belonging to C3-like complement factors, A2M, and CD109 classes have been identified in the gastropod *Littorina littorea* [29]. However, only few data are available concerning the functional role of such TEP proteins in mollusc phylum.

The freshwater snail *Biomphalaria glabrata* (Bg) is the intermediate host of the trematode parasite *Schistosoma mansoni*, the agent of human intestinal schistosomiasis. As such, many studies have been conducted on the immunobiological interactions between these two protagonists [30,31,32]. In this host–parasite interaction was defined the compatible/incompatible status of the so-called compatibility polymorphism [33,34,35,36] that resulted from the co-evolutionary arm race between host and parasite [37]. Compatibility polymorphism can be defined as follow: A specific *B. glabrata* strain harbors variable degrees of susceptibility toward different parasite strains, whereas a defined *S. mansoni* strain presents different levels of virulence/infectivity toward different *B. glabrata* strains. Several studies have thus investigated key molecules of snail immune system and parasite virulence factors in order to decipher the molecular dialogue between *B. glabrata* and *S. mansoni*, and to explain this compatibility polymorphism phenotype [30,38,39,40,41,42,43]. Among the snail immune determinants playing a role in the host–parasite interplay, proteomic analyses have demonstrated the presence of a thioester-containing protein (BgTEP) within an immune complex formed by parasite antigen and snail immune recognition receptor [39,44]. A recent study characterized this BgTEP as being similar to insect TEP, secreted by hemocytes, expressed following immune challenges, and able to bind to different pathogen teguments including the parasite *S. mansoni* [45]. Binding of full-length and processed forms toward such pathogens, suggests important immune functions and potential opsonizing role [45]. In addition, a protease inhibitor activity of this BgTEP following proteolytic cleavage was previously provided by several biochemical studies, and more particularly against a cysteine proteinase secreted by *S. mansoni* [46,47,48]. Altogether, these results suggested a potential dual function of BgTEP as protease inhibitor and potential opsonin involved in snail immune response [45].

In the present study, we used transcriptomic data, as well as the *B. glabrata* recently published genome [49], to explore the diversity of TEP family members in the snail *B. glabrata*. We described 10 additional members of TEP family, based on the presence of thioester and A2M domains. We subsequently performed a phylogenetic analysis to classify these molecules and analyzed their exon-intron structures. We also determined the tissue expression pattern of BgTEP genes by quantitative PCR.

## 2. Materials and Methods

### 2.1. Ethical Statements

Our laboratory holds permit # A66040 for experiments on animals, which was obtained from the French Ministry of Agriculture and Fisheries and the French Ministry of National Education, Research, and Technology. The housing, breeding, and care of the utilized animals followed the ethical requirements of our country. The experimenter possesses an official certificate for animal experimentation from both of the above-listed French ministries (Decree # 87–848, 19 October 1987). The various protocols used in this study have been approved by the French veterinary agency of the DRAAF Languedoc-Roussillon (Direction Régionale de l’Alimentation, de l’Agriculture et de la Forêt), Montpellier, France (authorization # 007083).

### 2.2. Database Mining, Gene Identification, and Sequence Analysis

The transcriptomes corresponding either to naïve [50] (NCBI BioProject PRJNA213050) or to *S. mansoni* infected [51] snails from Brazil (BgBre1) (Recife, Brazil), and whole-genome sequence databases of *B. glabrata* [49] were screened to search for the presence of A2M (pfam00207) and/or thioester-containing (cd02897) domains to identify TEP related genes. When only partial transcripts were obtained from transcriptomic data for a given gene, the lacking portions of cDNA were confirmed by standard PCR amplification to complete cDNA sequences.

Then, genomic structures of the corresponding genes were determined using VectorBase database (https://www.vectorbase.org/organisms/biomphalaria-glabrata). 

Open reading frames for each gene and their encoded amino acid sequences were determined using Translate tool from the Expasy Bioinformatics Resource Portal of the Swiss Institute of Bioinformatics (https://web.expasy.org/translate/).

Conserved protein domains were searched using SMART tool [52,53] from the European Molecular Biology Laboratory (http://smart.embl-heidelberg.de/). Signal peptide sequences were predicted using both SignalP 4.0 Server (http://www.cbs.dtu.dk/services/SignalP-4.0/) and Interproscan. Transmembrane domains were predicted using the TMHMM Server version 2.0 (http://www.cbs.dtu.dk/services/TMHMM-2.0/), whereas glycosylphosphatidylinositol (GPI)-anchor sites were predicted using the GPI-SOM Server (http://gpi.unibe.ch/). Amino acid sequence alignments between all TEP proteins were generated by BioEdit software version 7.1.3.0 using ClustalW multiple alignment.

### 2.3. RNA Extraction and Quantitative RT-PCR Analysis 

Tissues were collected from 9 individual *B. glabrata* snails (Brazilian strain, Recife) under binocular microscope dissection. Albumen gland, head-foot, intestine, stomach, hepatopancreas, and ovotestis were recovered. Six hemocyte samples were each recovered by pooling the hemolymph of 20 snails followed by a 10 min centrifugation at 10,000× *g*. Total RNA was then extracted with TRIzol reagent (Sigma Life Science, Saint Louis, Missouri, USA) according to manufacturer’s instructions and subsequently reverse transcribed to first strand cDNA from oligo dT using Maxima H Minus First Strand cDNA Synthesis Kit with dsDNase (Thermo Scientific, Whaltham, Massachussetts, USA) according to manufacturer’s instructions. 

Quantitative PCR analyses were performed with the LightCycler 480 System (Roche, Basel, Switzerland) in a 10 µL volume comprising 2 µL of cDNA diluted to 1:30 with ultrapure-water, 5 µL of No Rox SYBR Master Mix blue dTTP (Takyon Eurogentec, Liege, Belgium), 1 µL of ultrapure-water, and 10 µM of each primer. Each couple of primers used in this study has been tested to determine the exponential and efficiency of PCR reaction. PCR amplification efficiencies were established for each target and house-keeping gene by calibration curves using two times serial dilutions of cDNA (from 1/20 to 1/640) in triplicates. Amplification efficiencies were calculated using slope values of the log-linear portion of the calibration curves by the LightCycler 480 Software release 1.5 (Roche). As a result, only the primer couples presenting a PCR amplification efficiency of 2 were retained for the study (Table S1). None of the primer used for quantification of gene expression was designed in region submitted to alternative splicing. The cycling program is as follows: Denaturation step at 95 °C for 2 min, 45 cycles of amplification (denaturation at 95 °C for 10 s, annealing and elongation together at 60 °C for 45 s) with single fluorescence acquisition at the end of each amplification cycle. Q-PCR was ended by a melting curve step from 65 °C to 97 °C with a heating rate of 0.11 °C/s and continuous fluorescence measurement. For each reaction, the cycle threshold (Ct) was determined using the 2nd derivative method of the LightCycler 480 Software release 1.5 (Roche). PCR experiments were performed in triplicate (technical replicates) from each biological replicate. The mean value of Ct was calculated. Corrected melting curves were checked using the Tm-calling method of the LightCycler 480 Software release 1.5. The relative expression of each TEP gene was calculated with the ΔΔCt method as the efficiency of both couple of primers (target and housekeeping genes) presented the same PCR amplification efficiency. Results were normalised with respect to a housekeeping gene: The S19 ribosomal protein gene, as previously described [45].

### 2.4. PCR Analysis of BgTEP2 Isoforms

According to BgTEP2 cDNA sequence, a couple of primer (F1: 5′-ACTACGGAGGCAGTGATGC-3′; R1: 5′-GATAGTATCTGGTACTGTTGC-3′) designed in non-variable parts of the sequence and framing the highly variable region was used to detect all the different alternatively spliced isoforms of BgTEP2 from snail tissues prepared as described above. Then, 1 µL of a 1:30 dilution of cDNA was used as template amplification with primers F1 and R1 using the GoTaq G2 HotStart enzyme (Promega, Madison, Wisconsin, USA) in a 50 µL final reaction, according to the manufacturer’s instructions. PCR reaction was performed in a Biometra TOne thermocycler (Analitik Jena AG, Jena, Thuringia, Germany), using the following cycling program: Initial denaturation 3 min at 95 °C, 45 cycles of amplification (denaturation at 95 °C for 30 s, annealing at 55 °C for 30 s, elongation at 72 °C for 20 s), final elongation of 2 min at 72 °C. Then, 20 µL of the resulting PCR were analyzed by electrophoresis on a 2% agarose gel. Another couple of primer targeting the cDNA coding for the ribosomal protein S19 was used as a positive control of PCR amplification (S19F: 5′-TTCTGTTGCTCGCCAC-3′; S19R: 5′-CCTGTATTTGCATCCTGTT-3′).

### 2.5. Phylogenetic Analyses

Homologous amino acid sequences were identified using BLASTp searches against the GenBank non redundant database (Bethesda, MD, USA). Full length protein sequences covering a wide range of animal phyla were used for multiple sequence alignment with the Guidance 2 server (http://guidance.tau.ac.il/ver2/) using the MAFFT algorithm [54] (Figure S1). Phylogenetic analyses of these aligned sequences was performed by using the maximum likelihood method and Whelan And Goldman + Freq. model [55], with pairwise deletion option as gaps/missing data treatment. A bootstrap analysis of 1000 replications was carried out to assess the robustness of the tree branches. The tree with the highest log likelihood (−345,206.19) is shown. Initial tree(s) for the heuristic search were obtained automatically by applying neighbor-join and BioNJ algorithms to a matrix of pairwise distances estimated using a JTT model, and then selecting the topology with superior log likelihood value. A discrete γ distribution was used to model evolutionary rate differences among sites (5 categories (+G, parameter = 1.5454)). The rate variation model allowed for some sites to be evolutionarily invariable ([+I], 0.06% sites). This analysis involved 134 amino acid sequences. There were a total of 5324 positions in the final dataset. Evolutionary analyses were conducted in MEGA X [56].

## 3. Results

### 3.1. Phylogenetic Analysis of *B. glabrata* Thioester-Containing Proteins

**Figure 1:**
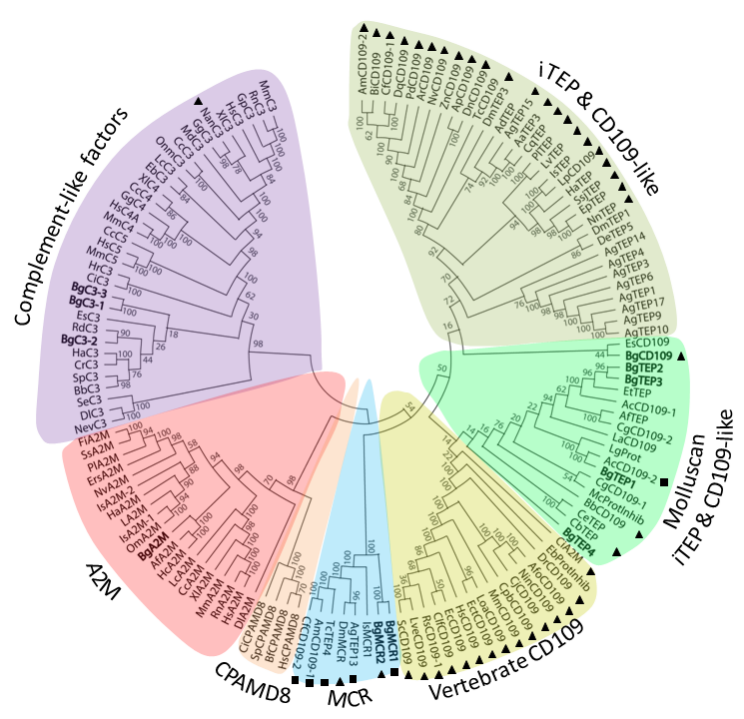
Phylogenetic tree of various thioester-containing protein (TEP) superfamily members. A total of 134 full-length protein sequences from diverse animal origin were used to construct the tree. A bootstrap analysis of 1000 replications was carried out on the tree inferred from the maximum likelihood method and the values are shown at each branch of the tree. BgTEPs are indicated in bold characters. Black triangles correspond to predicted glycosylphosphatidylinositol (GPI)-anchor signal, while black square corresponds to C-terminal transmembrane helix. TEP subgroups are highlighted by different colors: Purple for complement-like factors, pink for A2M, orange for CPAMD8, blue for MCR, and shades of green for iTEP and CD109-like.

Interproscan analysis of two snail transcriptomes was conducted to identify new TEP-related members and had revealed several positive transcripts that were assembled to generate a total of 11 TEP cDNA sequences (Figure S2). In order to investigate the relationship of the different snail TEP (BgTEPs) identified with various orthologous sequences recovered from other animal species, a phylogenetic analysis was performed using 123 other sequences (Table S2). Amino-acid sequences of full-length TEP superfamily proteins from both vertebrate and invertebrate phyla, including Complement-like factors, A2M, MCR, CPAMD8, iTEP, and CD109, and the 11 BgTEP sequences were used to construct a phylogenetic tree with the maximum likelihood method (Figure 1).

TEP molecules from *B. glabrata* belong to four clades of the TEP superfamily, and were named according to the actual nomenclature: (1) Complement-like factors (BgC3-1, BgC3-2, and BgC3-3), (2) Alpha2-macroglobulins (BgA2M), (3) Macroglobulin complement-related proteins (BgMCR1 and BgMCR2), and (4) insect TEP/CD109 molecules (BgTEP1, BgTEP2, BgTEP3, BgTEP4, and BgCD109). BgC3 molecules are close to the C3-like proteins from two other mollusk species, with BgC3-2 being more distant from BgC3-1 and BgC3-3 that clustered together. It is of note that a hypothetical transcripts corresponding to incomplete BgC3-2 cDNA was previously identified from the *B. glabrata* genome publication [49]. BgA2M also clustered with the A2M members from other mollusks. The two BgMCR clustered together and are the first macroglobulin complement-related proteins reported from the Mollusca phylum. Between the four iTEP, only BgTEP2 and BgTEP3 cluster together, while the two others are distributed among the cluster of molluscan iTEP and CD109-like. It is noteworthy that the name of BgTEP1 was assigned to the sole BgTEP previously identified from *B. glabrata* [39,45]. The taxonomic position of BgCD109 is less clear as it is more distant from BgTEPs and clusters with the CD109 from the bobtail squid *E. scolopes* (EsCD109).

As vertebrate CD109 are characterized by the presence of GPI-anchoring site, we looked for the presence of such GPI-anchor signal prediction as well as C-terminal transmembrane helix prediction in all the TEP members used in this analysis. This confirmed the presence of GPI-anchor site in vertebrate CD109, but also in numerous invertebrate molecules either named CD109-like or iTEP, including BgCD109. Most of the GPI-anchor site predicted molecules tend to form a specific cluster sustained by a bootstrap value of 92%. Another notable result is that iTEP and CD109-like molecules from mollusks tend to form a specific cluster separated from other invertebrate iTEP and CD109. Such classification has been previously highlighted [45] but is not well supported by bootstrap values, and need to be validated by an analysis including much more TEP members from Mollusca phylum.

### 3.2. BgTEP Protein Features Analysis

**Figure 2:**
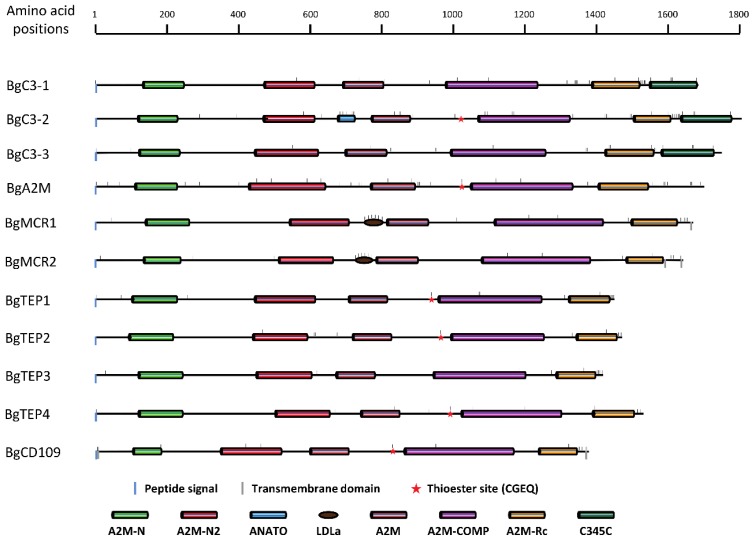
Schematic representation of BgTEP protein domains organization. Colored rectangles correspond to SMART conserved domains with following accession number: N-terminal alpha2-macroglobulin domain (A2M-N, PF01835), N-terminal α-2-macroglobulin domain2 (A2M-N2, SM001359), anaphylotoxin domain (ANATO, SM000104), low-density lipoprotein receptor domain class A (LDLa, SM000192), α-2-macroglobulin domain (A2M, SM001360), α-2-macroglobulin complement domain (A2M-COMP, PF07678), α-2-macroglobulin receptor-binding domain (A2M-Rc, SM001361), Netrin C-terminal domain (C345C, SM000643). Blue and grey vertical bar positioned under the main horizontal line respectively correspond to peptide signal and transmembrane helix predictions. Small horizontal bars positioned above the main lines correspond to cysteine positions in BgTEP protein sequences.

To confirm taxonomic positions revealed by the phylogenetic analysis, we investigated for the presence of evolutionary conserved domains in the BgTEP amino acid sequences using SMART tool. Figure 2 shows an overview of the protein domain organization for each TEP member. All BgTEP candidates possess a signal peptide meaning that all these molecules are secreted. Several conserved domains (A2M-N, A2M-N2, A2M, A2M-complement, and A2M-Receptor binding domain) defining the A2M proteins were also recovered in BgA2M with the similar organization. In addition, A2M proteins include the presence of a highly variable bait region, which determines the specificity of these protease inhibitors [57].

The other TEP superfamily members are defined by the presence of conserved domains similar to the A2M, but also possess additional features or domains. Indeed, TEP complement factors possess an extra C-terminal C345C domain characterized by four conserved cysteine residues that are likely to form internal disulphide bonds [58]; this domain was recovered in our three BgC3 (See Figure 2 and Figure S3). On the other hand, the anaphylotoxin domain and the arginine-rich region, two other specific features of C3-like molecules, were only recovered for BgC3-2 and not for BgC3-1 and BgC3-3 (Figure S3). The anaphylotoxin domain is characterized by a stretch of six cysteines, and the arginine-rich region is a classic posttranslational proteolytic cleavage site where C3 molecules could be cut into α- and β-chain by respective serine protease [59,60]. Furthermore, the canonical CGEQ thioester site is mutated in BgC3-1 and BgC3-3 protein sequences, which clearly indicates that these two members cannot bind target molecules via a functional thioester bond.

MCR molecules contain an additional low-density lipoprotein receptor domain class A (LDLa domain), in place of (i) the anaphylotoxin domain harbored by the complement factors or (ii) the A2M bait region [15]. This LDLa domain constituted by a cysteine-rich repeat typical from the N-terminal part of LDL receptor involved in lipoproteins binding is present in each BgMCR molecule. The presence of one or two C-terminal transmembrane helices were predicted respectively in BgMCR1 and BgMCR2, which is also consistent with MCR in other organisms [15]. Like all MCR orthologs, the canonical thioester site is mutated in BgMCR sequences. Finally, they also present a C-terminal stretch of cysteine residues, four of them being extremely conserved between all known MCR (See Figure 2 and Figure S3).

The four BgTEPs and the BgCD109 do not contain additional domain when compared with BgA2M. Nevertheless, they are shorter in length by 200 to 350 amino acids and present a C-terminal stretch of cysteines, a common signature of iTEP (Figure 2). Only BgTEP3 possesses a point mutation in the thioester site, changing the key cysteine into a serine residue (Figure S3). In addition, N- and C-terminal transmembrane domains were also predicted for BgCD109 molecule, which is consistent with the predicted GPI-anchor site, as CD109 are known to be cell surface-membrane bound molecules [25,61].

Another important feature is the presence of a catalytic residue located approximately 110 amino acids downstream of the thioester site in the primary sequence, but very close in the tridimensional structure of TEP molecules [3]. The nature of this catalytic residue partly determines binding specificity of the thioester site [62,63]. This catalytic amino acid was identified as a serine residue for BgA2M like in other mollusk A2M [26] and most other protostomia invertebrate A2M, while asparagine residue is the most commonly encountered in vertebrates [64]. Histidine, which is the typical catalytic residue of complement factors, was only found is BgC3-2 sequence, but also in BgTEP2, BgTEP4, and BgCD109, while BgTEP1 harbors an aspartic acid (Figure S3). It is noteworthy that aspartic acid is a well-known alternative catalytic residue also found in human C4a molecule [62].

### 3.3. Organization and Structure of BgTEP Genes

BgTEPs are large proteins ranging from 1389 to 1804 amino acid lengths (Figure 2). In order to determine their genomic organizations, BgTEP cDNA sequences obtained from two snail transcriptomes [50,51] were mapped to the genome BglaB1assembly [49] using the Blast tool of the VectorBase website. BgTEP genes are composed of various exon numbers ranging from 35 to 43 (Table S3). All reconstructed cDNA were retrieved in the recently published genome of *B. glabrata*. However, 9 out of 11 corresponding genes were distributed upon several different scaffolds in the BglaB1 genome assembly (from 2 to 11 different scaffolds). Only BgC3-3 and BgA2M genes were each assembled on a single scaffold. Moreover, several genes comprised missense or badly positioned exons, for example: BgC3-1 (exons 27-29), BgC3-3 (exon 33), BgA2M (exon 1), BgMCR1 (exons 23-26), BgMCR2 (exon 34), BgTEP1 (exon 31), BgTEP4 (exons 7, 16, 17), and BgCD109 (exons 10, 28, 29) (Table S3). BgA2M was the only gene not completely determined as the exon(s) coding for signal peptide and 5′UTR were not recovered in the snail genome assembly. Sequences of exon-intron boundaries were conserved along all sequences (Table S4) as already described for BgTEP1 [45]. Importantly, none of the BgTEP protein was correctly predicted from BglaB1 genome assembly (Figure S4).

**Figure 3:**
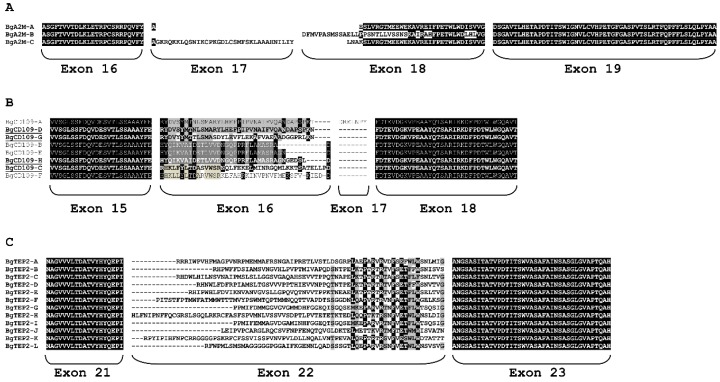
ClustalW alignment of alternative splice variants for three BgTEP proteins. Amino acids encoded by alternative exons corresponding to the highly variable region of some BgTEP were aligned, together with amino acids from border conserved exons, using clustalW. Identical amino acids are highlighted in black, similar ones in grey. (**A**) Alignment of BgA2M variants showing alternative splicing in exons 17 and 18. (**B**) Alignment of BgCD109 splice variants. Underlined variant names correspond to variants recovered from *Biomphalaria glabrata* transcriptomes. The other variants were only predicted from BglaB1 genome assembly. (**C**) Alignment of the 12 BgTEP2 splice variants.

Interestingly, alternative splicing was identified from transcriptomic data for the three genes encoding BgA2M, BgTEP2, and BgCD109 (Tables S3 and S4). The alternative exons encode the highly variable region corresponding to the bait region of BgA2M protein and to the proteolytic cleavage site for BgTEP2 and BgCD109 proteins. Thus, we identified three variants of BgA2M protein of which two were also predicted from BglaB1 assembly (Figure 3A). Regarding BgCD109, a total of eight protein variants were predicted from BglaB1 genome assembly, while transcripts corresponding to only four of these eight variants were recovered from Bg transcriptomes (Figure 3B). Particular attention was paid to BgTEP2 gene for which we identified 12 possible variants from *B. glabrata* transcriptomes (Figure 3C, Table S3), of which only one was predicted from BglaB1 assembly. The 12 possible alternative exons number 22 are positioned tandemly along 30kb length of the genome (Tables S3 and S4).

### 3.4. BgTEPs Genes Expression Pattern in Snail Tissues

**Figure 4:**
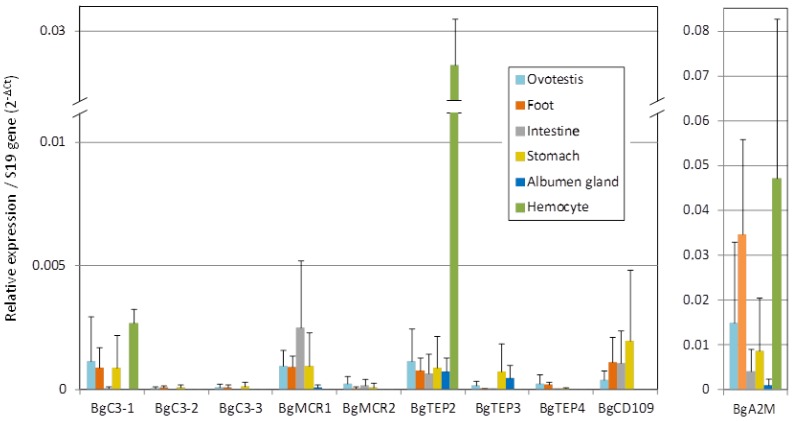
Relative expression of BgTEP genes in snail tissues. Transcription profile of BgTEPs in different tissues derived from nine individual snails. Six snail tissues were dissected: Ovotestis, head-foot, intestine, stomach, albumen gland. Six pools of hemocytes were each collected from 20 snails. Quantitative RT-PCR was performed on mRNA. C_t_ values of transcripts were normalized to the transcript level of the reference S19 ribosomal protein gene. Error bars represent the SD of the ΔC_t_ mean values obtained for each tissue. BgA2M is presented apart as transcripts level reaches a different scale.

Tissue specific expression of BgTEP by quantitative RT-PCR was conducted using head-foot, albumen gland, ovotestis, intestine, stomach, and hemocytes of *B. glabrata* (Figure 4). BgTEP1 was not included in the present analysis as already published [45]. We observed a large variability between the different *B. glabrata* TEP members in both tissue distribution and expression levels. At the tissue distribution level, the expression of all BgTEPs was detected in ovotestis, head-foot, and stomach, while it is more variable for other tissues. Six members belonging to the four TEP clades (BgC3-1, BgMCR1, BgMCR2, BgTEP2, BgCD109, and BgA2M) are also expressed in intestine, whereas the expression of only four members (BgMCR1, BgTEP2, BgTEP3, and BgA2M) was detected in albumen gland, and finally, three members (BgC3-1, BgTEP2, and BgA2M) were exclusively detected in hemocytes. At the expression level, BgA2M is 10 to 50 times more expressed than all other members, depending on the tissue considered, with the exception of BgTEP2 in hemocytes, which is expressed at the same level than BgA2M. The expression of BgC3-1, BgMCR1, BgTEP2, and BgCD109 was easily detected, while BgC3-2, BgC3-3, BgMCR2, BgTEP3, and BgTEP4 are more weakly expressed in most of the tissues tested.

We paid a particular attention to the splice variants of BgTEP2. We investigated the presence of putative spliced variants in different snail tissues by end point PCR amplification (Figure S5). In most tissues, several bands in the expected size range of 296-371 bp, were recovered, indicating that several isoforms of BgTEP2 are expressed in these tissues. In addition, we obtained variable band patterns between snail tissues suggesting that different BgTEP2 splice variants might be expressed depending of the tissue considered (Figure S5).

## 4. Discussion

In the present study, we identified 10 new TEP members containing A2M domain and/or thioester domain in addition to the previously identified BgTEP1 [45]. These new TEP members were named according to their classification provided by a phylogenetic analysis based on their full-length amino acid sequences. Three C3-like complement factors, one A2M, two MCR, three iTEP, and one CD109 were identified (Figure 1). Such diversity of *B. glabrata* TEP members is unexpected, as solely few members of the TEP family have been previously reported in Mollusca, and especially for Gastropoda. The class of iTEP/CD109 is the most represented in *B. glabrata* with a total of four iTEP (including the previously described BgTEP1) and one CD109.

All BgTEPs were further characterized by the presence of specific domains, like A2M domains for all members, C345C for C3-like complement factors, or LDLa for MCR. BgTEP1-4 present the C-terminal cysteine stretch that is a characteristic of iTEP [39], the MCR possess a C-terminal transmembrane domain and a mutated thioester site, and the CD109 show a GPI anchor-signal (Figure 2). The analysis of BgTEP gene structure revealed a number of exons from 35 to 43. Such structure is consistent with the TEP gene family yet described for bivalve [28] or for human [61]. However, these BgTEPs are rigorously different from *A. gambiae* or *D. melanogaster* TEPs, which are composed of 11 to 15 exons [65,66]. Such results suggest a different evolutionary history between mollusks and insects TEPs, as previously raised by other studies [13,67]. However, relationships between the two clusters formed by on the one hand vertebrate CD109 and iTEP/CD109 from mollusks and on the other hand iTEP/CD109 from other invertebrates are not clear as the clustering is not sufficiently sustained by bootstrap values (Figure 1). Additional iTEP/CD109 sequences from *Mollusca* phylum are needed to answer this question.

Herein, the presence of MCR molecules was reported for the first time from a mollusk species, whereas this subclass of TEP was previously demonstrated solely in few arthropod species. In addition, the presence of MCR genes in mollusk genomes may be much more common than previously thought. Indeed, in a recent study on the Yesso scallop *Patinopecten yessoensis,* five TEP members were identified from this bivalve species [68], among which a member was classified as a CD109 whereas it is more likely a MCR. Indeed, this molecule assigned as CD109 because of the presence of a C-terminal transmembrane domain, also possesses an LDLa domain and a mutated thioester site, which are typical features of MCR family members [15]. In the same way, two CD109-like molecules have been previously reported from the *Littorina littorea* transcriptome [29]. Nevertheless, the LlCD109-2 molecule from this gastropod also bears all the features of MCR, including LDLa domain, mutated thioester site, and C-terminal transmembrane domain.

The canonical CGEQ thioester site is mutated in 5 out of 11 BgTEP proteins: BgC3-1, BgC3-3, BgMCR1, BgMCR2, and BgTEP3. BgTEP3 harbors a single nucleotide mutation in this site leading to the replacement of the cysteine residue to a serine residue, but the Gln-moiety of the thioester is preserved. Such replacement of the cysteine residue by a serine was also reported for tsetse fly TEP2, an iTEP of which the expression is particularly up-regulated in the salivary gland following *Trypanosoma brucei* infection [69]. The replacement of cysteine residue in the thioester site was also evidenced for chicken ovostatin [70], without alteration of its proteinase-inhibitor activity [71]. The absence of thioester site in the BgMCRs and two of the three BgC3 suggests that these molecules cannot covalently bind to microbe surface, but that does not mean that they are not functional. Indeed, these molecules possess several other protein–protein interacting domains, and many other TEP proteins without thioester site were shown to exert important immunological functions. For example, MCR from *A. aegypti* was shown to be implicated in the regulation of Flavivirus infection, by binding to a scavenger receptor that also interacts with viral particles, leading to the regulation of antimicrobial peptide with anti-flavivirus activity [24]. MCR from *D. melanogaster* was reported to specifically bind the *Candida albicans* surface, and to subsequently promote its phagocytosis [15]. Otherwise, some TEP molecules with canonical thioester site were shown to be able to bind pathogens in a thioester-dependent or independent manner, like the mosquito AgTEP1 towards bacteria [72]. Moreover, it was recently evidenced that some invertebrate TEPs also play roles in other functions than immunity. Drosophila MCR was found to be implicated in development [73], nitric oxide regulation, and metabolic processes [74,75], while AgTEP1 was shown to increase male fertility [76].

The level of BgTEP genes expression from *B. glabrata* tissues was investigated by RT-qPCR in order to investigate a tissue-specific expression. BgA2M and BgTEP2 were recovered from all tested tissues, with BgA2M being the more expressed in all tissues (Figure 4). We found that BgMCR1, BgA2M, BgTEP2, and BgTEP3 were expressed in the albumen gland, an accessory gland of the genital tract also known to produce immune relevant molecules, like antimicrobial glycoprotein LBP/BPI, or C-type lectin [77]. Otherwise, expression of BgC3-1, BgA2M, and BgTEP2 in hemocytes suggests a potential role in immune functions in addition to BgTEP1 known to be expressed by a specific subtype of hemocytes [45]. The probable immune function of BgTEP2 is supported by its expression in hemocytes and by its identification in a proteomic screen searching for the *B. glabrata* plasma proteins able to bind surface of *S. mansoni* sporocyst [44]. Indeed, in this study, Wu and collaborators identified peptides corresponding to BgTEP1 but also to another candidate named CD109 antigen-like (see Table 2 from [44]). We confirmed that the peptide sequences of this “CD109 antigen-like” perfectly matched to BgTEP2 protein sequence described herein. This suggests that BgTEP2 is able to directly or indirectly bind *S. mansoni* tegument, as shown for BgTEP1 [45].

Finally, we paid particular attention to BgA2M, BgCD109, and BgTEP2 that present alternative splicing in their highly variable central region. We found from *B. glabrata* transcriptomes three possible forms of BgA2M (BgA2M-A to BgA2M-C) coded by either one or two exons (Figure 3A), while only two of these three variants were predicted from BglaB1 genome assembly (Figure S4). Such alternative splicing was also reported for the A2M of the shrimp *Fenneropenaeus chinesis* [64] or in insects, and may serve to extend the repertoire of inhibited proteases [21].

Concerning BgCD109, eight isoforms were predicted from BglaB1 genome assembly (BgCD109-A to BgCD109-H), while only four were recovered from *B. glabrata* transcriptomes (BgCD109-C, -D, -G, and -H) (Figure 3B). This might, of course, be due to the fact that the unrecovered transcript forms are not expressed in the available transcriptomes used herein. However, incorrect prediction of some variants from BglaB1 genome assembly has also to be considered as (i) BgCD109 isoforms A and D have the same exon 16 (same genome position), but differ by the presence of an additional exon 17 only predicted for isoform A that was never reported to be expressed and probably does not exist, and (ii) BgCD109 isoforms B, E, and H are predicted from the transcription of the same exon 16, which starts at the same position but ends at different positions for the three isoforms, that is highly improbable and explains why only one of these isoforms (BgCD109-H) was recovered from Bg transcriptomes. Unlike other TEP members, CD109 is a GPI-linked glycoprotein, which clearly indicates different function than other TEP. CD109 was originally found on endothelial cells, platelets, and activated T-cell of human [78], it was shown to interfere with TGF-β signaling, to be highly expressed in some cancer diseases, and to play role in bone metabolism [79,80,81,82]. The role of CD109 in invertebrates remains seldom investigated, nonetheless for juvenile Hawaiian bobtail squid *E. scolopes*, a down-regulation of CD109 (EsCD109) expression in the light organ of the squid after colonization by its bioluminescent symbiont *Vibrio fischeri* was shown. The authors suggested that the presence of the symbiont modulated the squid’s immune system, including EsCD109, to permit symbiont growth and colonization of the light organ [25]. In the present study, the only data available about BgCD109 is that it is not expressed by naïve snail hemocytes (Figure 4). However, it is to note that for bobtail squid too, EsCD109 transcript was not detected in hemocyte transcriptome [83], while the EsCD109 protein was detected by Western blot in the animal blood [25], suggesting that circulating CD109 might be produced by other immune tissues than hemocytes and secreted in the hemolymphe.

The more intriguing molecule is BgTEP2 for which 12 different variants were recovered from *B. glabrata* transcriptomes. After mapping on the genome, the variants were found to be the result of alternative splicing from 12 mutually exclusive exons tandemly positioned along 30 kb in the BglaB1genome (BgTEP2-A to BgTEP2-L) (Table S4). The amino acid sequences encoded by these 12 exons are variables in length and composition. However, some amino acids are extremely conserved in the C-terminal part of the sequences encoded by these exons (Figure 3C) suggesting a key role in the structure-function of the molecule. To our knowledge, such degree of diversity is not common. Moreover, BgTEP2, the most expressed member in hemocyte, might display a central role in the innate immune function of the snail as it was shown able to bind *S. mansoni* larva surface [44].

The highly variable region of BgTEP2 and BgCD109 corresponds to the bait region of A2M, which is known to be structurally exposed and sensitive to protease cleavage [57]. Such cleavages induce conformational changes of the protein and activation of the internal thioester, leading both to fine interactions with targeted proteins, and to the exposition of receptor binding site allowing fixation to receptor-bearing cells followed by clearance of the targeted protein [84,85,86]. Such diversity in the sequences of these highly variable regions might produce different avidities/affinities to various ligands. Alternative splicing in this bait-like region was previously highlighted in several invertebrate species. The A2M-2 of the shrimp *F. chinesis* harbors six variants in the same part of the sequence that are activated differently toward *Vibrio* challenges [64]. Moreover, five isoforms of *Drosophila* TEP2 were transcribed by alternative splicing of the bait region encoding exon 5 [14]. Finally, seven isoforms of *Chlamys farreri* TEP (CfTEP) were reported, being produced by alternative splicing of six mutually exclusive exons. Only one isoform is expressed in the gonads, which differs between male and female, and the other isoforms were induced following immune challenges, in a pathogen-dependent specific pattern [87]. In *B. glabrata,* several isoforms of BgTEP2 display a restricted expression pattern in naive snail tissues suggesting a broad repertoire of recognition molecules. Their patterns of expression following specific immune challenges will deserve further investigations.

*D. melanogaster* possesses six TEP differing in their preference between opsonizing different pathogens [15]. The discovery of 11 TEP proteins in the genome of *B. glabrata* and the multiple alternatives splicing for three of them will generate a high level of protein diversity in this family, increasing in the same time the affinity/avidity of such immune receptors towards potential pathogens. Moreover, biochemical study has shown that BgTEP1 is likely to form homo-tetramers [46], that might also contribute in increasing the binding capacities of these BgTEP molecules by heteromeric recognition complex formation. To conclude, this paper revealed a diversity of TEP family member higher than expected in a gastropod species and could potentially enhance the snail immune repertoire.

## Supplementary Materials

The following are available online at https://www.mdpi.com/2073-4425/11/1/69/s1, Figure S1: Alignment file of TEP protein sequences used for the phylogenetic analysis; Figure S2: cDNA sequences of the 11 TEP of Biomphalaria glabrata; Figure S3: ClustalW alignment of BgTEP protein sequences with related sequences from other organisms; Figure S4: ClustalW alignment of each BgTEP protein sequence with corresponding protein sequences predicted from BglaB1 genome assembly; Figure S5: BgTEP2 alternative splicing in snail tissues, Table S1: Table list of primers used to measure gene expression by quantitative PCR; Table S2: Table list of protein sequences used to construct phylogenetic tree; Table S3: Genome organization of BgTEP genes; Table S4: Genome organization of BgTEP2 splice variants; File S1: Supplementary materials caption.
