# Supplementary material for: A New Assessment of Thioester-Containing Proteins Diversity of the Freshwater Snail Biomphalaria glabrata
Source: Genes (Basel). 2020 Jan 7;11(1):69. doi: 10.3390/genes11010069 (PMC7016707; doi:10.3390/genes11010069)
Supplement: Supplementary file 1 [file genes-11-00069-s001.zip › Figure S2.docx]

**>BgC3-1**

TGACGTGTCGAGTACTAGTCTCACAGAAGTCTTATTACAACATGTCTAGACGTAGAAGTCACTGAGGCCTGAGTTCAAGGCTTGCTTATAAAACTCAGTTTTGTTTCTATTTGACTGAGACATCTTCTAAATAGCATTACCTCACTTTTGTTTTACGATTGTGTGATAACAAAGAAAGTCTCAGACTTCGTATTGTGCAAGTCAACCTTAAACTTGGTGGTTCTTCTCGTGGCTCTCAATAGATCAAGAGGCCATAACTCCAACAAAAAGTTTGACTCTTCTAATCAGAGATACTTAACGAGATACATTCCAAAGGATAAATTCTTATTTTTTAACGGATAACCAAGTGCTGATTGCATTAGTTCTATTTTAAATCACAAGAGAGCTGAGTATCAACTACACGTATTTTAAGTTATCTACACTAGACTAGATCAAGAACAAGATCATTTAATTAAGTTTACAAATTGAAGAAAA**ATGCCATCTATCAGATTTACGAGATCAACTTCAAGATTTATATTTTGTACCCATTTGTTGTTTTTACTTGTTTTTGTCAACATGATGAAAAACTGCTTCAGCACAAAATATCTGTTAACTGTTCCTAAAGAGCCAACTTATGACACAGACCTAACAGTTACTATAACAGCTTTAACTAAACCAGAAGTACCTGAAAATATTACTTTAGAGTTCAGAGGATTGAAAGATAAAAGCAAAGTTTTAAACAGCACACAGATAAATTTTAGCGAAGATGAAACCAAAAATTGGACAGTTGTTTTTCCTTGGGAACGTATGCTAGGGCTGGATGAGACTGGAGTCTTACTTAAAATGAGAACTACAAATGAGATGGAGAATGTAGTGGAAAAGGATTTATCTTTAAAGTTTCGTAATACATCAGGATATATTTTTATCCAGACAGATAAACCCATTTACACCCCAAGACAAACAGTGAAGTTCAGGATAATTGCTTTAGATGAGTACCAAAGGCTTACAAAGTATCCAATCAAAGTTGACATTAAAAACCCACAGGGTGTGATTCTGGAAAGGATGAGGTACTCTGCTGAAGATGCCTTTAAGAGCCAAGAGTTTGAGTTGCCCAAAGACACTCCACCAGGAATATGGACTATATCAGCAAACTTGGAAGGACTGGGTCAGTTATATAGTCTAGCTCATACTGTTGCCTTTGAGGTTAGAGAGTATGTACTGCCAAGGTTTTCAGCGGTGTTTAAGATTGATACAGATGTCATAACCATGGACACAACTTGGATAAGGATGAATGTTACTGCCAAATATGTCTATGGACAGCCTGTGGTGGGTAAAGTTGAAATGCGTCTGGGAACATGGGATGAAAATTCCTCTGTGACATTGATACCAAGTGCTTCTTATCGGGGTGAGCTGATCAATGGGGTGTTCAAACGTGATGTTAAAAGGTCATCATTGTTTCCGACCAATGAGTCATTTAATGGAGTTAAAAGACTCTATGTACAGGTCAATGTAACAGAAACTGCTACCCAGGAGACAATTACTATTGAGGACACTTCTACCTTCGTCTCTCACCCTTATTATGAAGTTGACTTCACACCTTCTAAAACTTACTTTAAACCAGGATTCCCCTACACTGTTCACGTTCAGGTCAAGGCCAGGAGTGGACGTCTTGCCTCCTGGGTGTTATTATATCTGCACCCTAAGTTCTATGACTCAGAAAAACACCTGTTGAGAGAATCAAACAACCATGTGATTGTCCAGCCTCTAGACATGTATGGGAAACTGAGTATGGAGGTACAAATACCATTGAACGCAGACAGAGTCATTTTTAGTGCTATAGTAATGGATTTTGCAAAAATCAAATTCAATGAATACATTCTCAATGTTTCAAAACTAGCTTCAGATATCAATGAATATATTGTAATATCTATGCCCACACCTATACAGAAAATCCAGAGGGGAGAAGTCATTTTAAATTATACTAAGCCCAGAAATTTACTGGACAAGATAACAGTGCTGGTTATAGCCAAAGGCTATGTTATCTACACTCTGAAAAATATTACTAAAAACAATAATGGAAGCAGCACAATATATCTGCCAACCAGTTTGCGTGGTGATGCATCTCCCAGCATGAGAATAGTTGCTTACTATTGGACTATGGGTGAAATCATCATGGATTCCTTGTTTATAGAGGCGCCAGTAAAATACTGTGTGGAAGAGCTGTATGTGAACAAGGGAGGTTTATTTAGCACAACTCCTCTGAAACCAAAAGATAAGCTTAATATTGATTTGAGAGGAGGCAGCAATATGAGAGTTGGTTTGGTGGCTGTGGACAAAGCAGTGTTGTTACTGAATGATAAGCAGACCCTGACCAGGAAGTTGCTTTTCAATGAACTGGAGAAACATGACCAGGGAACACATAAAAATAATGGAACCTTTGAGGAAATATTAAAAAGTAATGGTCTTCAGTACATATTTCTAGACACAGTTCAGGTAGAATCAGATGATCCACCACCTATAGGTACGGTTAAAGAAGATGGGTTTGATGAAACAGATAGCTTCATTGCAATGCAGCAAGCTCTACCACCACAAACAGTGCGGAGTTATTTCCCCGAGTCCTGGATGTTTGAAGAACATGTTTTGCCCAAAAGTGGATTCTTACGCTTAAGTTGGCCACTTCCTGATTCCATCACTACCTGGTCAGTGCTAGTTGTTGGAGTCTCTGCCAACCGAGGTGTCTGTGTATCAGAGCCTGTGGATCAAATTGCCATGAAGATGTTCTTTGCTGATGTTCATGTGCCTTACAAAGCTACCAGGTTGGAGGAGGTGAAGGTCAAGATAGCTATCTACAACTTTTATAACTACACGCTGGCTGTTCAGGGAACTGTCACCAGCGAACCTGGCCTCTGCATATCCAGTAACTCCAGCCAAACTTTCAATTCAGGAACATCATTGAAAACCTTGACATTTTCCATGAACATTGCTGCTTTTCAGACAGCCAGTGAGATTATCAAGGTTATTCCCCTTAAAGTTGGGGAGCTAGGTTTGTTAGTCCATGTTAAGTCTCAGAAGGATGAAGATATTGTTAAGAAGACCCTTCATGTTGTGTCTGAAGGACTCAGAGTATTTAAAACTATTACCTTTGTACTGGATCCTGAAGCCAAACATGCAACATTTCAAGGTCGCACTCATTTCTCAACTATCAGAAATCACATTGATAAAATCAAGAAGCAGCAGTTTACAACTATAGATTTGGCATTGCCTAAAGATGTCATCAAAGGAACAGAATTCTGTGGCATTTCAGCTTTTGGTGATTTAATGGGTGATATCATCACACATGGTATAGTCCGCTCCAAAAGTTTTGTTGACCAACCTTTAGTCAATGCAGAAGAAGTCATTGGTGATCTTGGGCCAGCTGTCTTTGCTCTGCAGTATGTCAATGACACTAAGTTGCTGACTGATGAGTTGAAAAACAAAGGGCAACGTTTCTTGTTACAGGGAATAACTCGTCTGTTGAACTACAGAAAGGAAAATGCTTTTAGTTTACATACAGACTCTAGTCCAGCTACCTGGCTGACTGCCTCTGTCGTGAAGATACTCTGCCATATTGAAAAAGCAAACCTGACCTTCATTGACAAGGAGAATCTGATAGACAATGCTATTAACTGGATCATGAGGCAGCGCAGGGAAGATGGCTCTTTGAAGGAAGCAGACCCAAGACTTAGCCAGGAATCACTGCAGTACAAAATCATGTTGGCAGCTGATGTGATGATCTCATTGCTAGAATGTAATAAGGATGAAGAGCAAGAGGCTGCTGAATTGATTATGGGTCTGGTAACATTTATTGAAAACAACATTGACAACATTAACAACTCCTTAGCACTTGCCAAGGCTGCCTATGCTATGAAACTTTTTGAAATAGACTCTGAGAGTACTGCACAAATCATCACCAAGTTAAAGGAGTTTATGAAGAGAGACAAACTCAGTCGTCTTTATTGGTCAGATTCTGTTATAGATAATCCACCTAGGCAACCTATATGGTACCATCAAGGGGCCACAGCTGCTGCAATTGAAGCTACATCTTATGCTCTCCTGGTGTTCCTGGACCATAGTATGATCAAAGAAGAAGCCATTGCAGACTGGCTGGTGGCACAGAGAAACCCAAGTGGAGCATTCATTGGTGCTATGGATAGCACTGTAGCCATTCAAGCTCTCACCAAGTATAGCCAGAAGAGATATGTCCTCTCTGGCAATGCTGTCTTTCTTCGTGGTAACATCACATCTGACATAACACGTAGCCGCAACCACCTGCATTCTTTCAAGTTCACTGAGGAAAATGCCACAAGCCCAGCATCTGTCAAAAATGTTCCAGTCGGACAAGTTCTAGAGGTATTTACTGAAGGTCAAGGTCTGGGTCAGATGCATGTGAATGTAGAATACAACATTCCCATTGAGAAGAACCTTCAGTGCCACTACAATGTGACTGTTGAGATCAAGCCAATACAATATATGCCTTCTTCTGTTAATACGAGTCCACTTTGCCAGTACTGTAATATTGGCTGCCCAGCGAACCTTAGATCTCGTAAAGAAATTAGTGACGTGTCTCCCATCATTAGGATTGGTCGTGCCAGAAGTAACCTAAGCAAGAGTAGAGCTGTCAGAAGTAGCAGTCACAGCAAAAAGGTCTACTGTTTCCACGTATGCTTGAGATTCATTCGTACTGAAGGCAATGTCCCTATCAATATCAAGATGGACATGTTGTCTGGATTTAAACCAGTGGCTTCAGACTTGGAGCTGATCAAGTCTGGACCCAATGTTCTACATGTTGAGTTTCAAGCAGGAACTGAAACACTGGTCATACAGTTAAGCAAGGTGGACACTGAGAGTCCTTCTTGTTTTGGCTTTAGAGTTGTAGATGATGAAGAGGTTGAGAGGAAAGTCCCAGCAGCTTTGATTATCCAGCAAGTTGGTCATCCAGTTCCATCCTGTACTTTGGAATATCATCTTCCTGACTCTCTAGAGAGTCTCAAAGTATTCTGTGCTGACTTCTCACACATCAACAGAGGAGAGTGCAGGTGTTACTCAGGTTTGTGCAGCAAATGTCGACCAACACACGCCAATGAACTTGATCTTGATAAAACTAAAAAACTTGTCTGCAAAAAAGAAATAGCCTACCAGCTGCGTCTTGGAGATGTACAAGACAAAATTCACTGGATGGAGATAGATGCTAGAGTTTTGTCTCTCAACAAGACTGGTAGCCACAAACTGGAAGCAGGGGACACAATCAAAATGATGTCTCCAAGCTCCTGTTCATGCTTGGTCAACAATTACAGAGATGAAGATTTCTACATGCTGTCTAAAGATGTGGAGAGATTGGTTGATAGAAGGGGAGAGACAGTCTATCGTTATTTGTTGGATGAAAATGCTGAATTCCTGCATGTGGAGAAGCCAGGTGTTCCATCAACAAGCAGTCTGGTCCCTCCTGTACCGTTTCCATACTTGCAACAAGCACTCAGCAAGGATAATGTTTGCAGAGCATAG**GTACTGAAAAGTATGTCGGCAGAGTTTTTTTTTTTTAAATTGGAAAAA**A**AGATAAAGATAAAATCAAAATATGTTATATCACAGAAGTCATAAAAAGCTTTTGTAT

**>BgC3-2**

AATTAAATATTTAAAATCTAGTGGTTAAGGAGCTATTAATGTGGTCTATAAAATAACATATTGTGATAAAGTAGATATATTGTGGTTCAGCAGATATTTTATTGTGGCCTAGCAGATATAATATTTTGGTCCAGCGGATATCTTATTAAGGTCCAGTGGATATCATAGTGTGGTCCAGCAGACATCATAGTGTGGCCAAGGAAATACATTTCTAGACAAGGATTTA**ATGAGAGACATCATGCAGACGTTTTGGATTCTACTGCTTCTTCTGGTTCCGGTATCCTGTAGTTCTCACCACTTTGTACTTCTGCCGAGCGTTCTGCGCCTGGAGACTGAGGAGGTCTTCAGTGTGACGTCGCTTGAAGCCGAGGGCGACGTTACATTCAAGATCTACCTGACGGATTACCCGGAGAGGAAGAGGAATTTCTCAGAAACAACGGTCACAGTACCCCAAGGAGAAAGTGTAATGGCCAGGGTGTTGATGACAGTGGGTGACCTTCCGCAGAACGCCCAGCCCCCACTTTTTGTCAATGTCATCGTCATGACAGTTGACCATGAGCCTCACTTTCAGAAGGAGGCTGTCATCTTGGTCAACGAGAGTCCCGGTTATATATTTATCCAGACTGACAAACCGGTTTATACACCGGACCAGTCGGTTTTTACCCGTGTGATGACTTTAAACGAACATTTCCGGCCAGCGAGCTGGCCACTACAAGTGGACATCCAGAACCCTGACGGAATGACCATAAGCCGAAAGGTCATCGACTCCAAAGATCTCATCCTAAAAGATGTGATGAAAATCCCAGAAAACCCTGTGTACGGCAACTGGACAGTGACGGCGAAATTCATCAACGGGCTCAGGACAACTAGCGCTGTCAGATTTGAGGTCAAGGAATACGTTCTCCCTACAATCTCTGTCAGTTTTCACATCCCAGACTCCAGGAAGGTCATCTTGCCCAACGAAACTCATTTCCATTTAGCTGTTGGAGCAAAATACATGTACGGAAAGCCTGTCAGAGGTCACGTGACGGTGACCTATGGACTCCTTTGGCATGGGCATGTCTTTACCGTTGGTAAACAGAGAAATCTACAGCTGAATGATACTGGCTTCGTGGAGTGTGGGATCACCGTCGACGACCTCAGGCTTCCGGTCCAGTCCGTCTGGTTCCCTAACGGCGGTAAGCTCCACGTGCAGGCGGCCGTGACGGAGACGGCTTCCGGTCACGTCGAAAAAGCAGACGACACCAGCGTGGTCTTTGCCGACCACTTGTACGTCATCAGGTTCACCCGATCGGACAGACATTTTAAACCAGGACTGCCTTATGTCTTAGAGATAGATGTCTTCAAAGCTAATGGTGAAACTGGCCCATACCTGGCACTGTCAGTGGAGTGTCAGATAGAGATGCAAGATGGCACCAAAGAAACTATTCCAGCTACTGGACAGTATGATGGACATTCAATGGTCACTGATGCTAGAGGAAAACTTAGCGTCCACTACAATATTCCGGCCAACGCAAAACAGCTTCATTTCAAGGTTTCTCCCAAAGAGAAGGCGACCACTGACCAGGCCAGTAAGTCCGACTACTTCTTCATCGCAAGCAGGTTCTACTCACCATCCTCTGTCTACATGCAGCTCCATGCCAGGCTGACTGATACGAACAGATTTGGCTACACTCCTGCAGTCGGAGATCACCTGACCGTATGGACCAGCTACACTTCGCCAGAGGAAATATCAACAGTTACGCTAGTGGTCATCTCAAGGGGCAGCATCGTTTGGCAGGTCAGCACCCGAAACATCCTTGGCAACTCCACCTACTTCCACTTCAAGATCACCCAGGATATGTCTCCAACGGCCTGGATCCTGGCCTTCGCCGTCAGGGGGAATGAGCCAGGTTCAGAGGTCATCTCCGACTCGGTCTGGCTGGAAATTGTTCCACAATGTGATGGCGAGCTTCACATTCAGCGCGAGAATGACGGGAAGAAGGTTCTCAAGCCTGGAGACATCGGCACAGTTACACTGACGGGTCAGCCCTACATGGTGGTTGGGGTCGTGGCCGTTGACAGTGCGGTCTACCGCTTGAAGAACAGCACTTTGACCAGACAATCTGTATTTCAACAAATCACTGCTCACGATCGAGGCTGTGGTTTTGGAGGAGGAAAGGACGCTGCCAAAGTATTTGAGAACTCTGGTCTGATGGCTCTGACCAATGCCGACCTGCCGATGACCCCTAAGACTGTGGATGGATGTGTGGACAAAGCCGTAAGGAAGAAGAGGTCTCCAGAAGCAAGGAAACGTGCCAGAGATATCTGCTGTGTGGAAGGAGCCCGGGTTCGCAACGCCACGTTGGCTCTCTGCTACTTCGCCACCCAAGAACTCAAGAAGACGATGAACTCCGAGTTCTGTGTCCGGGAGTTCTTCCAGTGCTGCAGGAGTTTCGTGAAAGGAACGCTATCCCTGGACGCTCTGGGCCGGCTGAGAACTTCCATTGAAAGACTCCCAGAGGACATCGAACTTAACTTTGATGAAGATGATCTGTCCAACATGAAGAACATTCCTGTAAGAACCAACTTCCCAGAGTCTTGGTGGTTTGAGGAATACAATCTAGGAGCCGAGGGTCGTGCTGACGTTGACTTTGTACTACCGGACTCCATAACCACTTGGTCAGTGGAGGCGCTGGGCATGTCAGTGGAAGCTGGGCTCTGTGTGGCGCCGCCGCTGGAACTGACCACCTTTACTTCTTGTTTCGTTCACCTAGACCTGCCGTACAGTGTGGTCAGGCTGGAGCAGGTGGAGGTCAGGGCCACCGTCTACAATTACATGACCAAGAAGATCAGGGTCAACCTGATCCTACAAAGTGTCGACGGCGTCTGCTACTCTGGGCAGCCAGGAGACGCCACGGATTACGTCAAGCTGGAGATCGACCCCAACGATGCTGCCTCTGCCTACTTCCCCATTGTCCCTCTGGAGATCGGCACTTTTCCCATCATTGTCAAGGCTTTCTCCACCTGGGGGAGGGACGCTGTGGAAAAGACGCTCAGGGTTGAGGGTGAAGGCCTGGAGAAGATCCACACTATCTCTGTCATGCTGGACCCAAGTGGTAAACGTTTTCTCAGAAGCAGGTCTTCAAATCATACTTTCAACATGAAAAATGAGGTCAGATCAGCAGAGAAGAAACAGAACGTGGAGCTTGACCTCGACCTTCCCCAGGAGGTCATACCAGACACAGAGAGCTGTTCCGTACATGCTATGGGTGACTTGCTTGGACCAACCCTTCAGGTTATGATAGAGGGCGTCACGGAACTACTACGTTTGCCCACTGGGTGTGGGGAGCAGAACCTCATCTACCTGGCTCCCAATGTGTTTGTTACTCGCTATCTCCGAGCTACCCGCAGACTGACAAGTTTCATAGAGAAGAAAGCCCTGGCCTTGATTCGACAAGGTGTTTCCAAGCAAATGTTTTTCCGAAAAGTCGACGGTTCCTTCGCAACCTGGCCTCATGCAGAATCCAGCACCTGGCTGACGGCGTTCGCCATGAAGACTCTGTGTCAGGCTGAGCACTACGTCACAGTAGACCACAACCAGACTTGCTCGTCTTTTCACTGGATCGCCAAGCAGCAGAAACCAGACGGCAGCTTTAGGGAAGAGGTCTGGGTGACCCATAGAGAGATGCTTGGTGGTGTGAATGGAGATGTCAGCCATGCGGCTTTCATTCTTATTGCATTGTTGGAATGTGATTGCCCTGGAAATGATCATAAGGACGTCACTGCCCAAGCGCTGCGATACATAGAAACAACAGTGGCCCAGACGGACCGCCCATTGGCATTGGCAATCAGCGCTTTCGCCTTGACATTAGCAGGCTCTCCATCCAGTGACGGTGTAGTCAAGAGACTGCAATCAATGGCTAAGTCATCTCCAGAAGGGTATACGTATTGGTCTCACGGCACAGAGGAGGATTACGAGGGGCATGAGAAGCCCTACTGGTACACCAAACAGCCCGGGGCCCTGGCCGTGGAGGTGACCTCCTACGCGCTTCTCACCAACCTGGCCCGGGGAGACATCTCGACCAGCACCGGCATCGTGGGCTGGCTTCTGTCACAGAGGAACTCACAGGGAGCATTTATTTCTACTCAGGACACTGTGGTTGGTCTACAAGCGCTGTCAGAGTACAGCATCAAGTCATACTCGGCCATTCTGGACATGACATGCCACATCAGGTCCGAGGTGGATGACCACTTCAGAAAGTCCATCTCGCTGACTCCGGAAGACGCTATGGTGGTCAAAACTGTCCCGAAGGTTCCAACTGGAGGCAAACTTCACTTCGAGGCTGAGGGTACAGGTGTCGGCATGATGCAGGTGGAAGTCAGGTTCAACGTCCCTGAGGACCGCAACAACTGTCACTTTGACGTCACAGTGGCAACACACCAGCATAACACCTTGCTGCAAAGTTTCTTCTGGGACAACAGAAAGTCCAAGTGTGAGCCTTGTTCAACGGACTGTGAAGAGCAGACGAGCGAGGAGGAGGAGGAGGAAGATTATGAAGACTTCACCTTCCCTCCAGTTCAACCCAGAATTCAAACCCTGTGGAAGAAGAAGGTCAAAGGTCTCAATTTGACAGTACATGAAGATGAGAACAGATCTGATTTTGAAGATCCTAACATGGTATCAAAGATTGGCCGCCCACGGCGAAAGCGACGATCTGTCAGACCGTACAGTGCCTCTGTCATTTGTGTTGAAGTCTGTGTCAGGTTTCTAGGTAACAAAACCACTGGCATGTCTGTAGTAGACGTGGGGCTATTTACTGGCTATCTCCCAGTGGACGAAGACTTGGAAAATTTGAAGTTGAAAGGTAAAATCGACCACTACGAGAAGTCGCAGAGATCTGTTGTCCTGTATGTTGATGAGTTCACAAACCGAGACAGAAAGTGTCTTAAACTGCGAGCGAGACAGGAACATGTTGCTGAGAATCTACAACCAGCTAAAGTTCAAGTCTTTGATTACTACAACCCAGATTCGAGGTGTACAGTCTTCTATAAGAACAACAACAATAGCGGACAGTTGGCGAACTTTTGTGATAACCAAAAACAGATCTGCCAGTGCTTAGAGAGTCGTTGTGCTGCGTGTGAGGAGAGCTGGTACGGTCTCGGTTGGATGGATATGATGAAGTTCGCCTGTAGCAACGCGTCTTATGTTCTTGAAATCAAGGCTCTCGACAGAGACTTGGAAAAAGCTGGTTTCGAGAGAATTCTTGGACAAATTCAAGCCGTTCATTCTCAGAGAGGCCGTCACGAGCTAAAGGTTGGTGACAAAGTCATTCTGCTGAAGAGAGCGTCTTGCTTCTGTCCAAGGGTCAGTCCAGACCAGACTTACTTCATGATGTTGTCCCAGCCAAAGAGATTCAAAGACTCGGACGGAAATCAAATATACGCCTTTCTGATGGACAAAAAAGTGCTGGTGATACAGAACTTCAAGCCAAGAGGTCTGAGCAGGGAACAGAAAGAGATCTCCAAGAACGTCAACAGGACTGTGAAGCGCCTCAAAAGACGAGGCTGCTCGGGAGGCGGGAAGACGCCCAAAGTGAACGGCAAGAATAGAAAGAGGAGAGCACGGCGCGGGAAAAACGGAAAAACCAAGAAAACGTAA**AAACGAGAATGTGACATTAATTTTTTTTGTTTCTCTTGACCGATTTGTTTTGTTTCCTTATGTGATTATGTTTTGACGTTACGTTATTTGTTTGTTTCATTCATTACAAATACAGTTCTATACAATGAAGAGATCAAGTGTTTATACAATGCATTATGCTTTCATGATTATGTTAATTCTTATTTTTCTGTATTTGTTGACTTTTTTTTAAACGTTTTTAGGAGAATAAATTCATTGTCAGTAAAACTAGAAGCTCGTGAATCAGTGATGCTCAAACTATGGCCCGCGGACCACATCCGACCCATGACTCGGTGATGTCAGACTCGTCGTGAGTTTTGTCAACAGAGCACAATCAAACCTCGATGGGTTCCACGATTTATATTTTTCAGGCCGTGTTGCGGTCGGCCTGTTCAATATATATATTTATGAGCCCATGATTATATATATAGTTAATCAAATATTACGTATTTCATATAATCCTACAATTTAAAACAAAGTATCGAAATGTTAAAATTGACCTTTTCATGGTTTTGTCTTCTGGCCTACATCCATGTTTTTGTGTTTGGAAATTCTTTCTTTTGTCACGTGGTAAGGTGATAATCCATATAGTTCTAATGGACATTAATGCCCTGTTCATTTAATGGTTTCGTCCAATAGAATGGAGCCTATTTCCTGTTCATTTGGTGACGTCGTACAATGAAACGAGCCAATGCAGCTTTATACTAAGCAATAAGTATTGACATTGGACTTAAGTGTCGAAGTGCCAGATATGGTCCACAGGCCGTTCCTTAGGCATCAGTTGCGTAGAATATATAGATCTATGTAAATAAAACCTTCTCCAGCATTTATTTTTCATAGTCCAATGAGTATATCGATGATGAACTCAAAATGCAAGTACCCTTTAAGTTTGCAATCATCGAGAACTGAACAATGAATAATCTTCAGAACTTGAATCTTTTTTTTCTTTTTTGAAGTAAATATAATGACAGGAAATATTCATAATGAAAATACAAGTGAGCAGATGCAGTGGCGTCAGTTAGAAGGGCGAAGGGTACGGATCGCACCTGATAACACCCATTATTTGGGGAGGGGGGGATTAGGTAACAATGTCAGAAACATGTCCACGCACATTTAGCAGTTGTGTAACAATTTATGAACTTTGGGAACTTGAGCTTTGTATTATGTTTCAAGATGGAGATTGAAAAAAAAGGATTTTATAAGAAAAATTTATTTTGGTAGGAGGAGATGGGGTGCCACCATTAACTAATCTCACAAGGTAACCCTAGCCTGACCTATGTATAGAATTAATCACTTTTTATTTTCGTGTCAGGGCCGGCCTTAGATAATTGAAGGCCCAATACCAAGTGAATTTGATGGCCTTAATGAATT

**>BgC3-3**

GCAGAAATAAGGTCAAGGAAGCTCGAACAGAGAATGAGAGGCAAGTGCGACGTATTGCCAGGATTTCCCACGTGTTTTTGGTGAAATAGTATTAACAGACCAGACAGTTTGGGGCGAAATTGAAAAGTGTTAACTTATAAACGTT

TTGGAATAACAAGGAAAGGAATGCCCGAAACAATCACGTGACTCTATTCTGTT**ATGTATGTGAAAAGAACAATGACAGTGGCTGATGGCCTAAGCCTAATCCTATCAGTTTGTCTAATTCAACAATCTGTGGGGACAAAATATTTTCTGACAGTGCCCCAAGTACCAAGCTATGATGCTAATGTGACTGCAGTGGTCACAGCTTTTCAACACACTTCTCAGTCTACTGAGAAAGTTCTTCTTCAATATATAGGAGGAGAGAACAGTAAAAACGTTTTGAACTCAACCCATCTTTCTTTTGATCAAGATGGTTCACAGCAATGGACTGTTACTTTTTCTTCTGAGTCGATGCAAGAATTGCGGGAGAGCAGTGTTGTTCTGCAGATGACATGCAATGGTCAAAAAAAAGAAATTTTACTAACATTCCGTCAGTCTTCTGGCTACATATTTATCCAGACAGACAAACCAATTTATACTCCAGGCCAAACAGTAAAGTTTAGAGTAATAGCAGTTGATGAGGATCAAAGGCTGTCCAAACATCACCTGAAAGTTGATATAATAAATGATCAGCAAGTTACAGTTGATAGAATGAGATACTCTGCTGAAGATGCATTCAAAGGTCAAAATTTTGAACTGCCTAAGGACATTGCACCTGGTCGATGGTATATTTCTGCCAATTTTGAAGGTTTGGACAGCAACTATAGATTGGCTCACAATGTGAGCATTGAAGTTAGAGAGTATGTACTTCCCAGATTCTCAGCCACACTCCATGCCAATACAAGTGTTATCACCAAAGATTCTAAAGCCTTAAAGCTCACTGTAACATCCAAATATGTGTTTGGTAGGCCTGTACATGGTAATGTTGAAATACATTTAGGGATATTGGATAATAACAAACTGCTTCCTCATGCTGTCTTAAGAGGTAAGCTTCAAAATGGACAGTTTTCACAAGATGTGGATGTCAACATCCTGACTCTGTCAAAGCTGATGTACACAAGTAACCAAAGGCTTCATGTGGGGGTCAATGTCATAGAGAAGGGCACTTTTGAAAACTACACACTAACAGACTCATCCATATTTATCAGTCATCCTTACTATATTGTTGATCTAAAGTCTTCAAAGGAGTTTTTCAAACCTGGATTTTCATACACACTAAAAGCAGTTATTAAAACTAAAGTCCCTCTGACAGTATCACATTTAGATCTTTACATTTTTGCTGAATTTTTAGATGCCAATGACAACATCATAAAAAGCGTCTCTGAAAATGTCCCTATAATGAAGAATACTATTGTGACTCAAGATTTCATTACACCAAAAACAGCTGAGAAAATCAACTTTAAAGTCCATGTTGTGGATGAGAATCATCCAAGCTTTGAACACTTCCATTTCACTGTAAAGAAATATATCTCAGCTAACCATGAGTATCTGCATATTAATATGTCCAAGTTTCAGCCAGTCATGAAATGGAGTGATGGAGTATTTTTCCTCGAATATACAAAGTCAGCCTATTTGAACAGCAGTAGTCTCATCACTGTTAATATATTGAGCAAAGGCCAAGTGATCTACTCAATAAATGTCAAGAAAAATATTCTGGGAGTCTCCCCTGTTAGTTTACCAAAGCAGCTTTTTGGAGAGCTCTCCCCAGCTTACAGGATTGTGGCCTACTACTACATAGCAGGGGCTGTGCCAGAGCTTGTGGCTGATTCTTTGTTAGTGGATACAGAATTGGATACTTGTGTGGATGAAGTTTATTTAATTCGTGACAAGTTCTCCCAATTTTCGCCTGTTCCTAAGAAGCCTAAGGATAAATTAGACTTGCTTATTATTGGTAGCCCTTTAATGAAGATTGGGCTACTGGCAGTGGACAAGGCAATATTTCTTCTAAATGATAAGCAAACACTGACAAGAGAACTGTTGTTTCATACATTAGGCAGCCATGATCCCAGTACCAGTGAAGGAGATGGTCTCAATGCAGAATTGATTCTAGAAAATTCTGGACTTTATCATATGATGGTAGATACAGATGCTTATTCAACTTCTGTCACTCCTCGTCGTGCCTTGAGTTCATTTGGCTCCTTCTATGATATTTCTTTTGACAGAATAAATATGCCAGAAGAAAATAGACATGGTAATCAAGTGTCAGAAAAGCAACCAGAGACTGAAGACTCACTTCCACAACGTTCTGATGTCAGATTTTATTTTCCTGAAACCTGGTTGTTTGAAGAAAAGATTATTCCAAGGGACAGGAAGTTACCTCTAGAGCTCTCTCTACCTGACTCCATCACAACATGGTCTTTTGTGGCTGTTGGGCTCTCAAACAATCGTGGCATTTGTGTATCCATCCCTCTGGAGCAAGTTGTGGAGAAACCAGTCTTCTTGGAGGTCAGAATGCCATTCAAAGCATCAAGGTTGGAGGAGCTCAACATCAATATCATCATACACAACTACCACACTAATGATGTCAGGCCTGAGGTGACTATTATTGGTGATTCAGGACTTTGCTTTGCTGAAAATGCTACAAGGGGAGGCAATCACAGTGATCATGGCTTTAACATGACAGTTACAGCAGGTGAAATGGCTGAAAGAACTGTTAGAATCATTCCACTAAAGATTGGAGAATTGACATTAAAAGTGTCCATGATAAGCCACTTAGGAAATGATACTGTGGAGAAAAAACTGCGAGTTATAGCTGAGGGACTTAGAGTGCGCAAAGCTATCACTTTCGTGTTGGATCCTGGAGCCAAACATACAACTTTTATGAATTATAGTGACAATAACATACAGCAGTCAAACACTGCCACTATTCAAAATAGATACATAGCATCCAGAAACATGCAACATACAACTATAGACCTGGCCTTGCCGCCAGAGGTGATCAAGGGAACTGAATCTTGTCAGATCTCTGCATTTGGTGACCTTATGGGAGACATCATTACCCATGCTGTTGTCCAGTCTAAGGGACTGATGGAGGAGCCAACACTCATTGCTCAGGAGGTGCTTAATGACCTTGGTCCAATAGTCCATGCCCTAAACTACATCAACGACTCAGGCTTGATGACTCATGACCTTAAGCTGAGAAGTCATCGCTTTATCAGGCATGGTGTGGTGAGATTATTGACCTACAAATCTGGTAAAGCTTTTAGTGTCAGACCAGGGATGAAGCCAGCTACATGGTTGTCAGCTCTCATACTAAAGTCACTGTGTCATGCCACATCCTTGGCATTCATTGACAAACATAACTTGATAGATACTGGCTTCAGCTGGCTTCAGGATCAGATTAAAAAAGATGGTTCTCTAAATGAGCTAGATTGGACAGGCAGGAAGAACAATGCTCAGTACAGAATAGAGCTAGCAGCTGAAGTTCTTATCTCTGTCTTAGAGTGTAATAGAAAAGAAAAGGAAGACCATCTTACCCTTCAGGACAAAATGGCAGACTACCTCCAGAAGCACATCGACAAGATCAAGCTACCTGTTGTGATGGCCAAGACTGCCTATGCCTTGATGCTGTACAATTCAGATTCCAATAAAACTCTGAATGCTGTGGACAAGTTGAAAAGACTGGCACTGAAGGGTGTACAAGGACATATCTACTGGGCTAATAAACCTAAAGATGAGGATGAGAAGAAGCCCCACTGGTACATTGACAGAGTGCCAGAGTCATCTATTGAGGCCACAGCCTATGGTCTACTGGTATTCCTACGTAAGAAGAACTTGTTGAATGTTGACGCTGTGGCCGACTGGTTGGTAGCACAGAGGAAACACAATGGTGCCTTCAATGGGGCAAAGGACAGCACTGCAGCTATTCAGGCACTGACAGAATATAGTTTACAAAAGCACAAAGAGGAAGAGATCAAAGTGAACATGAACCTGACAGTACGTGCTGGGAAAGCTGAAAAGAATCAATACAAGTTCAAGTTTACTCAGGAAAATGCCACACAGCCAGAATCTAGATCTAATGTGCCTGTTTATCAATTTTTAGAAGTGCTGACTGAAGGCCAAGGTCTGGGCCAGATGCAGATAAATGTAGAGTACAACATCCCTGTAGATAAAAATGAAGATTGCTCTTTCAACATTTCAGTGGAAGTCAAAACAGCGAAAATAGCCCTGGACTCAAGCAATCTCTTGTGCTCCAGCTGTGACTTCAACTGCCCAGGGGCTAAAATTAACTACAACATTGATGATACAATTAAAGACAGGACAGCCATCAGTAAAACAGTCTCCATGTTGACAAGTGGTAGGAATCCAAAAACTAATGCTAAACCTAAGACTAAAAAGAATAAACCTAAGCCAACAAAACAGCCAAGAAGAAGGCCCCAGCTCAGTAGAAAGCCTAAGCCTCATGGTAAAAGATCTAAACGTGCATTAAGCAGTGGCAAATCTTACTGCGTGACTGTATGCATTAGGCATCTCCAAGGTGTGAGTAGACCTGTGGATGTCAGGATTCAGATGCTGACTGGAATCAGACCTTTAGATGAAGATGTGAGAAAAATAAGTCAAACAATACCAAATGTCATAGATGCAAGACTAACAGAAAATGCTGAATTTTTAATAGTAAAATTTTCAAAGGTTGAAGCTACCAAGAATACTTGTTTTGCTTATAGAGCAAGAGTTGAGAATGATGCCACAAGAATCAATGGAGCCAACATAGAGATCATTCAGGAAAATTCTCCAAAGCCATCCTGTGTTCTAGAGTACCATCCACCAGAAGACAAAGAGAGTCTGAAAGTGTATTGTGCTGACTACAATCACATCAACAGAGGAGAATGCAAATGTTTTTCAGGCCAGTGTGGAAAGTGTGGTCCAATGACAAGCAGTGAGTTTGATCTGGACAAGACCATCAAACTGACATGTAAAGCTGATTTAGTTTATCAACTCAAGCTAGGCTCTCAAGAAGACAAAATTCATTGGCTAGAGATCAATGCAACTGTTCACTCGGTCAATAAGACAACTGGAACTCATGAGTTGAAAGAAGGGGATGAAATCATCATGATGTCACCAGGTTACTGCATGTGTTTCAGAGACTACTTTGGTAAAGAGGAAAAATTTTATTTACTTTCTTCAGATGTGGATAGGTTAATGGACAGACAAGGAACTATAGTGCATCGCTATGTTCTAGATGAGAACACAACTTTACTCAGAGTTTCTCAGCCCTTGTCCATGGCTGGACACAACAATAGCAAGTCTGAGCAGACTAAGGAAATTTTCATCTCCCAGCCACTGAACAAGCATGAACAGGACTTGAGCCCTCATTTTAAGTTGGCTGTGTCTCATGAGCACTTAGCTGCTGGTCTGTCCCAAGGTGATAAATGTGAGCTTTAG**CCCTTGTCTTCACATCATAAATTCAAAGTCATGAATAACTTTGAAATGTAGAATAATACCTTACTGATTGAAATCAGCATTCTCTGATACACAGATATTTTTGGCTTTGAAAACTAATTATTATGAACCACAACAGATTGGTATCCATAAAAAAATGTATCAGATTCTTTCCTTGATATGTGCAACATTTTCTATAAGGAAAGTCAATCACTTTGTAAATAAATTTTTTTAAATGTCTTCCAACCAATGTCTTTATTATTCCATTAACAGAAACTTATTGTCCAATTTAAGATAAACTGTATATGAAACTTTTTGAATTAAATTTGCAAGAACTAGAATTATGTCAAATTTGTATATTTTAAATGGTTTATTATAGTTCTGTAAAATAATAAAGTCAAATAGATGCCATTGTAAATAGAATTTGAGGTTTACTTTCTAAGAAACTAACAGAATAAGGGGAGACACATTTTAAAGAAACAAGAATTTTTTTTAATGAAATAATTTGTTGATTAAAGCTTTCATTGAGTCTTTTGGTTCTATTTAAGCTAAGCACACTGTAAAGTAGAAGATATCTTACTCTTTATCTAAACACAAGTTTGGTAATGATATCAAGTTTGAAAATAATATTTTAAAGCAAATTTATTTATGTAACTAAAATATTCCTTTCACAAATATAGATATATATTGAAAAATACTATAGAATTTTATATAAGTTTCAATCCCTGAATTCTCATTCAAATGATAAAGAGAAATGTCTGTGGATAATTATCTCATCATAAATATTGCTATTACTTTGACATAAGACCTCTTGCATTCTTTGATCTACTACTATCATATTACTTTTCATTATTGTGTACAATGAAACATTACATTTGTATGTATTGACTCATAATGAATTTTTCATCACATTTAACACTGTTTGTTTACATATTTTTCTGGACTTTATCATATTCCTTTAGCTGAAGTGCTTATATGTAACTGACATGTTTCTTGCTGTTAATCATATTTTGATTACTTTTTTTTGTCCTACAAAATTGTAATATAAACAAAATTTATTTCACAGTTTTATTTTTATCCTAATTGGCATGTTGTAGAAAAGTTCTCTCTAAAATCTTATTAGAATACAAATTAGTAGAAAAATCTTTTTAAAAATGTTTCTGTCAACTTCAGTGCTTAAAAATTCTGCCATGTTTTCAGATTTTATAATTATTTCTGTACTGAGAAAGTTTTTGTTAATTTATTTAAGCTGACCCAGACATTGAGATCTTGAATAAAGGATAACTTTGTCTTTAATGAGAATATCATTGAAACACTTTTGTATTAAACTTTCTTCTTTTATATGTAAGCCTGAGTAGGCTTTATTATTCACCCTCTGCTTATAGTGGTTATCTTAATATTAGATAAATAAACATTTAAAAATTTTCTATTAATTATTAAAATTATATTTGTACTATATATATATTTATTATTTTGTGTCCTATCACTGTGCAAAAAAAAAAATAAACCAACTAATATTTTCATGATATCAATTGTTGAACTAAAAAGAAATGACTGTACATCC

**>BgA2M**

CCACGGCTGGCTTGAATAGCGAGCTTACCTCTCGCTAACTCAGTGAGGACGTTTTTACTAGATGTATATTTCTGACA**ATGGATGTTACGAGTAGAAGCCTCACACTATTTTTTATAACATTGGCCTCACTTTGTCATGCTGAAAACAACTTTCTATTAACATTGCCTAAAGCTATTTACGCTGGAAGTAAAACAGAGTTTTGTCTGACAGCTTACAACGACATCAAAGTGACAATAGATTTCATAAGTTTAAGAAATGTACAAGACACGCCTGTTCTAATTAAAGATAGCTACTCTAGGGGCGAACAAAAATGCACAAATTTCCAAGCTCCACCACAAGGGGAATACAGGCTCGAGGTGACCACACAATCCACAGAACCCGGGGCAGTTAGTGAACTTCACAACTCAACAAAAGTCACTGTTCATGGCAGCAAACTGATCACTTTTATTCAGACTGACAAACCCATGTACAAGCCAGGCCAGAAAGTGATGTTCCGTGTCTTTACTTTGATGAGGAATCTCAAACCAAGAACAGAAAATATAAAAAGTATTTATGTCTTGGATCCGAATGATGTTCGAGTAAAGCAATTCTTGGATGTTGAGCAAAAAGGCATTGGCAGTTTTGAGTTTCAGCTCATAGAAGAAGCCAAACTAGGGCCATGGAAAATTGAAGTATATTTAGATGATGAGGATGAAGTCAGACAGCAAGCAACTGTTCAAGAATTTGAAGTCAAAGAATATGTGCTGCCTAGGTTTGAAGTGCTGATAACTGCACCAAATAACATTTTGATTACTGATAAGAATATCAAAGGCAAAGTATGCGCTCAATATACATATGGCAAGCCAGTGAGTGGGTTTGTTCATATAGAGCTGAAGACATCCACTAACTTGTACAGATATTCACCTTCAGATAGCCAAGAACAAGTTAAGCAGATCTCTGGTTGTTATGAATTCTCATTTGACATACCTGTTGAAAAACACTACACACTTTACTCATATAAACTGAATGTTACAGTCACAGAAAAAGGCACTGGAGTTGTTGTTAATAATGTCTTTGATGGACCGAAAATAACATATGAACCACTGACAATAGAAATTGAAGATTTTACTAAGGGTTTTTTTAAACCAGGATTACCTTATTATGGAAAAGTAACAGTGAAAAAAATTGATGGATCTCCTGCAGAGGGAGAGAAAATTATAGTTAGTACTCAGAATGAATTTCTGTACGCTGGAGAGTTTATCACAGATAGCAATGGCACATTCCTCTTCAGCCTGTGTGAAGGACTGACAAACAACAGATCATCTGTGCAGATCTCAGCTGAGGCTCTGGGTTACAACTCTAGCCGTTACATTACCCGAGGCTTTAAAACTATTCAGCAGTGGTACTCTCCTTCACGCAGTTATGTGCAAATACCACCTGCAGAGGCACAGCTCAAATGTTCCGGTAAAGTGTCTTTGACTGTTCCTTTTACCACTAAAGAGAACTCTTTGGTACAATTTTACTACCAGGTGATTGCTAGAGGAAATCTGGTCAAGTCTGGTCACATAATACACAGTGGTGAATCAACTTATGATGACACTGCTCAGTCTCAGAGCAAATGTCTCAGACAGTTGTCAGAAGAAGAAAGAAATGAAACCCTTGGGTCAATTCATTATTACAGACCATCAAGGGGATATTACCAAGGATCTCATGATGACACATTTCTGAAAGAGGTTGTAACTTCTGACTTCCAAGCTGATAAAGTATCCTTCTTTGTTCTTGACCTGGACGTTGTACCTGTGATGTCTCCCCAGTTTAATGTACTAGTCTATCACATCCTCCCTGATGGGGAAGTAGTGGCTGATGGTAGGGATTTCAGTGTTCAGCCATGCTTTGAGAATCAGGTAGAGATGACCTTCAGCAAGACTACTGTAGCACCAGGAGAGAAAGTGGATGTCTTCATAGGGGCTCAGCCTGCTTCCATCTGTGGGCTAGGGGTTGTGGACAAGAGCATCAACCTACTGGGAGGAAACCATCAAGTGACCCCAGAGATGGTGTTTAAAAAAATTGAAGAGTTTAACTTGGTTCCTCCTCCCGGGGCTGATGAATACTTCAACAATAAGGATTACCAGTACTGTATGAAAAATGTGAAATCTACTTCTGAAGGTCAAGATCATGAAGATTATTTTTGGATACTTTCAAGTCCATTTGTTGATGCACTTCAGGCCTTTGAAGCATCTGGTTTCACAGTGGTTACGGATTTAAAATTAGAGACAAGACCCTGTAGTCGTAGGCCACAAGTATTTTATGCAGGTAAAAGACAAAAAAAACTCCAATCCAACATCAAGTGTCCAAAGGGAGACTTGTGTAGTATGTTCAGTAAACTAGCAGCAGCTCACAACATTCTTATATATCTTAATGCTAAATCCCTAGTAAGGGGTACGATGGAAGAATGGGAAAAAGCTGTCCGTGAGATCTTCCCAGAAACTTGGCTTTGGGACATCAGTGTAGTTGGGGATTCAGGGGCAGTAACCCTCCATGAGACAGCCCCTGACACTATCACCAGCTGGATCGGGAATGTCCTGTGTGTGCATCCAGAAACAGGATTTGGTGCATCACCAGTGACATCCCTGAGAACCTTTCAGCCTTTCTTCCTGTCCCTCCAGTTGCCCTATGCTGCAGTCAGAGGGGAGAAGTTGCCCATTATGCTCACTGTATACAATTATTTGGAGAAGTGTCTGCATATAAAAATGGCGTTAGATATGGAGAAAAACTTTGCGGTGGATAAAAATGAGCTTCTTAAAGAACCAGTCTGTGTTTGTGGTGGCAAGTCTCATACTGTGAAAATATATGTCACACCCAAAGGCCTGGGCTATCTGCCCATTATAGCAAAGGCAGAGATCATCCCTGGACTTTGTAGCAACACCATTGATGTGGACACTCAATACATTGGACTGTCTGATGCTGTGAAGAGACAAATGTTTGTTAAGGCTGAGGGAATTGAGCAGGTGAACACAAATACAATGTTTGTTTGTTCTAAAGTGGACAGTCCCAAGCAGGAGGAGCTAGTCTTGTCTGTACCATCAGATGAAGAGATTGTCAAGGATTCCATCAGAGGGGAGCTCAAAGTCATAGGTGATATCATGGGACCAGCACTGACCAATCTAGACCGACTGGTCAAACTACCAACTGGCTGTGGCGAACAGAATATGGTTGGCTTTGTGCCCAACATTTTTGCTTTGAAATATTTAACAGAGACTAGGAGAATTACAGATGAGATCAAATCAAAGGCTCTGAAGTTCATGGAAGTTGGATACCAAAGGGAATTAACATTTCGACATATAGATGGTTCGTACAGTGCTTTTGGAGACAAGGATCCTCAGGGCAGCATCTGGTTGACTGCCTTCGTGGTGAAATCCTATGCTCAGGCCCAGCCATACATATACATTGATGAGAAGGATCTCCAAGTCAGCCTTAAGTACCTCCATCTGAACCAGCTTGAGACTGGCTGCTACAGGGAAACTGGTAGAGTCTTGGGCTCCTACATGATGGGAGGTTTGAAAGGTGACAACAAGGAGGAAGAGTCATTCACTGCCCTAACTGCTTATGTGGTGATAGCTTTATTGACTGCAGGAGTTAATTCTTCTCAACCAGGTATATATGGAGCAATGGAATGCATCAATGCTGATTTTGACAGTCTGAGGGAACAAATGGATCCTTATGCTTTAGCACTGGTAGCTTATGCCAATGCCCTCTATGCCCCATCCAGTCACAGAACTTCTGAAATTATTGCAGCACTAGAGGCTGTGGCAAGAACAGAAGGAGACTTCAAGTACTGGGCCAGAAAAGATTTTCAACCTAAAGTCAGTAACTCTTGGTACACATACTCAATGCCTTCAGCTGAAGTTGAGATGACAGCTTATGTCTTGCTGACCTATATTAAACTCTTTGGGCCCAGGGCAGTGGAAAGGACTCATAACATAGCTATGTGGCTTTCAAAACAAAGAAGTCCCTATGGTGGCTTTTCATCCACTCAGGACACAGTGGTAGGTCTGAATGCTCTGTCAGAATATTCAAGGCTGGCCTTCAATGGAGGGAAAACTGAATTGAAAGTCTCTATCACTGGTTCAAAACTGAAGCAGACATTCAGTCTGTCACAGAAGAAGAAGACAACATTACTACTCCATAGAGCCTCTATCCCTGTTCTCCCCAATCAAATTTCACTCATCTCAGAAGGTGAAGGCTGTGCACTAGTTCAGTTTAGTGTTTTCTATAACAAACTGTCTAAAGAGTTTAAAGACAAATCTAGCTTTCATTTGGAAGTAAACCCAAGTCACTATAAACCCAACAAAGACAAATGTGATCACAGATCAATCGTCATAAGTGCAGGCACAAAAGGCAAAGCAAGAGAAACATCTGGCATGGTGTTGATTGAGTTGAAGCTTGTCACTGGATGGACACCACTCCCAGAGTCATTGACCAAGATCCAGTTAAGATTTGTGGACATCAAGAAAATAGAATACAATGAAAATGAAGGTCTTATTGCCTTTTATTTTGATCAGTTGAGTGGAAAGCCGATTGAGTTTACTTTAGATGTGAAACAAGACTTAGAATTAGGAGTTTCAAATCCCAAACCAGCTGATGTGAAGGTATACTACTATTATGAGAAAGATGTGTTTAAGGTACAGTCATACAAAATCAAGACTACTTGTGGAACTAAAGAGGAAATACCTCACAAAAACACAGACCCTGAATTTGGACCTGAAGGTCCTAATCAAGTTAGAATCAATCCTGGCATAGATGCACCTTTTACAATGAGTTCCGATGGTTGCCCAGTATGCATTCCAGTTTCTGTATTACCACTTAATTTTAAAGATTTGATCTGCAGATCTAGTGCCGTTTACAAAGTTGCCATCATGAAAGGCAAAACTGTGAAACTCCTACAAGACTTACGCCCACCCAGCCTAGTGAAGAAGATCAATATTGTTGTGGAGCTGGAACTCCCTCCTGGATGTACCTGTGGTCTGCTTACCAATCAAGGTAAAAAGGCTCTCCTGTTAGTAAAGAAACCAATCACAGCTGACTCGACACTTGTCAAGCTAGATAACACTTCAGTGATCACCTTAGAGGACAAAAAGTTCACCAAGACCACCAGAAACACACAGAAAACTTGTCCCTTGAAAAAATTAGAGGAAAAAAAAAAGAAGCATGAGAAAAGTTGA**ACTGTCCAGCAACACTTGAAATGAATTTAACCCCAAAAAATTGTATCCAGTTAAATTATAATTT**A**GAGAATCATGTTTCTTATTTTTTTAGATACATATACTTGTATAATCTTTATGGTGCTTATAATATTTGTAGAAAATGACAAGCTGGCTATAAAACAAATATCAGAGTTCTATCAGTTGTTATTACTTGCAATAGAATTTTTTTTGTCATTACTTCTTTGCCTTGAAGAATCCTTGTTGGTGTTTCGATAGATCACTAATGTTGGACATGCCAGGCATTACAGGCAGTTCAATCAAAGTGATCTAATAGAAGTCTTGCTGTGTTCTGCTCTTAGCCATGCTTCCCTTCCCAATGTTTGAACTCATGCCACTATTTGTCATTAGATCAGGTCTGTGACTTCTGGAGTTCCTTGTGTTTCATCAATTAACATGACAGCAAGCCATTGTCTAGTGGCATGTATACAGCAAGCCACATTGTCTAGTGGCATGTATATATACAATGTAATTGAAAATTACTGTAATATTTAGACTTTAAAATAAAAGTTCATTTTGTTTGTTTTGTTGTAAAGTTATATAAGGAATAGGGTAGAAGCAAACAAGAGAAATAGGATAAAGGCACACACCCAAAATACATTTCTTATTTCAGATCTAATCTTTGATGGGTTATTCAGTAAGATAAATAGTGAATAAATTGTTGATTTTTGTTTCATAATTTGTTTAAACAATAACATTTCCTTCCTTGTGTAAACAAATAGAAAC

**>BgMCR1**

CGCACTTTCTTAGAAGAAAACCTAAAAATTGTGGGCATCAAAA**ATGTGGACGTCGCTTGCCGCCCTATTCGACGAAATGCGCAGAAAGATCGAAGTTCAATCTAGAAATATGTGGCATCTAATATGCTCAACGATCCTGATTGCCATGGTGTCAGCACAACAGCCAGGTGGTGGTCCTCCAAGAGAAGACAATTGTTTAATTGATGCCAGTGTAGGATGTTCATATGCAAGAGCTCCGCAATATCTTGTTATTACACCTAAAAAGATTCGACCCAATCAAGTTTTTCAAATATTTGCAACCATTTTGAAGATGGAATATAATCAAGAGTTTGTTCATGTTATTGTGTCTATAATAAAAGATAACATTGAATATGCCAACACTGCTTTACGTTTTGACAGGCCATCTAGTCGTATTATGCAGCTTCAGATGCCTTCAAATGCTCAAGAAGGAAAATACAGGCTTAGAGTTGAAGGACGTCTCAACGAGCAAGACACTGGAAATATTTGGCAGAATGAGACAGATATTGATTTCACTACTAAACAAGCTTCATTATTTTTACAAATGAGCAGGCCCCTTTACAGACAAGGTCAAAAAGTGCATTTCCGCATCATACCAATATTACCAAACATGATGCCTAAATATGGAAGCATGGTTATCTATGTTGATGATCCAACTGGAATCCCTGTCAGGAGATGGCAAAGCATTCAAACCAATGCTGGTGGCATTATAAGTCAGAGTTTCACATTATCAGATCAACCAAATTTTGGAACTTGGATTATTAGAGTGGAAGCTTTTGGTCATGTTTATAGGCATCCTTTTACGGTAGAAGATTTTTGGGAACCACGTTTTGATGTAAATGTGTCAGTTCCATCATATGTGATGGAAACACCAGAAATTTCAGTTGCTGGTGTCATGCTTGCTAATCATACAAGTGGACGACCTTGTATTGGTAATGCTTCTATTACTGCATTCTTCCGACCACGAGAAGAGATTTGGAATCGAACTAAGGGCTGGGAAAAACCTTACTGGGATGCTCAAGCAGGAGGCACACCAAATTCTGGTGTACCAATGGAATTACCTCAATATAAACCTGTTCATACAATTCCTGTTAAAGATTACTACATGTACTTTGCCTATGAATATAGATTTATTGATTATTTTCAAGGCCGTATTGACTTTGAATGGACTCTTGAGCAGCTCATGACCATTGCAAAACGTGGAGGTGAATCTGGTTCTTTGGTTGACAGTGAATTTGTTTTCTTTGCCAATGTATCTGACTGGTATTCTGGCCTGAATCGTACAGGTTGGGCTGGCACTATCTTTTTTGACTCTAAAATCAAATTAAAATGGGTTGGAGATCAAATCAGAACATTCAAACCTTCTCAAGTTATGAGAGTCCAGGTGGCAGTGACAAAATATGATGGTACTCCTGTAGAAAATGTTGGGACTGTGACACTTACTGATGTTACCACTGATAGCTCTGGCACTGCTGTTCAGTCAAAAAGTAACACTCAAGTTCCCAAAAATGGAATAGCTGATTTTGAGTACCAACTATCAGCCACAACACAAACTCTCAAACTAACAGCAACATATGCGGATGCAAGGAAGCCTTACAGTGGTGACAACAAAATTTATTTGGATCCTCGATTCAATTTAGGAACTACTGTTCCAATAACGATGTATGCTACTCGATATTATTCACCATCTAATTCATATATCACAATATTGACATCCACTGATCGACCTCAGGTTAATGAATACATGGTTTTCCATGTGAAGACCAGTAATTATGTACCCAGGATCTACTATCAGATTGTAGCACAAAGTAACATAATCATAGGAGATTGGCTTGAAATGACTTCAAGACAGAAAACATTTGCTGTTGCCCTATCTCGTGATATGGTTCCAACAGCACGTTTAGTTGTTTACTACATTAGACAGCCTGAAGAAATTGTTGTTGATGTTTTAAACTTCTTTGTCAATGGCACAAGGCAAAATTTGGTAACCTTGAACATTAATCGTGGTAAAGACTTCAGCCGTGATACTATTGAATTTAATGCCTATGCTGACCCAGGCTCCTATGTTTCATTCAGTGGGATGTTACTGGATTTGTATAGTAGAGGATTAAGTGATGGTATTACAGAAAACAAACTTATTGATGAACTGATGACATATGACTCAACACAAAATGGCAGTTATCGCCATCTGTGGAGAGTCAGTGATACAGAGTATGAGTATGTGTTTTACCATGGTCCTGATTATGGCATTGATGCTAACACAAGTTTTGATACTGCAGGCCTGCTTGTTTTAACTGATGCTAGAGTATCAAGACTTTACAATGATAAATTTTGCACAGATCAGAAAAGTTTCCCCTGCTTTGTTGGAATAGAAAGTCAATGTTTCAAACCTGAACAAAGATGTGATGGAACTATTGATTGTGTAAATGATGGTGCTGATGAATGGGGATGTACATTCCAAGAATCCAAAGAAATACATAACCCAGCTATGGACAGAGTTAGTAGAGTGATGCGTTTTTATGATAACAGTTCGTGGGCATGGCAAGAAATATTTGTCAAGCCTGATGGTAGAGTGGATTTCCGTGTTGATGTACCTAAATATCCTCTGTCATGGGTAATAAATGGGGTATCTGTCAGTAGAGAACTAGGACTGGGCATCATGCTTAAACCTGTTAGGTATGATGCTGCCAGATACATGTATATGCAAGTTGAACATCCTAAACACATTATCAGAGGTGAACAAGTCGGTGTAAGAGTGACAGTATTTAATTACTGGTATGATGATGACTACCTTGAGGTTTTGATAACTATGCATGGAGGCGATGGCTATTCTTTTGTGACTGTTGGTGAATATGGTTATGTAACATCTTACACGCCCCCCACTCATAAAGGAGATCATCAGACTATAGTCTTTTTAGAGCCTGGTGAGTCCAAAGATATTTACATGCCCATTGTACCAGATGGTGGTGTGGTTAGAGGTCAAATTGAATTTAAGGTTTCAGCTAGTTGTTTTATGCAAAAAGATGAATATATTGGAACCATGTATGTCAAGCCTGATGGGGTCATGAATTACTACCATACACCTTATCTAATAGATCTGATTAGATTTGGTTCTATTCAAATTCCTCAATTTGATGTGCCTGTGCCTGAACAGTTTAGAAAACTTGAAGTTCGTGAAAATCTTTATATCCCTCAGAGTCCTGAAGCAGTAGTGTCACTTTTTGGTGATGTAGTGACTCCTGGCTTTTTCCAAGATTATCTCAATGCTGAAAATATTCTGTGGAGACCATATGGAGGAGGAGAAATGATAGTATTTAATTTTGCTTACAATTTATATTCATTGAAATTTATGAAATATTCCCAGCAGTTGGATGATGCCCAATTAAGTAAATCTTTACAAGAAATGAATATTGCATTTCAGCGTATTTTAAGCTACATGAATGCAACTGACGGTTCTTTCAAGATGTTTAGAGATGATCCAAAACCAAGTTTATGGCTTTCTGCTTTTGTTGTTAAAATTGTCAAAGAAGCTACTTTTGGTGAGTGGGAGCGTGACCTCTTTATGCCCAGAGAATTAATCAACAAGGTTGTCTTATACATCTGTTCTCGTCAAAATGAAACAACTGGAGCATTTGAACCAGATGACATTGAAGCTACATATGATAGAAAAATGACCCTTCTAGAAAGTTTGAAGGGAGATAAACTCCATGCACATCCTATTCCCTTAACTGCATATGTATTGATTGCATTGAGTGATTTACAAAACTACGTCTCTGAGGAAGCAGCTGCATGTCGAGACACAGCTGTACGTAATGCAGCTAATTATCTTTTTAACATAGTGCCAGAAATCAAAGAAGTTTTTCACATGGCCATTACGACATATGCACTTTCACTGACACAGAAACGTAGCCCATTTGAAACTTTGTGGAAAATGAAAAGAAATGATTCGGACTTTCTATATTTTAGTGAAGAAACATCTTATGAAAATCCATATGACTTTTTAAATAATGTACGATACCTAAAGCCACGTCAGGAGTTGATGAATGATGCCCATGCTGTTCAAGCAACAGCTTATGCTCTTATGGCACACATGAACAGTAACTTAGGAACCAAAGTTGAGCGTGAGATGATGATGGCTTGGCTAAACACAATGAGGAATTCTATTGGAGGTTTTGCTGCTACTCAGGATACTATACTGGCAATGGAAGCTCTTCTTAAATTCACACAAGTTGATCCACATCGTAATGTATTTGACTTGAGCACAACTGTCGAATCAACTTCTTCACCTTCATGGGCTGCCACTTTTAATTTAAAAAAATTAGATTATATTCGACTGAAGACTAATAGTCTTCCTGCAGATAAAGTTTGGGGTTTTATTGTTCCTAATGCTCAAGGAACAGGAAGAGCCTTGTTACAATTAACAACTACTGTCAATGTGGAATATATGTGGCTTCAAAAAAAGCCAATGCGTCCTAACAATGACCCAAATGAAGAGCCTATAAAATTTTTTGATCTCATTGTAGAAGACCTTAGATTTAGTGGCAGAAACGACTCTATTATGGAAATGACTAATTGTGTTAGCTGGCTTTACACTGAAAAGAGTTTAACGTCTGGCCTTGCAGTACTAGAAGTAGATATTCCTACTGGATATGTTGTTATGAATGACACTTTAAGGGACTATGTACGTTCCAATAGAGTTCCCAGCCTAAAAAGAGCTGAACATTATGATAGAAAAGTGATCTTCTATTTTGAATATCTTGATGAAAGTAAAACTTGTGTCTATTTTCGAGCTGATAGATGGTTTCCTGTTGCCAATGCTACTATACAGCATCGAATGAGAGTTTATGATTACTATGAACCTGGTATGCATAATACAACATTATACACAACACGTAACCTGTTCCTGCTCAATATTTGCTTTGTTTGTGGCTCCTACCAATGTCCCTATTGTCCATACTTTAATTCAGCTACAATAATGAGTGCTGGATTCAGTGTAGTTTTATTGATGGCTGCTACCTACTTTGTTCAGAGACTGCTTCTTAGAAATAGATAG**ATCTAATTTATTTTTTTTGGTATTTTGTCATTTTACATTTTTTTTAAACAATGGAACCAATGTACAGCAATATTCATACAGGTATGGAAAAAATAACAGATAAATGTGAGAAATGTTTGTAATTAATTTCATTCTGTACATTAAAGCTCCTATCAACATTTATCCATATATTTTCTCTTGAGTTTTTATACATTCCAGTTTGAATGACAAGGAAGTGGAAGTAACTGTTTTGATTTAACCTTCAAATTACCTTTTGTCATAAAAAAAAGTACACTTTTAAAGGATTTTATTTCCTTTCTGCAGCATTTTCTGCAGATATGAGTTGTCAATAATTATTTTTTTTAGCATTTTGTCATTAGTGAGCTGATGGAAATACCTTAATATTTTTCTTAATTATGAAACATTAGTATGACTTTTACTCTAAAACAAAAGTTAATGGTTAGATTGAGGCCACACAAAGGGGAAAGCCTTGATGTGTACCATTATCTTTCTTGTTGTAGAAAATATTGAATATAATGGCTTTAAAAACCTTGGCTAAAAAAAATTATTGAACATTGTAGATCAACAAATTGATAAATCAATATCTCCACACTTAATTAAAATATTATTTTTTTTGTACAGAAAACAAAATGTTTTTTATTGTCTGAAAAAATGACATTTTTTTGTGTATTGTAAATTGTATGTACTTGTGTAAATATGAAGATATTCAACTATATTTTTGAATATGTTAATTTTACATTCCATTAACCTCATTGTGTTTGATATTTTATCAACCTGATATTGGAATAATATACCAGCAAGTGAACTTTTTTTTTTCTTAAATTAAAAATGTTAGAATCGTTGTTGTATATTCAAATGCATCCTTTCATATCATGCCATCATATGTATAGTGATGCTGAGTAAACTACTTTATCTGCAAGTA

**>BgMCR2**

AACATTATCAAATGGGGTCAGCTGAAAATGGCGGAAAAGCTTTCTTTCTTATTATTTTTGAAAAATAGTTTCGTTAGATGTTGATGCAAAACTTTGATTTCAAGCCAGAAGTTGAAAACATTTGATAGTTTTGTCCTTCTGGATAAGTGAATTCTTCAAGCAGTAACA**ATGTTTTTTGTCATTCTTATTTTGTACTTCGTTTCACTAGCTATTGGTCAGTTAGGTCATGAACATTGCTTGATTGAAAACTCTTCAGGATGTGAGCTTGGGAGACCTCCCATATATATGATAACAGCACCAAGAAGAATTCGTGCTGGACAAATGTTTCAAGTTTTTGCTACAATTTTAAGAATGGAGTATCATGAAAATGCAATAAGTGTGCGGGTATCCATCGTTGAAAGTGATAAAGAATATACAAGCAGTATTTTAAAATTTGAACGACCATCTAGCAGGCTTATGCAACTACAGATGCCGTCCAATGCTGAAGCTGGGAATTATAAACTTCGAGTTGAAGGTCGTCTAGATGAACTTGTTAGTGGCAACATCTTTTTCAATGAAACTGAGATTGAATTTACACCGAAACACGCATCAATTTTCATACAGATGAGCAAACCAATTTACACACAGGAACAACTAGTTCATTTTCGTATAATACCACTTCAGCCGGATTTGATGCCTAAATATGGAAATTTAATTATTTATATTGAGGACCCATCTGGAGTGCCTGTCAAAAGATGGCCAGGTTTACAGACTAACGCTGGAGGAATCATTAGTCAGAGCTTTCAACTTTCTGATCAACCAAACTATGGCACATGGCATATACTAGTTGATGCTTTTGGTTTCAAGTACAGAAGACCTTTTATTGTTGAAGAATTTTGGGAACCAAGATTCGACGTGAATGTGTCAGTGCCAGCTTATGTAATGGATGTATCAAGTTTATCTATTCAAGGAGTTGTGTTAATAAACCATACAAGTGGAAGATCTTGTGTTGGAAATGGGTCTATAACATCTTTTTTTATTCCATCTGAAGAAATTTGGAATGCAACAAAGGGATGGGAGAAACCCTATCATGACGCTATGAGCAGACGTCTCTCTGGAACTCCTCTAGATGTACCCAAATACAGGAGCATTGACAGTATTCCTGTGTCAGACTACTCTGTTTACTTTGCTTATGAATACAGATTTATTGATTATTATAAAGGTCGCATAAACTTTGAATGGCATCGTGATGATTTGTTAGGACTGGCGAAAAGAGGAGGAGCTACAAGCGATGAACTTTTTGGAAATGAGTTTGTGTTTGTAGCCAATATGACTGACTGGTATTCAGGTTTGAATCGCACTGGCTGGGCAGGAACGATTGTATATGACGATGAACTGAATCTTAAATGGATAGGAGGAAACGTCAGAACATTCAAGCCAGGCTCTTTATTCAGAGTTCAGGTTGCTGTTTCATACTATGATGGTAGACCAGTGAGTGGTGGCTCGGTTACTCTAGTACCAACTGTAGGTAGCCAGTCGGTTAAAACTGCCATGTCGGAATATTCTCCTCAAACGAATCCTGTTGTCAATGGAATTGCTCACTTTCAAATACCATTAAATGTATACGTGACCAGACTGGCATTAACAGCCTCATATCATGATCCTGGCGAGACTTTAGCCATGAAAACTTCAGTGAACCCAAATTTTGTCACCAGCCAGTCAAGAAGTATTCAGATGCTGTGCACTAAATATTATTCCCCTACAAACTCATATCTATCTATTTACACTTCTACTTTTGAGCCTCAGATTAATGAATATATGATCTTTCATGTCACATTGAGTCATTTTGTTCCCAGAATTTATTATCAGGTTGTGGCTCAGAGCAATATTATTATCGCAGATGAACTGGAGATGTCAACCAAGCAGAAGACTTTTTCTGTTGCACTTTCAAGAGAGATGGTTCCCACAGCCAGAGTTATAGTTTATTATATCAAAGAGCCTGAGGAGATAGTATCTGATGTTCTCAGTTTTTTTGTAAATGGCACTAGACAAAATCAAGTGTCACTCTACATTAACCGTGGTAAAGATTTCAGCAGAAATACAGTAGAATTTAATGCATATGCTGATCCTGGTTCTTATGTGGCATTTAGTGCTATGTTACTGGATCTTTACTCTAGAGGAATGAATGATGGCATCACAGAGAATAAGCTGATAGATGAACTGTTAAGTTATGACCAGCCTGCCAACAGTAGTTTCAAACACCTATGGAGAGTCAGTGACACAGAATATCAATACACTTTTTTTCATGGTTCAGATTATGGCATTGATGGAAACACAACATTTAAGAGTGCTGGAATCATCATTATCACTGATGCAGATGTCACAAGATTACCGAACCAAGAATCGTGCAATCCATTAGATGGAAAGTTTCCTTGTTTCTCGGGTGTGGAGACAGAATGTTTTACTAGTGAGCAACGCTGCAATGGACTGTTCGATGGATGTCCAAACGATGGTGCAGATGAATGGGGATGTATATTTAAAGATATGGAAATTGATTTAAAGTCTCCACTACAGAGGATAAGTAGAGTCATGCGATACTATGACAACAGTTCTTGGGCATGGCAGGAAATTTTCATCAAGCCAGATGGGAGAACAGATTTTCGCGTGGATGTTCCCAAATATCCATTGTCTTGGGTAATTAATGGTATCTCTATAAGTCAAGAATTAGGATTTGGAATTATGCAGCAACCATTAAAATTTGATGCTTCAAGATTCATGTATATGCAAGTAGAATATCCCAAGTATATAGTGTGGGGAGAACAGATTGGTGTTCGAGTCACTGTCTTTAATAACTGGTATGATGATGACTATATGGAGATACTAGTGACCATGCATGAAGGACAAGATATTGAGTTTGTTAGTGTTGGAGAAATGGGCTATGTCACCTCATATAGTCCTACTACACATAAGGGAGATCATCAAACTATTGTTTTTCTGGAGCCAGGTGATTCTCAAGATATTTACTTGCCTATTGTGCCAGCTAAGTCTTTCAGGAAAGATAAATTAACTTTTAGAGTAACTGCTGTGTCTTTTATGGGGAAGGACGAACATATTGGAGAGATGATTGTGAAGCCAAATGGAGTACTGAACTATTATCATACACCCTATCTGATTGACCTGATTCGATACCCATCGATTGATTTGCCCCAGTTCAAAGTCAATGTTCCAGAATTATTTCGTAAGCAGGAATACCGACCTAATCTTTATGTTCCACAGTCACCTAAAGCTACAATAAACATTTTTGGGGATGTTGTTACACCTGGTTTCTTTGAAAGTTATTTAAATGCTGAGAATCTATTATATCGTCCATATGGTGGTGGAGAAATGGTGACATTCAATTTCGCTTATAACGTGCTAGCATTGGTGTTTATGAGAAATTCCAACCAGTTGGATGCTTACCAGACCAAAACAGTTTTGAATGAATTAAATATTGCTCTGCAAAGAATATATAGCTACATGAATGAGGATGGATCCTTCAAAATGTTTCGTGATGATGATAACTCTAATCTCTGGCTAACTGCGTTTGTTGGAAAAACCTTGGCTGTTGCAGGTGAAGAAGATTATTGGGAATTAGAACTATATATTGCCAAAGAATGGGCAGCTAAAATAGTAAATTTTATTTGCAGTAGACAAAATACAACAACAGGAGCTTTTGAGCCATTGGAGAATGAAATAGCTTTTGACCGAAAAATGGCATCTTTGAGAAAAATGAAATCTGATGTAATGATAACACAAACAGTACCTTTAACAGCTTATATTCTTATTGCGTTGGAGAAAATGTCTAAATTTGTTGAGGGGACAACTTGTTTAAATACTGCCAAAAGAAATGCAGTCAAATATTTACAAAGTCAAGTCAACTCATTAAGCAAGGACGAAATCTTCTACATGGCCATAACAGCATATGCTTTGTCTTTGACGTCTAATGCTTTTGACATAGTTAATGAGCTGTGGAAACTAAAGAGAAATGATTCTGACTTTACTTACTTTGCGGACCAACTCGTCTATGAAAATCCATCCGCTATACAAAACAACGTAAGGTATTTGATGCCTCGCCAGGAACTTCTCAATGATGCTTATGCTGTTCAAACTACAGCCTATGCCCTTCTGGCTCACATTACAGCAAACAAGGCGGATAAGTTAGAGCGTGACATGACTATGACTTGGTTGAACACGATGAGAAACTCTTTTGGAGGGTTTTCCTCTACTCAGGATACAATAGTGGCAATAGAAGCACTAATGGAATATACTAGACAAGATCAAAAGCGTAATGAATCAGATATGTCAATAGACCTTCAATCAATGGCATCCCCTGGCTGGAAGAATAGTGCATACATTGTCAGAAATAATTTCACTCAACTTTATCAAATTCCTTTGCCATTGAATGAAGTATTTGGCTATGTAATACCGTCAGCTAAAGGTGTTGGCAGAGCGTTGCTTCAGCTTACTGTCACATCGAATGTCGAGTATGAAGAACTTATGAAAACCCAACAACACTACAACAATAATCCACAAGAAGACTTAATTCCCTTTTTTGATTTGCAAGTGGAAGCACGTTGGGGTGGTCGCAACGATTCCATAATGTTCATGAGATCTTGTATCAGCTGGCTGTATACTGAACGTAGCTTGACCTCTGGCCTTGCTGTACTGGAAGTTGACATGCCCACTGGCTATATTGTCATGAATGACACACTGAGATCCTATGTGCAGTCTCGGGTTGTGCCTAATTTAAAACGAGCTGAATTTTATGCTAGAAAAACTGTTTTTTATTTTGAATATCTGGATACAAGTAAGACCTGTGTAGATTTCAGAGCTGATAGATGGTTCCCAGTTGCTAACAGTACTAAGGAGCACAGGATACGCGTTTATGATTACTATGAACCTGGAATGCACAGAACAAGACTTTATACAGTGCAAAATCTATTTCTTATGAACATCTGTTTTGTTTGTGGATCATATCAGTGTCCCTATTGTCCATATTTTAATAGATCAGTTTTAATTATAGGTAGTAACTGGTCTTTATTTGTAGTAGTGATGTATATAATACACTTATGTTTTCAATAA**TATATAATGACCAGCAAGATCAAACCTTAATTATCAATAATCATAACGTTTTTGTGTGTTCGGTAATATATATATATATATACAGGCCTTTTTTTGCGACAGTACGCACCGGTACGGCGTACCGGCACCTTTTTCAATGTGAGGGAAAAATTATACATTTTCTTGTATTTTAACGTATATTATTATTTTTTACAAA

**>BgTEP1**

AAAATATTTCAGTGATTTTCTTCTTGTTTTAAATTTGGAAACATTTCAGCAATTGGTAAATGCTCTACCTGAACCAGGTAATAGCAAAATACTAATCCCGAACACAGCGGCCAATTACAGTAACAATCATACTGGATTTAAGA**ATGAAGCTGAATTTGATTTTATTTGTTTTTTATCTTGTCTTTCAAGAGTGCCAGGGGGGAAAATATTTTATTTCGGCACCACGTAATGTTGTGCCTGGCACTGCATATGACATTAGTGTTGACATCTTGAAACAAGATATTGACAATGTAACAGTTGAAGCAATACTACAAGATTATAGCTTCTCTATACCCGAAGGCCCAAAGAGTTTACTAACTGCAAATGGCACATTTTCACCAGGAGTTAGAGGGACTCTGTCAATGCCAATTGACTTCAACTTACACTGTTCTTACTGTCGAATACTTTTAAAAGGATATAATCCACTTCAGTTTGAACAAGACATTTTTATTCAAATATCTTCTGATATTTTATCAATATTGATCCAAACTGATAAAGCCATCTACAAACCTAAAGAGAGAGTTAACTTCAGAATCCTGGCAGCATATTACAACTTACAACTTTACACTGGAACATTTCATTATGAAATTCTGGATCCTTATGATAATAAAATCAATGTTTTGAGTGGAGTCAGCGGAACATTTGGTGTAGTGGAAGGTTTCTTTGATTTAAGTGATCAGCCTTCTTTTGGAACATGGAAAATAAATGTCAGAACTGAGACTGTTTCAGGAGCGGAGAGTCAATTTTTTGAAGTAGCAGAGTATGATTTACCAAGATTTCAAGTGGATGTTGGGTTACCTCCTTTTGCTTTACTTTCTGATACAACACTATCAGGCTCAGTAGAGGCCAAATACACGTTCGGCCAGCCAGTATATGGTTTAGTACTACTTCAAATTGGTGAAAATGTTGACACAATAGACAAATGCAATGTCAACAGAAAAGTGACGGAAATTAGTTTTGAGATTAAAGGAAAAGGAAACTTCAGCGTACCTTTGGAGGATATTCGGAGAAGCGTTCATCTCAATGAGAAAAAGAAAATTAAAATCACAGCTTTCGTTACTGAGGCATCAACTGGCATTAAGCTAAATGGCAGCTCGGTCATTACATACTATGGAAACAGATATCAGATCAAATTTTTAGAAATGACTCCAGCTGTTTTCAAGCCAGGTCTCCAATATACTGCATACGTTCAAGTAACAACTCCAGATGGACTACCTCCAACAGATTCAAATCTTTCTCTCAGTGTATACACTTCAGTCACTTATCAAATGACTGTACCAGATCAGGAGTTGTACAGTCCTTCCAGCTTCTCTGGATCTTATCCACTACCTGGACAGAACATGAGCCTGCCAGCAAATGGAATACTGTCCATTGATATAGATATCCCCTTGAATGCTACGTCTATTGATATCAAGGTGTCGTTAAACAAAGAAACAACTGCCGAGAAACGTATTTCTAAATCTTATTCAATGAGTAACAATTATTTACAATTGTCTTTGCTGAGTAAACTGGTAAAAGCAGAATCAGATGTACTTATTAAAATAACATCAACAGAAGCAATCGATTCATTAGCATATGAGATCAGATCTAGGAGTGATCATGTGAAGTCTGGAGTATTGGAGCTGAGCGGTCAGAGAGAGTTTAACGCTACATTCAAAGTTGAGCCTAGCTGGGCTCCCATAGCACAGCTGTTGATGTATTACATTAGACGAGACTCCAATGAAGTTGTAACTGATAGTTTGGCTTTTAATGTGGAGGGAATGTTCAAGAACAAAGTAAATGTAGCCTTCAAAGAAAATGAAACTGACATTAATAAGAATGTTTCCCTTGAGCTGTCTGCTGACTCAGATTCACAAATCTATGTCCTGGCTGTGGATCAGAGTGTCCTTCTTCTGAAGACAGGAAATGATTTAACACCAAATAAGGTCAAAGATTCATTCATCTCCAAGTTTCACAAAGGAGCAATTCCTACAGATTCAAACTTTGCTCTTTCCTACAGTGGAAGTTCAATCAATGAAGTATTCTCAAATATGGGCTTAGTGATTGCAACAGATTTAAATATATTTGCTCCTTTCAGGCCTATAGCACTTGGAAGATTTCCCTCAAGTGGCTTTGATAGACAAGGCATGATGGGTGCACCAATGGCGATGTCATTTAGAGATGACAATGCAATGGAAAGCGCTTCATTTGAGATGGATGTAGCGACATCAACTAAGCCTGTTGAGAGAGTCAGAAGCTTTTTTCCAGAGAGTTGGCTCTGGACAAGTGTAAAGAGCATCAATGGTCATGCCACACTTACTACCACAGTACCAGACACCATCACTTCGTGGATTGTTAGTGCCTTTGCAACTAACTCGGACACAGGTCTCGGCGTGGCACCAACTACTTCAAAGCTTCGTGTGTTCAGACCATTTTTTGTAAGTCTGACTTACCCTCGCTCAGTCACACGGAATGAACAGTTTATTGTCCAGGCCACAGTTTTTAATTATCTTCCAGTGGATCTAATGGTCACTGTCTCTTTAAAAGAAAACCCATTCTTGACACCTATCACACCTGGACCTGGAAATCAAGCATCAAATATTCAGGTAAGAGCCAATGAACAGGGAATAGTGTATTTCTCATTATCAGCACTGACTGTGGGCTCTTTGGACATTGAAGTTTCAGCTAGAAGTAATATGGCAGCTGATGCTATTGTAAGACAGATTCTCATCAAACATGAAGGAGCTCCTGTTGTTTACAACAATCCTATTTTAATAAACTTGTCCAACAACCAGTCTACTTTTGAAAAAAATATTGCCTTCACACTTCCTGACTCTTTGGTTCCAGAATCTCAAAGAATTAGAGTTAAAGTGACAGGTGACCTAATTGGATCAACTGTACAAAGTCTCACATCTCTTTTAACATTACCAACTGGGTGTGGGGAACAAAGCCTGGTCAAGTTTACACCTAACATCCACATAGGCAGATATTTAAAGGCTACAAATCAACTATCCGAAGAACTGAATAAGAAGATCATTGATCTGTTAAATGATGGATATCAACGGCAACTAACCTACAAGAGATATGATAATGGTTTTAGTGCATTCGGAAACTATGATATAAGTTCCAGTACTTGGCTAACAGCTTTAGTGGTAACATCATTTGCTGAAGCTCAAGAGTTTATTTTTGTGGATAAAGAAATCATTCTTAAAGCATCAATGCTGTTAATAGACAGACAAAATATTGATGGATCCTTCAATGAATTTGGTAAAGTTTTAGACAGAAATACTCAGGGAACAACTGCTGGTCCAGCCTTAACTGCATTTGTATTAGTTGCACTGCTCAAAGCCAAGGAGCTTGCTGATGTTCAGGATTGCAAAAACAATAACAAGTGTAGATATTATCTATTGGGTAATGCGACTCTAAATGCTACAAGAAATTTAGAAAGGCTGATGCTTGCTGATTCAATTGATGACCAGTTTTCTCTTGCTGTGGCCAGTTATGCTTTTGCTGAAGCAAAAAGTCAACTTGCTCAATCAACATTTGAAAAGTTATTGACATTTGTTAAACAAGAAGGAGGTCTTGAGTACTGGAGTGCCAACAGTACAGTGAACAATGAAGAATTAAACAGATTTATCAACTGGCGCCCTCCTCGTCTTCAGGCCAGACCCATTGACATTTTGATTACATCATATGCTATATTGACCTATAGTTCTCTGGGGCGTCTTGATGAGGCTCTTCCATCTGTCAGGTGGTTAACATTACAGAAAAATGCACAGGGAGGTTTTGTGTCTACTCAGGACACAGTTGTAGGACTACAAGCACTTTCTTCTTATGGCAGCAAGTCATTCAGGCCTGATACTAACATTACCATTTATGTGTCAGACATGAACACACATCTGACAATGAATGTGAACAGTGAAAATGCTTTATCACTACAAATTCAAGAGATTCAAAGCAATAGTCAAGATTTTAGTATCACTGCTTCTGGTTCAGGCCTTGCACTTTTAGATATTGAATACAGTTTTAATGTCCTGAAAGAATTGTCCAAGCCTGTTTTTGATGTGAACACAGTTCTATTAGATGACAAGTTGGATTCTTTTAATATAATGGTCTGCACTAAATTTTTGATGAAGCATGATACAGGCATGGTTGTCCAAGAACTTAGCATTCCTTCAGGGTTTGTGCCAGACTTAAGTACTTTGGGACAAGTAGCAGGTGTCAAGAGATCAGAAAGAAAAGGCTCTATTGTAGCAATATACTTTGACAAGATCAGTGGGTCTTCTCTGTGTTACAGTATTGTTATGACCCGTGAAGCTAAAGTAGCTAAGTCCCAGAAGAGCTATGTCAGAACCTATGATTATTATGAACCAGCTAATCAAGCAACAGTGTTCTATCAGCCCCGAACTTTAAGAGACTCAACTGTCTGTGATGTTTGCCTCAACTGTTGCCCATAG**ATGAAGAGGACACAATAGGACAAACTTCCAGCACTAATCATGCTAACTACTAAACAGAATAAATCATTATTAAACTGTATAGAGGTTGTTCTGTGTTATTTTATTTGAACTAACAATGTTTACAATGCAC

**>BgTEP2**

TTATACCGCAGCAGACGGACCTAGCTAGGTCACTGGAAGATAAACAAGGCGAATTAGTGTGACAATGTGCGCGAGAGCGCTGTCCCTTATATAAAGAGACTCGTACCAGGTGTTCGCCCACTTAGCCTGCCTTTCAGTTTGAGTCAGGTAAAGCAAG**ATGTGGAAGTTAATTCTATTGGCGGTGGTTATTGCCACTGCTAGCGCAACAAACTCCTATGTGGTCATTGCACCTTCCAAGGTCCGAGCCAACATGGACCTGTCCCTCAGTGTCAACATCCTCAACGCCACTGGTGATGTTACAGTCGTAGCATCCCTTTTACGTGAACAAACAACAGTGGTCAGTGCTACGAAAGTCTTTCAAGAAGGTTCACCTGGTACACTAAATATGAAGTTGCCAGCTGACCTTCCCAGCAGCACCTACACCCTGAATGTAAAAGGAAGTGGTGGACTGACCTTTGACAAGTCAGAAAACTTGAACTACAACAATAAGGAAACTTCAGTCTTCATCCAGTTAAACAAGGCTATTTTTAAACCAGGGGACACAGTCAACTTCAGAGTTTTTGGAGTCTACTCTGACCTGAAGTCTTACACAGACCCTATTGATATCTCTATTTATGATGCCAACTCCAACAAAATCAAGCAATGGCTTAAAGTGACTCCCACCAATGGCGTGATCACACAAGAACTGACTCTGTCTACACAACCAGTACTTGGAGACTGGAAGATCTCTGTTGATGCTGGTAGAACGAAAGAAGAGAAAGTGTTCACAGTTGCTGAATATGTTCTTCCCAAGTTTGAGGTGGATGTTGTTATGCCATCCTATGCTTTGACTACAGACAATGACGTCACAGTGACAGTCAAATCTAAATACACCTATGGTAAACCAGTCAATGGTACTGCTGATGTCTTGGTCAAGTTACATGAAAGCTTCAATACCTTTGACTACAGCAGAGCCCTCCCAGTAACCACACTGCAAGTGCCTCTGAATGGTGAGGCCAAAGTGACCATCCCAATGTCCCAAGTGAAAGCCATCAATCCATATTTAAATCAGCATGTTTTGATAGTCATTGCCAATGTGACAGAGTCTTTGACAGGCAACCAGATGTCAGGAAACGGAACTGTCACGTTGTATGACAAAGGTGTCAAGTTGGACTTCCCAGAATCCAATCCTAAAACTTTCAAACCAGCTCTTCAGTACATCGCCTACCTTAAAGTCACCCAACCCGATGGTCTTCCAATGACCTCCACAGCTGAGCAGGTCAAGGTCAGCATTAGAGTCACAGCTGAGCTCCCTGGTACCACACCTACTCCTTACTACTGGTACGTCCCACCCACTGAGAGCAGAGACCTCCCTGCACTGAGCTTGGCCATCCCAGATAACGGCCTTGTTGCTATCCCTGTTGATGTCCCTGCCGATGCTAAGGATGTTCATGTTACTGCTAATTTTCAAGGAGTAAGCAAGGAACTGACACTTGGAAAAAGTCATTCTCCTAGCAATTCTTACATTCAACTGATATTAAAGTCTGGCTCTGTCATTAAGGCTGGAGACAGTATTTCCTTTGAAGTTAAAGGGACACAAGCTTTGACCAAATTAGTTTACCAAATCCTATCAAGAGGTGGCATTGTCAAAACAGGAACTGTGGATGCTAATGGTCAGCTTGTCTACCAGTTCTCTATACCATCAGACTCTAGCATGGCTCCTAATGCAAGAATTGTTTTGTACTATGTCAGAGCTGATGGGGAGATTGTCACAGACAGCATCAGTTTTGATATTTCTGGAGCTTTTAAAAACAAGGTATCTATTGACCTTGACAAAACCGATGTTGAGCCAGGAGATGATGTCACAGTGACTGTTAAGGCTGACCCAGATTCTACTGCCTACTGCCTGGCCATTGACCAAAGTGTTCTTCTGCTCAAAGGAGGAAATGATGTCACCGATAATGACGTATACACAGAGCTAAAGGAATATGACACAATCACAGAGTCTAGTAGCAACAAAGGAATTATTGACTGCCCAATGTGCAAGAGGAGAAAGAGAATGATCTGGTGGCCATTCCCAACCTACTACGGAGGCAGTGATGCTCAGCAAATCTTCAGTAATGCTGGTGTAGTTGTTCTCACAGACGCGACAGTTTACCATTATCAGGAACCAATTCACTTGTTTAACATTCCAAACTTCTTCCAATGTGGCCGGAGCCTCAGTGGTCAGCTCAGGAAGCGTTGCTTCGCGTCCTTCTCTCCTGTCATGAACCTGGTCTCTAGTGTCGTCTCGGACCCAATCACACTGCCGGTGACAGAAACAGAGACTAAAACAGAAGATCTCCAGCAGCCGACTAAGACCCGGTCCAACTTTGTGGAGACCTGGCTGTGGAATTCTTTGGACATTGGTGCCAATGGCTCAGCTTCTATTACTGCAACAGTACCAGATACTATCACATCCTGGGTGGCCAGTGCTTTTGCCATCAACTCAGCTTCTGGCCTCGGTGTTGCTCCAACTCAGGCTCATCTTCGAGTGTTCAGACCATTCTTTGTTAGCCTCAATTTACCCTACTCAGTAACCCGCGGGGAGCATCTGGCTCTACAAGCTAATGTTTTCAACTACATGACTGAAGATATGCAGGTCCGTGTGACACTTGCCAAGTCTGACAACTTCTTCAACATTGAAATTGATGCTAATGGAGCTGAAGTTTTGAAACAAGTTGAAAGTGTTCAAGATGTGATGATCAAGGCTGGTGAGGCCAAGTCTGTGTACTTCCCTATTGTGCCAGCTGACTTGGGTAAAATTGATATTGAAGTGAAGGCTCAGTCTACCAAAGCTGCTGATGCAGTTAGGAGACAACTCTTGGTGGAGGCTGAAGGAGTCCCCAAAATATACAATGTTCCTGTCCTCATTGATCTGACAGAAGGCAAAACTAGTTTCTCTAAAACTGTTGATCTTACTCTACCTTCCAATACTGTGAAAGGTTCAGAACTGGCCAGAATATCTGCTGTTGGTGACTTGATGGGTCCAACCATCGCTGGATTAGATTCACTTCTTCAGATGCCTACTGGTTGTGGTGAACAAACTATGATTGGTCTAGCCCCTGACGTCTATGTAACTGACTACCTTAAATCTGTGAATCAACTCAGCGGAGACATTCAAACTAAAGCTTTGAGCTATATGGAAAGTGGTTATCAAAGAGAACTGACCTATAAACACACAGATGGCTCTTTCAGTGCTTTTGGTAACAGTGATGCATCAGGCAGTATGTGGTTAACTGCCTTTGTTACCAGAGTGTTCAAGCAAGCAAAGGCCCACATCTACATTGATGATGAGGTCCTAATTAAAGCTATCCAATGGATGGTATCAAAACAAAATGCTAATGGAAGCTTCCCTGAACCTGGAAATGTGATTCACAAGAATATGCAGGGTCAAGCTGGCTCTGGAGTTGGACTGACTTTGTTTGTTTTAATTTCTCTTCTTGAGAATAAAGATCTTTTGGTCAATACAAATGCTGCTGGTGTCCTGGTTGATGAAGCCAGACAGAAAGCTTTAGTTTACTCTGAACAGGAAGTTGCTAAAACCGATGACCTTTACATACTGAATATGGCTGCTTATGCTTTCCAACTTGCCAACAGCAGTCAAGTCCAGACAGTTCTGAATAAACTTGAGCAGAAAGCCACAGTTAAAGATGGACGTAAATACTGGCACCAACCAGAACAACCCAAGACAACCAACACTTGGGATTACCCCAACCCTACTAAAGCCGTTGACATTGAAATGACATCTTATGCTTTGTTGACATATGCTGCCAGAGGAAACATTGTTGCGGGAAAGAGTATCATGCAATGGTTAACAGAACAGAGAAACTCAAACGGTGGCTTCTCCTCCACACAGGACACTGTGCTGGCCCTGAATGCCTTGTCCGAGTTTGCCAAACAGACCTACAGCAACAACTTCAATGTTCAGATCACCACTCAGCTGAATGCTACAACATCTTACACTTTCAACATTGACAAAACGAATTCCCTTCTACTACAGTCTAGAGAAACACCAGATGTTCCTTCTCAAGTAAAGATTGATGCCACAGGATCTGGTATGGCTTTGGTTCAGGTTGCCGTCTCCTTCAATGTTGAATCTGAAATCTTTGAAACAACTTTTGACCTGACTGTTAAATTAATAGAAGAGACAATCAATAATTTATTTGTTGAAACATGTGCCAAGTGGCTAGGCAGTGGACCAAGCAGTGCTATGGCTGTTCAAGAAATTGGCATCCCATCAGGCTTTGAGGCTGACCTTGAGAGTATCCCACAGTTGGACATTCTGAAGCGAATAGAGACACAAAACAAGAAAGTTATCCTTTATTTTGACCAGATTGGAACAACACCTGTTTGCCTAAACTTCCGCGCTGTGAGAACTGGACTAGTTGCTAAATCCCAACCTGCTGCTATCCGAGTCTATGACTATTATGAACCACGTAACCAAGTGACTGCCTTCTATCAGTCTCAGATTCTCAAAGATTCCACGTGTGTGGTCTGCAAAGAAGAATGTGCAACTGTTTAA**ACTCCAGTTGTGTTGCTTCAAGTATTATGCATGAAATAGTTTTGGAGCTGTTAAAAATGTATACATTAGAGAAATCCCAAAGAATGAAGTTTTTACAGTACTAAAAAGTGTGATAAAGACTTAAGGCTAAAATCAAAAACAGAAATGTTATTTCCTTGTAGATTATAGTTTCATGTATTTATTTGTATCAAAATGTATCAGTGAAATCTTCAATAAATGTTGAATGTGATCTGTATAATACAAAA

**>BgTEP3**

ttcagtatatataacacatttgctgtgcagg**atgtggaagttaattcttatagctgttgtcataacaagcggcagagctcagaatattccaggcagacaacaagctgcttcaaactccacgcagcggaaatcaacatggagagattgtacctacatgatcatagcaccttccaaggtgagagccaacatggacctgtcattaagtgtccatatcctcaacgccagtagtaatgtcatgcttttagtgaccctttcacaaggtcagaaaactgtggtcagtgctaacaaagtctttcgacaaggtgctcctgaaatttttaaattaaagttaccagctgaccttcctaacagtatctatactctaaaagtcaagggtagtggagcattaaccttcaatcaatccactgatctttcatacaacagcaaggaagctttttcagtttttattcagattaacaaagctatttttaagccagatgacacagtgaacttcagagtatttggaatctatccagacttaaagtcctattcaggtcctatggatgtttcaatttatgatgccaactctaacaaaatcaaacaatggaataaagtgaatcccgtaaatggtgtcttcacccagaagctggttctatcagcacagcctgtattaggagactggaagattgaagccaaagctaatacaacaagggcgcaaaaaatatttacagttgcaaaatatgttcttcctaaattcaaagtggaaatagtaatgccctcatttgctttgaccagtgacaatgacatcacagtgactatcaagtcaagatacacctatgacaagccagtgaagggaacagctgatgttctggtcaaactaaaccaatttgaaaattccatgccaattgattttgctcagtctctaccagtgacatcactacaaatgtctataaatggtgaagccaaagtgacggttccaaaggatctgattaatgccaaagacaaagatgttttgattgtgattgccaatgtgacagaatctttaacaggcaacaaaatgacagcaaaaaatacagtcactttttatgaccaaggtgcccagttggaatacccaaaaattaaccctacaagttttaagccaggacttaaatactctgcttatcttcaaatcactcagcctgatggcctgccaacaacctccatcactgaaccagtaaggatttcttggaaagttgaaaaagagaaaaaaccaatagacagtatcagagtacgcttacctaaaaatggtctcgtaagcttttctgtgaatgtaccgcttaatgcaagctcattaattatatctgcaacatttcaaggagtaactaaagaacttatagtggaaccaagttactctcctagcagttcttacatgcaactagcattgaagactgcctctgttattagggctggagatgttgtgttttttgaagtgacgagtacaacaccaatgacccagctggtttaccaagtcttatccaaaggtgtcattgtgaaagttggaagtgaaaacgccaccagtaaattttcccaccagttctctgtagtatcagactccagcatggctcccagtgcaagaatggtgatctatttctacagaagagatggggagattgtcatagacagcatcagttttgatgtttctggggcttttaaaaacaaggtctccttcggtttcaactccaagtctgtagagcctggaaataatgtaacagtgactgtcagagctgacccaaactctgctgcatatcttctagctattgaccagagtgtacttctgataaggggagataatgatgtcactagtgatgatgtgttcactgatcttaaaaagtatgacacagctgctgactccgccgactgctccacatgcaagagtggcttgtggccttcatggacccttggtggcgctgttgcccttgagatcttcactaaagctggtgtggtagctctcacagatgcagttatcattcagtccaaaccttcagacgaaaataaagacgggctaattcgaaatccaggggcaataatggtgccaaaacagatacgccaagttttctctgaaacatttctctggtcagatttaaccattggtgtcaatggatcagcttctattactgcaacagtaccagatactatcacatcctgggtggccagtgcctttgctgtcaattcagagtctggtcttggtattgctccttctcagtcttatctccgagtattcagaccattttttgtcaaccttaacctaccatactctgttattcgtggggaacatttggttgtacaagcaaatgtttttaactacatgactgaagatatgcaggtgacagtcacattggccgcgtctgacaagttctacaacattgaaatagatgcaaatggagcaaatggattgtttcaacaaagtcaaagtgtaaaagttatcatggttgaggctggcgaggctatatctgtttatttcccaatattcccacatgaattgggtaaaattgatattgaagtgaaggctcagtctaccaaagctgctgatgcagttaggagacaactcttggtggaggctgaaggcattcctaagacaaaaaattaccctatactaattgatctgacagaaggcagaacaagtttctcacagactcttaatcttccactcccttccaatactgtgaaagattcacaacgcaccagattttctgttgtaggggacttaatgggtccaactattgctggattagatgcccttctacagatgccaactggcagtggtgaacaaaacatggtaaatctagccccaaatatctatgttgttaactatctacagtctgtgaatcagctttctacagacatcaaatcaaaggcttcgaattttatggaaaaaggctatcaaagagaactgatgtatagacacccagatggttcttttagtaactttggtagcaatgatacatctggcagtatttggttaacagcttttgttgttaaaatattccatcaggctcagggccatatctatattgatgataatgtcctaatagaggcccttcaatggatcgtcacacaacagaaccctgatggcagcttccaattaccatcaaagggtcaagcaggctctagcgttgtactcactttgcatgtcttaatttcactgtttgagaatgaggatgttctggcggaagataatattgttgaagcacggggaaaggctttaacttttgttgaaagtgaagttgacaaaactaatgacctttatgttttgagcttggctgcgtataccttccaactagccggcagcacacgtgttcaagcagttctggacaaattggagctaagagcaactgttaaaggtggacgtaaattctggcttctacctgaacaacccaagaaaaaaactattttgaattacccaaatcaaaccaaatccattgacattgaaattacatcctatgttttgttgacatatgctgccagaggaaatcttgttgcaggaaagagtattatgtggtggctagctgaacaaagaaactctcaaggtggatttcccactgcacggtgtagtattattgctttgaatgccttagctgtgtttgctgaaaagacttacagaaataatttcaatatgaaaataacagccaaagtggccccccaaaaaatgttacagtataggattgacaggactaatgctcttatcttacaatctggagaagtatcagatgttccagctcaagttcagattgaagctactggttcaggcttagttttggctgaaattgctgtctcttttaatgttgaatcagaaatattcagaacaacatttgacctcaaagtgactctagtcgaagaatcaatgaactatttcattcttcagacttgtactaagtggattggaagtgagtctgatggtgttatgacagtccaagaaattggtattccaactgggtttgaggctgaccttgatagcataccaaatttggaaaatctgaagcgcattgaatcacagtttaaaaacttatatttgtacatagaccagattgattccacgccagtttgtttaacaatgaaagcagtaagaataggtgttgtctctggactacaaccatctactgttagagttattgattactatgaaccaagtaaccaagtaacagccttctatcagtctcagatccttgaggcctctattatctgtgatgtctgtaaagaatgtgacaactgtttttaa**cctccagttacttcagccagtgttgttcatgatgaaatgacctatataatagagaaatacttccattgtaaaatatcagttactttgtgtgttaaagaagaagagtgcaagc

**>BgTEP4**

GGAAGCAACCATTTACTATTCGCTAGAGTGAATTCACCACAAGGTCCGCTGTTATAAAGCACAAAAAAGTGATTTAAA**ATGAATCAGATTTGGCTAGCTGCAGCATTTTTAGCTGCAGCAATAGTCCACTTCCCAGCTCAATGCCAATTAGTACCTATCCAAGATGAGTCTACTACCCCATTGCCTCTGAAGAATGCTACATACTGGATGACCGTGAGCTCCACTGTGCGTCAGGGTCAGCCTCTGGAATTTCGAGGACAGATATTAGTGGGCAGTGATCCTGTCTCTGTGACAGTCACTCTCCTGAATGGGGAAGGCACTAAAACACTGAAGACCAGTCCAGCAATAACTTTGAGCCCTGGAGCGGTTCAAAGTTTTAAAGTTGATGTTCCTGAAAACATAATGGACCTCAGTGGTGAGGAATATCTTTACCAGATAAAAGTTCAAATGGTTGGTAAGGGTAAGACAGTCAACTTCAAAGAGGAAGTACTCTTAACATATGAATCAAAGTCTTTTTTTACCTTCATTCAGACAGACAAGGCCATGTACAAGCCAGGGCAAACAGTGAAATTTCGTGTTTTATCAATGACTCCTGATTTAAAAGTTATCAGAGACAATTCAAATGATATCATTATTGAGGACTCGAATAAGAATAAAATTAGACAGTGGCAAGGAGTCAAAGACCCCAATGGAAGAGGTGTCATGGAACTTAGTCTGAAAATAGCTAAACAAGTAGTCTTTGGAGATTGGACAATAACAGTGAAAACCAAGGGTACTGAAACGTTAAAAACATTTACAGTTCAAGAATATAAGTTGCCAAAATATGAAGTTATGATTACCACTCCACCATTTGGAATCATCTCTGATCCTGTTTTGCCAATCACAGTAAAGGCCATATACACATTTGGCCAGCCTGTAAGTAAAGGGACAGTTGATGTTGTCATTACATTGGTTTATTCCTTAAAACCAGAAATAAAAATTTCTGGCCTACTTAATAAAGATGGAGAATTTACACTGCAAGTCAGCAGCAAACAACTTTTAGGGCTAGTGAGTAATGGACAAACGGATCTTAATTATCAGTCTTTTAAAATTAATGCCAATGTGACAGAAACAGACACTGGTCGCAATGAGGGGAGCTCAGTAACCATCATCTACTACAAAACTCCACTACAATTGACATTTTTGGGCATCTCACCAAACAATTTTAAACCAGGTTTAGGCTACACAGCTTATTTGGAAGTCAAAAAGAAAGATGACACCTTATTTACCTTAGCAGAAGCCAGTCAAATTCGTTTGCTAATTAATGTGACTTACACAGTTCAATTGAACAAAGAAGAGATGGCCCAACGAGAGAAAGAGCTCAATATTTCAAAGTCTAATATAGATAATACATCAGGCACAGATGAAAAACAGCTCTTAATCCGCCCAGGTTTTATTCCATACTATGATAAAACGAAGACTTTAATACTAACCATTAATGACCCCATAAGAACTGTGCCAGACAATGGATTGATACCTATCAACCTTGATATACCAATGGAAGCAGAATCAGTGAGCATTGAAGTTAACGGTCTGGAGCCATTTGCTGCTGAAAAGGCATACAAGTCTGTCTCAAAGATGAAGTCACCAACTGGTACATATCTTCAGCTTAAAGTTCCCACAGAGACCCCAAAGGTTGGATCAACAATCAAAGTCACAGCTGTTGCAACAGAAGTGATAACAAAACTGAACTTTCAGGTCTACTCAAAAGGACAGTTGCTTTTGTCTGAAATAATTAACAATCCACAAAGCAATACTAAATCAGTGGAATATTCTTTTACCATCTCACAAGCAATGGCCCCGACACTTACTATAATAGCATTCTTTATGAAAGCAGAAAATTCAGAGTTTGTTGTTGACAGCTTGTCAATTGGTGTTGATGGACTTTTTCAAAAACCAATAACTGTTGAATTCAGTAAGACTCAAGTCAAACCTGGAGAGAAAGTTGATGTCACTATAAAAGCCGAAAGTGATTCTATAGTCTATTTGTTAGGTGTTGATAAAAGTGTTCAACTATTGAAATCAGGCAATGACATCACACAGGCAATGGTACAAGAAGAGCTCATGGGTTATGGAACTTCAGGCGATTATGGCATGTGGAGATTCATGTTTTTCTGTGGATGGCCCTCTTATTTTGGAGGCACAGATGCTAAGTCAATATTGTCGAGTGCAGGTGTGCATATAATAACAGATGGTCTAGTTTACAAGTCTGCTTTTGATAACTCATTTGCCACAGATTCTGGAGATTTAGAAATGCAAAAGCAGCCTGAACCTGATGAAGCTAGATTCTCAGGATCAAATCTTGTGCTTACAAACGTGGTGAAAAGAAGGAAATATTTTTCTGAAACTTTTCTATGGGCAATGGAGATAATAAATGCTGACTCTAATGGACAGGTAACTTTATCAGTTACTGCTCCTGACACTATCACAACATGGGTAGTGACTGCATTCAGTGCCCACCCTGTGTATGGATTGTCGATTGTAAAGGAGTCCGCCAATCTTACGACTTTCAGAGACTTGTTTGTTAGCCTGGACCTCCCTATCAGTATCATCAGAAATGAAAATTTCTGTTTTGTGGCTACAGTGTTTTGTTACAACAAAGAGGAAATACCAGTTCTGCTCACATTGGATAAAAGTGATAACTTCAGCAATATTCATGTTAAAGTTGAAAATGGTCAAGTCATCCTTTCCAAAGAGAGTCTTCATTATTCTCACTTCCTTGGATATCTTGCTGAGAGGGACATCTCCTCTGTCAAGTTTTGTTTTATGCCAACTGCTCTAGGAGATATACCACTGAGAGTGAGTGCATTGACCAATGTCCCAGGATTGAGTGATGCTATGGAACAAATCATTACTGTTAAGCCTGAAGGAGCTGCACGCAGTACTAGTAATTCCTACTTAATAGACATGGCAACTGGCAGATGGGAGATGAATGTTACTGTCAAGTTTCCTGCTGCCACTGTCACAGGCTCAGAGACAATAATATTTAACACAGCTGGTAATCTTCTGGGACCAATGTTTGACAATCTTGATGACTTGTTGAAAAAGCCTTATGGCTGTGGTGAACAAAATATGCTAAATTTTGCTCCAAATATATTTTTACTGGAGTTTCTTTTTTCAACAAATAAAAACAGATCAGTTGCCATGGAAAAAGCTAAAGATAATATGCTAATTGGTTATCAAAAAGAAATCACTTATGAACATTCAAACACAGGCGGATTTAGTGCATTTGGTCATCATGAGGGAAGCAAGGACTCTGCAAGCTCATGGTTAACTTCTTTTGTGGTAAAGTGTTTTGCAATAGCCTTCCAATTAGATGCTGCACAAGGTAATGTGATTACCATTGAAAAAGAAATCATCCAACGATCTGTAAGATTTATGATTTCACAACAGAATCTGAATGGATCATTTACTGAGAAGGGAAAAGTTTTCCATAAAGAAATGCAGGGTGGATCAGCCGAGGGTGAGGCTTTAACAGCCTACACTGTAATTGCTTTGTATGAAGCCCAGAAAGTCTTTGCATCAGGTGACTCAATTGTTGCAAATATTAGTCAGAGCATCAAGCTTGGTGTAGACTTCCTAGTGAGGAGGTTGCCATTCCTGACTGATCCTTATGATATCTGTATTGTGACTTACACACTGCATTTGGTGAATGATAACAATAAAGAAACAGCATTTAACAAGATGCAGTCTATTGCAATTACTGGAGATGGTCTGAGGTACTGGAAAAGAGCAACACCAGCTGAGTCCAACATAGCAAAATATGAGTGGACAGCCTCTGCTGACTCTATTAGCATTGAAATGACTTCATATGCCCTTCTAGTGTATGCTTTTAGAGAAATAGCCAATACTGAAGGACTGCCCATTGTAAGATGGATCACTAACCATAGAGGACCTAATGGTGGCTTCATCTCAACTCAGGATACTGTCATTGGTCTACAAGCACTGGCCAGAGTGGCAGCCAAAATATATTCCAATGAAGATATTCCCATCACATTAGCTGTTAGCTATGAAAGTAAAGGACAACTTGTGAAAGAAATAATTAAGATCAACAAGAGCAATGAAATGTTGCTACAGTCAGTTGACATCAATTACAAAGATGAACAACCTAATTTTGTGAATATTGTGGCTACAACAGATAGTGGGAAAACTGGTCCAAGCACAGTCATAGCTGAAATTGTCTTAGGCTACAACATATTAGCAGAAACTAGTGCCAAGTTTTATGACATGTCTCACACACTAGACAAATTGTCAGCTGGCTTTGTTTTAACTATCTTAATCAAAACAACAAAGGATTCAAGCAGCATGTGTATCCTTGAAGTGGACATCCCTCCAGGCTTTACCCCAGACTCTGATGCCTTAAAATTGAATAAAGCTATTAGTCTGTCGGAAATTTTGGGAGATGTGTTGGCCATATACTTTAACACTGATATGATCACTACTAAAGAAACGCCAGTGAAGATTTTTATGGTCTCTACTGGTGGAGTACTGACAAAATCTCAGCCAAGGATGTACAGGGTATATGATTATTATACTCCAGACAGAGAATTGTCAAAGAATTATCTATTGGAAGATACAGACTTCTGTACTGCTGCCCCTGATGTTGGTGGATGCCAATATAGACAAAAATAA**AGAATGAATCTCATCTCACAACTGTCTAAAAGTTTGTTTGGTACATGGAAACAAATATTGACTATGTAGATAATAAACTTTTTATTCTATGCTGTAGAACTTAGTAGAGCATTTTATAAGCAAAGAAAAATTGGTTATACATTGCCGTTTAATGTAATTACATTCCAAAACAAAAAGCTTTATTGTAAAATTTGTAGTTATATTTGATATGTGATATATGAAACTTTTTTGTTCTTGTCGAGCTGTGTAAAGCTTAAGTTATATTAAAAACTAGTTCATTCTGGTTATTTTTATCTCAATCTCTTATAACTGTTATAATCTGAAAAAAATATTGCAAAAATCTTATTTATATGAATTGAAATACATTTCTACAAATATTAATAAA

**>BgCD109**

TATACTGTGATACATAAAGCTCACGTGGCAATGAACTGATGGTACAACTAACTCCCAGCTCTGAGACTCTTTTGATGAGTCTTTGTCTTCTTGAACAAGAGGAATAACTTATACTTCTGACACCATTGTAACAGGTC**ATGTCGTGGACTTCTACTTCCGCTTTGTGTTTGCTTTGTGCTTATTCCACATTATGGATTACATGCTACGGGTCCTTCATGGTACTGACCCCCAAATCTGTGTACCCGGGAATACCTTTAGGTGTGTCAGTGACGGCACATAAAGTGGTGACGGCTCCTGTCTCTGTGGCTCTCTCACTTGAAACTGTACAACATGAGAGGTCAATTGGCAACGCTGAGACTATTCTTTTACCAGGTGAAACAAAGTTACTCACTATCCAGGTTCCACTGTTAAATTATACCTCCCCTTTCCTGCAACTCAAAGTGTCAGCAACAGGAGGTTTCAGAGATTCCCAAACCAAGATGATATCAATTAATCAGAACACTTCCCTCATACTGGTGCAGACAGACAAAGCAATCTACAAGCCTGGACAGAAAGTTCGCATTCGAGTTGTAAATGTTGACCGATATTTAAAACCAGTGTTTAATCCTCTGACTGTCATTATTGAGAATGCTAAAAATGACAAATTAGAAGAATACAAAGATGTTAATTCTAAAAATGGTAATTACACTTATGGCAAAGGTGTCCAAGGTCAGTGTGAACTTACAGTACATTACACAGCATCTAGTCAAGAAATATACCACAAAGAACTCAATTCAGACGGTGTGGCTGTCTTTGATCACTTGGATTGGAAGAAGCTCTCACGCAATGTGGACAATATCACTGTGCAGGCTGCAGTGACAGATGAAACAGGACGTAAGGAGCAGGGGGAGACAACATTGGCTGTCTATGCTGATCCCAAGAGAGTTCGAATTTTAGACACCTCTACTACAATACTCAGACATGGATTACCTGCCCATATTTATATCGAGGTTTCGGATCACAGTGGTAACCCAGTCTCCCCTGTGACCCTGATGATGGATGTTACCCATCCTGAGTTGAAGGGCTTTACAGAAGTGCTGAACGTTCCTGCGGGAGAGACCATAGTAAAGTACACCTTCATTGCGATCAAATCTCAAGAACAAAGTTATTACTACAACAGGGGAGATGGCACTTTGAAGGCCTGGCTGCAGATGAATGATAACGTTTTTGATAGTAAGACATTTACTGTGTATAGAACTAAGAGTCCATTGGCTCTTTCTATCTTACCATTGGAATCTCAGACTATTAGGGTCGGTGAGTCAGCCATAATAAAAGTGAATACGTCCCTTCCCTCATATTTTGACTCTACTTTTGCTTATCTGGTTATGAGCCAAGGAAATATTGTGAGTGCTGGACAGCTGAAAGACAACTCTTTTGTCATCACACCCACGTTAGAGTTTTGCCCCTTGAGTAGGCTACTTGTATACATGATTGCCGGTTCTGAATCGGAGAATGGAGAAGTTGTGCTGGATGCAGTGGATCTCACACTGACTGGCTGTTTCACCAAAGAGGTGAAAGTTGAGTTTGAGGCATCAGAGACAAGAACTGGCACTGAGGTGGAAATGAAGGTGGATGTCTCCCGACTAGATGGCTCCAATGAAATGCCAGGCCAGCATGACGTCTTCTACCTGGCCGTAGATCAGAGCATCGTGCTGTTGCAGGGAAGCACTGACCTAAACACGGATAAGGTTGTGTCTGGCCTGTCCAGTTTTGACCAGGTGGATGAATCAGTGACCTTGTCCTCAGCGGCAGCTTATTTTGAGCGCCACAAATTGTTTTATCTGACTGATGCTAGTGTGTGGAGCAGAAACCAGCTCTTTGAAAAAGAATTAATGATTAATCGTGGCCCAATGTTGAAAAAAACTTCTGCAACAGAATTACTACCTGATTTTGATTCGGAAGTAGACGGGAAAGTTCCAGAAGCAGCATATCAGACGTCAGCAAGAATTCGCAAAGATTTTCCAGACACTTGGCTCTGGGGACAGGCTGTGACAGATGTTAATGGTCATTTAAGAAGCAAGGTAGTGCTGCCAGACACTATCACTTCCTGGATAGTTTCAGCTTTTGCTGTCAACAGTGAAGGCCTAGCAGTAGCCAAGGAACCGTTTAAGTTGACAGCCTTCCAGTTGTTTTTCTTAAGCATGAACTTACCCTATTCCATCAAGAGGGGAGAAGTTTTTGTGCTGAGAGTAACTGTGTTTAATTATAGAAGCCAGCATGTGCAGGCAGTTGTTTCCCTTGCCCATAGTGACCAATTTATGGTGGTTGATGAGACTGAGAGTGAAGGCTGGTACAGCAAGTCCCTGTCATTGGAAGCGTACAGAGCCTCCAGTGTTTCCTACAGAATCAATGCCACGACTCTTGGCCAGATTACTTTGCATGTCACAGCCACTGACCCAGCAGACGGCCAAAAGGATGAAGTCAAGAGAGAGTTGCTTGTTAAGCCTGAGGGAGTTGAAAGGTCAAGGGCCATCACTAAAGTCATGATCTTGAATTCTGGGAAGAGCTTGAGTGAAACTTTTAATATCAAGTGGCCTCAGGAGAAAATTGTTCCAGACTCACAGAGAGTTGAGATCAAAGTAACTGGGGAAGTGTTTGGCCAGGCTCTGTCTGGCCTGGAGAATCTGGTCAGCATCCCCTTTGGGTGTGGTGAACAGAACATGATTTCGACTGTGCCAAATATTTTTGGCCTGAAGTACATTCGAGGCACATCCCAAGATGGAATGGAAGACTTGGCTGCTAAACTGACCAACAATATGAAGCTAGGCTACCAGCGTCAAGTTGAGAATTACAGACATGAAGATGGTTCCTACAGTGCATGGGGAGATAAATTTGGAAATGCAGAATCTGGTAGTACTTGGTTGACAGCATTTGTTATACGAAGCTTTGCTCAAGCCTCTAAATTTATTTCAGTAGACACCAATGTCCTGGAAACTGGTATTGAGTTCCTTAAATCCTGCCAGGACAGAACAGGAAAATTCATAGAGAGAGGACAGGTTTTTCATTCAGATATGCAGTCAGGTACTGGCTCTGGTGATGGGCTGACTGTCTATGTATTGATCAGTATGTTGGAGGCATCACAAGCCTTAGGGGAGACCGGCTCACTCTCTTTTAAAAATCAGATTGATCTAGCTCTGAACTACATTCGAAGAAATCAAGACCCAGAAAAACTTAAACAGGAGAAGCAAATATATTTAGCTGCCATTACTGCCTACAGTCTTTCCCTGGTCAGCAACAAGGATAAGGACATTCTACAACTTATTGAGCAACTACTAATGGTCATCAAAGAACTACAGGTGCCATGGTCCAAAGTGGACTCTCAAGATATTAAAACTCTTCAAAGTAAACAGGCCGCAGGTGACGTGGGCCCCCCATATATAGTCAAAGCTCAAGCCACCAGAGATTTAGAGATTGGGGCCTACGTCCTGCTGACCCTCACACGTATAGAGAATTTAGCTGAAGGTCTGGAACTGATGAAGTGGCTACAGTCTCAACAAAATAGCAAAGGTGGATTCTATAGTACACAGGATACAATCATGGTTTTACAAGCTCTGTCAGAATTCGGCTCCAAATTTAGACCAGGAGAAGTATCCTCTCAGCTTCAAGTGACACATCCTGTTAACTTGGCATTTACATTGTCAGGGTCAAGAGCGTTACTGCTACAGACTGCGACGCTTCCATGGGATACAACAAAAGTCAATGTGACACTGACTGGTGGGACCAACTCTTTGGCTGTAGTCAAAGTGGTGTATACTTACTATACCTTCGCTGGAGATGATGACCAGGTCCCAACAGAGACCTTGCTGTTTTTGGAGACCAAGTCCATAAGACTGGGCAATGGCATGCATAAGGTGGAGGCTTGTGTTAAAAGTTCCAAGTCTCTGAAGTACAAAGGAATGTTTGTGACTACCATGGCACTGCCATCTGGAGAAAAGCCAGCAGATGACCAGTCCACAATACTGGCTAGCAACCCCATGGCATCTAGAGTTGAGGCTGATGAAAAATTTATTCATTTTTATATTGATAAGGCTCCTTCCAATGAAGGTTATTGCTTGACTGCCAATGTGGAGCCCCATTTAGAGTTTGAAGTTCAGAAACCTGGCTTTGCTCAATTTTATACTTACTATGATCCAGACAATGTTGCGGAGGTGCCTCTTTCACTGACATGTCACAATTGTGACACAGACACGGCTGTAATGGTGAACATGGCCAGCGTCCTGCTGACTACTGTTGTTTGTTTGCTGGCCAGCTTGTTGGCCTGCATGTAA**ATAATTTTTTTTTTAATGTTGCTAAGGAGCGTTTTTTTTTTTCCTAAAGGTGGCATCAATCAGTGCAAATACGAATGTTCATAATATGTCATAGCTTTAATAAACTACAGCTTCTTTCCTCTTTAACAAAAAAAGTTTTTATTTTTTATTTTTATATTGAAACACATTTTTATATTGTGTTGGACAATTCTGAGCAGCCAAAATGTGTATTTTTTTAATGAAATGTTAGCAATGACTTAAATTTGTTCAAGTGCTGTGCTATCATGAACTGTGTTATGAATTGCTGTTATTGCAATATTTTTTTGTTATCTATTTCGTGAAGTTTTTATTTGCTTACACTGTGATGTGAATTCTTTGACTTGTTGTTATATAATGTCAAGTATAATTGGATTGTTGTTTTCATTCAGCAAGAGTTCTTGTTCTTGTTGTTGAAAAAAAAAAGTGTTTTGTTTTGTTGAATTTTATATTTTTTATAATGGGCTGAACTTTTTGAAGTTGCACTTTTTATATTCTAGAAATAGTATTTTTTCCATAACCTGATTTGTTTATCTCTTTATAAAAAACATTGCTAGAAACAGGTTGATGAGCTTACAGCTGAATTGTATATAGTCCTAGCAACATGTGAGCAGCCTAGGAAAAAACAATGCAACCTTTGACAGTTTTATGGCAGACACCTATAGAGTAGCTGCACTTTTTACTTTTAACATTTTTATATATTTACAAAAGAAATTCTATTTCTTTCTATGTAAATGTTGCTCAGGACTTGTGTGAATTTTGTTTTTTTCTTTCTTTTGTTAATCATTTTGAATTAGCCTGTTGATTTATTGTCCTCACAGTTGTGGAAATATTGAAAACACTTTTCTTTAACTCCAAACTCTTTTCTGACCACATTTTGTTTTTTATTTATTGAATTATTTATAAAGCAAATTATATTCTTTTAATGAGATCCTTCTATATATATCATTGTGTTGTAGACAGTTTTAGTACCTGATGTAAAAATACAATGTTGCACAGGAAGTAAACACTGACTCCAGATTTTTAATTTACCAGTACCAGAATTGTGAATAACTTGTTAGCATTATCTCAAGACTTGTAGCTTAAGTTTTTACATAGATTTGTAAGGAAAGAAATAAGGGATTAAAATAGTATCAAAACAGCTAAAAAA
