# Supplementary material for: A New Assessment of Thioester-Containing Proteins Diversity of the Freshwater Snail Biomphalaria glabrata
Source: Genes (Basel). 2020 Jan 7;11(1):69. doi: 10.3390/genes11010069 (PMC7016707; doi:10.3390/genes11010069)
Supplement: Supplementary file 1 [file genes-11-00069-s001.zip › Figure S3.docx]

**A) ClustalW alignement of BgC3-like proteins**

**A2M-N**

______________________________________________________

10 20 30 40 50 60 70 80 90 100 110 120 130 140 150 160 170 180 190 200

....|....|....|....|....|....|....|....|....|....|....|....|....|....|....|....|....|....|....|....|....|....|....|....|....|....|....|....|....|....|....|....|....|....|....|....|....|....|....|....|

HsC3 **-----------MGPTSGPSLLLLLLTHLPLALGSPMYSIITPNILRLESEETMVLEAHDAQGDVPVTVTVHDFP--GKKLVLSSEKTVLTPATNHMGNVTFTIPANREFK-----SEKGRNKFVTVQATFGTQVVEKVVLVSLQSGYLFIQTDKTIYTPGSTVLYRIFTVNHKLLPVG-RTVMVNIENPEGIPVKQDSLS**

BbC3 **------------MIRSLVCVCLLF-AAV-LGQQGPAYFISVPNLLKVGTEETVSVNVFNVANPVRVKVYLQDYP--DRKTTFSEAEVDVNQDEPSLVTVRVNPDNLPES----RATKRYVYVVAKSDDPQLTFQKEAQVLLSYQQGYVFVQTDKPIYTPNQKVKMRIMPLDQDMTPAS-QPVKLEILNPQGIIVERKTFP**

CrC3 **------------MESRILLFTLTV-IFLCIRIEAANIFVASPNTLRIGEQETISVLLEGNK-AETVEVYLQDHP--GKTKTFSKTVGRVEPRISTEFKVQVNPEDLPDKDILVATAKHYVSLVAKAGN---WFHKETLLLVNPRSGYVFIQTDKPIYTPKQTVHMRIITLNEDLIPQN-KMITLQIKNPQDITVEHHSWK**

RdC3 **-----------------MLAILLLFGILVSEVQCGYYFVTAPNVLRFDQDETVVVSVFGLN-NAAVKVWLEHENKQFSVKNVLVKDEKHPENIFVRVTENDTVSTLTEEK------TRKVKLCAEWNGQK----QTREIILSYHSGYLIIQTDKPIYTPKEKVKIRTLALDESLKAVDGWQVGMDIVSPSNKTLGRKLIK**

BgC3-1 **MPSIRFTRSTSRFIFCTHLLFLLVFVNMMKNCFSTKYLLTVPKEPTYDTDLTVTITALTKP-EVPENITLEFRGLKDKSKVLNSTQINFSEDETKNWTVVFPWERMLGLD-----ETGVLLKMRTTNEMENVVEKDLSLKFRNTSGYIFIQTDKPIYTPRQTVKFRIIALDEYQRLTK-YPIKVDIKNPQGVILERMRYS**

BgC3-2 **-----------MRDIMQTFWILLL--LLVPVSCSSHHFVLLPSVLRLETEEVFSVTSLEAEGDVTFKIYLTDYP--ERKRNFSETTVTVPQGESVMARVLMTVGDLPQN----AQPPLFVNVIVMTVDHEPHFQKEAVILVNESPGYIFIQTDKPVYTPDQSVFTRVMTLNEHFRPAS-WPLQVDIQNPDGMTISRKVID**

BgC3-3 **-------MYVKRTMTVADGLSLILSVCLIQQSVGTKYFLTVPQVPSYDANVTAVVTAFQHTSQSTEKVLLQYIGGENSKNVLNSTHLSFDQDGSQQWTVTFSSESMQELR-----ESSVVLQMTCNGQKK-----EILLTFRQSSGYIFIQTDKPIYTPGQTVKFRVIAVDEDQRLSK-HHLKVDIINDQQVTVDRMRYS**

_________________________________________________

210 220 230 240 250 260 270 280 290 300 310 320 330 340 350 360 370 380 390 400

....|....|....|....|....|....|....|....|....|....|....|....|....|....|....|....|....|....|....|....|....|....|....|....|....|....|....|....|....|....|....|....|....|....|....|....|....|....|....|....|

HsC3 **SQNQLG---VLPLSWDIPELVNMGQWKIRAYYE---NSPQQVFSTEFEVKEYVLPSFEVIVEPTEK-FYYIYNEKGLEVTITARFLYGKKVEGTAFVIFGIQDGEQR-ISLPESLKRIPIEDGSGEVVLSRKVLLDG------VQNPRAEDLVGKSLYVSATVILHSGSDMVQAERSGIPIVTSPYQIHFTKTPKYFKPG**

BbC3 **GSATG----FIAETFDFPAFPLFGNWTAIAHYG---PEMQLNVSTQFEVKEYVLPTYGVRIIPSN--PYILPQDDVISGEVEALYTYGKGVDGFLDVKFGIIDLEGN-RQLFAQLQ-TEVNGGFGFYEIDT----------QRIKDLDLWFPEGSRLYLEAAVTEEAGGLREMAVLTSVRFETSPFRIGYDLTATHFKPG**

CrC3 **VGKVGRRIEFYTFDYSFPPYPLFGEWSAIVSYG---YDLLYNTTVKFEVKEYVLPTFSVDVTAP---EIILESTKKIIGSAHAKYVYGEPVHGTANFKFGVKLNTGD-IIIIGTLYNKQLQDGKVDYQIDVNEF------MKHGKISGFPDLEGNHLFVEVSVLEQATGQRESGINENGIFTLSPYDVSFKRCLNNFMPG**

RdC3 **GSPSG----FYQNDFTLPPYPEIGLWSARAFYKG---QFETESHALFEVREYVLPTFGVTIDVDV--EYILPQTKHITVTVKAKYVYGKPVQGNARLTLRLKGEDKDTDYIIDLKKKQLENSPDVEGSATEFRLDVKKDILDSSLLRDKPFPNGKRLEVIATVYESATGNEEAASHDGTIFTESPFIFKFTKSKLNFRPG**

BgC3-1 **AED-----AFKSQEFELPKDTPPGIWTISANLEGLGQLYSLAHTVAFEVREYVLPRFSAVFKIDT--DVITMDTTWIRMNVTAKYVYGQPVVGKVEMRLGTWDENSSVTLIPSASYRGELINGVFKRDVKRSS----------LFPTNESFNGVKRLYVQVNVTETATQETITIEDTSTFVSHPYYEVDFTPSKTYFKPG**

BgC3-2 **S-----KDLILKDVMKIPENPVYGNWTVTAKFI---NGLRTTSAVRFEVKEYVLPTISVSFHIPDSRKVILPNETHFHLAVGAKYMYGKPVRGHVTVTYGLLWHGHV-FTVGKQRNLQLNDTGFVECGITVDD--------LRLPVQSVWFPNGGKLHVQAAVTETASGHVEKADDTSVVFADHLYVIRFTRSDRHFKPG**

BgC3-3 **AED-----AFKGQNFELPKDIAPGRWYISANFEGLDSNYRLAHNVSIEVREYVLPRFSATLHANT--SVITKDSKALKLTVTSKYVFGRPVHGNVEIHLGILDNN---KLLPHAVLRGKLQNGQFSQDVDVNI----------LTLSKLMYTSNQRLHVGVNVIEKGTFENYTLTDSSIFISHPYYIVDLKSSKEFFKPG**

**A2M-N2**

____________________________________________________________________________________________

410 420 430 440 450 460 470 480 490 500 510 520 530 540 550 560 570 580 590 600

....|....|....|....|....|....|....|....|....|....|....|....|....|....|....|....|....|....|....|....|....|....|....|....|....|....|....|....|....|....|....|....|....|....|....|....|....|....|....|....|

HsC3 **MPFDLMVFVTNPDGSPAYRVPVAVQGE-----------------DTVQSLTQGDGVAKLSINTHPSQKPLSITVRTKK-QELSEAEQATRTMQALPYSTVGNSNNYLHLSVLRTELRP-GETLNVNFLLRMDRAHEAKIRYYTYLIMNKGRLLKAGRQVREP--GQDLVVLPLSITTDFIPSFRLVAYYTLIGASGQREV**

BbC3 **LPFLVKLTLTYPDTKPAQDIPVRVSATAIIPGQDPIVILGRNNEH-NSDTTNQYGQASFTVDVPPGTQTLTVTAKTEQ-VGLPVAHQAQENFEATPYQSPSGSYLLVRVLQRGP------VPVDEAIDVEAVVTKQNDIQSYNYMVVTRGQVTLQGKIVRQG---GVLKTITFRTSAVMAPISRLIVYYIN----LQGEV**

CrC3 **YTTLIPVDINFVSGKPAPGIPAVITVTDEKGR-------AVVVQK-SEDTSDERGRCNFLVNPSKDLKEIKIEVKTND-GRGAQYQRKGQHRMGEQSSEFGGVIAIDRGTTKKD------LKVNEEFSASVLTNPAGGISLISYMVISRGKILIHKFLPKVE---LIGHKIFFVVNTDMSPSFRLVVYAV-----YKGHL**

RdC3 **YKYYLKVELFYVNGKPAKDTDVEVSMFEDGVLK-----------EEKAGVTNDDGRIIQIQSTAAKAKKISFKVSTSN------NEFESDEFVVTAYPGKNQMQVEYVTTDNNLN-----------MMRAFTNIKGNSYTGMFFVVVTRGKIVFIKYKDAAN---EASEETSDQLEELVSPDARLLVFYVDT---ETDVI**

BgC3-1 **FPYTVHVQVKARSGRLASWVLLYLHPKFYDSEK---HLLRESNNHVIVQPLDMYGKLSMEVQIPLNADRVIFSAIVMD---FAKIKFNEYILNVSKLASDINEYIVISMP--TPIQK---IQRGEVILNYTKPRNLLDK--ITVLVIAKGYVIYTLKNITKNNNGSSTIYLPTSLRGDASPSMRIVAYYWTMG----EII**

BgC3-2 **LPYVLEIDVFKANGETGPYLALSVECQIEMQDGTKETIPATGQYDGHSMVTDARGKLSVHYNIPANAKQLHFKVSPKEKATTDQASKSDYFFIASRFYSPSSVYMQLHARLTDTNRFGYTPAVGDHLTVWTSYTSPEEISTVTLVVISRGSIVWQVSTRNIL---GNSTYFHFKITQDMSPTAWILAFAVRGNE-PGSEV**

BgC3-3 **FSYTLKAVIKTKVPLTVSHLDLYIFAEFLDAND---NIIKSVSENVPIMKNTIV---TQDFITPKTAEKINFKVHVVD---ENHPSFEHFHFTVKKYISANHEYLHINMSKFQPVMK---WSDGVFFLEYTKSAYLNSSSLITVNILSKGQVIYSIN-VKKNILGVSPVSLPKQLFGELSPAYRIVAYYYIAGA-VPELV**

**ANATO**

_______________________________________________________________ **++++** _____________________________________________________________

610 620 630 640 650 660 670 680 690 700 710 720 730 740 750 760 770 780 790 800

....|....|....|....|....|....|....|....|....|....|....|....|....|....|....|....|....|....|....|....|....|....|....|....|....|....|....|....|....|....|....|....|....|....|....|....|....|....|....|....|

HsC3 **VADSVWVDVKDSCVGSLVVKSGQSEDRQPVPGQQMT---LKIEGDHGARVVLVAVDKGVFVLNKKNKLTQSKIWDVVEKADIGCTPGSGKDYAGVFSDAGLTFTSSSGQQTAQRA--ELQCPQPAARRRRSVQLTEKRMDKVGKYPKELRKCCEDGMRENPMRF-------------SCQRRTRFISL-----GEACKKV**

BbC3 **VADSTLLEIENVCRNKVMVSSTEDVEPQEQAN-------IEVNADPNSLVGLLAVDQAVYLLNNYNRLTSQKMFQAMAKYDQGCGPGGGQDSANVFKDAGVTVLTNTQLSPAVRS--NAGCG-GQSRRKRDLQ--NSLEAKVMEFNETLQPCCMDGQQWDPLGR-------------SCLQRAKLNST----SQDECYFA**

CrC3 **LTDSILYNVEPTCKESVKFKLETDVIDRPKPGQLVK--INIVEATKDTKIGLLAVDEAVYILRNKDRLTREKMFQEMEKHDLGCGPGGGSNIQSVLANAGVAILSSTTLTNYKRE--DYICA-VRIRKKR-----EIMEEIVKKYEKRDKQCCILGMKHDPDQR-------------SCEERHAIFEKYDFDGKETCMAA**

RdC3 **VADSVKFEVEKKCRGDGLHLETDAQTVKPGSNG-----KLTVTGTPLMFVGLNIIDKALLLLNDKNVLKKKKMFETLQSHDLGCGEGSGKSGADVFKNSGLTVLTNAKVEEADLERQTDGCK-RNTRKRRDAD---------SCWFGVAETCCTEGYDFAVTLFELEEYPERMNSRRKCLPKALILAE-ENRLSIKCVMA**

BgC3-1 **MDSLFIEAPVKYCVEELYVN--KGGLFSTTPLKPKDKLNIDLRGGSNMRVGLVAVDKAVLLLNDKQTLTRKLLFNELEKHDQGTHKNNGT-FEEILKSNGLQYIFLDTVQVESD------------------------------DPPPIGTVKEDGFDET-------------DSFIAM-QQALP---------------**

BgC3-2 **ISDSVWLEIVPQCDGELHIQRENDGKKVLKPGDIG---TVTLTGQPYMVVGVVAVDSAVYRLKN-STLTRQSVFQQITAHDRGCGFGGGKDAAKVFENSGLMALTNADLPMTPKT--VDGCVDKAVRKKRSPE----------ARKRARDICCVEGARVRNATLA------------LCYFATQELKK--TMNSEFCVRE**

BgC3-3 **ADSLLVDTELDTCVDEVYLIRDKFSQFSPVPKKPKDKLDLLIIGSPLMKIGLLAVDKAIFLLNDKQTLTRELLFHTLGSHDPSTSEGDGLNAELILENSGLYHMMVDTDAYSTS---------VTPRRALSSF---GSFYDISFDRINMPEENRHGN-------------------------------------------**

**A2M**

_____ _______________________________________________________________________________________________

810 820 830 840 850 860 870 880 890 900 910 920 930 940 950 960 970 980 990 1000

....|....|....|....|....|....|....|....|....|....|....|....|....|....|....|....|....|....|....|....|....|....|....|....|....|....|....|....|....|....|....|....|....|....|....|....|....|....|....|....|

HsC3 **FLDCCN-----------YITELRRQHARASHLGLARSNLDEDIIAEENIVSRSEFPESWLWNVEDLKEPPKNGISTKLMNIFLKDSITTWEILAVSMSDKKGICVADPFEVTVMQDFFIDLRLPYSVVRNEQVEIRAVLYN--YRQNQELKVRVELLHNPAFCSLATTKR---------RHQQTVTIPPKSSLSVPYVIV**

BbC3 **FLTCCNH-----ARSLRRLGRGRGRMGGGGGLLDIDIDEDE-----SQLVARTEFPETWIFEDVQVDDRGQ-----AVVPVTVPGSITTWVIQAVGISTANGMCVAKPFRMKSFKKFFIHLQLPYSIIRGEQVAIRATIFN---YDQQDLRVNVYMQGVEGVCSGARAGE--------RSERKTLFIKGNDAASVLFPII**

CrC3 **FLGCCNEKHLYLLKNIEKEGRGRFGFDPADQLITVGLEEEQEL--LKQLNVRKDFRETWIFDDVYVGPKGR-----VEKELSLPHSITTWVVQAVGISNTGGMCIAEPLKITTFKSIFVQLNIPYSVVRNEQVEIQATVFNN--HAHQSVRASVYMYGVKGLCSGAEEGQ--------RTERKVLDIGRSSAKSVSFPVV**

RdC3 **FFKSCVDESEYMITEETKIVSKSLDDESEYLEDIAKLADNGIISKTRSDFRESWLFSVYNLDQQGQ----------MQMQLKVPDSITEWRIQAIGITKDIGMCTADPVDFKAFRDFFIQLDLPYKAARLEHFNVKATIFNYGQPGDAAKTANVYLKGVDNLCYNSDPGK--------PSPRVQVKLPPNSAKTVSFPMI**

BgC3-1 **----------------------------------------------PQT-VRSYFPESWMFEEHVLPKSGF-----LRLSWPLPDSITTWSVLVVGVSANRGVCVSEPVDQIAMKMFFADVHVPYKATRLEEVKVKIAIYN---FYNYTLAVQGTVTSEPGLCISSNSSQTFNSGTSLKTLTFSMNIAAFQTASEIIKVI**

BgC3-2 **FFQCCRSFVKG-TLSLDALGRLRTSIERLPEDIELNFDEDDLSN-MKNIPVRTNFPESWWFEEYNLGAEGR-----ADVDFVLPDSITTWSVEALGMSVEAGLCVAPPLELTTFTSCFVHLDLPYSVVRLEQVEVRATVYN---YMTKKIRVNLILQSVDGVCYSGQPGD--------ATDYVKLEIDPNDAASAYFPIV**

BgC3-3 **------------------------------QVSEKQPETEDSLP--QRSDVRFYFPETWLFEEKIIPRDRK-----LPLELSLPDSITTWSFVAVGLSNNRGICVSIPLEQVVEKPVFLEVRMPFKASRLEELNINIIIHN---YHTNDVRPEVTIIGDSGLCFAENATR----GGNHSDHGFNMTVTAGEMAERTVRII**

**Thioester domain**

__________________________ ____________________________

1010 1020 1030 1040 1050 1060 1070 1080 1090 1100 1110 1120 1130 1140 1150 1160 1170 1180 1190 1200

....|....|....|....|....|....|....|....|....|....|....|....|....|....|....|....|....|....|....|....|....|....|....|....|....|....|....|....|....|....|....|....|....|....|....|....|....|....|....|....|

HsC3 **PLKTGLQEVEVKAAVY--HHFISDGVRKSLKVVPEGIRMNKTVAVRTLDPER-----------------------LGREGVQKEDIPPADLSDQVPDTESETRILLQGTPVAQMTEDAVDAERLKHLIVTPSGCGEQNMIGMTPTVIAVHYLDETEQWEKFGLEKRQGALELIKKGYTQQLA-FRQPSSAFAAFVKR---**

BbC3 **PLEVGTFPIRVVAFS---TAAGGDIIEKSLQVIPEGVERRLVRSIFVDPKGRARDRKR----EGEEEVALPTEHDVDPDNGLQFDVVDVRLPPETIEGSEQCAVSIMGDIMGPTITTTIGGL--GTLLRLPTGCGEQTMIKLAPNVYVLSYLHCTDQITKDVEEK---AYDFIRQGYNKQLS-HRRPEGCFSVWGQNNRY**

CrC3 **PLKVGEFPVRVVVFT---VHG-SDFIEKKLNVVPEGAKDSKLISLQLDPTNQQKRQKR----SIHEKYYID---SIDPEKKMQISIVELQPPPNYVPDTAKCLVSVIADRFGPVVETALENT--EKLIQHPRGCGEQTMLFMAPTLYTVKYLKVTGQLNANTEKN---GYKFIRDGYSRELT-FRKNDGSYAAWQNR---**

RdC3 **PLKDGLFPVTVSAIVTDLGIPEVDVIEKRLYVVNEGIEEKLTIVVCLDPLKQKEDCVN----------DKRVVSDIQASNDERHYEIDLTLPENSISQTGAATAYIRSNIITDIVNTIIEGV--DSLFLKPAGCGEQTMIRLAPTVYALSYLKQTKQMTVDIEKK---GNQWVRDGVSREISGYRHGDGAYAAWKHR---**

BgC3-1 **PLKVGELGLLVHVKS---QKD-EDIVKKTLHVVSEGLRVFKTITFVLDPEAKHATFQG-------RTHFSTIRNHIDKIKKQQFTTIDLALPKDVIKGTEFCGISAFGDLMGDIITHGIVRS--KSFVDQPLVNAEEVIGDLGPAVFALQYVNDTKLLTDELKNK---GQRFLLQGITRLLN-YRK-ENAFSLHTDS---**

BgC3-2 **PLEIGTFPIIVKAFS---TWG-RDAVEKTLRVEGEGLEKIHTISVMLDPSGKRFLRSR----SSNHTFNMKN--EVRSAEKKQNVELDLDLPQEVIPDTESCSVHAMGDLLGPTLQVMIEGV--TELLRLPTGCGEQNLIYLAPNVFVTRYLRATRRLTSFIEKK---ALALIRQGVSKQMF-FRKVDGSFATWPHA---**

BgC3-3 **PLKIGELTLKVSMIS---HLG-NDTVEKKLRVIAEGLRVRKAITFVLDPGAKHTTFMNYSDNNIQQSNTATIQNRYIASRNMQHTTIDLALPPEVIKGTESCQISAFGDLMGDIITHAVVQS--KGLMEEPTLIAQEVLNDLGPIVHALNYINDSGLMTHDLKLR---SHRFIRHGVVRLLT-YKS-GKAFSVRPGM---**

**A2M-COMP**

________________________________________________________________________________________________________________________________________________________________________________________________________

1210 1220 1230 1240 1250 1260 1270 1280 1290 1300 1310 1320 1330 1340 1350 1360 1370 1380 1390 1400

....|....|....|....|....|....|....|....|....|....|....|....|....|....|....|....|....|....|....|....|....|....|....|....|....|....|....|....|....|....|....|....|....|....|....|....|....|....|....|....|

HsC3 **APSTWLTAYVVKVFSLAVN---LIAIDSQVLCGAVKWLILEKQKPDGVFQEDAPVIHQEMIGGLRNNNEKDMALTAFVLISLQEAKDICEEQVNSLPGSITKAGDFLEANYMNLQR-SYTVAIAGYALAQMGRLKGPLLNKFLTTAKDKNRWEDPGKQ---------------------LYNVEATSYALLALLQLKDFD**

BbC3 **PCSTWLTAFVNKVFCQAKK--FVTSIDEEAVCKATEWLLS-TQREDGAFKEVYKVHHREMTGGV----QGDASMTAFVLISLLENCECPIAERSIAI---ERATLFLERQLEQLKR-PYVIAIVTYALHLADSPLKGAANEKLRSIAKYDEGTNSRYWEADASSIADGQQPYWYTRKPSAIAVETTAYALLTQMHIGDIQ**

CrC3 **PSSTWLTAFVMRVFCQAQK--LIE-IDEKVICSGMQWLVQ-RQKPDGSFVDEKPVIHQEMIGGV----KGALPMTAFVLMALHECSCTTVPGLKLAK---IRAAAYLEIKVPHIQD-PYIMSLVAYSLSLAGNNAKVEANRKLLSMATFQADKNYHYWGDPNS----------------PRAIETAGYGLLVQILNNDIE**

RdC3 **TASTWLTAFVAKVFCQAKKVVDDAVDKEEDIKITIDWLND-HVNADGSFIDAMPVIHREMIGQIN---EGDPTLTAFVLITLQECSIRSDKLNATIR---KAMKYLESLPKRNLKNNPYLLAISTYALALSDSTKKNDFRKLLLGIQKKDREGSYWGNPGGKP--------------ASAHSVETTAYGLLAMLEFNDFR**

BgC3-1 **SPATWLTASVVKILCHIEKANLTFIDKENLIDNAINWIMR-QRREDGSLKEADPRLSQESL-------QYKIMLAADVMISLLECNKDEEQEAAELI---MGLVTFIENNIDNINN-SLALAKAAYAMKLFEIDSESTAQIITKLKEFMKRDKLSRLYWSDSVIDNPPRQPIWYHQGATAAAIEATSYALLVFLDHS-MI**

BgC3-2 **ESSTWLTAFAMKTLCQAEH--YVT-VDHNQTCSSFHWIAK-QQKPDGSFREEVWVTHREMLGGV----NGDVSHAAFILIALLECDCP-GNDHKDVT---AQALRYIETTVAQTDR-PLALAISAFALTLAGSPSSDGVVKRLQSMAKSSPEGYTYWSHGTEEDYEGHEKPYWYTKQPGALAVEVTSYALLTNLARGDIS**

BgC3-3 **KPATWLSALILKSLCHAT--SLAFIDKHNLIDTGFSWLQD-QIKKDGSLNELDWTGRKNNA-------QYRIELAAEVLISVLECNRKEKEDHLTLQ---DKMADYLQKHIDKIKL-PVVMAKTAYALMLYNSDSNKTLNAVDKLKRLALKGVQGHIYWANKPKDEDEKKPHWYIDRVPESSIEATAYGLLVFLRKKNLL**

____________________________________

1410 1420 1430 1440 1450 1460 1470 1480 1490 1500 1510 1520 1530 1540 1550 1560 1570 1580 1590 1600

....|....|....|....|....|....|....|....|....|....|....|....|....|....|....|....|....|....|....|....|....|....|....|....|....|....|....|....|....|....|....|....|....|....|....|....|....|....|....|....|

HsC3 **FVPPVVRWLNEQRYYGGGYGSTQATFMVFQALAQYQKDAPDHQ------ELNLDVSLQLPSRSSKITHRIHWESASLLRS---EETKENEGFTVTAEGKGQGTLSVVTMYHAKAKDQLTCNKFDLKVTIKPAPETEK---------------------------------------------------------------**

BbC3 **YSNPIVVWLTQQRNSAGGFVSTQDTVVALQALSSYCGSTKVDP-----TQFTCQMTSDNDLDYNEEIHVDKDNALINQEK---TAP-VGGKLFLSTSGTGIGQMQVEVRYHTPDVHRERC-LFEVVVTTEEAEGPVE------PDEEPEEGQE-----GDEDYPDYDGELDARER----------------------SAF**

CrC3 **YANSIVNWLNSKRTLSGAFKSTQDTVIALYALSEYSILAKKPE-----TDLQCNVTLNNDPSFFKELHFKESTANILQQF---QISNLGGHLIFNTTGYGMGQLAVELKYNVPVPPEKLC-KFDIDVKVNEVKEEIQ------QVIDPRMPGNDVFDLLPDALLRNLGFPKRKER----------------------SLS**

RdC3 **RSSSIVSWLISQRDGQGSFRTTQDTVVGLQALSQYSIATYSAD-------VDLKVTLSADNWITEEIRVTEENAILQKVIPKLPVERGDNRLLVQVQGTGSAVMAIDLRYNRPATEEETC-PFSVTDIDVQDVEDAPIDKRLADARNCDICGK-CEGGDVNQYDDYDIDNFG----------------------------**

BgC3-1 **KEEAIADWLVAQRNPSGAFIGAMDSTVAIQALTKYSQKRYVLSGNAVFLRGNITSDITRSRNHLHSFKFTEENATSPASV---KNVPVGQVLEVFTEGQGLGQMHVNVEYNIPIEKNLQC-HYNVTVEIKPIQYMPS---SVNTSPLCQYCNIGCP---------ANLRSRKEISDVSPIIRIG----------------**

BgC3-2 **TSTGIVGWLLSQRNSQGAFISTQDTVVGLQALSEYSIKSYSAI-----LDMTCHIRSEVDDHFRKSISLTPEDAMVVKTV---PKVPTGGKLHFEAEGTGVGMMQVEVRFNVPE-DRNNC-HFDVTVATHQHNTLLQSFFWDNRKSKCEPCSTDCEEQTSEEEEEEDYEDFTFPPVQPRIQTLWKKKVKG--LNLTVHED**

BgC3-3 **NVDAVADWLVAQRKHNGAFNGAKDSTAAIQALTEYSLQKHKEE--EIKVNMNLTVRAGKAEKNQYKFKFTQENATQPESR---SNVPVYQFLEVLTEGQGLGQMQINVEYNIPVDKNEDC-SFNISVEVKTAKIALD---SSN--LLCSSCDFNCPGAKINYNIDDTIKDRTAISKTVSMLTSGRNPKTNAKPKTKKNKP**

**A2M-Rc**

_______________________________________________________________________________________________________________

1610 1620 1630 1640 1650 1660 1670 1680 1690 1700 1710 1720 1730 1740 1750 1760 1770 1780 1790 1800

....|....|....|....|....|....|....|....|....|....|....|....|....|....|....|....|....|....|....|....|....|....|....|....|....|....|....|....|....|....|....|....|....|....|....|....|....|....|....|....|

HsC3 **------------------------RPQDAKNTMILEICTRYRGDQD----ATMSILDISMMTGFAPDTDDLKQLANGVDRYISKYELDKAFSDRNTLIIYLDKVSHSEDDCLAFKVHQYFNVELI--QPGAVKVYAYYNLEESCTRFYH-PEKEDGKLNKL-CRDE------LCRCAEENCFIQKSDDKVTLEERLD---**

BbC3 **SSRSRIGGLLSSGRSRS-RIARQAEDDQSQHFYLVRVCTSYRGQRG---ASNMAIMDIGMFSGFEPVKQDLEQLLNRAGKKAVQRYETTNRA----VLLYFDEIT-SEEICVSIRMKRVMLVGAV--QPVPVSVYDYYQPDEACTTFYH-PGQGSPLLATL-CDGS------QCVCAEGKCPKETSPKKLRELTQRD---**

CrC3 **DNIQDYARVKRDNRGRVGDGGRRGNDGKSKLLLEIQICVKYLSHVD----SNMAIIEAGIFTGFKVLIDELKQLVKEK-NSKIARFEASDKS----VVFYMDSAPHDKPYCFKFRTVRQFIVGNI--QSSVVKVYDYYKPNESCSQFYS-PDNQSPLIRTI-CEGS------VCQCAEGGCPPRHPFQGVTQIHDISESR**

RdC3 **------NQPEIDPKMESNFGRKKRAANRRVRATATKKCIRFSVSSKNKKKYGMSIVKFGLETGVEVIKSDLDKLIAENNTNIARYEMPSDGKG--FIVFYLHEITSSETN-FIFRLKDGFDGEKNSRQPSSVYVYDYYNPDRHCTQFYG-TGLNRGKSVSYQCGKD----KKECMCFQSMCVQPVEEEIKKLAKKKKKKA**

BgC3-1 **------RARSNLSK-----SRAVRSSSHSKKVYCFHVCLRFIRTEG----NVPINIKMDMLSGFKPVASDLELIK-SGPNVLHVEFQAGTETL----VIQLSKVDTESPSCFGFRVVDDEEVERK--VPAALIIQQVGHPVPSCTLEYHLPDSLES-LKVF-CADFSHINRGECRCYSGLCSKCRPTHANELDLDKT---**

BgC3-2 **ENRSDFEDPNMVSKIGRPRRKRRSVRPYSASVICVEVCVRFLGNKT----TGMSVVDVGLFTGYLPVDEDLENLKLKG---KIDHYEKSQRSV----VLYVDEFTNRDRKCLKLRARQEHVAENL--QPAKVQVFDYYNPDSRCTVFYK-NNNNSGQLANF-CDNQK----QICQCLESRCAACEESWYGLGWMD-----**

BgC3-3 **KPTKQPRRRPQLSRKPKPHGKRSKRALSSGKSYCVTVCIRHLQGVS-----RPVDVRIQMLTGIRPLDEDVRKISQTIPNVIDARLTENAEFL--IVKFSKVEA-TK-NTCFAYRARVENDATRI--NGANIEIIQENSPKPSCVLEYH-PPEDKESLKVY-CADYNHINRGECKCFSGQCGKCGPMTSSEFDLDKT---**

**C345C**

_______________________________________________________________________________________________________________________________________________

1810 1820 1830 1840 1850 1860 1870 1880 1890 1900 1910 1920 1930 1940 1950 1960 1970 1980 1990

....|....|....|....|....|....|....|....|....|....|....|....|....|....|....|....|....|....|....|....|....|....|....|....|....|....|....|....|....|....|....|....|....|....|....|....|....|....|...

HsC3 **-----KACEP-GVDYVYKTRLVKVQLSNDFDEYIMAIEQTIK--SGSDEVQVGQQRTFISPIKCREALKL--EEKKHYLMWGLSSDFWGE----KPNLSYIIGKDTWVEH--WPEEDECQDEENQKQCQDLGAFTESMVVFGCPN------------------------**

BbC3 **--LEEKACN--DLDYAFRVIVMEVRQEGSFDRISMIVESPIK--KGLDDVFDGEERVFWKRKTCSGLQLV---EGTTYLLMGKDGTKYTDEQ-GFDSFRYVITEQSFVAE--WPTGAKAERKKFKRVADKFQQLADKIFLQGCTT------------------------**

CrC3 **KLLLDRACV--DHDYVWKGTVESKRKENGFRYISFRVTSVFKEGIEQKQNILHTSKDLMVRDSCSVADLD---IQQEYVIMGRDGAQFKDEDTGILLYRYILDQSTSIFK--WTRISVAENKQLT-KAFRWLEKHMVMGEGGCPQ------------------------**

RdC3 **LKLYKYACNTDKANYAVVVTIDEVSYNPAVQEKNARGVVVTSINKGIEDLNEEDEIAFFWKFSCPYPELE---EGKTYLIIGRDGNKFRLG--EEEKYRYPLMGTALVIENITPQQARQNRKKYKLLHTVLTNFKRLMLGNGCQN------------------------**

BgC3-1 **---KKLVC-KKEIAYQLRL-GDVQDKIHWMEIDARVLS-LNK--TGSHKLEAGDTIKMMSPSSCSCLVNNY-RDEDFYMLS-KDVERLVDR-RGETVYRYLLDENAEFLHVEKPGVPS--TSSLVPPVPFPYLQQALSKDNVCRA------------------------**

BgC3-2 **--MMKFACSNASYVLEIKALDRDLEKAGFERILGQIQAVHSQ--RGRHELKVGDKVILLKRASCFCPRVSP--DQTYFMML-SQPKRFKDS-DGNQIYAFLMDKKVLVIQNFKPRGLS---REQKEISKNVNRTVKRLKRRGCSGGGKTPKVNGKNRKRRARRGKNGKTKKT**

BgC3-3 **---IKLTC-KADLVYQLKL-GSQEDKIHWLEINATVHSVNKT--TGTHELKEGDEIIMMSPGYCMCFRDYFGKEEKFYLLS-SDVDRLMDR-QGTIVHRYVLDENTTLLRVSQPLSMAGHNNSKSEQTKEIFISQPLNKHEQDLSPHFKLAVSHEHLAAGLSQGDKCEL------------------------**

**B) ClustalW alignment of BgA2M protein**

**A2M-N**

________________________________________________________________

10 20 30 40 50 60 70 80 90 100 110 120 130 140 150 160 170 180 190 200

....|....|....|....|....|....|....|....|....|....|....|....|....|....|....|....|....|....|....|....|....|....|....|....|....|....|....|....|....|....|....|....|....|....|....|....|....|....|....|....|

HsA2M **MGKNKLLHPSLVLLLLVLLPTDASVSGKPQYMVLVPSLLHTETTEKGCVLLSYLN---------ETVTVSASLESVRGNRSLFTDLEAENDVLHCVAFAVPKSSSNEEVMFLTVQVKGPTQEFKKRTTVMVKNEDSLVFVQTDKSIYKPGQTVKFRVVSMDENFHPL-NELIPLVYIQDPKGNRIAQWQSFQLEGGLKQF**

HcA2M **------MRVRTVTRFTSLVLLLWMSCVKSEYLFTMPRSLRGGSQGKYCLTLRNLDHENPAGYNCQVSLHFLTSESIITSEHIYAFNSTESDWSQCVNFDAPFKEDRYTATVTVHID--GKKLFMHDEKVDVWTAKNITLIQTDKPQYKPGQTVKFRILRMDYRLLPL-TDLFELITIDNPGGVRVMQWKNVDVSKGLVSL**

LA2M **-MEEIKWQKMSTLLFLLLLFTHDVY-SKSGFILTAPKSLTPGKSNILNLHLFDIKTNG----FLRIGVKDQDDGNVVAETEVSFNKDNPSSS-IQLTIPSGVEVKRPKLYANGSYSSPSSNDFFFEKDINMHKDKLIVFVQTDKPLYKPGQTVKVRILPTTPDLKLVPKETIGSFQIENPDGIVLGYWPMLSFAEGIAQF**

HaA2M **-MDNLT--RFLVLCCTLCSLTIAVRGSKGNFILTAPRAIDAGSIVYFTLTVFDIPQGG----TVTLRLTHLISNVLIAESRVSVHN-NYNTW-VEMNVPPMSSDISATLHIIGSFS--SDYHIEASQNIHIRHNTILTFIQTDKPLYKPGQTIRFRVLPMDNQLKPLDANTMGDIWIEDPSGIRVAQWNHEQFTEGIKQF**

BgA2M **------MDVTSRSLTLFFITLASLCHAENNFLLTLPKAIYAGSKTEFCLTAYN---------DIKVTIDFISLRNVQDTPVLIKDSYSRGEQ-KCTNFQAPPQGEYRLEVTTQSTEPGAVSELHNSTKVTVHGSKLITFIQTDKPMYKPGQKVMFRVFTLMRNLKPR-TENIKSIYVLDPNDVRVKQFLDVEQ-KGIGSF**

_______________________________________________________

210 220 230 240 250 260 270 280 290 300 310 320 330 340 350 360 370 380 390 400

....|....|....|....|....|....|....|....|....|....|....|....|....|....|....|....|....|....|....|....|....|....|....|....|....|....|....|....|....|....|....|....|....|....|....|....|....|....|....|....|

HsA2M **SFPLSSEPFQGSYKVVVQKKSGG-----RTEHPFTVEEFVLPKFEVQVTVPKIITILEEEMNVSVCGLYTYGKPVPGHVTVSICRKYSDASDCHGEDSQAFCEKFSGQLNSHGCFYQQVKTKVFQLKRKEYEMKLHTEAQIQEEGTVVELTGRQSS-EITRTITKLSFVK---VDSHFRQGIPFFGQVRLVDGKGVPIPN**

HcA2M **EMKLSDDPVLGTWNIKALGADD-------TVQSFKVEEYVLPKFEVKIMPPKYLLPTTTSISGKVCADYTYGQPVKGSLTMKVCFGPEYYP-SYYPEQPCVDVIET-NFDGCHSFSISPSELKLGVQGYPTWGSLKISATVRETATGIELNGTSTGPPLTNDPLKIEISD--ESDGYFKPGFPYKGKVTVTLPDGNPASN**

LA2M **ELALPDEPTYGMWRIKGNIEDT------EIYENFEVKEYVLPKFEVKITPPSYLLTNADSITWKICAQYTYGQPVEGTFVAETNVVKYN----WEKEGVPVIHKEG-LIDGCLDVTVNSSALGFNEQRLSYR-AVNMFAEVTEKGTGIKMN-ATDSIYRTSNPLNIMYLEPTSGKGYLKPGLPFYGKLKVEKPDGTPAPG**

HaA2M **ELPLSGEPPLGTWNIHAFINQV------TTSQTFIVKKYVLPKFDVSIKPPAVIMADAQTIPIEVCAKYTYGKYVEGSLKAKVTYKKLWTFMYRDQRTIPSVEHQA-ELPGCHTFQVNTDDLLMQTEEFGGK-ELEIFAEVTENVTGIVRN-ATTSFEISHQKVFLEFLR---DNDYYKPKMPYVGQLEAKNPDGTPALS**

BgA2M **EFQLIEEAKLGPWKIEVYLDDEDEVRQQATVQEFEVKEYVLPRFEVLITAPNNILITDKNIKGKVCAQYTYGKPVSGFVHIELKTSTNLYR-YSPSDS---QEQVK-QISGCYEFSFDIP-----VEKHYTLYSYKLNVTVTEKGTGVVVNNVFDGPKITYEPLTIEIED--FTKGFFKPGLPYYGKVTVKKIDGSPAEG**

_____________________________________________________________________________________________________

410 420 430 440 450 460 470 480 490 500 510 520 530 540 550 560 570 580 590 600

....|....|....|....|....|....|....|....|....|....|....|....|....|....|....|....|....|....|....|....|....|....|....|....|....|....|....|....|....|....|....|....|....|....|....|....|....|....|....|....|

HsA2M **KVIFIRGNEAN--------------YYSNATTDEHGLVQFSINTTNVMGTSLTVRVNYKDRSPCYGYQWVSEEH--------EEAHHTAYLVFSPSKSFVHLEPMSHELPCGHTQTVQAHYILNGGTLLGLKKLSFYYLIMAKGGIVRTG--------------------------------------------------**

HcA2M **EVIRIKAEKYNL----------QYYWSREFTTDSSGIINFSLSN----LGRDVYSLSLMATAVKYEKQAEIGYRPYSLRVFTPNGYRTITQWFSPSLSSIYIPTIKDSVPCDSTVDLDVIYSTDGSSTN-----VFNFVVKSGDDVIHMVRKKVVFDGVDIPMNVLQFNPAEELEYHEKEVTKAPRPPRPPFVTKGSPLP**

LA2M **EQIELCRFADRERWNRKRWLEEKIRACKEFTSDEAGIIKFTVPP----QTPDITSFRFKAKALQYGKKDGDNK------LNQPQHSFTVSSWYSPSGSHLQLEPITEEIECGKPLTVKFKYTTGEEKKQ-----KFYYQIMARNFIVDTG--------------------SFEHEFLLSE--------------------**

HaA2M **EKIQICVTLEG-------------KQCLIFTSDKNGLIGFAIKPSPVSRDIRVEATTINYEDVHYSSTFWTRK------LRKPTATMILSPWYSPSWSYLQIQPTTEEFECDKAQKVTVQYTAERGSTI-----KFYHQVLSKGRIVQQG--------------------SHQRTFYS----------------------**

BgA2M **EKIIVSTQN-------------EFLYAGEFITDSNGTFLFSLCEG---LTNNRSSVQISAEALGYNSSRYITR-----------GFKTIQQWYSPSRSYVQIPPAEAQLKCSGKVSLTVPFTTKENSLV-----QFYYQVIARGNLVKSG----------------HIIHSGESTYDDTAQSQS------------KCLR**

**A2M-N2**

______________________________________________________________________________________________________________________________________________________________

610 620 630 640 650 660 670 680 690 700 710 720 730 740 750 760 770 780 790 800

....|....|....|....|....|....|....|....|....|....|....|....|....|....|....|....|....|....|....|....|....|....|....|....|....|....|....|....|....|....|....|....|....|....|....|....|....|....|....|....|

HsA2M **-------------------THGLLVKQED-----------------------------------------MKGHFSISIPVKSDIAPVARLLIYAVLPTGDVIGDSAKYDVENCLANKVDLSFSPSQSLPASHAHLRVTAAPQSVCALRAVDQSVLLMKPDAELSASSVYNLLPEKDLTG---------FPGPLNDQDNE**

HcA2M **KVPEPEPVPVVEEEIIPKVSTPLGGGSNQGAEVQEELVVPEVVKSLGVEKAIEGGKNPKRRSSDEPGMSYGIGYFKMPVPIKTSGS-EVTVLVYYIRQDKEVVATSLKIPVESCFKNKVKMEFASAKVRPGEETMFRLQASPNSLCSVGMVDKSVNLMGGDHQLTPARVLDEVKKVQTG---GRYHSYWFDDEKYCMKKN**

LA2M **-----DKSGLTDETYLPIDVTALSLNPPNEPEWENNVIV-----------------------------PPHIGETSLTLIPSFEMNPSAKILVFYVREDGETVADSTKITVKKCLRNKVGLKFGEEKVLPGASSTLQLTASPYSICGIGAVDKSVHILSSDNRITEEEVFNKLGGHDYY-----WPKQATSDYKYCEDYK**

HaA2M **---------KVEEIQYDFEEQIVKKGSSSGNSSE-----------------------------------TEIGEFILAFDTRATMSPISRLLIFYVRDDKEVVADSRKFRIKKCLQNKVSLHFRHEQQYPNTEATMLLSASPSSLCGIHMVDKSIRLLEDDTTFNTDKLFKIMESYDTG-----KDPTEFPG-ICIEDSK**

BgA2M **QLSEEERNETLGSIHYYRPSRGYYQGSHDDTFLKEVVTS--------------------------DFQADKVSFFVLDLDVVPVMSPQFNVLVYHILPDGEVVADGRDFSVQPCFENQVEMTFSKTTVAPGEKVDVFIGAQPASICGLGVVDKSINLLGGNHQVTPEMVFKKIEEFNLVPPPGADEYFNNKDYQYCMKNV**

Bait Region **A2M**

----------------------------------------------------------------- ________________________________________________________________________________

810 820 830 840 850 860 870 880 890 900 910 920 930 940 950 960 970 980 990 1000

....|....|....|....|....|....|....|....|....|....|....|....|....|....|....|....|....|....|....|....|....|....|....|....|....|....|....|....|....|....|....|....|....|....|....|....|....|....|....|....|

HsA2M **DCINRHNVYINGITYTPVSSTNEKDMYSFLEDMGLKAFTNSKIRKPKMCPQLQQYEMHGPEGLRVGFYESDVMGRGHARLVHVEEPHT-----------------------ETVRKYFPETWIWDLVVVNSAGVAEVGVTVPDTITEWKAGAFCLSEDAGLGISSTASLRAFQPFFVELTMPYSVIRGEAFTLKATVLNY**

HcA2M **KNETVSKGMYHHHHHFTSGRPETKDSIEAFRSSKMLVVTDVLLET-RPCSE-PSPYYPIAYSSVVAETMGLPGLAGERSLNFRAKSHPKGNEMERE----SAED---TQVQTVVRSYFPETWLWDLHTIGDDGVVNVTTEIPHTVTEWVGNTLCSNSKDGVGISPMIGITVFQPFFLSFTLPYSAIREENLPVLVTVFNY**

LA2M **FKQTEGEHEGSFSSGFTSTN--YLDSITAFDEAGLVVISDMELET-RPCKP---SGFEDGGRPCPQYDVAFAAPQAANRIGGGGEAGGFGGGIRKKTNKP----------VVEIRTYFPETWLWELQNIGATGELSLKRDIPHTITEWVGSAICISEETGLGVSEAATVKGFQPFFVSFTLPYSVIRGEKVPIIVTVFNY**

HaA2M **EDKPRMPRNMIFQDTFPRSGRPYVDARQAFEEAGMTVITDLKLKS-YHCTY---YELPIP-LMLPDSRYEDTEPQFITRVEALPSNS------------------------EEIRSFFPETWLWELHSVDSTGETAIKRQLPHTITEWVGGAVCVHPKTGLGIWDISSVTTFQPFFIDFHLPYSVIRGESFPLVVTVFNY**

BgA2M **KSTSEGQ---DHEDYFWILSSPFVDALQAFEASGFTVVTDLKLET-RPCSRRPQVFYAGKRQKKLQSNIKCPKGDLCSMFSKLAAAHNILIYLNAK----SLVRGTMEEWEKAVREIFPETWLWDISVVGDSGAVTLHETAPDTITSWIGNVLCVHPETGFGASPVTSLRTFQPFFLSLQLPYAAVRGEKLPIMLTVYNY**

**Thioester domain**

___________ ______________________________ _____

1010 1020 1030 1040 1050 1060 1070 1080 1090 1100 1110 1120 1130 1140 1150 1160 1170 1180 1190 1200

....|....|....|....|....|....|....|....|....|....|....|....|....|....|....|....|....|....|....|....|....|....|....|....|....|....|....|....|....|....|....|....|....|....|....|....|....|....|....|....|

HsA2M **LPKCIRVSVQLEASPAFLAVPVEKEQAPHCICANGRQTVSWAVTPKSLGNVNFTVSAEALESQELCGTEVPSVPEHGRKDTVIKPLLVEPEGLEKETTFNSLLCPSG---GEVSEELSLKLPPNVVEESARASVSVLGDILGSAMQNTQNLLQMPYGCGEQNMVLFAPNIYVLDYLNETQQLTPEIKSKAIGYLNTGYQR**

HcA2M **MTECLTMEVRMKETKDFRIQSVSGAILKMCVCGGDSKSAKFHIVPLTVGEIDLEATAVSIEDDATCVNQIISKEGVGVQDGVRRKLLVEPEGIPQEYTTSFYLCPEG-RLLSQDIDLPVASADKLVPDSQRAKVNVIGDIMGPTLSNLKDLLKMPYGCGEQNMASWSPNIYVLQYLTNTNQLTDAIQDEAKGYMRVGYQR**

LA2M **LSECLPIKLSLEQSDKFEMQN-DTNSYTSCVCGGKSDTTRWMIKPRSLGQVNLTVYGASLP-NEAICGNQDY-STVTARDAATRQLLVEPEGFPKEDTWSTFACPKD-QNGKFTATSDLLLPEDLVEDSARGYVSITGDLMGPAIKNLDHLVRLPTGCGEQNMVKFVPNIFVLDYLTATGSITDSIKEKALNNMRKGYAR**

HaA2M **LSECLPIKLSLEPSDDYTLLT-ELRFQKTCVCGGQSSSVSFPVRPATLGMVNFTVYGYSIEQDDEACGNEIT-ARLSARDAITKEILVEAEGFPKEDVFNYFICPEN-TNGSFATEIPLLLPDDVIMDSARAYMTITGDVMGPSIKGLKKLVSLPFGCGEQNMVLFVPNIFVLDYLTSTEKLTDDIKEECLHNMKTGYQR**

BgA2M **LEKCLHIKMALDMEKNFAVDKNELLKEPVCVCGGKSHTVKIYVTPKGLGYLPIIAKAEIIPGLCSNTIDVDT-QYIGLSDAVKRQMFVKAEGIEQVNTNTMFVCSKVDSPKQEELVLSVPSDEEIVKDSIRGELKVIGDIMGPALTNLDRLVKLPTGCGEQNMVGFVPNIFALKYLTETRRITDEIKSKALKFMEVGYQR**

**A2M-COMP**

________________________________________________________________________________________________________________________________________________________________________________________________________

1210 1220 1230 1240 1250 1260 1270 1280 1290 1300 1310 1320 1330 1340 1350 1360 1370 1380 1390 1400

....|....|....|....|....|....|....|....|....|....|....|....|....|....|....|....|....|....|....|....|....|....|....|....|....|....|....|....|....|....|....|....|....|....|....|....|....|....|....|....|

HsA2M **QLNYKHYDGSYSTFGERYGRNQ-GNTWLTAFVLKTFAQARAYIFIDEAHITQALIWLSQRQKDNGCFRSSGSLLNNAIKGGVEDE-------VTLSAYITIALLEIPLTVTHP-VVRNALFCLESAWKTAQEGDHGSHVYTKALLAYAFALAGN-QDKRKEVLKSLNEEAVKKD-NSVHWERPQ-KPKAPVGHFYEPQAP**

HcA2M **QLKYRHHDGSYSAWGDNEYQNSTGSTWLTAFVVKSMAQSRPFIDIDSKDLHLSMQWLLKHQNGDGCIQSVGKVFSSYLKGGLADG----ENVGGLTAFALIALLEAGIDKNDP-AIVNGFTCLSK----QQTNAD---TYTLTVMAYAYTLYNVDSPKRGQIMAELEARTRVPNPGQKHWIREEEEKKEKDSNYFYWRAP**

LA2M **QQNYRHPDGSYSAFGNRDKQ---GNLFLTAFVYRSFAQAERFILINKNKLNETENWILNRQRSNGCFRKIGKLFNSALKGGISSN---DETPAPLTAYVLISLLEAGYKNET--VIDQGISCLEA-----LSNPS---TYSLALFAYATSLAG--HPSAKDYLAKLEERAITEG-GKTFWK--------SPSSGRYYWGN**

HaA2M **ELQYKHSDGSYSAFGASDKE---GSLWLTAFVLRSFGQARRFMNVDENDLSATRSWILKKQFENGCFIPSGTVLNKEMKGGLSSS---EQSLAPLTAYVLISLLESDMEKHDTLVVKNALKCLES-----EKQPN---IYVLSLFAYASALAKE-NETYGRYLDELDKRAITKD-YMKYWE--------PSSNS-----K**

BgA2M **ELTFRHIDGSYSAFGDKDPQ---GSIWLTAFVVKSYAQAQPYIYIDEKDLQVSLKYLHLNQLETGCYRETGRVLGSYMMGGLKGDNKEEESFTALTAYVVIALLTAGVNSSQP-GIYGAMECINADFDSLREQMD---PYALALVAYANALYAPSSHRTSEIIAALEAVARTEG-DFKYWARKDFQPKVSNS-WYTYSMP**

________________________________________________________________ _______________________

1410 1420 1430 1440 1450 1460 1470 1480 1490 1500 1510 1520 1530 1540 1550 1560 1570 1580 1590 1600

....|....|....|....|....|....|....|....|....|....|....|....|....|....|....|....|....|....|....|....|....|....|....|....|....|....|....|....|....|....|....|....|....|....|....|....|....|....|....|....|

HsA2M **SAEVEMTSYVLLAYLTAQPAPTSEDLTSATNIVKWITKQQNAQGGFSSTQDTVVALHALSKYGAATFTRTGKAAQVTIQS-SGTFSSKFQVDNNNRLLLQQVSLPELPGEYSMKVTGEGCVYLQTSLKYNILPEKEEFPFALGVQTLPQTCDEPK---AHTSFQISLSVSYTGSRSASNMAIVDVKMVSGFIPLKPT---**

HcA2M **SAEVEMTAYVLMAYIAGGQE---GAVSTAQPVVQWLTKQRNAQGGFSSTQDTVVALQALSMYATLVYQGG---LDISVRVDTPSKSYQTGINDSNSLVLTTWDLSPQTTKLNVQVQGKGCTMVQANMKYNIYKDEEKETGQASFEVKVSVYRSRTNIDNCKRRTLRICARYALP-NFSNMAIVEVKMITGWIPVKS---T**

LA2M **SIGVEIAGYAVLTLLQHGGA---SNLAKVTPIIRWLAKQQNYRGGFYSTQDTVIALQAMSKFATIIYKDE---LDLEVGVESSGFEKKIMLTKDNSILMQTFRLQTVPSPVDFEATGSGCGLVQTSLRYNVNTPPPRKGFHLEVTVKRGLYR------DCINAHIATCVKYDGKGGVSNMAVLEMKMVSGWIPDEES-IK**

HaA2M **SVAVEIASYYMLARFEMEEA---KALKSVLPVVRWITHQRNSYGGFISTQDTVVALQALAKYASYISKNP---VDIALAVETDDMTQGFKLDESNKLVTQQLKIVDLPTTVDIDAYGDGCAVVQFSLRYNVEKVSNTGGLELNVNARRRGSN------ECNLPSLGICMRYAVHKEKTNMAVLSVKLPSGYVADEWS-LL**

BgA2M **SAEVEMTAYVLLTYIKLFGP---RAVERTHNIAMWLSKQRSPYGGFSSTQDTVVGLNALSEYSRLAFNGGKTELKVSITGSKLKQTFSLSQKKKTTLLLHRASIPVLPNQISLISEGEGCALVQFSVFYNKLSKEFKDKSSFHLEVNPSHYKPNKDKCDHRSIVISAGTKGKAR-ETSGMVLIELKLVTGWTPLPESLTK**

**A2M-Rc**

______________________________________________________________________

1610 1620 1630 1640 1650 1660 1670 1680 1690 1700 1710 1720 1730 1740 1750 1760 1770 1780 1790 1800

....|....|....|....|....|....|....|....|....|....|....|....|....|....|....|....|....|....|....|....|....|....|....|....|....|....|....|....|....|....|....|....|....|....|....|....|....|....|....|....|

HsA2M **VKMLERSNHVSRTEVSSNHVLIYLDKVSNQTLSLFFTVLQD--VPVRDLKPAIVKVYDYYETDEFAIAEYNAPCSKDLGNA-----------------------------------------------------------------------------------------------------------------------**

HcA2M **VKELLAAKQIQKYEINPDNVDFYFDEFDSQERCFAFEVEQT-DIVVTDPKPALIKVYDYYETKDSVMILYDIKTTCGTKEELPFPKP-----------------------------------------------------------------------------------------------------------------**

LA2M **NIVDREELNLRRYEVDGNQLNLYFSELTDQNLCFNFWLEQD--IEVQETKPATIRLYDYYELEQEVVTSYSIDENCEK--LPPLP-------------------------------------------------------------------------------------------------------------------**

HaA2M **LLENDKEVQLMRHEIEENVVNLYFEEITNDARCFEFHVKSE--FEVENVMPSIIRLYDYYQPDRQVTKDYSIPSTCNSTFLPDLTRFPLFKSSEPLHSDFDEFQEFSDTLNGELPEIITPPETTDYQTESRNVSGSDWNSEETPDHLNDSLYPLEVLETSNSIDQREGNISQISTFVDVDHDLDFPDGLEGNMPVSVLPP**

BgA2M **IQLRFVDIKKIEYNENEGLIAFYFDQLSGKPIEFTLDVKQDLELGVSNPKPADVKVYYYYEKDVFKVQSYKIKTTCGTKEEIPHKNT-------------------------------~~~~~~~~~~~~~~~~~~~~~~~~~~~~~~~~~~~~~~~~~~~~~~~~~~~~~~~~~~~~~DPEFGPEGPNQVRINPGIDAP**

1810 1820 1830 1840 1850 1860 1870 1880 1890 1900 1910 1920 1930 1940 1950 1960 1970 1980 1990

....|....|....|....|....|....|....|....|....|....|....|....|....|....|....|....|....|....|....|....|....|....|....|....|....|....|....|....|....|....|....|....|....|....|....|....|....|....|....|.

HsA2M **--------------------------------------------------------------------------------------------------------------------------------------------**

HcA2M **--------------------------------------------------------------------------------------------------------------------------------------------**

LA2M **--------------------------------------------------------------------------------------------------------------------------------------------**

HaA2M **PDFVQPDCPVCSDSFPSNFSA~~VYCNSAFALKVMKRENNMKTVKIIQDVSFYIDSPKAIKKFGELEYEEECTCTELAEDGKILFIVGSPLSLWNSNGKKHRIHLTSSVHVLLVPPKQIYSFITEAKSSCANDP------**

BgA2M **FTMSSDGCPVCIPVSVLPLNFKDLICRSSAVYKVAIMKG~~KTVKLLQDLRPPSLVKKINIVV~ELELPPGCTCGLLTNQGKKALLLVKKPITADSTLVKLDNTSVITLEDKKFTKTTRNTQKTCPLKKLEEKKKKHEKS**

**C) ClustalW alignment of BgMCR proteins**

10 20 30 40 50 60 70 80 90 100 110 120 130 140 150 160 170 180 190 200

....|....|....|....|....|....|....|....|....|....|....|....|....|....|....|....|....|....|....|....|....|....|....|....|....|....|....|....|....|....|....|....|....|....|....|....|....|....|....|....|

DmMCR **-----MMWHLLRALLVVAAVLDALQPAVGQNDNYYNPNQNQQNPQQPLLPNQ------------------------QWGNNPQTNQYSNNNQNFGQTNPSDRP------------PYRTDSGSYNDIAGQDDYNKRVGGGYQDNEEPSLTRGKSSYNIKATFLESLHSREPTYFIVASRMVRPGLIYQVSVSILQAQY--**

CfCD109-2 **------MWIWILLLFLASGNGQVSQESPE--------YTSRSPP---------------------------------------RNRYDENTGFR-----------------------DQNTG-FRD--------QNTGFGFWNPQN-----------------ENVVIKEATYFVVASRMVRPNQVYRIAVNILHSPL--**

TcTEP4 **MKFKFVSWSWIFLVVACATAQDININNPNQQPDLNNPYSTSSTPNYDLNNNPFTQEPNYNGDQFGNRDQFGQNQPYDPNNFPQRGQFDPNNPQRGQFDPNNPQRGQFDPNNPQGGQYNPDTG-LYEPGGRGPPWRQGGFGYGQGRNEIDA-------------AGSAIKEATYFLVASKTVRPGQLYRVAVTILQEDE--**

BgMCR1 **------MWTSLAALFDEMRRKIEVQSRNMWHLICSTILIAMVSAQQPGGGPP-----------------------------REDNCLIDASV-----------------------------------------------------------------------GCSYARAPQYLVITPKKIRPNQVFQIFATILKMEYNQ**

BgMCR2 **------MFFVILILYFVSLAIGQLG---------------------------------------------------------HEHCLIENSS-----------------------------------------------------------------------GCELGRPPIYMITAPRRIRAGQMFQVFATILRMEYHE**

**A2M-N**

_____________________________________________________________________________________________

210 220 230 240 250 260 270 280 290 300 310 320 330 340 350 360 370 380 390 400

....|....|....|....|....|....|....|....|....|....|....|....|....|....|....|....|....|....|....|....|....|....|....|....|....|....|....|....|....|....|....|....|....|....|....|....|....|....|....|....|

DmMCR **-PITVHASIACDGVQISGDSKDVKEGIPETLLMRIPPTSVTGSYKLRVEGFYQNVFGGLAFLNETRLDFSQRSMTIFVQTDKPLYMQGETVRFRTIPITTELKGFDNPVDVYMLDPNRHILKRWLSRQSNLGS-VSLEYKLSDQPTFGEWTIRVIAQGQQEESHFTVEEYYQTRFEVNVTMPAYFFTTDP-FIYGRVMAN**

CfCD109-2 **-PMTVRSSIQRNGVEIAADYQEVKEGIPETLMMRMPPTSVGGDYKLRVEGMYNDLKGGQAFLNETKLIFSQRSMTIFIQLDKPVYMQGETIRFRTIPIDTELKAYNNPVDVYMLDPNRRIMRRWLSRQSNLGT-VSLSYQLSDQPVFGEWIVQIIAQNQVEEKTFLVEEYYQTRFEVNVTMPAFFFDNDP-YIYGIVQAN**

TcTEP4 **-PLTVRASITRNGVEMTEDHKRVKVGVPETLLMRVPPTSVPGEYKLRVEGLYDDILGGIYFVNETNLIFSQRSMTIFIQLDKPVYKQGEKVRFRTIPINTELKAFDQAIDVYMLDPNGHIMKRWLSKQSNLGT-VSLDYELSDQPVFGEWRVRVIAQNQIEESTFLVEEYYQTRFEVNVTMPAFFLNTDE-YLHGIVMAN**

BgMCR1 **EFVHVIVSIIKDNIEYANTALRFDRPSSRIMQLQMPSNAQEGKYRLRVEGRLNEQDTGNIWQNETDIDFTTKQASLFLQMSRPLYRQGQKVHFRIIPILPNMMPKYGSMVIYVDDPTGIPVRRWQSIQTNAGGIISQSFTLSDQPNFGTWIIRVEAFGHVYRHPFTVEDFWEPRFDVNVSVPSYVMETPEISVAGVMLAN**

BgMCR2 **NAISVRVSIVESDKEYTSSILKFERPSSRLMQLQMPSNAEAGNYKLRVEGRLDELVSGNIFFNETEIEFTPKHASIFIQMSKPIYTQEQLVHFRIIPLQPDLMPKYGNLIIYIEDPSGVPVKRWPGLQTNAGGIISQSFQLSDQPNYGTWHILVDAFGFKYRRPFIVEEFWEPRFDVNVSVPAYVMDVSSLSIQGVVLIN**

410 420 430 440 450 460 470 480 490 500 510 520 530 540 550 560 570 580 590 600

....|....|....|....|....|....|....|....|....|....|....|....|....|....|....|....|....|....|....|....|....|....|....|....|....|....|....|....|....|....|....|....|....|....|....|....|....|....|....|....|

DmMCR **FTSGLPVRGNLTIKATIRPIGYFSNQVLNEKYRLGRSPLEQTNLYNERWRYNNPNQNPQVQYNVPGQLPQDGADLSQDILYRNQYVVERHYQFDEEWPFWVRKPEYQDSSYEAWSGTYRKTLPYLRYFNGTFDFKWPLRELELLVP-------NLANSEVLITATVGEKFYDEIIS-GYSVARVYNSSLRVVFLGDSPQV**

CfCD109-2 **YTSGAPVRGNLTLKASF---------KPLDRTRISSG----------------------------------------------IEPVEMYFNFNEYYPAWFRA-----------LTSDEERIPVLRFFNGSYHFQYPMRELFNFVQ-------SIDGMEATIIATVGERFLDEVIV-GYSVARIFNSTTKIRFLGGSPQV**

TcTEP4 **YTSGAPVRGNLTLKAIVRPI------KPIDRYRLPHRNRNRN---RNRDRYDNRGYNRFEDRNRYGPQRYDDFEYEYDEFERDKPIVEKYFNFDEQMPFWFK------------ITNYYEPVPSLKYFYGVYEFKYPIRELLHYVT-------SLEGMEVVVVATVGERFLDEVIE-GYSTARIFNSSIKLSFLGGSPQV**

BgMCR1 **HTSGRPCIGNASITAFFRPR-----EEIWNRTKGWEKP-------------------------------------YWDAQAGGTPNSGVPMELPQYKPVHTIP-------VKDYYMYFAYEYRFIDYFQGRIDFEWTLEQLMTIAKRGGE-SGSLVDSEFVFFANVSDWYSGLNRT-GWAGTIFFDSKIKLKWVGDQIRT**

BgMCR2 **HTSGRSCVGNGSITSFFIPS-----EEIWNATKGWEKP-------------------------------------YHDAMS--RRLSGTPLDVPKYRSIDSIP-------VSDYSVYFAYEYRFIDYYKGRINFEWHRDDLLGLAKRGGATSDELFGNEFVFVANMTDWYSGLNRT-GWAGTIVYDDELNLKWIGGNVRT**

**A2M-N2**

________________________________________________________________________________

610 620 630 640 650 660 670 680 690 700 710 720 730 740 750 760 770 780 790 800

....|....|....|....|....|....|....|....|....|....|....|....|....|....|....|....|....|....|....|....|....|....|....|....|....|....|....|....|....|....|....|....|....|....|....|....|....|....|....|....|

DmMCR **FKPAMPFTTYLAVEYHDGSPIDPNLLRQGLMEVSGFVESRNGGRRDWPAQRLPMSQQSDGIWEVKIDIRNDLNLDDRPQARDFLNGVQNMRLQANFVDPRGERIQTELLLVSHYSPRNQHIKVTTSTEKPVVGEYIIFHIRTNFYLEEFNYLIMSKGVILVNDRETITEGIK--TIAVVLSSEMAPVATIVVWKINQQGQ**

CfCD109-2 **FKPAMPFSLNLVASFHDNSPLRPTQLNGALMEIRADVEMRSG-RRNIDTQYLRALPEHADVWSVKMDLRKQLGLEQNVDPNQILNDVTSMRIYAHFTDGEGHEARTELLLLAHESPNMRHIKISTSTEKPKVGEYMVFHVQTNFHIDAFNYIIMSKGTILLTGEDNMQHSVK--TFAIPLSPEMAPVATAVVYYVGRYGE**

TcTEP4 **YKPGMPVAAYIAASFHDGSSLPLERLTNGVMEVFTYVESGSG-RRDLPSRQLFMVDGSPGVWEYKFDIKSELGLEG-TKAFEDPTQRGSLRIQARFRDGLGHVAETELLMLSHYSPNNQHIKVFTSTLKPQVGANMIFHIKSNFFIRKFNYMIVAKGIVLVSSDQDMFDYIS--TMAVTLSAEMAPVATIIVWHLGQYGE**

BgMCR1 **FKPSQVMRVQVAVTKYDGTPV--ENVGTVTLTDVTTDSSGTAVQSKS-NTQVPKNGIADFEYQLSATTQTLKLTATYADARKPYSGDNKIYLDPRFN--LGTTVPITMYATRYYSPSNSYITILTSTDRPQVNEYMVFHVKTSNYVPRIYYQIVAQSNIIIGDWLEM--TSRQKTFAVALSRDMVPTARLVVYYIRQPEE**

BgMCR2 **FKPGSLFRVQVAVSYYDGRPV--SGGSVTLVPTVGSQSVKTAMSEYSPQTNPVVNGIAHFQIPLNVYVTRLALTASYHDPGE--TLAMKTSVNPNFV--TSQSRSIQMLCTKYYSPTNSYLSIYTSTFEPQINEYMIFHVTLSHFVPRIYYQVVAQSNIIIADELEM--STKQKTFSVALSREMVPTARVIVYYIKEPEE**

**LDLa**

______________________________________________________ ____________________________________________

810 820 830 840 850 860 870 880 890 900 910 920 930 940 950 960 970 980 990 1000

....|....|....|....|....|....|....|....|....|....|....|....|....|....|....|....|....|....|....|....|....|....|....|....|....|....|....|....|....|....|....|....|....|....|....|....|....|....|....|....|

DmMCR **VVADSLTFPVNGISRNNFTVYINNRKARTGEKVEVAIFGEPGSYVGLSGIDSAFYTMQAGNELTYAKIITKMSNFDEQTNGTYKHIWYSHEGNPDELVYFPASSFGVDANRTFEYSGLIVFTDGYVPRR--QDTCNRTLGFGECLSG---RCYRLEKQCDGLFD-C-DDGTDEINCHARNDTELLNYRKYRFNRVLRHYE**

CfCD109-2 **VVADSLTFPVNGISRNNFTVFINNKKARTGERVEVAIYGEPGAYVALSGIDRSFYTMQAGNELTYANVITKMAHFDEDTNGTYLHTWQYHDGDPDEIVYFPSSTFGIDVNRTFDYVGLVVFTDAVIHRR--HELCNQTQGYGECLSG---RCYKLEKKCDGVFD-C-EDGTDETRCVERNATDLAQFRKWRFNRIQRHYE**

TcTEP4 **VTVDSLTFPVNGISRNKFKVYINNKKARTGHKVEVAIYGEPGSYVGLSGIDRAFYTMQAGNELTYAKVLTKMASFDENINGTHEHIWFSHDGNPDDLVYFPSSTYGIDANRTFEYAGLVVFSDFELPRR--WSRCNATLGWAECLNG---ACYRFDKRCDYFQD-C-TDGTDEAGCKYDNGTELAMFRKYRFNRIQRQYE**

BgMCR1 **IVVDVLNFFVNGTRQNLVTLNINRGKDFSRDTIEFNAYADPGSYVSFSGMLLDLYSRGLSDGITENKLIDELMTYDSTQNGSYRHLWRVSDTEYEY-VFYHGPDYGIDANTSFDTAGLLVLTDARVSRLYNDKFCTDQK-SFPCFVGIESQCFKPEQRCDGTID-CVNDGADEWGCTFQESKEIHNPAMDRVSRVMRFYD**

BgMCR2 **IVSDVLSFFVNGTRQNQVSLYINRGKDFSRNTVEFNAYADPGSYVAFSAMLLDLYSRGMNDGITENKLIDELLSYDQPANSSFKHLWRVSDTEYQY-TFFHGSDYGIDGNTTFKSAGIIIITDADVTRLPNQESCNPLDGKFPCFSGVETECFTSEQRCNGLFDGCPNDGADEWGCIFKDMEIDLKSPLQRISRVMRYYD**

**A2M**

_____________________________________________________________________________________________

1010 1020 1030 1040 1050 1060 1070 1080 1090 1100 1110 1120 1130 1140 1150 1160 1170 1180 1190 1200

....|....|....|....|....|....|....|....|....|....|....|....|....|....|....|....|....|....|....|....|....|....|....|....|....|....|....|....|....|....|....|....|....|....|....|....|....|....|....|....|

DmMCR **NV-WLWKDVNIGPHGRYIFNVEVPDRPAYWMVSAFSVSPSKGFGMMNKALEYVGVQPFFINVEMPEACRQGEQVGIRVTVFN--YMITPIEAIVVLHDSPDYKFVHVEEDGIVRSYNPRTSFGEHQFFIYLEAQGTTVVYVPVVP---QRLGNVDVTLHVATLLGTDTITRTLHVESDGLPQYRHQSVLLD-LSNRAYVL**

CfCD109-2 **NV-WLWKDINIGPHGRQIFNLDVPRRPVHWMITAFSMSPSMGFGMLPKALEYMGVLPFFINVEMPTRSRQGEQIGIRVSVFN--YMRHNIEATVVLADSREFKFVHVEKNGIVQSYKPRTSFGEHQFFIWIPAQDASVVYLPIVP---VRLGNIKIQIDASTLIGRDSVTRNLYVEADGVPQHRHESMLLD-LSNRAYVL**

TcTEP4 **NV-WVWKDVNIGPHGRYIFNVPVPARPVHWMVSAFSMSPSLGFGMLNKAIEYIGVLPFFINVEMPTMCMQGEQVGIRVSVFN--YMMDAMEATVVLVGSRDYKFVHVEENGVVRAYNPRTSFGEHQFFIYIKPQDAAVVYIPIVP---TRLGDIDVTVYASTLIGKDQVTRKLHVESDGLPQHRHQSMLLD-LSNRAYAF**

BgMCR1 **NSSWAWQEIFVKPDGRVDFRVDVPKYPLSWVINGVSVSRELGLGIMLKPVRYDAARYMYMQVEHPKHIIRGEQVGVRVTVFNYWYDDDYLEVLITMHGGDGYSFVTVGEYGYVTSYTPPTHKGDHQTIVFLEPGESKDIYMPIVPDGGVVRGQIEFKVSASCFMQKDEYIGTMYVKPDGVMNYYHTPYLIDLIRFGSIQI**

BgMCR2 **NSSWAWQEIFIKPDGRTDFRVDVPKYPLSWVINGISISQELGFGIMQQPLKFDASRFMYMQVEYPKYIVWGEQIGVRVTVFNNWYDDDYMEILVTMHEGQDIEFVSVGEMGYVTSYSPTTHKGDHQTIVFLEPGDSQDIYLPIVPAKSFRKDKLTFRVTAVSFMGKDEHIGEMIVKPNGVLNYYHTPYLIDLIRYPSIDL**

_________________________________________________________________________________________________________

1210 1220 1230 1240 1250 1260 1270 1280 1290 1300 1310 1320 1330 1340 1350 1360 1370 1380 1390 1400

....|....|....|....|....|....|....|....|....|....|....|....|....|....|....|....|....|....|....|....|....|....|....|....|....|....|....|....|....|....|....|....|....|....|....|....|....|....|....|....|

DmMCR **EYMHVNVTQTPEIPYQVDRYFVYGSNKARISVVGDVVGPIFPTMPVNASSLLSLPMESGEQNAFSFAANLYTIMYMRLINQRNKTLEKNAFYHMNIGYQRQLS-FMRPDGSFSLFRSDWNNSDSSVWLTSYCLRVFQEASFYE-WEN--FIWIDATIIEKNMRWLLQHQTPQGSFFEVT---WLPDRKMNRTN-------**

CfCD109-2 **QYMHVNITETPIIPYDENRYYVFGSNKATISLVGDVVGPIFPTMPVNATSLMNLPMDCAEQNMFSFAANMYNTLYMRLINQRNRTQEKESFYYMNIGYQRQLS-FMNLDGSFSFFRTDWNQSMPSVWLTAFCARIFQEASFYE-WEN--YLYIDPEVIAQSVSWILKHQTENGSFYEVT---WLPDRKMNSSLNYRNDLM**

TcTEP4 **QYMHVNVTETPIIPYEYDRYYVYGSNRARISVVGDVVGPIFPTMPVNATSILHLPMDSAEQNMFSFAANMYTTLYMRYTQQRNRTLEKLAFYYMNIGYQRQLS-FMQPDGSFSLFRSDWNQSDSSVWLTAYCARIFQEASFYE-WEN--YIYIDPAVIAKSVEWVLRHQNQDGAFYETT---WLPDRKYNSSLNFNNDPI**

BgMCR1 **PQFDVPVPEQFRKLEVRENLYIPQSPEAVVSLFGDVVTPGFFQDYLNAENILWRPYGGGEMIVFNFAYNLYSLKFMKYSQQLDDAQLSKSLQEMNIAFQRILSYMNATDGSFKMFRDD---PKPSLWLSAFVVKIVKEATFGE-WERDLFMPRELINKVVLYICS-RQNETTG-AFEPDDIEATYDRKMTLLESLKGDKL**

BgMCR2 **PQFKVNVPELFRKQEYRPNLYVPQSPKATINIFGDVVTPGFFESYLNAENLLYRPYGGGEMVTFNFAYNVLALVFMRNSNQLDAYQTKTVLNELNIALQRIYSYMNE-DGSFKMFRDD---DNSNLWLTAFVGKTLAVAGEEDYWELELYIAKEWAAKIVNFICS-RQNTTTG-AFEPLENEIAFDRKMASLRKMKSDVM**

**A2M-COMP**

___________________________________________________________________________________________________________________________________________________________________________________

1410 1420 1430 1440 1450 1460 1470 1480 1490 1500 1510 1520 1530 1540 1550 1560 1570 1580 1590 1600

....|....|....|....|....|....|....|....|....|....|....|....|....|....|....|....|....|....|....|....|....|....|....|....|....|....|....|....|....|....|....|....|....|....|....|....|....|....|....|....|

DmMCR **FDKNITLTSHVLITLATVKDISGTLGSRVALATQRALAYIERNMDFLRHQAQPFDVAITAYALQLCNSPIAEEVFAILRRQARTIGDFMYWGNQEIPQPPRKLENQKWFSLPRLPYEYDSLNIETTAYALLVYVARR---EFFVDPIVRWLNSQRLNDGGWASTQDTSAALKALVEYTVRSRLREVSSLTVEIEASSQGG**

CfCD109-2 **AHRNISLTAHVLITLESVKDLTGGLGSQVALSAAKAVKWLERNLNLLEQSGKPYEVAIVAYALLVAKASTAEQAFNILARHARREGGLTYWGREQVPLPPTKTENQRSFLLPRLPYKYDSENIETTAYALLVHVARQ---EIMIEPIVMWLNAQRLTDGGWASTQDTVWAIKALMEYTVRSRIRDVSSLTVSVEATALPG**

TcTEP4 **RHRNITLTAHVLIMLESVKDLTSGLSSKVAIAQRNAVHWLERNMDLIKERGRPFDVAIVAYALMKSKAAFAEAAYLELSRHRREEGGLLYWGRQSVPQPPYKIENQKPFSLPRLPYEYDSENIEATAYALMVYVARQ---EIFMNDIVRWLNTQRLTDGGWASTSDTANALKALIEYTSAQRIRDISSLSVTVEATALPG**

BgMCR1 **HAHPIPLTAYVLIALSDLQNYVSEEAAACRDTAVRNAANYLFNIVPEIK--EVFHMAITTYALSLTQ---KRSPFETLWKMKRNDSDFLYFSEETSYENPYDFLNNVRYLKPRQELMNDAHAVQATAYALMAHMNSNLGTKVEREMMMAWLNTMRNSIGGFAATQDTILAMEALLKFTQVDPHRNVFDLSTTVESTSSPS**

BgMCR2 **ITQTVPLTAYILIALEKMSKFV--EGTTCLNTAKRNAVKYLQSQVNSLSKDEIFYMAITAYALSLTSN--AFDIVNELWKLKRNDSDFTYFADQLVYENPSAIQNNVRYLMPRQELLNDAYAVQTTAYALLAHITANKADKLERDMTMTWLNTMRNSFGGFSSTQDTIVAIEALMEYTRQDQKRNESDMSIDLQSMASPG**

**A2M-Rc**

___________________________________________________________________________________________

1610 1620 1630 1640 1650 1660 1670 1680 1690 1700 1710 1720 1730 1740 1750 1760 1770 1780 1790 1800

....|....|....|....|....|....|....|....|....|....|....|....|....|....|....|....|....|....|....|....|....|....|....|....|....|....|....|....|....|....|....|....|....|....|....|....|....|....|....|....|

DmMCR **KTQTLYIDDTNLAKLQSIEIPD--AWGTIKVQAKGAGYAILQMHVQYNVDIEKFQTKPPVPAFGLHTKA-----------IFHGRNQSHISYVACQNWINQNESERSGMAVLDVAIPTGYWIQQQKLDTYVLSNRVRNLRRARYLERKIVFYFDYLDHEDICVNFTIERWYPVANMSYLPVRIYDYYAPERFNESIFDAL**

CfCD109-2 **QTKTLTINDKNLARLQTIEIPHGDAWGTVRVQAKGAGYAILQMHVQYNVDIWRFQTQPPVKSFDLVTRA-----------NFHGRNQSHISYFSCQRWTNLNESARSGMAVLDVAIPTGYIIQQQTLDKYILSKQVRNLQRARFQDKKVLFYFDYLDYEETCVNFTVERWYPVANMSYLPIRVYDYYSPERFNESIFDAL**

TcTEP4 **KTAVLHVNDKNRAKLQHIDIPN--AWGTVKVQAKGTGYAILQMHVQYNVDIARFQTKPPVPAFDLWIRP-----------YFYGRNMSHITLFSCQRWNHLGESPRSGLAVLDVTIPTGYIVQQQDLDAYILSRQVRNLQRARFSERKVLFYFNYLDYEETCINFTVERWYPVANMSYLPIRIYDYYAPERFNETIFDAL**

BgMCR1 **WAATFNLKKLDYIRLKTNSLPADKVWGFIVPNAQGTGRALLQLTTTVNVEYMWLQKKPMRPNNDPNEEPIKFFDLIVEDLRFSGRNDSIMEMTNCVSWLYTEKSLTSGLAVLEVDIPTGYVVMNDTLRDYVRSNRVPSLKRAEHYDRKVIFYFEYLDESKTCVYFRADRWFPVANATQHRMRVYDYYEPGMHNTTLYTTR**

BgMCR2 **WKNSAYIVRNNFTQLYQIPLPLNEVFGYVIPSAKGVGRALLQLTVTSNVEYEELMKTQQHYNNNPQEDLIPFFDLQVEA-RWGGRNDSIMFMRSCISWLYTERSLTSGLAVLEVDMPTGYIVMNDTLRSYVQSRVVPNLKRAEFYARKTVFYFEYLDTSKTCVDFRADRWFPVANSTEHRIRVYDYYEPGMHRTRLYTVQ**

1810 1820 1830 1840 1850

....|....|....|....|....|....|....|....|....|....|....|....

DmMCR **PTYLLNICEVCGSSQCPYCSIYNMGWRASMSMSLLFFSVFIYLLRSRTHLVLNMMQLLT**

CfCD109-2 **PTYTLNICEVCGSSQCPYCPIYNAATLLATPAGFLLIASLVVTITRYFRTQQFSDG---**

TcTEP4 **STYNLDICHVCGSSQCPYCWIYNATTRVTIPL-FTLIFTSIILIVRYFGVQDNII----**

BgMCR1 **NLFLLNICFVCGSYQCPYCPYFNSATIMSAGFSVVLLMAATYFVQRLLLRNR-------**

BgMCR2 **NLFLMNICFVCGSYQCPYCPYFNRSVLIIG--SNWSLFVVVMYIIHLCFQ---------**

**D) ClustalW alignment of BgTEP and BgCD109 proteins**

_________________________________________________

10 20 30 40 50 60 70 80 90 100 110 120 130 140 150 160 170 180 190 200

....|....|....|....|....|....|....|....|....|....|....|....|....|....|....|....|....|....|....|....|....|....|....|....|....|....|....|....|....|....|....|....|....|....|....|....|....|....|....|....|

AgTEP1 **MWQFIRSRILTVIIFIGAAHG--------------------LLVVGPKFIRANQEYTLVISNFNSQLSKVDLLLKLEGETDNGLSVLN--VTKMVDVRRNMNRMINFNMPEELTAG-------NYKITIDGQRGFSFHKEAELVYLSKSI-SGLIQVDKPVFKPGDTVNFRVILLDTELKPPARVKSVYVTIRDPQRNVI**

AfTEP **MLWVGLTTLLLGLAAAKDS----------------------YVVITPKDVRPGVSLNISVNILQAAGDVHVTAKLIHVADKSVKAFS------TGTFQQHVPDTMQIMIPDMIPSG-------TNQLTVEGSNGLTFSGKTNLHYASKGM-SVFIQTDKAMYKPGQTVNFRAFAIFPNLTVYSG--PLDIEIYDPNSNKI**

BgTEP1 **MKLNLILFVFYLVFQECQGG--------------------KYFISAPRNVVPGTAYDISVDILKQDIDNVTVEAILQDYSFSIPEGPKSLLTANGTFSPGVRGTLSMPIDFNLHCS-------YCRILLKGYNPLQFEQDIFIQIS-SDILSILIQTDKAIYKPKERVNFRILAAYYNLQLYTG--TFHYEILDPYDNKI**

BgTEP2 **MWKLILLAVVIATASATNS----------------------YVVIAPSKVRANMDLSLSVNILNATGDVTVVASLLREQTTVVSAT--------KVFQEGSPGTLNMKLPADLPSS-------TYTLNVKGSGGLTFDKSENLNYNNKET-SVFIQLNKAIFKPGDTVNFRVFGVYSDLKSYTD--PIDISIYDANSNKI**

BgTEP3 **MWKLILIAVVITSGRAQNIPGRQQAASNSTQRKSTWRD-CTYMIIAPSKVRANMDLSLSVHILNASSNVMLLVTLSQGQKTVVSAN--------KVFRQGAPEIFKLKLPADLPNS-------IYTLKVKGSGALTFNQSTDLSYNSKEAFSVFIQINKAIFKPDDTVNFRVFGIYPDLKSYSG--PMDVSIYDANSNKI**

BgTEP4 **MNQIWLAAAFLAAAIVHFPAQCQLVPIQDESTTPLPLKNATYWMTVSSTVRQGQPLEFRGQILVGSDPVSVTVTLLNGEGTKTLKTSP-----AITLSPGAVQSFKVDVPENIMDLSGEEYLYQIKVQMVGKGKTVNFKEEVLLTYESKSFFTFIQTDKAMYKPGQTVKFRVLSMTPDLKVIRDN-SNDIIIEDSNKNKI**

BgCD109 **MSWTSTSALCLLCAYSTLWITCYG----------------SFMVLTPKSVYPGIPLGVSVTAHKVVTAPVSVALSLETVQHERSIGN-----AETILLPGETKLLTIQVPLLNYTSP------FLQLKVSATGGFRDSQTKMISINQNTS-LILVQTDKAIYKPGQKVRIRVVNVDRYLKPVFN--PLTVIIENAKNDKL**

**A2M-N**

_________________________________________________

210 220 230 240 250 260 270 280 290 300 310 320 330 340 350 360 370 380 390 400

....|....|....|....|....|....|....|....|....|....|....|....|....|....|....|....|....|....|....|....|....|....|....|....|....|....|....|....|....|....|....|....|....|....|....|....|....|....|....|....|

AgTEP1 **RKWSTAKLYAG--VFESDLQIVPTPMLGVWNISVEVEGEELVS-KTFEVKEYVLSTFDVQVMPSVIPLEEHQAVNLTIEANYHFGKPVQG-VAKVELYLDDDKLNQKKE------------LTVYGKGQVELRFDNFAMDAD-----QQDVRVKVSFIEQYTNRTVVKQSQITVYRYAYRVELIKESPQ-FRPGLPFKCA**

AfTEP **KQWFGMKDSSG--VITNFMAMDTKPVLGDWKIRVKTYGGLTKD-KMFTVARYVLPKFEVTVDLPSYDWTTATSILGAVKAKYTYGKPVNG-TVKIRAHADFYHYNYYHPAPIP---TIELTMDINGETKFTLPVSGLTSHTYYTSLNSRNVVVEANVTESLTQITLNGTGKMHFYTHAEKIELLPSNPTTFKPGLQYIAY**

BgTEP1 **NVLSGVSGTFG--VVEGFFDLSDQPSFGTWKINVRTETVSGAESQFFEVAEYDLPRFQVDVGLPPFALLSDTTLSGSVEAKYTFGQPVYG-LVLLQIGENVDTIDKCNVNRKVT----EISFEIKGKGNFSVPLEDIRRSVHLN--EKKKIKITAFVTEASTGIKLNGSSVITYYGNRYQIKFLEMTPAVFKPGLQYTAY**

BgTEP2 **KQWLKVTPTNG--VITQELTLSTQPVLGDWKISVDAG-RTKEE-KVFTVAEYVLPKFEVDVVMPSYALTTDNDVTVTVKSKYTYGKPVNG-TADVLVKLHESFNTFDYSRALP---VTTLQVPLNGEAKVTIPMSQVKAINPY--LNQHVLIVIANVTESLTGNQMSGNGTVTLYDKGVKLDFPESNPKTFKPALQYIAY**

BgTEP3 **KQWNKVNPVNG--VFTQKLVLSAQPVLGDWKIEAKAN-TTRAQ-KIFTVAKYVLPKFKVEIVMPSFALTSDNDITVTIKSRYTYDKPVKG-TADVLVKLN-QFENSMPIDFAQSLPVTSLQMSINGEAKVTVPKDLINAK------DKDVLIVIANVTESLTGNKMTAKNTVTFYDQGAQLEYPKINPTSFKPGLKYSAY**

BgTEP4 **RQWQGVKDPNGRGVMELSLKIAKQVVFGDWTITVKTKGTETLK--TFTVQEYKLPKYEVMITTPPFGIISDPVLPITVKAIYTFGQPVSKGTVDVVITLVYSLKPEIKISGLLN-KDGEFTLQVSSKQLLGLVSNGQTDLN------YQSFKINANVTETDTGRNEGSSVTIIYYKTPLQLTFLGISPNNFKPGLGYTAY**

BgCD109 **EEYKDVNSKNG---------------------------------------------------------------------NYTYGKGVQG-QCELTVHYTASSQE-------------IYHKELNSDGVAVFDHLDWKKLSRN----VDNITVQAAVTD-ETGRKEQGETTLAVYADPKRVRILDTSTTILRHGLPAHIY**

________________________________________________________________

410 420 430 440 450 460 470 480 490 500 510 520 530 540 550 560 570 580 590 600

....|....|....|....|....|....|....|....|....|....|....|....|....|....|....|....|....|....|....|....|....|....|....|....|....|....|....|....|....|....|....|....|....|....|....|....|....|....|....|....|

AgTEP1 **LQFTHHDGTPAKGITGKVEVSDVGFETTTTS---------------------------------------------------------DNDGLIKLELQPSEGTEQLGINFNAVDGFFFYED---VNKVETVTDAYIKLELKSP---IKRNKLMRFMVTCTERMT-FFVYYVMSKGNIIDAGFMR--PNKQTKYLLQLNA**

AfTEP **AKVVQQDDMPLAAGSSKSLTVHTSVTANLP---------------------------------ETTTPLYYYGPRTMNYQLPDQSFTITDTGLVQAKIDIPDNATSISLQFKY-GQITQYHS---VQRSYSPSDSFIQIFLESNNLQAGHDKVVDFRVVSTSPID-KLVYQVLGRGSIAVSGAIN--GNNAKAFPFNVPL**

BgTEP1 **VQVTTPDGLPPTD-SNLSLSVYTSVTYQMT---------------------------------VPDQELYSPSSFSGSYPLPGQNMSLPANGILSIDIDIPLNATSIDIKVSLNKETTAEKR---ISKSYSMSNNYLQLSLLSKLVKAESD--VLIKITSTEAID-SLAYEIRSRSDHVKSGVLE--LSGQREFNATFKV**

BgTEP2 **LKVTQPDGLPMTS-TAEQVKVSIRVTAELPG--------------------------------TTPTPYYWYVPPTESRDLPALSLAIPDNGLVAIPVDVPADAKDVHVTANF-QGVSKELT---LGKSHSPSNSYIQLILKSGSVIKAGD-SISFEVKGTQALT-KLVYQILSRGGIVKTGTVD--ANGQLVYQFSIPS**

BgTEP3 **LQITQPDGLPTTS-ITEPVRISWKVEKEK-------------------------------------------------KPIDSIRVRLPKNGLVSFSVNVPLNASSLIISATF-QGVTKELI---VEPSYSPSSSYMQLALKTASVIRAGD-VVFFEVTSTTPMT-QLVYQVLSKGVIVKVGSEN--ATSKFSHQFSVVS**

BgTEP4 **LEVKKKDDTLFTLAEASQIRLLINVTYTVQLNKEEMAQREKELNISKSNIDNTSGTDEKQLLIRPGFIPYYDKTKTLILTINDPIRTVPDNGLIPINLDIPMEAESVSIEVNGLEPFAAEKAYKSVSKMKSPTGTYLQLKVPTETPKVGST--IKVTAVATEVIT-KLNFQVYSKGQLLLSEIINNPQSNTKSVEYSFTI**

BgCD109 **IEVSDHSGNPVSPVTLMMDVTHPELKGFTEVLN---------------------------------------------VPAGETIVKYTFIAIKSQEQSYYYNRGDGTLKAWLQMNDNVFDSKTFTVYRTKSPLALSILPLESQTIRVGESAIIKVNTSLPSYFDSTFAYLVMSQGNIVSAGQLK-------DNSFVITP**

**A2M-N2**

_________________________________________________________________________________________ ------------------------------------------------------------

610 620 630 640 650 660 670 680 690 700 710 720 730 740 750 760 770 780 790 800

....|....|....|....|....|....|....|....|....|....|....|....|....|....|....|....|....|....|....|....|....|....|....|....|....|....|....|....|....|....|....|....|....|....|....|....|....|....|....|....|

AgTEP1 **TEKMIPKAKILIATVA---GRT--VVYDYADLDFQ-ELRNNFDLSIDEQEIKPGRQIELSMSGR---------PGAYVGLAAYDKALLLFNKNHDLFWEDIGQVFDGFHAINEN---------------------------------------EFDIFHSLGLFARTLDDILFDSANEKTGRNALQS-------------**

AfTEP **NAKMAPNARIVAYYVR---ADG-EIVTDSISFDVSGTFENDVSIRFDKSKAQPGDGINVDVSAD---------PNSIVNLLAVDQSVLLLKSGNDITPAEVVDELKSYDTIVHSNNNGPIFFGGGGIMPEPMPVGRRKRMIWWPFTTYYGGSDAEQIFQNAGVNVMTDALVYHHVEPHIYLPTFQHHGFLGGFGGFESVH**

BgTEP1 **EPSWAPIAQLLMYYIR---RDSNEVVTDSLAFNVEGMFKNKVNVAFKENETDINKNVSLELSAD---------SDSQIYVLAVDQSVLLLKTGNDLTPNKVKDSFISKFHKGAIPTDS-------------------------NFALSYSGSSINEVFSNMGLVIATDLNIFAPFRPIALGRFPSSGFDRQGMMGAPMAM**

BgTEP2 **DSSMAPNARIVLYYVR---ADG-EIVTDSISFDISGAFKNKVSIDLDKTDVEPGDDVTVTVKAD---------PDSTAYCLAIDQSVLLLKGGNDVTDNDVYTELKEYDTITESSSNK--------GIIDCPMCKRRKRMIWWPFPTYYGGSDAQQIFSNAGVVVLTDATVYHYQEPIHLFNIPNFFQCGRSLSGQLRKR**

BgTEP3 **DSSMAPSARMVIYFYR---RDG-EIVIDSISFDVSGAFKNKVSFGFNSKSVEPGNNVTVTVRAD---------PNSAAYLLAIDQSVLLIRGDNDVTSDDVFTDLKKYDTAADS--------------ADCSTCKSG----LWPSWT-LGGAVALEIFTKAGVVALTDAVIIQSKP------------SDENKDGLIRN-**

BgTEP4 **SQAMAPTLTIIAFFMK---AENSEFVVDSLSIGVDGLFQKPITVEFSKTQVKPGEKVDVTIKAE---------SDSIVYLLGVDKSVQLLKSGNDITQAMVQEELMGYGTSGDYGMWR--------------------FMFFCGWPSYFGGTDAKSILSSAGVHIITDGLVYKSAFDNSFATDSGDLEMQKQPEPDEARF**

BgCD109 **TLEFCPLSRLLVYMIAGSESENGEVVLDAVDLTLTGCFTKEVKVEFEASETRTGTEVEMKVDVSRLDGSNEMPGQHDVFYLAVDQSIVLLQGSTDLNTDKVVSGLSSFDQVDESVTLS----------------------------------SAAAYFERHKLFYLTDASVWSRNQLFEKELMINRGPMLKKTSATELLP**

Protease Cleavage site **A2M**

----------------------------------------------- ________________________________________________________________________________________________

810 820 830 840 850 860 870 880 890 900 910 920 930 940 950 960 970 980 990 1000

....|....|....|....|....|....|....|....|....|....|....|....|....|....|....|....|....|....|....|....|....|....|....|....|....|....|....|....|....|....|....|....|....|....|....|....|....|....|....|....|

AgTEP1 **------------------------------------GKPIGKLVSYRTNFQESWLWKN--VSIGRSGSRKLIEVVPDTTTSWYLTGFSIDPVYGLGIIKKPIQFTTVQPFYIVENLPYSIKRGEAVVLQFTLFNNLGAEYIADVTLYNVANQTEFVGRPD------TDLSYTKSVSVPPKVG---VPISFLIKARKLGEM**

AfTEP **ALAGVSSAVNSIGMAPGAGSAAIPSHPQPQHIDNHATQDLKEPARVRLVFPETWLWTN--RTVGADGHVTIAATVPDTITSWVASAFAVHPTSGLGIAPTSAKVEAFRPFFVSLTLPYSVVRGEQLVLQANVFNYMTTDMDVVVTLEKNDDLVNVVFDTQGAESYIAQT-TAKTVHVTAGGS---KSVFFPVVPAGLGSV**

BgTEP1 **SFRDDNAMESAS-------------------FEMDVATSTKPVERVRSFFPESWLWTS--VKS-INGHATLTTTVPDTITSWIVSAFATNSDTGLGVAPTTSKLRVFRPFFVSLTYPRSVTRNEQFIVQATVFNYLPVDLMVTVSLKENPFLTPITPGP---------GNQASNIQVRANEQ---GIVYFSLSALTVGSL**

BgTEP2 **CFASFSPVMNLV------SSVVSDPITLPVTETETKTEDLQQPTKTRSNFVETWLWNS--LDIGANGSASITATVPDTITSWVASAFAINSASGLGVAPTQAHLRVFRPFFVSLNLPYSVTRGEHLALQANVFNYMTEDMQVRVTLAKSDNFFNIEIDANGAE-VLKQVESVQDVMIKAGEA---KSVYFPIVPADLGKI**

BgTEP3 **------------------------------------PGAIMVPKQIRQVFSETFLWSD--LTIGVNGSASITATVPDTITSWVASAFAVNSESGLGIAPSQSYLRVFRPFFVNLNLPYSVIRGEHLVVQANVFNYMTEDMQVTVTLAASDKFYNIEIDANGANGLFQQSQSVKVIMVEAGEA---ISVYFPIFPHELGKI**

BgTEP4 **S---------------------------------GSNLVLTNVVKRRKYFSETFLWAMEIINADSNGQVTLSVTAPDTITTWVVTAFSAHPVYGLSIVKESANLTTFRDLFVSLDLPISIIRNENFCFVATVFCYNKEEIPVLLTLDKSDNFSNIHVKVENGQVILSKESLHYSHFLGYLAERDISSVKFCFMPTALGDI**

BgCD109 **DFDSEVDG-------------------------KVPEAAYQTSARIRKDFPDTWLWGQ--AVTDVNGHLRSKVVLPDTITSWIVSAFAVNSE-GLAVAKEPFKLTAFQLFFLSMNLPYSIKRGEVFVLRVTVFNYRSQHVQAVVSLAHSDQFMVVDETES-------EGWYSKSLSLEAYRA---SSVSYRINATTLGQI**

**Thioester domain**

______________________________ _____________________________________________________________________

1010 1020 1030 1040 1050 1060 1070 1080 1090 1100 1110 1120 1130 1140 1150 1160 1170 1180 1190 1200

....|....|....|....|....|....|....|....|....|....|....|....|....|....|....|....|....|....|....|....|....|....|....|....|....|....|....|....|....|....|....|....|....|....|....|....|....|....|....|....|

AgTEP1 **AVRVKASIMLGHETDALEKVIRVMPESLAQPKMDTSFFCFDDY-KNQTFPFNLDIN-KKADNGSKKIEFRLNPNLLTMVIKNLDNLLAVPTGCGEQNMVKFVPNILVLDYLYATGSK-EQHLIDKATNLLRQGYQNQMRYRQT-DGSFGVWEKSG-----SSVFLTAFVATSMQTASKYMN------DIDAAMVEKALDW**

AfTEP **SINVKAQSTLA--ADAVRRQLLIEAEGVPKEYNIPMLVDLKHN-TNFAETVDVTLP-AGVVAGSQRVRISAIGDLMGPTVNGLDKLLRMPTGCGEQTMLGFAPDVFVTNYLTDTHQL-TSSVEEKAINFMEKGYQRELTFQHK-DGSFSAFGDND---PSGSMWLTAFVAKSFHQAKRH-------VFIDDETLTRAIDW**

BgTEP1 **DIEVSARSNMA--ADAIVRQILIKHEGAPVVYNNPILINLSNNQSTFEKNIAFTLP-DSLVPESQRIRVKVTGDLIGSTVQSLTSLLTLPTGCGEQSLVKFTPNIHIGRYLKATNQL-SEELNKKIIDLLNDGYQRQLTYKRY-DNGFSAFGNYD---ISSSTWLTALVVTSFAEAQEF-------IFVDKEIILKASML**

BgTEP2 **DIEVKAQSTKA--ADAVRRQLLVEAEGVPKIYNVPVLIDLTEGKTSFSKTVDLTLP-SNTVKGSELARISAVGDLMGPTIAGLDSLLQMPTGCGEQTMIGLAPDVYVTDYLKSVNQL-SGDIQTKALSYMESGYQRELTYKHT-DGSFSAFGNSD---ASGSMWLTAFVTRVFKQAKAH-------IYIDDEVLIKAIQW**

BgTEP3 **DIEVKAQSTKA--ADAVRRQLLVEAEGIPKTKNYPILIDLTEGRTSFSQTLNLPLP-SNTVKDSQRTRFSVVGDLMGPTIAGLDALLQMPTGSGEQNMVNLAPNIYVVNYLQSVNQL-STDIKSKASNFMEKGYQRELMYRHP-DGSFSNFGSND---TSGSIWLTAFVVKIFHQAQGH-------IYIDDNVLIEALQW**

BgTEP4 **PLRVSALTNVPGLSDAMEQIITVKPEGAARSTSNSYLIDMATG--RWEMNVTVKFP-AATVTGSETIIFNTAGNLLGPMFDNLDDLLKKPYGCGEQNMLNFAPNIFLLEFLFSTNKN-RSVAMEKAKDNMLIGYQKEITYEHSNTGGFSAFGHHEGSKDSASSWLTSFVVKCFAIAFQLDAAQGNVITIEKEIIQRSVRF**

BgCD109 **TLHVTATDPADGQKDEVKRELLVKPEGVERSRAITKVMILNSG-KSLSETFNIKWPQEKIVPDSQRVEIKVTGEVFGQALSGLENLVSIPFGCGEQNMISTVPNIFGLKYIRGTSQDGMEDLAAKLTNNMKLGYQRQVENYRHEDGSYSAWGDKFGNAESGSTWLTAFVIRSFAQASKF-------ISVDTNVLETGIEF**

**A2M-COMP**

________________________________________________________________________________________________________________________________________________________________________________________________________

1210 1220 1230 1240 1250 1260 1270 1280 1290 1300 1310 1320 1330 1340 1350 1360 1370 1380 1390 1400

....|....|....|....|....|....|....|....|....|....|....|....|....|....|....|....|....|....|....|....|....|....|....|....|....|....|....|....|....|....|....|....|....|....|....|....|....|....|....|....|

AgTEP1 **LASKQHSSGRFDETGKVWHKDMQGGLRNGVALTSYVLTALLENDIAKVKH-------------AVVIQNGMNYLSN-----QLAFINNPYDLSIATYAMMLNGHTMKKEALDKLIDMSISDNNKKERYWGTTN---------------------------QIETTAYALLSFVMAEKY-LDGIPVMNWLVNQRYVTGSFP**

AfTEP **MINRQAANGSFPEPGRIIHKNMQGGSASGASLTAFVLIALLENSDLQGGVHMR---------IQSAASKAQAYLE-----GEVSAMTDPYGLSICSYALTLASSQSSATTFQKLMAKAVTKDGMT--HWHEPES----A---APSTGHYWSPPHQQSKPVDIEMTSYGLMVFAHNSQF-TEGLPFMKWITKQRNPNGGFS**

BgTEP1 **LIDRQNIDGSFNEFGKVLDRNTQGTTTAGPALTAFVLVALLKAKELADVQDCKNNNKCRYYLLGNATLNATRNLERL---MLADSIDDQFSLAVASYAFAEAKSQLAQSTFEKLLTFVKQEGGLE--YWSANST----VNNEELNRFINWRPPRLQARPIDILITSYAILTYSSLGRL-DEALPSVRWLTLQKNAQGGFV**

BgTEP2 **MVSKQNANGSFPEPGNVIHKNMQGQAGSGVGLTLFVLISLLENKDLLVNTNAAG------VLVDEARQKALVYSE-----QEVAKTDDLYILNMAAYAFQLANSSQVQTVLNKLEQKATVKDGRK--YWHQPE---------QPKTTNTWDYPN-PTKAVDIEMTSYALLTYAARGNI-VAGKSIMQWLTEQRNSNGGFS**

BgTEP3 **IVTQQNPDGSFQLPS-------KGQAGSSVVLTLHVLISLFENEDVLAEDN-----------IVEARGKALTFVE-----SEVDKTNDLYVLSLAAYTFQLAGSTRVQAVLDKLELRATVKGGRK--FWLLPE---------QPKKKTILNYPN-QTKSIDIEITSYVLLTYAARGNL-VAGKSIMWWLAEQRNSQGGFP**

BgTEP4 **MISQQNLNGSFTEKGKVFHKEMQGGSAEGEALTAYTVIALYEAQKVFASGDSIVAN------ISQSIKLGVDFLVR-----RLPFLTDPYDICIVTYTLHLVNDNNKETAFNKMQSIAITGDGLR--YWKRAT---------PAESNIAKYEWTASADSISIEMTSYALLVYAFREIANTEGLPIVRWITNHRGPNGGFI**

BgCD109 **LKSCQDRTGKFIERGQVFHSDMQSGTGSGDGLTVYVLISMLEASQALGETGSLS--------FKNQIDLALNYIRRNQDPEKLKQEKQIYLAAITAYSLSLVSNK-DKDILQLIEQLLMVIKELQVPWSKVDSQDIKTLQSKQAAGDVGPPYIVKAQATRDLEIGAYVLLTLTRIENL-AEGLELMKWLQSQQNSKGGFY**

**A2M-Rc**

__________________ _______________________________________________________________

1410 1420 1430 1440 1450 1460 1470 1480 1490 1500 1510 1520 1530 1540 1550 1560 1570 1580 1590 1600

....|....|....|....|....|....|....|....|....|....|....|....|....|....|....|....|....|....|....|....|....|....|....|....|....|....|....|....|....|....|....|....|....|....|....|....|....|....|....|....|

AgTEP1 **RTQDTFVGLKALTKLAEKISPSR-----NDYTVQLKYKKNTKYFNINSEQIDVQNFLEIPEDTKK---------LEINVGGIGFGLLEVIYQFDLNLVNFEHRFKLDLEKQNTGSDYELR----LRVCANYIPELTDSQSNMALIEVTLPSGYVVDRNPISEQTTVNPIQNMEIRYGGTSVVLYY--YKMGTERNCFTVT**

AfTEP **STQDTVLALQSLSEFARIGYSEH----FDMQIG-IVAGQTTHTFSVTRQNALLLQSLELPSIPSH---------VTVTGTGSGMGLVEVSVFFNVEQEVEQPSFEVDVTIMEETINSLK-----VRSCTKWLKTGASG---MTVQEVGVPTGFAPDVESIGKIATLKKTE-----TENRKVILYF--DEITTTPLCVTMN**

BgTEP1 **STQDTVVGLQALSSYGSKSFRPD----TNITIY-VSDMNTHLTMNVNSENALSLQIQEIQSNSQD---------FSITASGSGLALLDIEYSFNVLKELSKPVFDVNTVLLDDKLDSFN-----IMVCTKFLMKHDTG---MVVQELSIPSGFVPDLSTLGQVAGVKRSE-----RKGSIVAIYF--DKISGSSLCYSIV**

BgTEP2 **STQDTVLALNALSEFAKQTYSNN----FNVQITTQLNATTSYTFNIDKTNSLLLQSRETPDVPSQ---------VKIDATGSGMALVQVAVSFNVESEIFETTFDLTVKLIEETINNLF-----VETCAKWLGSGPSSA--MAVQEIGIPSGFEADLESIPQLDILKRIE-----TQNKKVILYF--DQIGTTPVCLNFR**

BgTEP3 **TARCSIIALNALAVFAEKTYRNN----FNMKITAKVAPQKMLQYRIDRTNALILQSGEVSDVPAQ---------VQIEATGSGLVLAEIAVSFNVESEIFRTTFDLKVTLVEESMNYFI-----LQTCTKWIGSESDGV--MTVQEIGIPTGFEADLDSIPNLENLKRIE-----SQFKNLYLYI--DQIDSTPVCLTMK**

BgTEP4 **STQDTVIGLQALARVAAKIYSNEDIPITLAVSYESKGQLVKEIIKINKSNEMLLQSVDINYKDEQPNFVNIVATTDSGKTGPSTVIAEIVLGYNILAETSAKFYDMSHTLDKLSAG--------FVLTILIKTTKDSSS--MCILEVDIPPGFTPDSDALKLNKAISLSE-----ILGDVLAIYFNTDMITTKETPVKIF**

BgCD109 **STQDTIMVLQALSEFGSKFRPGE------VSSQLQVTHPVNLAFTLSGSRALLLQTATLPWDTTKVN-------VTLTGGTNSLAVVKVVYTYYTFAGDDDQVPTETLLFLETKSIRLGNGMHKVEACVKSSKSLKYKG--MFVTTMALPSGEKPADDQSTILASNPMASR----VEADEKFIHFYIDKAPSNEGYCLTA**

________________________________

1610 1620 1630 1640 1650 1660

....|....|....|....|....|....|....|....|....|....|....|....|....|...

AgTEP1 **AYRRF-KVALKRPAYVVVYDYYNTNLNAIKVYEVDKQNVCEICEEEDCPAECKK--------------**

AfTEP **AYRTD-QVAKSQPAPIRVYDYYEPSNQVTKFYQSTVLKNSGVCDLCKECGCHGQH-------------**

BgTEP1 **MTREA-KVAKSQKSYVRTYDYYEPANQATVFYQPRTLRDSTVCDVCLNCCP-----------------**

BgTEP2 **AVRTG-LVAKSQPAAIRVYDYYEPRNQVTAFYQSQILKDSTCVVCKEECATV----------------**

BgTEP3 **AVRIG-VVSGLQPSTVRVIDYYEPSNQVTAFYQSQILEASIICDVCKECDNC----------------**

BgTEP4 **MVSTGGVLTKSQPRMYRVYDYYTPDRELSKNYLLEDTDFCTAAPDVGGCQYRQK--------------**

BgCD109 **NVEPHLEFEVQKPGFAQFYTYYDPDNVAEVPLSLTCHNCDTDTAVMVNMASVLLTTVVCLLASLLACM**
