# Supplementary material for: A New Assessment of Thioester-Containing Proteins Diversity of the Freshwater Snail Biomphalaria glabrata
Source: Genes (Basel). 2020 Jan 7;11(1):69. doi: 10.3390/genes11010069 (PMC7016707; doi:10.3390/genes11010069)
Supplement: Supplementary file 1 [file genes-11-00069-s001.zip › Figure S4.docx]

**A) BgC3-1**

10 20 30 40 50 60 70 80 90 100 110 120 130 140 150 160 170 180 190 200

....|....|....|....|....|....|....|....|....|....|....|....|....|....|....|....|....|....|....|....|....|....|....|....|....|....|....|....|....|....|....|....|....|....|....|....|....|....|....|....|

BgC3-1 **MPSIRFTRSTSRFIFCTHLLFLLVFVNMMKNCFSTKYLLTVPKEPTYDTDLTVTITALTKPEVPENITLEFRGLKDKSKVLNSTQINFSEDETKNWTVVFPWERMLGLDETGVLLKMRTTNEMENVVEKDLSLKFRNTSGYIFIQTDKPIYTPRQTVKFRIIALDEYQRLTKYPIKVDIKNPQGVILERMRYSAEDAFKS**

BGLB021062-PA **--------------------------------------------------------------------------------------------------------MLGLDETGVLLKMRTTNEMENVVEKDLSLKFRNTSGYIFIQTDKPIYTPRQIVKFRIIALDEYQRLTKYPIKVDIKNPQGVILERMRYSAEDAFKS**

BGLB025256-PA **--------------------------------------------------------------------------------------------------------------------------------------------------------------------------------------------------------**

BGLB000085-PB **--------------------------------------------------------------------------------------------------------------------------------------------------------------------------------------------------------**

BGLB000085-PA **--------------------------------------------------------------------------------------------------------------------------------------------------------------------------------------------------------**

210 220 230 240 250 260 270 280 290 300 310 320 330 340 350 360 370 380 390 400

....|....|....|....|....|....|....|....|....|....|....|....|....|....|....|....|....|....|....|....|....|....|....|....|....|....|....|....|....|....|....|....|....|....|....|....|....|....|....|....|

BgC3-1 **QEFELPKDTPPGIWTISANLEGLGQLYSLAHTVAFEVREYVLPRFSAVFKIDTDVITMDTTWIRMNVTAKYVYGQPVVGKVEMRLGTWDENSSVTLIPSASYRGELINGVFKRDVKRSSLFPTNESFNGVKRLYVQVNVTETATQETITIEDTSTFVSHPYYEVDFTPSKTYFKPGFPYTVHVQVKARSGRLASWVLLYL**

BGLB021062-PA **QEFELPKDTPPGIWTISANLEGLGQLYSLAHTVAFEVREYVLPRFSAVFKIDTDVITMDTTWIRMNVTAKYVYGQPVVGKVEMRLGTWDENSSVTLIPSASYRGELINGVFKRDVKRSSLFPTNESFNGVKRLYVQVNVTETATQETITIEDTSTFVSHPYYEVDFTPSKTYFKPGFPYTVHVQVKARSGRLASWVLLYL**

BGLB025256-PA **--------------------------------------------------------------------------------------------------------------------------------------------------------------------------------------------------------**

BGLB000085-PB **--------------------------------------------------------------------------------------------------------------------------------------------------------------------------------------------------------**

BGLB000085-PA **--------------------------------------------------------------------------------------------------------------------------------------------------------------------------------------------------------**

410 420 430 440 450 460 470 480 490 500 510 520 530 540 550 560 570 580 590 600

....|....|....|....|....|....|....|....|....|....|....|....|....|....|....|....|....|....|....|....|....|....|....|....|....|....|....|....|....|....|....|....|....|....|....|....|....|....|....|....|

BgC3-1 **HPKFYDSEKHLLRESNNHVIVQPLDMYGKLSMEVQIPLNADRVIFSAIVMDFAKIKFNEYILNVSKLASDINEYIVISMPTPIQKIQRGEVILNYTKPRNLLDKITVLVIAKGYVIYTLKNITKNNNGSSTIYLPTSLRGDASPSMRIVAYYWTMGEIIMDSLFIEAPVKYCVEELYVNKGGLFSTTPLKPKDKLNIDLR**

BGLB021062-PA **HPKFYDSEKHLLRGKSLSFGE-----------------------------------------------------------------------------------------------------------------------------------------------------------------------------------**

BGLB025256-PA **-------------------------MYGKLSMEVQIPLNADRVIFSAIVMDFAKIKFNEYILNVSKLASDINEYIVISMPTPIQKIQRGEVILNYTKPRNLLDKITVLVIAKGYVIYTLKNITKNNNGSSTIYLPTSLRGDASPSMRIVAYYWTMGEIIMDSLFIEAPVKYCVEELYVNKGGLFSTTPLKPKDKLNIDLR**

BGLB000085-PB **--------------------------------------------------------------------------------------------------------------------------------------------------------------------------------------------------------**

BGLB000085-PA **--------------------------------------------------------------------------------------------------------------------------------------------------------------------------------------------------------**

610 620 630 640 650 660 670 680 690 700 710 720 730 740 750 760 770 780 790 800

....|....|....|....|....|....|....|....|....|....|....|....|....|....|....|....|....|....|....|....|....|....|....|....|....|....|....|....|....|....|....|....|....|....|....|....|....|....|....|....|

BgC3-1 **GGSNMRVGLVAVDKAVLLLNDKQTLTRKLLFNELEKHDQGTHKNNGTFEEILKSNGLQYIFLDTVQVESDDPPPIGTVKEDGFDETDSFIAMQQALPPQTVRSYFPESWMFEEHVLPKSGFLRLSWPLPDSITTWSVLVVGVSANRGVCVSEPVDQIAMKMFFADVHVPYKATRLEEVKVKIAIYNFYNYTLAVQGTVTS**

BGLB021062-PA **--------------------------------------------------------------------------------------------------------------------------------------------------------------------------------------------------------**

BGLB025256-PA **GGSNMRVGLVAVDKAVLLLNDKQTLTRKLLFNELEKHDQGTHKNNGTFEEILKSNGLQYIFLDTVQVESDDPPPIGTVKEDGFDETDSFIAMQQALPPQTVRSYFPESWMFEEHVLPKSGFLRLSWPLPDSITTWSVLVVGVSANRGVCVSEPVDQIAMKMFFADVHVPYKATRLEEVKVKIAIYNFYNYTLAVQGTVTS**

BGLB000085-PB **--------------------------------------------------------------------------------------------------------------------------------------------------------------------------------------------------------**

BGLB000085-PA **--------------------------------------------------------------------------------------------------------------------------------------------------------------------------------------------------------**

810 820 830 840 850 860 870 880 890 900 910 920 930 940 950 960 970 980 990 1000

....|....|....|....|....|....|....|....|....|....|....|....|....|....|....|....|....|....|....|....|....|....|....|....|....|....|....|....|....|....|....|....|....|....|....|....|....|....|....|....|

BgC3-1 **EPGLCISSNSSQTFNSGTSLKTLTFSMNIAAFQTASEIIKVIPLKVGELGLLVHVKSQKDEDIVKKTLHVVSEGLRVFKTITFVLDPEAKHATFQGRTHFSTIRNHIDKIKKQQFTTIDLALPKDVIKGTEFCGISAFGDLMGDIITHGIVRSKSFVDQPLVNAEEVIGDLGPAVFALQYVNDTKLLTDELKNKGQRFLL**

BGLB021062-PA **--------------------------------------------------------------------------------------------------------------------------------------------------------------------------------------------------------**

BGLB025256-PA **EPGLCISSNSSQIFNSGTSLKTLTFSMNIAAFQTASEIIKVIPLKVGELGLLVHVKSQKDEDIVKKTLHVV---------------------------------------------------------------------------------------------------------------------------------**

BGLB000085-PB **--------------------------------------------------------------------------------------------------------------------------------------------------------------------------------------------------------**

BGLB000085-PA **--------------------------------------------------------------------------------------------------------------------------------------------------------------------------------------------------------**

1010 1020 1030 1040 1050 1060 1070 1080 1090 1100 1110 1120 1130 1140 1150 1160 1170 1180 1190 1200

....|....|....|....|....|....|....|....|....|....|....|....|....|....|....|....|....|....|....|....|....|....|....|....|....|....|....|....|....|....|....|....|....|....|....|....|....|....|....|....|

BgC3-1 **QGITRLLNYRKENAFSLHTDSSPATWLTASVVKILCHIEKANLTFIDKENLIDNAINWIMRQRREDGSLKEADPRLSQESLQYKIMLAADVMISLLECNKDEEQEAAELIMGLVTFIENNIDNINNSLALAKAAYAMKLFEIDSESTAQIITKLKEFMKRDKLSRLYWSDSVIDNPPRQPIWYHQGATAAAIEATSYALL**

BGLB021062-PA **--------------------------------------------------------------------------------------------------------------------------------------------------------------------------------------------------------**

BGLB025256-PA **-----------------------------------------------------------------------------------------------------------------------------------------YMKKKRLDLNSNKKKGKNENSILPPTGRLYWSDSVIDNPPRQPIWYHQGATAAAIEATSYALL**

BGLB000085-PB **---------------------MYCIRLTASVVKILCHIEKANLTFIDKENLIDNAINWIMRQRREDGSLKEADPRLSQESLQYKIMLAADVMISLLECNKDEEQEAAELIMGLVTFIENNIDNINNSLALAKAAYAMKLFEIDSESTAQIITKLKEFMKRDKLMSNSGNQLMNVDWYRSIGSQAFDVANSGPLVWMTHHV**

BGLB000085-PA **-----------------------------------------------------------MRQRREDGSLKEADPRLSQESLQYKIMLAADVMISLLECNKDEEQEAAELIMGLVTFIENNIDNINNSLALAKAAYAMKLFEIDSESTAQIITKLKEFMKRDKLSKSKKSSGHFVYTVVPSKKSSTETILPMDKWKASIRE**

1210 1220 1230 1240 1250 1260 1270 1280 1290 1300 1310 1320 1330 1340 1350 1360 1370 1380 1390 1400

....|....|....|....|....|....|....|....|....|....|....|....|....|....|....|....|....|....|....|....|....|....|....|....|....|....|....|....|....|....|....|....|....|....|....|....|....|....|....|....|

BgC3-1 **VFLDHSMIKEEAIADWLVAQRNPSGAFIGAMDSTVAIQALTKYSQKRYVLSGNAVFLRGNITSDITRSRNHLHSFKFTEENATSPASVKNVPVGQVLEVFTEGQGLGQMHVNVEYNIPIEKNLQCHYNVTVEIKPIQYMPSSVNTSPLCQYCNIGCPANLRSRKEISDVSPIIRIGRARSNLSKSRAVRSSSHSKKVYCF**

BGLB021062-PA **--------------------------------------------------------------------------------------------------------------------------------------------------------------------------------------------------------**

BGLB025256-PA **VFLDHSMIKEEAIADWLVAQRNPSGAFIGAMDSTVAIQALTKYSQKRYVLSGNAVFLRGNITSDITRSRNHLHSFKFTEENATSPASVKNVPVGQVLEVFTEGQGLGQMHVNVEYNIPIEKNLQCHYNVTVEIKPIQYMPSSVNTSPLCQYCNIGCPANLRARKEISDVSPIIRIGRARSNLSKRRAVRSSSHSKKVYCF**

BGLB000085-PB **PALGTSVTVVIDVVKHLCDGHAAAFLCPELASKDWLGDSVV---------------------------------------------------------------------------------------------------------------------------------------------------------------**

BGLB000085-PA **GQVDNRK-------------------------------------------------------------------------------------------------------------------------------------------------------------------------------------------------**

1410 1420 1430 1440 1450 1460 1470 1480 1490 1500 1510 1520 1530 1540 1550 1560 1570 1580 1590 1600

....|....|....|....|....|....|....|....|....|....|....|....|....|....|....|....|....|....|....|....|....|....|....|....|....|....|....|....|....|....|....|....|....|....|....|....|....|....|....|....|

BgC3-1 **HVCLRFIRTEGNVPINIKMDMLSGFKPVASDLELIKSGPNVLHVEFQAGTETLVIQLSKVDTESPSCFGFRVVDDEEVERKVPAALIIQQVGHPVPSCTLEYHLPDSLESLKVFCADFSHINRGECRCYSGLCSKCRPTHANELDLDKTKKLVCKKEIAYQLRLGDVQDKIHWMEIDARVLSLNKTGSHKLEAGDTIKMM**

BGLB021062-PA **--------------------------------------------------------------------------------------------------------------------------------------------------------------------------------------------------------**

BGLB025256-PA **HVCLRFIRTEGNVPINIKMDMLSGFKPVASDLELIKSGPNVLHVEFQAGTETLVIQLSKVDTESPSCFGFRVVDDEEVERKVPAALIIQQVGHPVPSCTLEYHLPDSLESLKVFCADFSHINRGECRCYSGLCSKCRPTHANELDLDKTKKLVCKKEIAYQLRLGDVQDKIHWMEIDARVLSLNKTGSHKLEAGDTIKMM**

BGLB000085-PB **--------------------------------------------------------------------------------------------------------------------------------------------------------------------------------------------------------**

BGLB000085-PA **--------------------------------------------------------------------------------------------------------------------------------------------------------------------------------------------------------**

1610 1620 1630 1640 1650 1660 1670 1680

....|....|....|....|....|....|....|....|....|....|....|....|....|....|....|....|.

BgC3-1 **SPSSCSCLVNNYRDEDFYMLSKDVERLVDRRGETVYRYLLDENAEFLHVEKPGVPSTSSLVPPVPFPYLQQALSKDNVCRA**

BGLB021062-PA **-------------------------------------------**

BGLB025256-PA **SPTPLKI----------------------------YRYLLDENAEFLHVEKPGVPSTSSLVPPVPFPYLQQALSKDNVCRA**

BGLB000085-PB **---------------------------------------------------------------------------------**

BGLB000085-PA **---------------------------------------------------------------------------------**

**B) BgC3-2**

10 20 30 40 50 60 70 80 90 100 110 120 130 140 150 160 170 180 190 200

....|....|....|....|....|....|....|....|....|....|....|....|....|....|....|....|....|....|....|....|....|....|....|....|....|....|....|....|....|....|....|....|....|....|....|....|....|....|....|....|

BgC3-2 **MRDIMQTFWILLLLLVPVSCSSHHFVLLPSVLRLETEEVFSVTSLEAEGDVTFKIYLTDYPERKRNFSETTVTVPQGESVMARVLMTVGDLPQNAQPPLFVNVIVMTVDHEPHFQKEAVILVNESPGYIFIQTDKPVYTPDQSVFTRVMTLNEHFRPASWPLQVDIQNPDGMTISRKVIDSKDLILKDVMKIPENPVYGN**

BGLB018444-RA **--------------------------------------------------------------------------------MARVLMTVGDLPQNAQPPLFVNVIVMTVDHEPHFQKEAVILVNESPGYIFIQTDKPVYTPDQSVFTRVMTLNEHFRPASWPLQVDIQNPDGMTISRKVIDSKDLILKDVMKIPENPVYGN**

BGLB030758-RA **--------------------------------------------------------------------------------------------------------------------------------------------------------------------------------------------------------**

BGLB011300-RB **--------------------------------------------------------------------------------------------------------------------------------------------------------------------------------------------------------**

BGLB023169-RA **--------------------------------------------------------------------------------------------------------------------------------------------------------------------------------------------------------**

210 220 230 240 250 260 270 280 290 300 310 320 330 340 350 360 370 380 390 400

....|....|....|....|....|....|....|....|....|....|....|....|....|....|....|....|....|....|....|....|....|....|....|....|....|....|....|....|....|....|....|....|....|....|....|....|....|....|....|....|

BgC3-2 **WTVTAKFINGLRTTSAVRFEVKEYVLPTISVSFHIPDSRKVILPNETHFHLAVGAKYMYGKPVRGHVTVTYGLLWHGHVFTVGKQRNLQLNDTGFVECGITVDDLRLPVQSVWFPNGGKLHVQAAVTETASGHVEKADDTSVVFADHLYVIRFTRSDRHFKPGLPYVLEIDVFKANGETGPYLALSVECQIEMQDGTKET**

BGLB018444-RA **WTVTAKFINGLRTTSAVRFEVKEYVLPTISVSFHIPDSRKVILPNETHFHLAVGAKYMYGKPVRGHVTVTYGLLWHGHVFTVGKQRNLQLNDTGFVECGITVDDLRLPVQSVWFPNGGKLHVQAAVTETASGHVEKADDTSVVFADHLYVIRFTRSDRHFKPGLPYVLEIDVFKANGETGPYLALSVECQIEMQDGTKET**

BGLB030758-RA **--------------------------------------------------------------------------------------------------------------------------------------------------------------------------------------------------------**

BGLB011300-RB **--------------------------------------------------------------------------------------------------------------------------------------------------------------------------------------------------------**

BGLB023169-RA **--------------------------------------------------------------------------------------------------------------------------------------------------------------------------------------------------------**

410 420 430 440 450 460 470 480 490 500 510 520 530 540 550 560 570 580 590 600

....|....|....|....|....|....|....|....|....|....|....|....|....|....|....|....|....|....|....|....|....|....|....|....|....|....|....|....|....|....|....|....|....|....|....|....|....|....|....|....|

BgC3-2 **IPATGQYDGHSMVTDARGKLSVHYNIPANAKQLHFKVSPKEKATTDQASKSDYFFIASRFYSPSSVYMQLHARLTDTNRFGYTPAVGDHLTVWTSYTSPEEISTVTLVVISRGSIVWQVSTRNILGNSTYFHFKITQDMSPTAWILAFAVRGNEPGSEVISDSVWLEIVPQCDGELHIQRENDGKKVLKPGDIGTVTLTG**

BGLB018444-RA **IPATGQYDGHSMVTDARGKLSVHYNIPANAKQLHFKVSPKEKATTDQASKSDYFFIASRFYSPSSVYMQLHARLTDTNRFGYTPAVGDHLTVWTSYTSPEEISTVTLVVISRGSIVWQVSTRNILGNSTYFHFKITQDMSPTAWILAFAVRGNEPGSEVISDSVWLEIVPQCDGELHIQRENDGKKVLKPGDIGTVTLTG**

BGLB030758-RA **--------------------------------------------------------------------------------------------------------------------------------------------------------------------------------------------------------**

BGLB011300-RB **--------------------------------------------------------------------------------------------------------------------------------------------------------------------------------------------------------**

BGLB023169-RA **--------------------------------------------------------------------------------------------------------------------------------------------------------------------------------------------------------**

610 620 630 640 650 660 670 680 690 700 710 720 730 740 750 760 770 780 790 800

....|....|....|....|....|....|....|....|....|....|....|....|....|....|....|....|....|....|....|....|....|....|....|....|....|....|....|....|....|....|....|....|....|....|....|....|....|....|....|....|

BgC3-2 **QPYMVVGVVAVDSAVYRLKNSTLTRQSVFQQITAHDRGCGFGGGKDAAKVFENSGLMALTNADLPMTPKTVDGCVDKAVRKKRSPEARKRARDICCVEGARVRNATLALCYFATQELKKTMNSEFCVREFFQCCRSFVKGTLSLDALGRLRTSIERLPEDIELNFDEDDLSNMKNIPVRTNFPESWWFEEYNLGAEGRAD**

BGLB018444-RA **QPYMVVGVVAVDSAVYRLKNSTLTRQSVFQQITAHDRGCGFGGGKDAAKVFENSGLMALTNADLPMTPKTVDGCVDKAVRKKRSPEARKRARDICCVEGARVRNATLALCYFATQELKKTMNSEFCVREFFQCCRSFVKGTLSLDALGRLRTSIERLPEDIELNFDEDDLSNMKNIPVRTNFPESWWFEEYNLGAEGRAD**

BGLB030758-RA **---MVVGVVAVDSAVYRLKNSTLTRQSVFQQITAHDRGCGFGGGKDAAKVFENSGLMALTNADLPMTPKTVDGCVDKAVRKKRSPEASR**

BGLB011300-RB **----------------------------------------------------------------------------------------------------------------------------------------------------------------------------MKNIPVRTNFPESWWFEEYNLGAEGRAD**

BGLB023169-RA **--------------------------------------------------------------------------------------------------------------------------------------------------------------------------------------------------------**

810 820 830 840 850 860 870 880 890 900 910 920 930 940 950 960 970 980 990 1000

....|....|....|....|....|....|....|....|....|....|....|....|....|....|....|....|....|....|....|....|....|....|....|....|....|....|....|....|....|....|....|....|....|....|....|....|....|....|....|....|

BgC3-2 **VDFVLPDSITTWSVEALGMSVEAGLCVAPPLELTTFTSCFVHLDLPYSVVRLEQVEVRATVYNYMTKKIRVNLILQSVDGVCYSGQPGDATDYVKLEIDPNDAASAYFPIVPLEIGTFPIIVKAFSTWGRDAVEKTLRVEGEGLEKIHTISVMLDPSGKRFLRSRSSNHTFNMKNEVRSAEKKQNVELDLDLPQEVIPDT**

BGLB018444-RA **VDFVLPDSITTWSVEALGMSVEAGLCVAPPLELTTFTSFFVHLDLPYSVVRLEQVEVRATVYNYMTKKIRVNLILQSVDGVCYSGQPGDATDYVKLEIDPNDAASAYFPIVPLEIGTFPIIVKAFSTWGRDAVEKTLRVEGEGLEKIHTISVMLDPSGKRFLRSRSSNHTFNMKNEVRSAEKKQNVELDLDLPQEVIPDT**

BGLB030758-RA

BGLB011300-RB **VDFVLPDSITTWSVEALGMSVEAGLCVAPPLELTTFTSFFVHLDLPYSVVRLEQVEVRATVYNYMTKKIRVNLILQSVDGVCYSGQPGDATDYVKLEIDPNDAASAYFPIVPLEIGTFPIVVKAFSTWGRDAVEKTLRVEGEGLEKIHTISVMLDPSGKRFLRSRSSNHTFNMKNEVRSAEKKQNVELDLDLPQEVIPDT**

BGLB023169-RA **--------------------------------------------------------------------------------------------------------------------------------------------------------------------------------------------------------**

1010 1020 1030 1040 1050 1060 1070 1080 1090 1100 1110 1120 1130 1140 1150 1160 1170 1180 1190 1200

....|....|....|....|....|....|....|....|....|....|....|....|....|....|....|....|....|....|....|....|....|....|....|....|....|....|....|....|....|....|....|....|....|....|....|....|....|....|....|....|

BgC3-2 **ESCSVHAMGDLLGPTLQVMIEGVTELLRLPTGCGEQNLIYLAPNVFVTRYLRATRRLTSFIEKKALALIRQGVSKQMFFRKVDGSFATWPHAESSTWLTAFAMKTLCQAEHYVTVDHNQTCSSFHWIAKQQKPDGSFREEVWVTHREMLGGVNGDVSHAAFILIALLECDCPGNDHKDVTAQALRYIETTVAQTDRPLAL**

BGLB018444-RA **ESCSVHAMGDLLGPTLQVMIEGVTELLRLPTGCGEQNLIYLAPNVFVTRYLRATRRLTSFIEKKALALIRQGVSKQMFFRKVDGSFATWPHAESSTWLTAFAMKTLCQAEHYVTVDHNQTCSSFHWIAKQQKPDGSFREEVWVTHREMLGGVNGDVSHAAFILIALLECDCPGNDHKDVTAQALRYIETTVAQTDRPLAL**

BGLB030758-RA

BGLB011300-RB **ESCSVHAM**

BGLB023169-RA **-------------------------------------------------------------------------------------------------LTAFAMKTLCQAEHYVTVDHNQTCSSFHWIAKQQKPDGSFREEVWVTHREMLGGVNGDVSHAAFILIALLECDCPGNDHKDVTAQALRYIETTVAQTDRPLAL**

1210 1220 1230 1240 1250 1260 1270 1280 1290 1300 1310 1320 1330 1340 1350 1360 1370 1380 1390 1400

....|....|....|....|....|....|....|....|....|....|....|....|....|....|....|....|....|....|....|....|....|....|....|....|....|....|....|....|....|....|....|....|....|....|....|....|....|....|....|....|

BgC3-2 **AISAFALTLAGSPSSDGVVKRLQSMAKSSPEGYTYWSHGTEEDYEGHEKPYWYTKQPGALAVEVTSYALLTNLARGDISTSTGIVGWLLSQRNSQGAFISTQDTVVGLQALSEYSIKSYSAILDMTCHIRSEVDDHFRKSISLTPEDAMVVKTVPKVPTGGKLHFEAEGTGVGMMQVEVRFNVPEDRNNCHFDVTVATHQ**

BGLB018444-RA **AISAFALTLAGSPSSDGVVKRLQSMAKSSPEGYTYWSHGTEEDYEGHEKPYWYTKQPGALAVEVTSYALLTNLARGDISTSTGIVGWLLSQRNSQGAFISTQDTVVGLQALSEYSIKSYSAILDMTCHIRSEVDDHFRKSISLTPEDAMVVKTVPKVPTGGKLHFEAEGTGVGMMQVEVRFNVPEDRNNCHFDVTVATHQ**

BGLB030758-RA

BGLB011300-RB

BGLB023169-RA **AISAFALTLAGSPSSDGVVKRLQSMAKSSPEGYTYWSHGSEEDYEGHEKPYWYTKQPGALAVEVTSYALLTNLARGDISTSTGIVGWLLSQRNSQGAFISTQDTVVGLQALSEYSIKSYSAILDMTCHIRSEVDDHFRKSISLTPEDAMVVKTVPK**

1410 1420 1430 1440 1450 1460 1470 1480 1490 1500 1510 1520 1530 1540 1550 1560 1570 1580 1590 1600

....|....|....|....|....|....|....|....|....|....|....|....|....|....|....|....|....|....|....|....|....|....|....|....|....|....|....|....|....|....|....|....|....|....|....|....|....|....|....|....|

BgC3-2 **HNTLLQSFFWDNRKSKCEPCSTDCEEQTSEEEEEEDYEDFTFPPVQPRIQTLWKKKVKGLNLTVHEDENRSDFEDPNMVSKIGRPRRKRRSVRPYSASVICVEVCVRFLGNKTTGMSVVDVGLFTGYLPVDEDLENLKLKGKIDHYEKSQRSVVLYVDEFTNRDRKCLKLRARQEHVAENLQPAKVQVFDYYNPDSRCTV**

BGLB018444-RA **HNTLLQSFFWDNRKSKCEPCSTDCEEQTSEEEEEDDYEDFTFPPVQPRIQTLWKKKVKGLNLTVHEDENRSDFEDPNMVSKIGRPRRKRRSVRPYSASVICVEVCVRFLGNKTTGMSVVDVGLFTGYLPVDEDLENLKLKGKIDHYEKSQRSVVLYVDEFTNRDRKCLKLRARQEHVAENLQPAKVQVFDYYNPDSRCTV**

BGLB030758-RA

BGLB011300-RB

BGLB023169-RA

1610 1620 1630 1640 1650 1660 1670 1680 1690 1700 1710 1720 1730 1740 1750 1760 1770 1780 1790 1800

....|....|....|....|....|....|....|....|....|....|....|....|....|....|....|....|....|....|....|....|....|....|....|....|....|....|....|....|....|....|....|....|....|....|....|....|....|....|....|....|

BgC3-2 **FYKNNNNSGQLANFCDNQKQICQCLESRCAACEESWYGLGWMDMMKFACSNASYVLEIKALDRDLEKAGFERILGQIQAVHSQRGRHELKVGDKVILLKRASCFCPRVSPDQTYFMMLSQPKRFKDSDGNQIYAFLMDKKVLVIQNFKPRGLSREQKEISKNVNRTVKRLKRRGCSGGGKTPKVNGKNRKRRARRGKNGK**

BGLB018444-RA **FYKNNNNSGQLANFCDNQKQICQCLESRCAACEESWYGLGWMDMMKFACSNASYVLEIKALDRDLEKAGFERILGQIQVVHSQRGRHELKVGDKVILLKRASCFCPRVSPDQTYFMMLSQPKRFKDSDGNQIYAFLMDKKVLVIQNFKPRGLSREQKEISKNVNRTVKRLKRRGCSGGGKTPKVNGKNRKRRARRGKNGK**

BGLB030758-RA

BGLB011300-RB

BGLB023169-RA

....

BgC3-2 **TKKT**

BGLB018444-RA **TKKT**

BGLB030758-RA

BGLB011300-RB

BGLB023169-RA

**C) BgC3-3**

10 20 30 40 50 60 70 80 90 100 110 120 130 140 150 160 170 180 190 200

....|....|....|....|....|....|....|....|....|....|....|....|....|....|....|....|....|....|....|....|....|....|....|....|....|....|....|....|....|....|....|....|....|....|....|....|....|....|....|....|

BgC3-3 **MYVKRTMTVADGLSLILSVCLIQQSVGTKYFLTVPQVPSYDANVTAVVTAFQHTSQSTEKVLLQYIGGENSKNVLNSTHLSFDQDGSQQWTVTFSSESMQELRESSVVLQMTCNGQKKEILLTFRQSSGYIFIQTDKPIYTPGQTVKFRVIAVDEDQRLSKHHLKVDIINDQQVTVDRMRYSAEDAFKGQNFELPKDIAP**

BGLB030610-RA **MYVKRTMTVADGLSLILSVCLIQQSVGTKYFLTVPQLPSYDANVTAVVTAFQHTSQSTEKVLLQYIGGENSKNVLNSTHLSFDQDGSQQWTVTFSSESMQELRESSVVLQMTCNGQKKEILLTFRQSSGYIFIQTDKPIYTPGQTVKFRVIAVDEDQRLSKHHLKVDIINDQQVTVDRMRYSAEDAFKGQNFELPKDIAP**

BGLB020436-RA **--------------------------------------------------------------------------------------------------------------------------------------------------------------------------------------------------------**

210 220 230 240 250 260 270 280 290 300 310 320 330 340 350 360 370 380 390 400

....|....|....|....|....|....|....|....|....|....|....|....|....|....|....|....|....|....|....|....|....|....|....|....|....|....|....|....|....|....|....|....|....|....|....|....|....|....|....|....|

BgC3-3 **GRWYISANFEGLDSNYRLAHNVSIEVREYVLPRFSATLHANTSVITKDSKALKLTVTSKYVFGRPVHGNVEIHLGILDNNKLLPHAVLRGKLQNGQFSQDVDVNILTLSKLMYTSNQRLHVGVNVIEKGTFENYTLTDSSIFISHPYYIVDLKSSKEFFKPGFSYTLKAVIKTKVPLTVSHLDLYIFAEFLDANDNIIKS**

BGLB030610-RA **GRWYISANFEGLDSNYRLAHNVSIEVREYVLPRFSATLHANTSVITKDSKALKLTVTSKYVFGRPVHGNVEIHLGILDNNKFLPRAVLRGKLQNGQFSQDVDVNILTLSKLMYTSNQRLHVGVNVIEKGTFENYTLTDSSIFISHPYYIVDLKSSKEFFKPGFSYTLKAVIKTKVPLTVSHLDLYIFAEFLDANDNIIKS**

BGLB020436-RA **--------------------------------------------------------------------------------------------------------------------------------------------------------------------------------------------------------**

410 420 430 440 450 460 470 480 490 500 510 520 530 540 550 560 570 580 590 600

....|....|....|....|....|....|....|....|....|....|....|....|....|....|....|....|....|....|....|....|....|....|....|....|....|....|....|....|....|....|....|....|....|....|....|....|....|....|....|....|

BgC3-3 **VSENVPIMKNTIVTQDFITPKTAEKINFKVHVVDENHPSFEHFHFTVKKYISANHEYLHINMSKFQPVMKWSDGVFFLEYTKSAYLNSSSLITVNILSKGQVIYSINVKKNILGVSPVSLPKQLFGELSPAYRIVAYYYIAGAVPELVADSLLVDTELDTCVDEVYLIRDKFSQFSPVPKKPKDKLDLLIIGSPLMKIGL**

BGLB030610-RA **VSEKVPIMKNTVVTQDFITPETAEKINFKVHVVDENHPSFEHFHFTVKKYISANHEYLHINMSKFQPVMKWSDGVFFLEYTKSAYLNSSSLITVNILSKGQVIYSINVKKNILGVSPVSLPKQLFGELSPAYRIVAYYYIAGAVPELVADSLLVDTELDTCVDEVHLIRDKFSQYSPVPKKPKDKLDLLIIGSPLMKIGL**

BGLB020436-RA **--------------------------------------------------------------------------------------------------------------------------------------------------------------------------------------------------------**

610 620 630 640 650 660 670 680 690 700 710 720 730 740 750 760 770 780 790 800

....|....|....|....|....|....|....|....|....|....|....|....|....|....|....|....|....|....|....|....|....|....|....|....|....|....|....|....|....|....|....|....|....|....|....|....|....|....|....|....|

BgC3-3 **LAVDKAIFLLNDKQTLTRELLFHTLGSHDPSTSEGDGLNAELILENSGLYHMMVDTDAYSTSVTPRRALSSFGSFYDISFDRINMPEENRHGNQVSEKQPETEDSLPQRSDVRFYFPETWLFEEKIIPRDRKLPLELSLPDSITTWSFVAVGLSNNRGICVSIPLEQVVEKPVFLEVRMPFKASRLEELNINIIIHNYHT**

BGLB030610-RA **LALDKAIFLLNDKQTLTRELLFHTLRSHDPSTSEGDGLNAELILENSGLYHMMVDTDAYSTSVTPRRALSSFGSFYDISFDRINMPEENRHGNQVSEKQPETEDSLPQRSDVRFYFPETWLFEEKIIPRDRKLPLELSLPDSITTWSFVAVGLSNNRGICVSIPLEQVVEKPVFLEVRMPFKASRLEELNINIIIHNYHT**

BGLB020436-RA **--------------------------------------------------------------------------------------------------------------------------------------------------------------------------------------------------------**

810 820 830 840 850 860 870 880 890 900 910 920 930 940 950 960 970 980 990 1000

....|....|....|....|....|....|....|....|....|....|....|....|....|....|....|....|....|....|....|....|....|....|....|....|....|....|....|....|....|....|....|....|....|....|....|....|....|....|....|....|

BgC3-3 **NDVRPEVTIIGDSGLCFAENATRGGNHSDHGFNMTVTAGEMAERTVRIIPLKIGELTLKVSMISHLGNDTVEKKLRVIAEGLRVRKAITFVLDPGAKHTTFMNYSDNNIQQSNTATIQNRYIASRNMQHTTIDLALPPEVIKGTESCQISAFGDLMGDIITHAVVQSKGLMEEPTLIAQEVLNDLGPIVHALNYINDSGL**

BGLB030610-RA **NDVRPEVTIIGDSGLCFAENATRGGNHSDHGFNMTVTAGEMAERTVRIIPLKIGELTLKVSMISHLGNDTVEKKLRVIAEGLRVRKAITFVLDPGAKHTTFMNYSDNNIQQSNTATIQNRYIASRNMQHTTIDLALPPEVIKGTESCQISAFGDLMGDIITHAVVQSKGLMEEPTLIAQEVLNDLGPIVHALNYINDSGL**

BGLB020436-RA **--------------------------------------------------------------------------------------------------------------------------------------------------------------------------------------------------------**

1010 1020 1030 1040 1050 1060 1070 1080 1090 1100 1110 1120 1130 1140 1150 1160 1170 1180 1190 1200

....|....|....|....|....|....|....|....|....|....|....|....|....|....|....|....|....|....|....|....|....|....|....|....|....|....|....|....|....|....|....|....|....|....|....|....|....|....|....|....|

BgC3-3 **MTHDLKLRSHRFIRHGVVRLLTYKSGKAFSVRPGMKPATWLSALILKSLCHATSLAFIDKHNLIDTGFSWLQDQIKKDGSLNELDWTGRKNNAQYRIELAAEVLISVLECNRKEKEDHLTLQDKMADYLQKHIDKIKLPVVMAKTAYALMLYNSDSNKTLNAVDKLKRLALKGVQGHIYWANKPKDEDEKKPHWYIDRVP**

BGLB030610-RA **MTHDLKQRSHRFIRHGVVRLLTYKSGKAFSVRPGMKPATWLSALILKSLCHATSLAFIDKHNLIDTGFSWLQDQIKKDGSLNELDWTGRKNNAQYRIELAAEVLISVLECNRKEKEDHLTLQDKMADYLQKHINKIKLPVVMAKTAYALMLYNSDSNKTLNAVDKLKRLALKGAQGHIYWANKPKDEDEKKPHWYIDRVP**

BGLB020436-RA **--------------------------------------------------------------------------------------------------------------------------------------------------------------------------------------------------------**

1210 1220 1230 1240 1250 1260 1270 1280 1290 1300 1310 1320 1330 1340 1350 1360 1370 1380 1390 1400

....|....|....|....|....|....|....|....|....|....|....|....|....|....|....|....|....|....|....|....|....|....|....|....|....|....|....|....|....|....|....|....|....|....|....|....|....|....|....|....|

BgC3-3 **ESSIEATAYGLLVFLRKKNLLNVDAVADWLVAQRKHNGAFNGAKDSTAAIQALTEYSLQKHKEEEIKVNMNLTVRAGKAEKNQYKFKFTQENATQPESRSNVPVYQFLEVLTEGQGLGQMQINVEYNIPVDKNEDCSFNISVEVKTAKIALDSSNLLCSSCDFNCPGAKINYNIDDTIKDRTAISKTVSMLTSGRNPKTN**

BGLB030610-RA **ESSIEATAYGLLVFLRKKNLLNVDAVADWLVAQRKHNGAFNGAKDSTAAIQALTEYSLQKHKEEEIKVNMNLTVRAGKAEKNQYKFKFTQENATQPESRSNVPVYQFLEVLTEGQGLGQMQINVEYNIPVDKNEDCSFNISVEVKTAKIALDS--------------------------------------------RTN**

BGLB020436-RA **--------------------------------------------------------------------------------------------------------------------------------------------------------------------------------------------------------**

1410 1420 1430 1440 1450 1460 1470 1480 1490 1500 1510 1520 1530 1540 1550 1560 1570 1580 1590 1600

....|....|....|....|....|....|....|....|....|....|....|....|....|....|....|....|....|....|....|....|....|....|....|....|....|....|....|....|....|....|....|....|....|....|....|....|....|....|....|....|

BgC3-3 **AKPKTKKNKPKPTKQPRRRPQLSRKPKPHGKRSKRALSSGKSYCVTVCIRHLQGVSRPVDVRIQMLTGIRPLDEDVRKISQTIPNVIDARLTENAEFLIVKFSKVEATKNTCFAYRARVENDATRINGANIEIIQENSPKPSCVLEYHPPEDKESLKVYCADYNHINRGECKCFSGQCGKCGPMTSSEFDLDKTIKLTCK**

BGLB030610-RA **AKPKTKKNKPKPTKQPRRRPQLSRKPKPHGKRSKRALSSGKSYCVTVCIR**

BGLB020436-RA **----------------------------------------------------------------MLTGIRPLDEDVRKISQTIPNVIDARLTENAEFLIVKFSKVEATKNTCFAYRARVENDATRINGANIEIIQENSPKPSCVLEYHPPEDKESLKVYCADFNHINRGECKCFSGQCGKCGPMTSSEFDLDKTIKLTCK**

1610 1620 1630 1640 1650 1660 1670 1680 1690 1700 1710 1720 1730 1740 1750

....|....|....|....|....|....|....|....|....|....|....|....|....|....|....|....|....|....|....|....|....|....|....|....|....|....|....|....|....|....|....

BgC3-3 **ADLVYQLKLGSQEDKIHWLEINATVHSVNKTTGTHELKEGDEIIMMSPGYCMCFRDYFGKEEKFYLLSSDVDRLMDRQGTIVHRYVLDENTTLLRVSQPLSMAGHNNSKSEQTKEIFISQPLNKHEQDLSPHFKLAVSHEHLAAGLSQGDKCEL**

BGLB030610-RA

BGLB020436-RA **ADLVYQLKLGSQEDKIHWLEINATVHSVNKTTGTHELKEGDEIIMMSPGYCMCFRDYFGKEEKFYLLSSDVDRLMDRQGTIVHRYVLDENTTLLRVSQPLSMAGHNNSKSEQTREIFISQPLNKHEQDLSPHFKLAVSHEHLAAGLSQGDKCEL**

**D) BgA2M**

10 20 30 40 50 60 70 80 90 100 110 120 130 140 150 160 170 180 190 200

....|....|....|....|....|....|....|....|....|....|....|....|....|....|....|....|....|....|....|....|....|....|....|....|....|....|....|....|....|....|....|....|....|....|....|....|....|....|....|....|

BgA2M-C **MDVTSRSLTLFFITLASLCHAENNFLLTLPKAIYAGSKTEFCLTAYNDIKVTIDFISLRNVQDTPVLIKDSYSRGEQKCTNFQAPPQGEYRLEVTTQSTEPGAVSELHNSTKVTVHGSKLITFIQTDKPMYKPGQKVMFRVFTLMRNLKPRTENIKSIYVLDPNDVRVKQFLDVEQKGIGSFEFQLIEEAKLGPWKIEVY**

BgA2M-B **MDVTSRSLTLFFITLASLCHAENNFLLTLPKAIYAGSKTEFCLTAYNDIKVTIDFISLRNVQDTPVLIKDSYSRGEQKCTNFQAPPQGEYRLEVTTQSTEPGAVSELHNSTKVTVHGSKLITFIQTDKPMYKPGQKVMFRVFTLMRNLKPRTENIKSIYVLDPNDVRVKQFLDVEQKGIGSFEFQLIEEAKLGPWKIEVY**

BgA2M-A **MDVTSRSLTLFFITLASLCHAENNFLLTLPKAIYAGSKTEFCLTAYNDIKVTIDFISLRNVQDTPVLIKDSYSRGEQKCTNFQAPPQGEYRLEVTTQSTEPGAVSELHNSTKVTVHGSKLITFIQTDKPMYKPGQKVMFRVFTLMRNLKPRTENIKSIYVLDPNDVRVKQFLDVEQKGIGSFEFQLIEEAKLGPWKIEVY**

BGLB016521-RB **---------------------------------------------------------------------------------------------------------------------------------MYKPGQKVMFRVFTLMRNLKPRTENIKSIYVLDPNDVRVKQFLDVEQKGIGNFEFQLIEEAKLGPWKIEVY**

BGLB016521-RA **---------------------------------------------------------------------------------------------------------------------------------MYKPGQKVMFRVFTLMRNLKPRTENIKSIYVLDPNDVRVKQFLDVEQKGIGNFEFQLIEEAKLGPWKIEVY**

210 220 230 240 250 260 270 280 290 300 310 320 330 340 350 360 370 380 390 400

....|....|....|....|....|....|....|....|....|....|....|....|....|....|....|....|....|....|....|....|....|....|....|....|....|....|....|....|....|....|....|....|....|....|....|....|....|....|....|....|

BgA2M-C **LDDEDEVRQQATVQEFEVKEYVLPRFEVLITAPNNILITDKNIKGKVCAQYTYGKPVSGFVHIELKTSTNLYRYSPSDSQEQVKQISGCYEFSFDIPVEKHYTLYSYKLNVTVTEKGTGVVVNNVFDGPKITYEPLTIEIEDFTKGFFKPGLPYYGKVTVKKIDGSPAEGEKIIVSTQNEFLYAGEFITDSNGTFLFSLC**

BgA2M-B **LDDEDEVRQQATVQEFEVKEYVLPRFEVLITAPNNILITDKNIKGKVCAQYTYGKPVSGFVHIELKTSTNLYRYSPSDSQEQVKQISGCYEFSFDIPVEKHYTLYSYKLNVTVTEKGTGVVVNNVFDGPKITYEPLTIEIEDFTKGFFKPGLPYYGKVTVKKIDGSPAEGEKIIVSTQNEFLYAGEFITDSNGTFLFSLC**

BgA2M-A **LDDEDEVRQQATVQEFEVKEYVLPRFEVLITAPNNILITDKNIKGKVCAQYTYGKPVSGFVHIELKTSTNLYRYSPSDSQEQVKQISGCYEFSFDIPVEKHYTLYSYKLNVTVTEKGTGVVVNNVFDGPKITYEPLTIEIEDFTKGFFKPGLPYYGKVTVKKIDGSPAEGEKIIVSTQNEFLYAGEFITDSNGTFLFSLC**

BGLB016521-RB **LDDEDEVRQRATVQEFEVKEYVLPRFEVLITAPNNILITDKNIKGKVCAQYTYGKPVSGFVHIELKTSTNLYRYSPSDSQEQVKQISGCYEFSFDIPVEKHYTLYSYKLNVTVTEKGTGVVVNNVFDGPKITYEPLTIEIEDFTKGFFKPGLPYYGKVTVKKIDGSPAEGEKIIVSTQNEFLYAGEFITDSNGTFLFSLC**

BGLB016521-RA **LDDEDEVRQRATVQEFEVKEYVLPRFEVLITAPNNILITDKNIKGKVCAQYTYGKPVSGFVHIELKTSTNLYRYSPSDSQEQVKQISGCYEFSFDIPVEKHYTLYSYKLNVTVTEKGTGVVVNNVFDGPKITYEPLTIEIEDFTKGFFKPGLPYYGKVTVKKIDGSPAEGEKIIVSTQNEFLYAGEFITDSNGTFLFSLC**

410 420 430 440 450 460 470 480 490 500 510 520 530 540 550 560 570 580 590 600

....|....|....|....|....|....|....|....|....|....|....|....|....|....|....|....|....|....|....|....|....|....|....|....|....|....|....|....|....|....|....|....|....|....|....|....|....|....|....|....|

BgA2M-C **EGLTNNRSSVQISAEALGYNSSRYITRGFKTIQQWYSPSRSYVQIPPAEAQLKCSGKVSLTVPFTTKENSLVQFYYQVIARGNLVKSGHIIHSGESTYDDTAQSQSKCLRQLSEEERNETLGSIHYYRPSRGYYQGSHDDTFLKEVVTSDFQADKVSFFVLDLDVVPVMSPQFNVLVYHILPDGEVVADGRDFSVQPCFE**

BgA2M-B **EGLTNNRSSVQISAEALGYNSSRYITRGFKTIQQWYSPSRSYVQIPPAEAQLKCSGKVSLTVPFTTKENSLVQFYYQVIARGNLVKSGHIIHSGESTYDDTAQSQSKCLRQLSEEERNETLGSIHYYRPSRGYYQGSHDDTFLKEVVTSDFQADKVSFFVLDLDVVPVMSPQFNVLVYHILPDGEVVADGRDFSVQPCFE**

BgA2M-A **EGLTNNRSSVQISAEALGYNSSRYITRGFKTIQQWYSPSRSYVQIPPAEAQLKCSGKVSLTVPFTTKENSLVQFYYQVIARGNLVKSGHIIHSGESTYDDTAQSQSKCLRQLSEEERNETLGSIHYYRPSRGYYQGSHDDTFLKEVVTSDFQADKVSFFVLDLDVVPVMSPQFNVLVYHILPDGEVVADGRDFSVQPCFE**

BGLB016521-RB **EGLTNNRSSVQISAEALGYNSSRYITRGFKTIQQWYSPSRSYVQIPPAEAQLKCSGKVSLTVPFTTKENSLVQFYYQVIARGNLVKSGHIIHSGESTYDDTAQSQSKCLRQLSEEERNETLGSIHYYRPSRGYYQGSHDDTFLKEVVTSDFQADKVSFFVLDLDVVPVMSPQFNVLVYHILPDGEVVADGRDFSVQPCFE**

BGLB016521-RA **EGLTNNRSSVQISAEALGYNSSRYITRGFKTIQQWYSPSRSYVQIPPAEAQLKCSGKVSLTVPFTTKENSLVQFYYQVIARGNLVKSGHIIHSGESTYDDTAQSQSKCLRQLSEEERNETLGSIHYYRPSRGYYQGSHDDTFLKEVVTSDFQADKVSFFVLDLDVVPVMSPQFNVLVYHILPDGEVVADGRDFSVQPCFE**

610 620 630 640 650 660 670 680 690 700 710 720 730 740 750 760 770 780 790 800

....|....|....|....|....|....|....|....|....|....|....|....|....|....|....|....|....|....|....|....|....|....|....|....|....|....|....|....|....|....|....|....|....|....|....|....|....|....|....|....|

BgA2M-C **NQVEMTFSKTTVAPGEKVDVFIGAQPASICGLGVVDKSINLLGGNHQVTPEMVFKKIEEFNLVPPPGADEYFNNKDYQYCMKNVKSTSEGQDHEDYFWILSSPFVDALQAFEASGFTVVTDLKLETRPCSRRPQVFYAGKRQKKLQSNIKCPKGDLCSMFSKLAAAHNILIYLNAKSLVRGTMEEWEKAVREIFPETWLW**

BgA2M-B **NQVEMTFSKTTVAPGEKVDVFIGAQPASICGLGVVDKSINLLGGNHQVTPEMVFKKIEEFNLVPPPGADEYFNNKDYQYCMKNVKSTSEGQDHEDYFWILSSPFVDALQAFEASGFTVVTDLKLETRPCSRRPQVFYDFMVPASMSS------------AELLPPSNTLLVSSN------------SKAIRAHFPETWLW**

BgA2M-A **NQVEMTFSKTTVAPGEKVDVFIGAQPASICGLGVVDKSINLLGGNHQVTPEMVFKKIEEFNLVPPPGADEYFNNKDYQYCMKNVKSTSEGQDHEDYFWILSSPFVDALQAFEASGFTVVTDLKLETRPCSRRPQVFYA-------------------------------------ESLVRGTMEEWEKAVREIFPETWLW**

BGLB016521-RB **NQVEMTFSKTTVAPGEKVDVFIGAQPASICGLGVVDKSINLLGGNHQVTPEMVFKKIEEFNLVPPPGADEYFNNKDYQYCMKNVKSTSEGQDHEDYFWILSSPFVDALQAFEASGFTVVTDLKLETRPCSRRPQVFYDFMVPASMSS------------AELLPPSNTLLVSSN------------SKAIRAHFPETWLW**

BGLB016521-RA **NQVEMTFSKTTVAPGEKVDVFIGAQPASICGLGVVDKSINLLGGNHQVTPEMVFKKIEEFNLVPPPGADEYFNNKDYQYCMKNVKSTSEGQDHEDYFWILSSPFVDALQAFEASGFTVVTDLKLETRPCSRRPQVFYA-------------------------------------ESLVRGTMEEWEKAVREIFPETWLW**

810 820 830 840 850 860 870 880 890 900 910 920 930 940 950 960 970 980 990 1000

....|....|....|....|....|....|....|....|....|....|....|....|....|....|....|....|....|....|....|....|....|....|....|....|....|....|....|....|....|....|....|....|....|....|....|....|....|....|....|....|

BgA2M-C **DISVVGDSGAVTLHETAPDTITSWIGNVLCVHPETGFGASPVTSLRTFQPFFLSLQLPYAAVRGEKLPIMLTVYNYLEKCLHIKMALDMEKNFAVDKNELLKEPVCVCGGKSHTVKIYVTPKGLGYLPIIAKAEIIPGLCSNTIDVDTQYIGLSDAVKRQMFVKAEGIEQVNTNTMFVCSKVDSPKQEELVLSVPSDEEI**

BgA2M-B **DISVVGDSGAVTLHETAPDTITSWIGNVLCVHPETGFGASPVTSLRTFQPFFLSLQLPYAAVRGEKLPIMLTVYNYLEKCLHIKMALDMEKNFAVDKNELLKEPVCVCGGKSHTVKIYVTPKGLGYLPIIAKAEIIPGLCSNTIDVDTQYIGLSDAVKRQMFVKAEGIEQVNTNTMFVCSKVDSPKQEELVLSVPSDEEI**

BgA2M-A **DISVVGDSGAVTLHETAPDTITSWIGNVLCVHPETGFGASPVTSLRTFQPFFLSLQLPYAAVRGEKLPIMLTVYNYLEKCLHIKMALDMEKNFAVDKNELLKEPVCVCGGKSHTVKIYVTPKGLGYLPIIAKAEIIPGLCSNTIDVDTQYIGLSDAVKRQMFVKAEGIEQVNTNTMFVCSKVDSPKQEELVLSVPSDEEI**

BGLB016521-RB **DLHLVGDSGAVTLHETAPDTITSWIGNVLCVHPETGFGASPVTSLRTFQPFFLSLQLPYAAVRGEKLPIMLTVYNYLEKCLHIKMALDMEKNFAVDKNELLKEPVCVCGGKSHTVKIYVTPKGLGYLPIIAKAEIIPGLCSNTIDVDTQYIGLSDAVKRQMFVKAEGIEQVNTNTMFVCSKVDSPKQEELVLSVPSDEEI**

BGLB016521-RA **DISVVGDSGAVTLHETAPDTITSWIGNVLCVHPETGFGASPVTSLRTFQPFFLSLQLPYAAVRGEKLPIMLTVYNYLEKCLHIKMALDMEKNFAVDKNELLKEPVCVCGGKSHTVKIYVTPKGLGYLPIIAKAEIIPGLCSNTIDVDTQYIGLSDAVKRQMFVKAEGIEQVNTNTMFVCSKVDSPKQEELVLSVPSDEEI**

1010 1020 1030 1040 1050 1060 1070 1080 1090 1100 1110 1120 1130 1140 1150 1160 1170 1180 1190 1200

....|....|....|....|....|....|....|....|....|....|....|....|....|....|....|....|....|....|....|....|....|....|....|....|....|....|....|....|....|....|....|....|....|....|....|....|....|....|....|....|

BgA2M-C **VKDSIRGELKVIGDIMGPALTNLDRLVKLPTGCGEQNMVGFVPNIFALKYLTETRRITDEIKSKALKFMEVGYQRELTFRHIDGSYSAFGDKDPQGSIWLTAFVVKSYAQAQPYIYIDEKDLQVSLKYLHLNQLETGCYRETGRVLGSYMMGGLKGDNKEEESFTALTAYVVIALLTAGVNSSQPGIYGAMECINADFDS**

BgA2M-B **VKDSIRGELKVIGDIMGPALTNLDRLVKLPTGCGEQNMVGFVPNIFALKYLTETRRITDEIKSKALKFMEVGYQRELTFRHIDGSYSAFGDKDPQGSIWLTAFVVKSYAQAQPYIYIDEKDLQVSLKYLHLNQLETGCYRETGRVLGSYMMGGLKGDNKEEESFTALTAYVVIALLTAGVNSSQPGIYGAMECINADFDS**

BgA2M-A **VKDSIRGELKVIGDIMGPALTNLDRLVKLPTGCGEQNMVGFVPNIFALKYLTETRRITDEIKSKALKFMEVGYQRELTFRHIDGSYSAFGDKDPQGSIWLTAFVVKSYAQAQPYIYIDEKDLQVSLKYLHLNQLETGCYRETGRVLGSYMMGGLKGDNKEEESFTALTAYVVIALLTAGVNSSQPGIYGAMECINADFDS**

BGLB016521-RB **VKDSIRGELKVIGDIMGPALTNLDRLVKLPTGCGEQNMVGFVPNIFALKYLTETRRITDEIKSKALKFMEVGYQRELTFRHKDGSYSAFGDKDPQGSIWLTAFVVKSYAQAQPYIYIDEKDLQVSLKYLHLNQLETGCYRETGRVLGSYMMGGLKGDNKEEESFTALTAYVVIALLTAGVNSSQPGIYGAMECINADFDS**

BGLB016521-RA **VKDSIRGELKVIGDIMGPALTNLDRLVKLPTGCGEQNMVGFVPNIFALKYLTETRRITDEIKSKALKFMEVGYQRELTFRHKDGSYSAFGDKDPQGSIWLTAFVVKSYAQAQPYIYIDEKDLQVSLKYLHLNQLETGCYRETGRVLGSYMMGGLKGDNKEEESFTALTAYVVIALLTAGVNSSQPGIYGAMECINADFDS**

1210 1220 1230 1240 1250 1260 1270 1280 1290 1300 1310 1320 1330 1340 1350 1360 1370 1380 1390 1400

....|....|....|....|....|....|....|....|....|....|....|....|....|....|....|....|....|....|....|....|....|....|....|....|....|....|....|....|....|....|....|....|....|....|....|....|....|....|....|....|

BgA2M-C **LREQMDPYALALVAYANALYAPSSHRTSEIIAALEAVARTEGDFKYWARKDFQPKVSNSWYTYSMPSAEVEMTAYVLLTYIKLFGPRAVERTHNIAMWLSKQRSPYGGFSSTQDTVVGLNALSEYSRLAFNGGKTELKVSITGSKLKQTFSLSQKKKTTLLLHRASIPVLPNQISLISEGEGCALVQFSVFYNKLSKEFK**

BgA2M-B **LREQMDPYALALVAYANALYAPSSHRTSEIIAALEAVARTEGDFKYWARKDFQPKVSNSWYTYSMPSAEVEMTAYVLLTYIKLFGPRAVERTHNIAMWLSKQRSPYGGFSSTQDTVVGLNALSEYSRLAFNGGKTELKVSITGSKLKQTFSLSQKKKTTLLLHRASIPVLPNQISLISEGEGCALVQFSVFYNKLSKEFK**

BgA2M-A **LREQMDPYALALVAYANALYAPSSHRTSEIIAALEAVARTEGDFKYWARKDFQPKVSNSWYTYSMPSAEVEMTAYVLLTYIKLFGPRAVERTHNIAMWLSKQRSPYGGFSSTQDTVVGLNALSEYSRLAFNGGKTELKVSITGSKLKQTFSLSQKKKTTLLLHRASIPVLPNQISLISEGEGCALVQFSVFYNKLSKEFK**

BGLB016521-RB **LREQMDPYALALVAYANALYAPSSHRTSEIIAALEAVTRTEGDFKYWARKDFQPKVSNSWYTYSMPSAEVEMTAYVLLTYIKLFGPRAVERTHNIAMWLSKQRSPYGGFSSTQDTVVGLNALSEYSRLAFNGGKTELKVSITGSKLKQTFSLSQKKKTTLLLHRASIPVLPNQISLISEGEGCALVQFSVFYNKLSKEFK**

BGLB016521-RA **LREQMDPYALALVAYANALYAPSSHRTSEIIAALEAVTRTEGDFKYWARKDFQPKVSNSWYTYSMPSAEVEMTAYVLLTYIKLFGPRAVERTHNIAMWLSKQRSPYGGFSSTQDTVVGLNALSEYSRLAFNGGKTELKVSITGSKLKQTFSLSQKKKTTLLLHRASIPVLPNQISLISEGEGCALVQFSVFYNKLSKEFK**

1410 1420 1430 1440 1450 1460 1470 1480 1490 1500 1510 1520 1530 1540 1550 1560 1570 1580 1590 1600

....|....|....|....|....|....|....|....|....|....|....|....|....|....|....|....|....|....|....|....|....|....|....|....|....|....|....|....|....|....|....|....|....|....|....|....|....|....|....|....|

BgA2M-C **DKSSFHLEVNPSHYKPNKDKCDHRSIVISAGTKGKARETSGMVLIELKLVTGWTPLPESLTKIQLRFVDIKKIEYNENEGLIAFYFDQLSGKPIEFTLDVKQDLELGVSNPKPADVKVYYYYEKDVFKVQSYKIKTTCGTKEEIPHKNTDPEFGPEGPNQVRINPGIDAPFTMSSDGCPVCIPVSVLPLNFKDLICRSSA**

BgA2M-B **DKSSFHLEVNPSHYKPNKDKCDHRSIVISAGTKGKARETSGMVLIELKLVTGWTPLPESLTKIQLRFVDIKKIEYNENEGLIAFYFDQLSGKPIEFTLDVKQDLELGVSNPKPADVKVYYYYEKDVFKVQSYKIKTTCGTKEEIPHKNTDPEFGPEGPNQVRINPGIDAPFTMSSDGCPVCIPVSVLPLNFKDLICRSSA**

BgA2M-A **DKSSFHLEVNPSHYKPNKDKCDHRSIVISAGTKGKARETSGMVLIELKLVTGWTPLPESLTKIQLRFVDIKKIEYNENEGLIAFYFDQLSGKPIEFTLDVKQDLELGVSNPKPADVKVYYYYEKDVFKVQSYKIKTTCGTKEEIPHKNTDPEFGPEGPNQVRINPGIDAPFTMSSDGCPVCIPVSVLPLNFKDLICRSSA**

BGLB016521-RB **DKSSFHLEVNPSHYKPNKDKCDHRSIVISAGTKGKARETSGMVLIELKLVTGWTPLPESLTKIQLRFVDIKKIEYNENEGLIAFYFDQLSGKPIEFTLDVKQDLELGVSNPKPADVKVYYYYEKDVFKVQSYKIKTTCGTKEEIPHKNTDPEFGPEGPNQVRINPGIDAPFTMSSDGCPVCIPVSVLPLNFKDLICRSSA**

BGLB016521-RA **DKSSFHLEVNPSHYKPNKDKCDHRSIVISAGTKGKARETSGMVLIELKLVTGWTPLPESLTKIQLRFVDIKKIEYNENEGLIAFYFDQLSGKPIEFTLDVKQDLELGVSNPKPADVKVYYYYEKDVFKVQSYKIKTTCGTKEEIPHKNTDPEFGPEGPNQVRINPGIDAPFTMSSDGCPVCIPVSVLPLNFKDLICRSSA**

1610 1620 1630 1640 1650 1660 1670 1680 1690 1700 1710

....|....|....|....|....|....|....|....|....|....|....|....|....|....|....|....|....|....|....|....|....|....|...

BgA2M-C **VYKVAIMKGKTVKLLQDLRPPSLVKKINIVVELELPPGCTCGLLTNQGKKALLLVKKPITADSTLVKLDNTSVITLEDKKFTKTTRNTQKTCPLKKLEEKKKKHEKS------**

BgA2M-B **VYKVAIMKGKTVKLLQDLRPPSLVKKINIVVELELPPGCTCGLLTNQGKKALLLVKKPITADSTLVKLDNTSVITLEDKKFTKTTRNTQKTCPLKKLEEKKKKHEKS------**

BgA2M-A **VYKVAIMKGKTVKLLQDLRPPSLVKKINIVVELELPPGCTCGLLTNQGKKALLLVKKPITADSTLVKLDNTSVITLEDKKFTKTTRNTQKTCPLKKLEEKKKKHEKS------**

BGLB016521-RB **VYKVAIMKGKTVKLLQDLRPPSLVKKINIVVELELPPGCTCGLLTNQGKKALLLVKKPITADSTLVKLDNTSVITLDDKKFTKTTRNTQKTCPLKKLEEKKRSMRKVELSSNT**

BGLB016521-RA **VYKVAIMKGKTVKLLQDLRPPSLVKKINIVVELELPPGCTCGLLTNQGKKALLLVKKPITADSTLVKLDNTSVITLDDKKFTKTTRNTQKTCPLKKLEEKKRSMRKVELSSNT**

**E) BgMCR1**

10 20 30 40 50 60 70 80 90 100 110 120 130 140 150 160 170 180 190 200

....|....|....|....|....|....|....|....|....|....|....|....|....|....|....|....|....|....|....|....|....|....|....|....|....|....|....|....|....|....|....|....|....|....|....|....|....|....|....|....|

BgMCR1 **MWTSLAALFDEMRRKIEVQSRNMWHLICSTILIAMVSAQQPGGGPPREDNCLIDASVGCSYARAPQYLVITPKKIRPNQVFQIFATILKMEYNQEFVHVIVSIIKDNIEYANTALRFDRPSSRIMQLQMPSNAQEGKYRLRVEGRLNEQDTGNIWQNETDIDFTTKQASLFLQMSRPLYRQGQKVHFRIIPILPNMMPKY**

BGLB036531-RA **----------------------MWHLICSTILIAMVSAQQPGGGPPREDNCLIDASVGCSYARAPQYLVITPKKIRPNQVFQIFATILKMEYNQEFVHVIVSIIKDNIEYANTALRFDRPSSRIMQLQMPSNAQEGKYRLRVEGRLNEQDTGNIWQNETDIDFTTKQASLFLQMSRPLYRQGQKVHFRIIPILPNMMPKY**

BGLB035268-RA **--------------------------------------------------------------------------------------------------------------------------------------------------------------------------------------------------------**

210 220 230 240 250 260 270 280 290 300 310 320 330 340 350 360 370 380 390 400

....|....|....|....|....|....|....|....|....|....|....|....|....|....|....|....|....|....|....|....|....|....|....|....|....|....|....|....|....|....|....|....|....|....|....|....|....|....|....|....|

BgMCR1 **GSMVIYVDDPTGIPVRRWQSIQTNAGGIISQSFTLSDQPNFGTWIIRVEAFGHVYRHPFTVEDFWEPRFDVNVSVPSYVMETPEISVAGVMLANHTSGRPCIGNASITAFFRPREEIWNRTKGWEKPYWDAQAGGTPNSGVPMELPQYKPVHTIPVKDYYMYFAYEYRFIDYFQGRIDFEWTLEQLMTIAKRGGESGSLV**

BGLB036531-RA **GSMVIYVDDPTGIPVRRWQSIQTNAGGIISQSFTLSDQPNFGTWIIRVEAFGHVYRHPFTVEDFWEPRFDVNVSVPSYVMETPEISVAGVMLANHTSGRPCIGNASITAFFRPREEIWNRTKGWEKPYWDAQAGGTPNSGVPMELPQYKPVHTIPVKDYYMYFAYEYRFIDYFQGRIDFEWTLEQLMTIAKRGGESGSLV**

BGLB035268-RA **--------------------------------------------------------------------------------------------------------------------------------------------------------------------------------------------------------**

410 420 430 440 450 460 470 480 490 500 510 520 530 540 550 560 570 580 590 600

....|....|....|....|....|....|....|....|....|....|....|....|....|....|....|....|....|....|....|....|....|....|....|....|....|....|....|....|....|....|....|....|....|....|....|....|....|....|....|....|

BgMCR1 **DSEFVFFANVSDWYSGLNRTGWAGTIFFDSKIKLKWVGDQIRTFKPSQVMRVQVAVTKYDGTPVENVGTVTLTDVTTDSSGTAVQSKSNTQVPKNGIADFEYQLSATTQTLKLTATYADARKPYSGDNKIYLDPRFNLGTTVPITMYATRYYSPSNSYITILTSTDRPQVNEYMVFHVKTSNYVPRIYYQIVAQSNIIIG**

BGLB036531-RA **DSEFVFFANVSDWYSGLNRTGWAGTIFFDSKIKLKWVGDQIRTFKPSQVMRVQVAVTKYDGTPVENVGTVTLTDVTTDSSGTAVQSKSNTQVPKNGIADFEYQLSATTQTLKLTATYADARKPYSGDNKIYLDPRFNLGTTVPITMYATRYYSPSNSYITILTSTDRPQVNEYMVFHVKTSNYVPRIYYQIVAQSNIIIG**

BGLB035268-RA **--------------------------------------------------------------------------------------------------------------------------------------------------------------------------------------------------------**

610 620 630 640 650 660 670 680 690 700 710 720 730 740 750 760 770 780 790 800

....|....|....|....|....|....|....|....|....|....|....|....|....|....|....|....|....|....|....|....|....|....|....|....|....|....|....|....|....|....|....|....|....|....|....|....|....|....|....|....|

BgMCR1 **DWLEMTSRQKTFAVALSRDMVPTARLVVYYIRQPEEIVVDVLNFFVNGTRQNLVTLNINRGKDFSRDTIEFNAYADPGSYVSFSGMLLDLYSRGLSDGITENKLIDELMTYDSTQNGSYRHLWRVSDTEYEYVFYHGPDYGIDANTSFDTAGLLVLTDARVSRLYNDKFCTDQKSFPCFVGIESQCFKPEQRCDGTIDCV**

BGLB036531-RA **DWLEMTSRQKTFAVALSRDMVPTARLVVYYIRQPEEIVVDVLNFFVNGTRQNLVTLNINRGKDFSRDTIEFNAYADPGSYVSFSGMLLDLYSRGLSDGITENKLIDELMTYDSTQNGSYRHLWRVSDTEYEYVFYHGPDYGIDANTSFDTAGLLVLTDARVSRLYNDKFCTDQKSFPCFVGIESQCFKPEQRCDGTIDCV**

BGLB035268-RA **--------------------------------------------------------------------------------------------------------------------------------------------------------------------------------------------------------**

810 820 830 840 850 860 870 880 890 900 910 920 930 940 950 960 970 980 990 1000

....|....|....|....|....|....|....|....|....|....|....|....|....|....|....|....|....|....|....|....|....|....|....|....|....|....|....|....|....|....|....|....|....|....|....|....|....|....|....|....|

BgMCR1 **NDGADEWGCTFQESKEIHNPAMDRVSRVMRFYDNSSWAWQEIFVKPDGRVDFRVDVPKYPLSWVINGVSVSRELGLGIMLKPVRYDAARYMYMQVEHPKHIIRGEQVGVRVTVFNYWYDDDYLEVLITMHGGDGYSFVTVGEYGYVTSYTPPTHKGDHQTIVFLEPGESKDIYMPIVPDGGVVRGQIEFKVSASCFMQKD**

BGLB036531-RA **NDGADEWGCTFQESKEIHNPAMDRVSRVMRFYDNSSWAWQEIFVKPDGRVDFRVDVPKYPLSWVINGVSVSRELGLGIMLKPVRYDAARYMYMQVEHPKHIIRGEQVGVRVTVFNYWYDDDYLEVNCTFLFQIK**

BGLB035268-RA **--------------------------------------------------------------------------------------------------------------------------------------------------------------------------------------------------------**

1010 1020 1030 1040 1050 1060 1070 1080 1090 1100 1110 1120 1130 1140 1150 1160 1170 1180 1190 1200

....|....|....|....|....|....|....|....|....|....|....|....|....|....|....|....|....|....|....|....|....|....|....|....|....|....|....|....|....|....|....|....|....|....|....|....|....|....|....|....|

BgMCR1 **EYIGTMYVKPDGVMNYYHTPYLIDLIRFGSIQIPQFDVPVPEQFRKLEVRENLYIPQSPEAVVSLFGDVVTPGFFQDYLNAENILWRPYGGGEMIVFNFAYNLYSLKFMKYSQQLDDAQLSKSLQEMNIAFQRILSYMNATDGSFKMFRDDPKPSLWLSAFVVKIVKEATFGEWERDLFMPRELINKVVLYICSRQNETT**

BGLB036531-RA

BGLB035268-RA **-----------------------------------------------------------------------------------------------------------------------------------------MNATDGSFKMFRDDPKPSLWLSAFVVKIVKEATFGEWERDLFMPRELINKVVLYICSRQNETT**

1210 1220 1230 1240 1250 1260 1270 1280 1290 1300 1310 1320 1330 1340 1350 1360 1370 1380 1390 1400

....|....|....|....|....|....|....|....|....|....|....|....|....|....|....|....|....|....|....|....|....|....|....|....|....|....|....|....|....|....|....|....|....|....|....|....|....|....|....|....|

BgMCR1 **GAFEPDDIEATYDRKMTLLESLKGDKLHAHPIPLTAYVLIALSDLQNYVSEEAAACRDTAVRNAANYLFNIVPEIKEVFHMAITTYALSLTQKRSPFETLWKMKRNDSDFLYFSEETSYENPYDFLNNVRYLKPRQELMNDAHAVQATAYALMAHMNSNLGTKVEREMMMAWLNTMRNSIGGFAATQDTILAMEALLKFT**

BGLB036531-RA

BGLB035268-RA **GAFEPDDIEATYDRKMTLLESLKGDKLHAHPIPLTAYVLIALSDLQNYVSEEAAACRDTAVRNAANYLFNIVPEIKEVFHMAITTYALSLTQKRSPFETLWKMKRNDSDFLYFSEETSYENPYDFLNNVRYLKPRQELMNDAHAVQATAYALMAHMNSNLGTKVEREMMMAWLNTMRNSIGGFAATQDTILAMEALLKFT**

1410 1420 1430 1440 1450 1460 1470 1480 1490 1500 1510 1520 1530 1540 1550 1560 1570 1580 1590 1600

....|....|....|....|....|....|....|....|....|....|....|....|....|....|....|....|....|....|....|....|....|....|....|....|....|....|....|....|....|....|....|....|....|....|....|....|....|....|....|....|

BgMCR1 **QVDPHRNVFDLSTTVESTSSPSWAATFNLKKLDYIRLKTNSLPADKVWGFIVPNAQGTGRALLQLTTTVNVEYMWLQKKPMRPNNDPNEEPIKFFDLIVEDLRFSGRNDSIMEMTNCVSWLYTEKSLTSGLAVLEVDIPTGYVVMNDTLRDYVRSNRVPSLKRAEHYDRKVIFYFEYLDESKTCVYFRADRWFPVANATI**

BGLB036531-RA

BGLB035268-RA **QVDPHRNVFDLSTTVESTSSPSWAATFNLKKLDYIRLKTNSLPSDKVWGFIVPNAQGTGRALLQLTTTVNVEYMWLQKKPMRPNNDPNEEPIKFFDLIVEDLRFSGRNDSIMEMTNCVSWLYTEKSLTSGLAVLEVDIPTGYVVMNDTLRDYVRSNRVPSLKRAEHYDRKVIFYFEYLDESKTCVYFRADRWFPVANATI**

1610 1620 1630 1640 1650 1660 1670

....|....|....|....|....|....|....|....|....|....|....|....|....|....|....|

BgMCR1 **QHRMRVYDYYEPGMHNTTLYTTRNLFLLNICFVCGSYQCPYCPYFNSATIMSAGFSVVLLMAATYFVQRLLLRNR**

BGLB036531-RA

BGLB035268-RA **QHRMRVYDYYEPGMHNTTLYTTRNLFLLNICFVCGSYQCPYCPYFNSATIMSAGFSVVLLMAATYFVQRLLLRNR**

**F) BgMCR2**

10 20 30 40 50 60 70 80 90 100 110 120 130 140 150 160 170 180 190 200

....|....|....|....|....|....|....|....|....|....|....|....|....|....|....|....|....|....|....|....|....|....|....|....|....|....|....|....|....|....|....|....|....|....|....|....|....|....|....|....|

BgMCR2 **MFFVILILYFVSLAIGQLGHEHCLIENSSGCELGRPPIYMITAPRRIRAGQMFQVFATILRMEYHENAISVRVSIVESDKEYTSSILKFERPSSRLMQLQMPSNAEAGNYKLRVEGRLDELVSGNIFFNETEIEFTPKHASIFIQMSKPIYTQEQLVHFRIIPLQPDLMPKYGNLIIYIEDPSGVPVKRWPGLQTNAGGI**

BGLB026925-RA  **MITAPRRIRAGQMFQVFATILRMEYHENAISVRVSIVESDKEYTSSILKFERPSSRLMQLQMPSNAEAGNYKLRVEGRLDELVSGNIFFNETEIEFTPKHASIFIQMSKPIYRQEQLGILEKLIFICNSSHYFFGTDICLNSDLYGP**

BGLB025060-RA

BGLB032488-RA

BGLB022655-RA

210 220 230 240 250 260 270 280 290 300 310 320 330 340 350 360 370 380 390 400

....|....|....|....|....|....|....|....|....|....|....|....|....|....|....|....|....|....|....|....|....|....|....|....|....|....|....|....|....|....|....|....|....|....|....|....|....|....|....|....|

BgMCR2 **ISQSFQLSDQPNYGTWHILVDAFGFKYRRPFIVEEFWEPRFDVNVSVPAYVMDVSSLSIQGVVLINHTSGRSCVGNGSITSFFIPSEEIWNATKGWEKPYHDAMSRRLSGTPLDVPKYRSIDSIPVSDYSVYFAYEYRFIDYYKGRINFEWHRDDLLGLAKRGGATSDELFGNEFVFVANMTDWYSGLNRTGWAGTIVYD**

BGLB026925-RA

BGLB025060-RA  **MDVSSLSIQGVVLINHTSGRSCVGNGSITSFFIPSEEIWNATKGWEKPYHDAMSRRLSGTPLDVPKYRSIDSIPVSDYSVYFAYEYRFIDYYKGRINFEWHRDDLLGLAKRGGATSDELFGNEFVFVANMTDWYSGLNRTGWAGTIVYD**

BGLB032488-RA

BGLB022655-RA

410 420 430 440 450 460 470 480 490 500 510 520 530 540 550 560 570 580 590 600

....|....|....|....|....|....|....|....|....|....|....|....|....|....|....|....|....|....|....|....|....|....|....|....|....|....|....|....|....|....|....|....|....|....|....|....|....|....|....|....|

BgMCR2 **DELNLKWIGGNVRTFKPGSLFRVQVAVSYYDGRPVSGGSVTLVPTVGSQSVKTAMSEYSPQTNPVVNGIAHFQIPLNVYVTRLALTASYHDPGETLAMKTSVNPNFVTSQSRSIQMLCTKYYSPTNSYLSIYTSTFEPQINEYMIFHVTLSHFVPRIYYQVVAQSNIIIADELEMSTKQKTFSVALSREMVPTARVIVYY**

BGLB026925-RA

BGLB025060-RA **DELNLKWIGGNVRTFKPGSLFRVQVAVSYYDGRPVSGGSVTLVPTVGSQSVKTAMSEYSPQTNPVVNGIAHFQIPLNVYVTRLALTVSRAAFICNS**

BGLB032488-RA  **MIFHVTLSHFVPRIYYQVVAQSNIIIADELEMSTKQKTFSVALSREMVPTARVIVYY**

BGLB022655-RA

610 620 630 640 650 660 670 680 690 700 710 720 730 740 750 760 770 780 790 800

....|....|....|....|....|....|....|....|....|....|....|....|....|....|....|....|....|....|....|....|....|....|....|....|....|....|....|....|....|....|....|....|....|....|....|....|....|....|....|....|

BgMCR2 **IKEPEEIVSDVLSFFVNGTRQNQVSLYINRGKDFSRNTVEFNAYADPGSYVAFSAMLLDLYSRGMNDGITENKLIDELLSYDQPANSSFKHLWRVSDTEYQYTFFHGSDYGIDGNTTFKSAGIIIITDADVTRLPNQESCNPLDGKFPCFSGVETECFTSEQRCNGLFDGCPNDGADEWGCIFKDMEIDLKSPLQRISRV**

BGLB026925-RA

BGLB025060-RA

BGLB032488-RA **IKEPEEIVSDVLSFFVNGTRQNQVSLYINRGKDFSRNTVEFNAYADPGSYVAFSAMLLDLYSRGMNDGITENKLIDELLSYDQPANSSFKHLWRVSDTEYQYTFFHGSDYGIDGNTTFKSAGIIIITDADVTRLPNQESCNPLDGKFPCFSGVETECFTSEQCCNGLFDGCPNDGADEWGCKCTQFI**

BGLB022655-RA

810 820 830 840 850 860 870 880 890 900 910 920 930 940 950 960 970 980 990 1000

....|....|....|....|....|....|....|....|....|....|....|....|....|....|....|....|....|....|....|....|....|....|....|....|....|....|....|....|....|....|....|....|....|....|....|....|....|....|....|....|

BgMCR2 **MRYYDNSSWAWQEIFIKPDGRTDFRVDVPKYPLSWVINGISISQELGFGIMQQPLKFDASRFMYMQVEYPKYIVWGEQIGVRVTVFNNWYDDDYMEILVTMHEGQDIEFVSVGEMGYVTSYSPTTHKGDHQTIVFLEPGDSQDIYLPIVPAKSFRKDKLTFRVTAVSFMGKDEHIGEMIVKPNGVLNYYHTPYLIDLIRY**

BGLB026925-RA

BGLB025060-RA

BGLB032488-RA

BGLB022655-RA  **ILVTMHEGQDIEFVSVGEMGYVTSYSPTTHMGDHQTIVFLEPGDSQDIYLPIVPAKSFRKDKLTFRVTAVSFMGKDEHIGEMIVKPNGVLNYYHTPYLIDLIRY**

1010 1020 1030 1040 1050 1060 1070 1080 1090 1100 1110 1120 1130 1140 1150 1160 1170 1180 1190 1200

....|....|....|....|....|....|....|....|....|....|....|....|....|....|....|....|....|....|....|....|....|....|....|....|....|....|....|....|....|....|....|....|....|....|....|....|....|....|....|....|

BgMCR2 **PSIDLPQFKVNVPELFRKQEYRPNLYVPQSPKATINIFGDVVTPGFFESYLNAENLLYRPYGGGEMVTFNFAYNVLALVFMRNSNQLDAYQTKTVLNELNIALQRIYSYMNEDGSFKMFRDDDNSNLWLTAFVGKTLAVAGEEDYWELELYIAKEWAAKIVNFICSRQNTTTGAFEPLENEIAFDRKMASLRKMKSDVMI**

BGLB026925-RA

BGLB025060-RA

BGLB032488-RA

BGLB022655-RA **PSIDLPQFKVNVPELFRKQEYRPNLYVPQSPKATINIFGDVVTPGFFESYLNAENLLYRPYGGGEMVTFNFAYNVLALVFMRNSNQLDAYQTKTVLNELNIALQRIYSYMNEDGSFKMFRDDDNSNLWLTAFVGKTLAVAGEEDYWELELYIAKEWAAKIVNFICSRQNTTTGAFEPLENEIAFDRKMASLRKMKSDVMI**

1210 1220 1230 1240 1250 1260 1270 1280 1290 1300 1310 1320 1330 1340 1350 1360 1370 1380 1390 1400

....|....|....|....|....|....|....|....|....|....|....|....|....|....|....|....|....|....|....|....|....|....|....|....|....|....|....|....|....|....|....|....|....|....|....|....|....|....|....|....|

BgMCR2 **TQTVPLTAYILIALEKMSKFVEGTTCLNTAKRNAVKYLQSQVNSLSKDEIFYMAITAYALSLTSNAFDIVNELWKLKRNDSDFTYFADQLVYENPSAIQNNVRYLMPRQELLNDAYAVQTTAYALLAHITANKADKLERDMTMTWLNTMRNSFGGFSSTQDTIVAIEALMEYTRQDQKRNESDMSIDLQSMASPGWKNSA**

BGLB026925-RA

BGLB025060-RA

BGLB032488-RA

BGLB022655-RA **TQTVPLTAYILIALEKMSKFVEGTTCLNTAKRNAVKYLQSQVNSLSKDEIFYMAITAYALSLTSNAFDIVNELWKLKRNDSDFTYFADQLVYENPSAIQNNVRYLMPRQELLNDAYAVQTTAYALLAHITANKADKLERDMTMTWLNTMRNSFGGFSSTQDTIVAIEALMEYTRQDQKRNESDMSIDLQSMASPGWKNSV**

1410 1420 1430 1440 1450 1460 1470 1480 1490 1500 1510 1520 1530 1540 1550 1560 1570 1580 1590 1600

....|....|....|....|....|....|....|....|....|....|....|....|....|....|....|....|....|....|....|....|....|....|....|....|....|....|....|....|....|....|....|....|....|....|....|....|....|....|....|....|

BgMCR2 **YIVRNNFTQLYQIPLPLNEVFGYVIPSAKGVGRALLQLTVTSNVEYEELMKTQQHYNNNPQEDLIPFFDLQVEARWGGRNDSIMFMRSCISWLYTERSLTSGLAVLEVDMPTGYIVMNDTLRSYVQSRVVPNLKRAEFYARKTVFYFEYLDTSKTCVDFRADRWFPVANSTKEHRIRVYDYYEPGMHRTRLYTVQNLFLM**

BGLB026925-RA

BGLB025060-RA

BGLB032488-RA

BGLB022655-RA **YIVRNNFTQLYQIPLPLNEVFGYVIPSAKGVGRALLQLTVTSNVEYEELMKTQQHYNNNPQEDLIPFFDLQVEARWGGRNDSIMFMRSCISWLYTERSLTSGLAVLEVDMPTGYIVMNDTLRSYVQSRVVPNLKRAEFYARKTVFYFEYV**

1610 1620 1630 1640

....|....|....|....|....|....|....|....|...

BgMCR2 **NICFVCGSYQCPYCPYFNRSVLIIGSNWSLFVVVMYIIHLCFQ**

BGLB026925-RA

BGLB025060-RA

BGLB032488-RA

BGLB022655-RA

**G) BgTEP1**

10 20 30 40 50 60 70 80 90 100 110 120 130 140 150 160 170 180 190 200

....|....|....|....|....|....|....|....|....|....|....|....|....|....|....|....|....|....|....|....|....|....|....|....|....|....|....|....|....|....|....|....|....|....|....|....|....|....|....|....|

BgTEP1 **--MKLNLILFVFYLVFQECQGGKYFISAPRNVVPGTAYDISVDILKQDIDNVTVEAILQDYSFSIPEGPKSLLTANGTFSPGVRGTLSMPIDFNLHCSYCRILLKGYNPLQFEQDIFIQISSDILSILIQTDKAIYKPKERVNFRILAAYYNLQLYTGTFHYEILDPYDNKINVLSGVSGTFGVVEGFFDLSDQPSFGTW**

BGLB021162-RC **MRMKLNLILFVFYLVFQECQGGKYFISAPRNVVPGTAYDISVDILKQDIGNVTVEAILQDYSFSIPEGPKSLLTANGTFSPGVRGTLSMPIDFNLHCSYCRILLKGYNPLQFEQDIFIQISSDILSILIQTDKAIYKPKERVNFRILAAYYNLQLYTGTFHYEILDPYDNKINVLSGVSGTFGVVEGFFDLSDQPSFGTW**

BGLB021162-RA **MRMKLNLILFVFYLVFQECQGGKYFISAPRNVVPGTAYDISVDILKQDIGNVTVEAILQDYSFSIPEGPKSLLTANGTFSPGVRGTLSMPIDFNLHCSYCRILLKGYNPLQFEQDIFIQISSDILSILIQTDKAIYKPKERVNFRILAAYYNLQLYTGTFHYEILDPYDNKINVLSGVSGTFGVVEGFFDLSDQPSFGTW**

BGLB021162-RB **--MKLNLILFVFYLVFQECQGGKYFISAPRNVVPGTAYDISVDILKQDIGNVTVEAILQDYSFSIPEGPKSLLTANGTFSPGVRGTLSMPIDFNLHCSYCRILLKGYNPLQFEQDIFIQISSDILSILIQTDKAIYKPKERVNFRILAAYYNLQLYTGTFHYEILDPYDNKINVLSGVSGTFGVVEGFFDLSDQPSFGTW**

BGLB039358-RA **--------------------------------------------------------------------------------------------------------------------------------------------------------------------------------------------------------**

BGLB035738-RA **--------------------------------------------------------------------------------------------------------------------------------------------------------------------------------------------------------**

BGLB028355-RA **--------------------------------------------------------------------------------------------------------------------------------------------------------------------------------------------------------**

BGLB031097-RA **--------------------------------------------------------------------------------------------------------------------------------------------------------------------------------------------------------**

BGLB035158-RA **--------------------------------------------------------------------------------------------------------------------------------------------------------------------------------------------------------**

210 220 230 240 250 260 270 280 290 300 310 320 330 340 350 360 370 380 390 400

....|....|....|....|....|....|....|....|....|....|....|....|....|....|....|....|....|....|....|....|....|....|....|....|....|....|....|....|....|....|....|....|....|....|....|....|....|....|....|....|

BgTEP1 **KINVRTETVSGAESQFFEVAEYDLPRFQVDVGLPPFALLSDTTLSGSVEAKYTFGQPVYGLVLLQIGENVDTIDKCNVNRKVTEISFEIKGKGNFSVPLEDIRRSVHLNEKKKIKITAFVTEASTGIKLNGSSVITYYGNRYQIKFLEMTPAVFKPGLQYTAYVQVTTPDGLPPTDSNLSLSVYTSVTYQMTVPDQELYS**

BGLB021162-RC **KINVRTEVNISQRAYF**

BGLB021162-RA **KINVRTEVNISQRAYF**

BGLB021162-RB **KINVRTEVNISQRAYF**

BGLB039358-RA **-------TVSGEESQLFEVAEYDLPRFQVDVGLPPFALLSDTTLSGSVEAKYTFGQPVYGLVLLQIGENVDTIDKCNVNRKVTEISFEIKGKGNFSVPLEDIRRSVHLNEKKKIKITAFVTEASTGIKLNGSSVITYYGNRYQIKFLEMTPAVFKPGLQYTAYVSKTGILN**

BGLB035738-RA **----------------------------------------------------------------------------------------------------------------------------------------------------------------------------------------------MTVPDQELYS**

BGLB028355-RA **--------------------------------------------------------------------------------------------------------------------------------------------------------------------------------------------------------**

BGLB031097-RA **--------------------------------------------------------------------------------------------------------------------------------------------------------------------------------------------------------**

BGLB035158-RA **--------------------------------------------------------------------------------------------------------------------------------------------------------------------------------------------------------**

410 420 430 440 450 460 470 480 490 500 510 520 530 540 550 560 570 580 590 600

....|....|....|....|....|....|....|....|....|....|....|....|....|....|....|....|....|....|....|....|....|....|....|....|....|....|....|....|....|....|....|....|....|....|....|....|....|....|....|....|

BgTEP1 **PSSFSGSYPLPGQNMSLPANGILSIDIDIPLNATSIDIKVSLNKETTAEKRISKSYSMSNNYLQLSLLSKLVKAESDVLIKITSTEAIDSLAYEIRSRSDHVKSGVLELSGQREFNATFKVEPSWAPIAQLLMYYIRRDSNEVVTDSLAFNVEGMFKNKVNVAFKENETDINKNVSLELSADSDSQIYVLAVDQSVLLLK**

BGLB021162-RC

BGLB021162-RA

BGLB021162-RB

BGLB039358-RA

BGLB035738-RA **PSSFSGSYPLPGQNMSLPANGILSIDIDIPLNATSIDIKVSLNKQTTAEKRISKSYSMSNNYLQLSLLSKLVKAESDVLIKITSTEAIDSLAYEIRSRSDRVKSGVLELNGQREFNATFKVEPSWAPIAQLLMYYIRRDSNEVVTDSLAFNVEGMFKNKVSQLSIS**

BGLB028355-RA **------------------------------------------------------------------------------------------------------------------------------------MYYIRRDSNEIVTDSLAFNVEGMFENKVSVAFKENETDINKNVSLELSADSDSQVYVLAVDQSVLLLK**

BGLB031097-RA **--------------------------------------------------------------------------------------------------------------------------------------------------------------------------------------------------------**

BGLB035158-RA **--------------------------------------------------------------------------------------------------------------------------------------------------------------------------------------------------------**

610 620 630 640 650 660 670 680 690 700 710 720 730 740 750 760 770 780 790 800

....|....|....|....|....|....|....|....|....|....|....|....|....|....|....|....|....|....|....|....|....|....|....|....|....|....|....|....|....|....|....|....|....|....|....|....|....|....|....|....|

BgTEP1 **TGNDLTPNKVKDSFISKFHKGAIPTDSNFALSYSGSSINEVFSNMGLVIATDLNIFAPFRPIALGRFPSSGFDRQGMMGAPMAMSFRDDNAMESASFEMDVATSTKPVERVRSFFPESWLWTSVKSINGHATLTTTVPDTITSWIVSAFATNSDTGLGVAPTTSKLRVFRPFFVSLTYPRSVTRNEQFIVQATVFNYLPV**

BGLB021162-RC

BGLB021162-RA

BGLB021162-RB

BGLB039358-RA

BGLB035738-RA

BGLB028355-RA **TGNDLTPNKVKDSFISKFHKGEVPTDSNFALSYSGSSINEVFSNMGLAIATDLYIFAP---PMLISPKVGRLSSSNFESSPYANSYRADNAKQSTSFEMDVATSTKPVERVRSFFPESWLWTSVKSINGHATLTTTVPDTMTSWIVSAFATNPNTGLGVAPTTSKLRVFRPFFVSLTYPRSIIRNEQFIVQATVFNYLPV**

BGLB031097-RA **--------------------------------------------------------------------------------------------------------------------------------------------------------------------------------------------------------**

BGLB035158-RA **--------------------------------------------------------------------------------------------------------------------------------------------------------------------------------------------------------**

810 820 830 840 850 860 870 880 890 900 910 920 930 940 950 960 970 980 990 1000

....|....|....|....|....|....|....|....|....|....|....|....|....|....|....|....|....|....|....|....|....|....|....|....|....|....|....|....|....|....|....|....|....|....|....|....|....|....|....|....|

BgTEP1 **DLMVTVSLKENPFLTPITPGPGNQASNIQVRANEQGIVYFSLSALTVGSLDIEVSARSNMAADAIVRQILIKHEGAPVVYNNPILINLSNNQSTFEKNIAFTLPDSLVPESQRIRVKVTGDLIGSTVQSLTSLLTLPTGCGEQSLVKFTPNIHIGRYLKATNQLSEELNKKIIDLLNDGYQRQLTYKRYDNGFSAFGNYD**

BGLB021162-RC

BGLB021162-RA

BGLB021162-RB

BGLB039358-RA

BGLB035738-RA

BGLB028355-RA **DVMVTVSLKENPFLTPVTPGPGNQTSNIQVTFRHTLF**

BGLB031097-RA **--------------------------------------------------------------------------------------------------------------------------------------------------------------------------------------------------------**

BGLB035158-RA **--------------------------------------------------------------------------------------------------------------------------------------------------------------------------------------------------------**

1010 1020 1030 1040 1050 1060 1070 1080 1090 1100 1110 1120 1130 1140 1150 1160 1170 1180 1190 1200

....|....|....|....|....|....|....|....|....|....|....|....|....|....|....|....|....|....|....|....|....|....|....|....|....|....|....|....|....|....|....|....|....|....|....|....|....|....|....|....|

BgTEP1 **ISSSTWLTALVVTSFAEAQEFIFVDKEIILKASMLLIDRQNIDGSFNEFGKVLDRNTQGTTAGPALTAFVLVALLKAKELADVQDCKNNNKCRYYLLGNATLNATRNLERLMLADSIDDQFSLAVASYAFAEAKSQLAQSTFEKLLTFVKQEGGLEYWSANSTVNNEELNRFINWRPPRLQARPIDILITSYAILTYSSL**

BGLB021162-RC

BGLB021162-RA

BGLB021162-RB

BGLB039358-RA

BGLB035738-RA

BGLB028355-RA

BGLB031097-RA **---------------------------------------------------------------------------------------------------------------------------------MVTTRRDESGTNFIRPYVIINSSGGLEYWSANSTVNNEELNRFINWRPPRLQARPIDILITSYAILTYSSL**

BGLB035158-RA **--------------------------------------------------------------------------------------------------------------------------------------------------------------------------------------------------------**

1210 1220 1230 1240 1250 1260 1270 1280 1290 1300 1310 1320 1330 1340 1350 1360 1370 1380 1390 1400

....|....|....|....|....|....|....|....|....|....|....|....|....|....|....|....|....|....|....|....|....|....|....|....|....|....|....|....|....|....|....|....|....|....|....|....|....|....|....|....|

BgTEP1 **GRLDEALPSVRWLTLQKNAQGGFVSTQDTVVGLQALSSYGSKSFRPDTNITIYVSDMNTHLTMNVNSENALSLQIQEIQSNSQDFSITASGSGLALLDIEYSFNVLKELSKPVFDVNTVLLDDKLDSFNIMVCTKFLMKHDTGMVVQELSIPSGFVPDLSTLGQVAGVKRSERKGSIVAIYFDKISGSSLCYSIVMTREA**

BGLB021162-RC

BGLB021162-RA

BGLB021162-RB

BGLB039358-RA

BGLB035738-RA

BGLB028355-RA

BGLB031097-RA **GRLDEALPSVRWLTLQKNAQGGFVSTQDTVVGLQALSTYGSKSFRPDTNITIYVSDMNTHLTMNVKSDNALSLQIQEIQSNSQDFSITASGSGLALLDIEYSFNVLKELSKPVFDVNTVLLDDKLDSFNIMVCTKFLLKHDTGMVVQEVSIPSGFVPDLSTLGQVAGVKRSERKGSIVAIYFDKISGSSLCYSIVMTREA**

BGLB035158-RA **----------------------------------------------------------------------------------------------------------------------------------MVCTKFLLKHDTGMVVQELSIPSGFVPDLSTLGQVAGVKRSERKGSIVAIYFDKISGSSLCYSIVMTREA**

1410 1420 1430 1440

....|....|....|....|....|....|....|....|....|

BgTEP1 **KVAKSQKSYVRTYDYYEPANQATVFYQPRTLRDSTVCDVCLNCCP**

BGLB021162-RC

BGLB021162-RA

BGLB021162-RB

BGLB039358-RA

BGLB035738-RA

BGLB028355-RA

BGLB031097-RA **KVAKSQKSYVRTYDYYEPANQATVFYQPRTLRDSTVCDVCLNCCP**

BGLB035158-RA **KVAKSQKSYVRTYDYYEPANQATVFYQPRTLRDSTVCDVCPNCCP**

**H) BgTEP2**

10 20 30 40 50 60 70 80 90 100 110 120 130 140 150 160 170 180 190 200

....|....|....|....|....|....|....|....|....|....|....|....|....|....|....|....|....|....|....|....|....|....|....|....|....|....|....|....|....|....|....|....|....|....|....|....|....|....|....|....|

BgTEP2 **MWKLILLAVVIATASATNSYVVIAPSKVRANMDLSLSVNILNATGDVTVVASLLREQTTVVSATKVFQEGSPGTLNMKLPADLPSSTYTLNVKGSGGLTFDKSENLNYNNKETSVFIQLNKAIFKPGDTVNFRVFGVYSDLKSYTDPIDISIYDANSNKIKQWLKVTPTNGVITQELTLSTQPVLGDWKISVDAGRTKEE**

BGLB030043-RB **MWRLILLAVVVVTSSARNSYVVIAPSKVRANMDLTLSVNILNATGDVTVVASLLRGQITVVSATKVFQEGSPATLNMKLPADLPSSTYTLNVKGSGGLTFDKSENLNYNNKEASVFIQINKAIFKPGDIVNFRVFGVYSDLKSYTDPIDISIYDADSNKIKQWLQVTPTNGVIALELTLSTQPVLGDWNISVEAGRTKEE**

BGLB030043-RC **MWRLILLAVVVVTSSARNSYVVIAPSKVRANMDLTLSVNILNATGDVTVVASLLRGQITVVSATKVFQEGSPATLNMKLPADLPSSTYTLNVKGSGGLTFDKSENLNYNNKEASVFIQINKAIFKPGDIVNFRVFGVYSDLKSYTDPIDISIYDADSNKIKQWLQVTPTNGVIALELTLSTQPVLGDWNISVEAGRTKEE**

BGLB030043-RA **MWRLILLAVVVVTSSARNSYVVIAPSKVRANMDLTLSVNILNATGDVTVVASLLRGQITVVSATKVFQEGSPATLNMKLPADLPSSTYTLNVKGSGGLTFDKSENLNYNNKEASVFIQINKAIFKPGDIVNFRVFGVYSDLKSYTDPIDISIYDADSNKIKQWLQVTPTNGVIALELTLSTQPVLGDWNISVEAGRTKEE**

BGLB021854-RA **-------------------------------------------------------------------MESLHFLFYSQLPADLPSSTYTLNVKGSGGLTFDKSENLNYNNKETSVFIQLNKAIFKPGDTVNFRVFGVYSDLKSYTDPIDISIYDANSNKIKQWLKVTPTNGVITQELTLSTQPVLGDWKISVDAGRTKEE**

BGLB003612-RB **--------------------------------------------------------------------------------------------------------------------------------------------------------------------------------------------------------**

BGLB036035-RA **--------------------------------------------------------------------------------------------------------------------------------------------------------------------------------------------------------**

210 220 230 240 250 260 270 280 290 300 310 320 330 340 350 360 370 380 390 400

....|....|....|....|....|....|....|....|....|....|....|....|....|....|....|....|....|....|....|....|....|....|....|....|....|....|....|....|....|....|....|....|....|....|....|....|....|....|....|....|

BgTEP2 **KVFTVAEYVLPKFEVDVVMPSYALTTDNDVTVTVKSKYTYGKPVNGTADVLVKLHESFNTFDYSRALPVTTLQVPLNGEAKVTIPMSQVKAINPYLNQHVLIVIANVTESLTGNQMSGNGTVTLYDKGVKLDFPESNPKTFKPALQYIAYLKVTQPDGLPMTSTAEQVKVSIRVTAELPGTTPTPYYWYVPPTESRDLPA**

BGLB030043-RB **KVFTVAEYVLPKFEVDVVMPTYALTTDDDVTVTVKSKYTYGKPVNGTADVLVKLQNEFIKSMDSKYLQVINVKLPLNGEAKVIIPVFQVKVINPYLNQHVLIVIANVTESLTGNQMSGNGTVTLYDKGVKLEFPESNPKTFKPGLQYIVYLKISQPDDLPIPATNEKVEINIRVESDYLSDFLSDY----------STSV**

BGLB030043-RC **KVFTVAEYVLPKFEVDVVMPTYALTTDDDVTVTVKSKYTYGKPVNGTADVLVKLQNEFIKSMDSKYLQVINVKLPLNGEAKVIIPVFQVKVINPYLNQHVLIVIANVTESLTGNQMSGNGTVTLYDKGVKLEFPESNPKTFKPGLQYIVYLKISQPDDLPIPATNEKVEINIRVESDYLSDFLSDY----------STSV**

BGLB030043-RA **KVFTVAEYVLPKFEVDVVMPTYALTTDDDVTVTVKSKYTYGKPVNGTADVLVKLQNEFIKSMDSKYLQVINVKLPLNGEAKVIIPVFQVKVINPYLNQHVLIVIANVTESLTGNQMSGNGTVTLYDKGVKLEFPESNPKTFKPGLQYIVYLKISQPDDLPIPATNEKVEINIRVESDYLSDFLSDY----------STSV**

BGLB021854-RA **KVFTVAEYVLPKFEVDVVTPSYALTTDNDVTVTVKSKYTYGKPVNGTADVLVKLHESFNTFDYSRALPVTTLQVPLNGEAKVIIPMSQVKAISANLNQHVLIVIANVTESLTGNQMSGNGTVTLYDKGVKLDFPASNPKTFKPALQYIAYLKVTQPDGLPMTSTAEQVKVSIRVTAELPGTTPTPYYWYVPPTESRDLPA**

BGLB003612-RB **--------------------------------------------------------------------------------------------------------------------------------------------------------------------------------------------------------**

BGLB036035-RA **--------------------------------------------------------------------------------------------------------------------------------------------------------------------------------------------------------**

410 420 430 440 450 460 470 480 490 500 510 520 530 540 550 560 570 580 590 600

....|....|....|....|....|....|....|....|....|....|....|....|....|....|....|....|....|....|....|....|....|....|....|....|....|....|....|....|....|....|....|....|....|....|....|....|....|....|....|....|

BgTEP2 **LSLAIPDNGLVAIPVDVPADAKDVHVTANFQGVSKELTLGKSHSPSNSYIQLILKSGSVIKAGDSISFEVKGTQALTKLVYQILSRGGIVKTGTVDANGQLVYQFSIPSDSSMAPNARIVLYYVRADGEIVTDSISFDISGAFKNKVSIDLDKTDVEPGDDVTVTVKADPDSTAYCLAIDQSVLLLKGGNDVTDNDVYTE**

BGLB030043-RB **LSLAIPENGLLTIPVDVPDYAANVYVTATFRGVSEELTLEKSHSPSSSYIQVRLKSGLIIKAGESISYEIKGTRPLATLVYQVSMWGAFV--------------------------------------------------------------------------------------------------------------**

BGLB030043-RC **LSLAIPENGLLTIPVDVPDYAANVYVTATFRGVSEELTLEKSHSPSSSYIQVRLKSGLIIKAGESISYEIKGTRPLATLVYQVSMWGAFV--------------------------------------------------------------------------------------------------------------**

BGLB030043-RA **LSLAIPENGLLTIPVDVPDYAANVYVTATFRGVSEELTLEKSHSPSSSYIQVRLKSGLIIKAGESISYEIKGTRPLATLVYQVSMWGAFV--------------------------------------------------------------------------------------------------------------**

BGLB021854-RA **LSLAIPDNGLVAIPVDVPADAKDVHVTANFQGVSKELTLGKSHSPSNSYIQLILKSGSVIK-------------------FQILSRGGIVKTGTVDANGQLVYQFSIPSDSSMAPNARIVLYYVRADGEIVTDSISFDISGAFKNKVSIDLDKTDVEPGDDVTVTVKADPDSTAYCLAIDQSVLLLKGGNDVTDNDVYTE**

BGLB003612-RB **--------------------------------------------------------------------------------------------------------------------------------------------------------------------------------------------------------**

BGLB036035-RA **--------------------------------------------------------------------------------------------------------------------------------------------------------------------------------------------------------**

610 620 630 640 650 660 670 680 690 700 710 720 730 740 750 760 770 780 790 800

....|....|....|....|....|....|....|....|....|....|....|....|....|....|....|....|....|....|....|....|....|....|....|....|....|....|....|....|....|....|....|....|....|....|....|....|....|....|....|....|

BgTEP2 **LKEYDTITESSSNKGIIDCPMCKRRKRMIWWPFPTYYGGSDAQQIFSNAGVVVLTDATVYHYQEPIHLFNIPNFFQCGRSLSGQLRKRCFASFSPVMNLVSSVVSDPITLPVTETETKTEDLQQPTKTRSNFVETWLWNSLDIGANGSASITATVPDTITSWVASAFAINSASGLGVAPTQAHLRVFRPFFVSLNLPYSV**

BGLB030043-RB **--------------------------------------------------------------------------------------------------------------------------------------------------------------------------------------------------------**

BGLB030043-RC **--------------------------------------------------------------------------------------------------------------------------------------------------------------------------------------------------------**

BGLB030043-RA **--------------------------------------------------------------------------------------------------------------------------------------------------------------------------------------------------------**

BGLB021854-RA **LKEYDTITESGSNKGIIDCPMCKRRKRMIWWPFPTYYGGSDAQQIFSNAGVVVLTDATVYHYQEPIHLFNIPNFFQCGRSLNGQLRKRCFASFSPVMNLVSSVVSDPITLPVTETETKTEDLQQPTKTRSNFVETWLWNSLDIGANGSASITATVPDTITSWVASAFAINSASGLGVAPTQAHLRVFRPFFVSLNLPYSV**

BGLB003612-RB **--------------------------------------------------------------------------------------------------------------------------------------------------------------------------------------------------------**

BGLB036035-RA **--------------------------------------------------------------------------------------------------------------------------------------------------------------------------------------------------------**

810 820 830 840 850 860 870 880 890 900 910 920 930 940 950 960 970 980 990 1000

....|....|....|....|....|....|....|....|....|....|....|....|....|....|....|....|....|....|....|....|....|....|....|....|....|....|....|....|....|....|....|....|....|....|....|....|....|....|....|....|

BgTEP2 **TRGEHLALQANVFNYMTEDMQVRVTLAKSDNFFNIEIDANGAEVLKQVESVQDVMIKAGEAKSVYFPIVPADLGKIDIEVKAQSTKAADAVRRQLLVEAEGVPKIYNVPVLIDLTEGKTSFSKTVDLTLPSNTVKGSELARISAVGDLMGPTIAGLDSLLQMPTGCGEQTMIGLAPDVYVTDYLKSVNQLSGDIQTKALS**

BGLB030043-RB **--------------------------------------------------------------------------------------------------------------------------------------------------------------------------------------------------------**

BGLB030043-RC **--------------------------------------------------------------------------------------------------------------------------------------------------------------------------------------------------------**

BGLB030043-RA **--------------------------------------------------------------------------------------------------------------------------------------------------------------------------------------------------------**

BGLB021854-RA **TRGEHLALQANVFNYMTEDMQVRVTLAKSDNFFNIEIDANGAEVLKQVESVQDVMIKAGEAKSVYFPIVPADLGKIDIEVKAQSTKAADAVRRQLLVEAEGVPKIYNVPVLIDLTEGKTSFSKTVDLTLPSNTVKGSELARISAVGDLMGPTIAGLDSLLQMPTGCGEQTMIGLAPDVYVTDYLKSVNQLSGDIQTKALS**

BGLB003612-RB **----------------------------------------------------------------------------------------------------------------------------------------------------MGPTIAGLDSLLQMPTGCGEQTMVNLAPNVYVTDYLKSVNQLSADIRTKALN**

BGLB036035-RA **----------------------------------------------------------------------------------------------------------------------------------------------------MGPTIAGLDSLLQMPTGCGEQTMIGLAPDVYVTDYLKSVNQLSGDIQTKALS**

1010 1020 1030 1040 1050 1060 1070 1080 1090 1100 1110 1120 1130 1140 1150 1160 1170 1180 1190 1200

....|....|....|....|....|....|....|....|....|....|....|....|....|....|....|....|....|....|....|....|....|....|....|....|....|....|....|....|....|....|....|....|....|....|....|....|....|....|....|....|

BgTEP2 **YMESGYQRELTYKHTDGSFSAFGNSDASGSMWLTAFVTRVFKQAKAHIYIDDEVLIKAIQWMVSKQNANGSFPEPGNVIHKNMQGQAGSGVGLTLFVLISLLENKDLLVNTNAAGVLVDEARQKALVYSEQEVAKTDDLYILNMAAYAFQLANSSQVQTVLNKLEQKATVKDGRKYWHQPEQPKTTNTWDYPNPTKAVDI**

BGLB030043-RB **--------------------------------------------------------------------------------------------------------------------------------------------------------------------------------------------------------**

BGLB030043-RC **--------------------------------------------------------------------------------------------------------------------------------------------------------------------------------------------------------**

BGLB030043-RA **--------------------------------------------------------------------------------------------------------------------------------------------------------------------------------------------------------**

BGLB021854-RA **YMESGYQRELTYKHTDGSFSAFGNSDASGSMWLTAFVTRVFKQAKAHIYIDDEVLIKAIQWMVSKQNANGSFPEPGNVIHKNMQGQAGSGVGLTLFVLISLLENKDLLVNTNAAGILVDEARQKALVYAEQEVAKTDDLYILNMAAYAFQLANSSQVQTVLSKLEQKATVKDGRKYWHQPEQPKTTNTWDYPNPTKAVDI**

BGLB003612-RB **FMESGYQRELTYKHTDGSFSAFGNRDASGSMWLTAFVTRVFKQAKTHIYIDDEILIKAVQWMVSKQNDDGSFPEPGEVLNKNMQGQAGSGVGLSLYVLISLFENKDVLANTL----LVDEARQKALAYVEQEVAKTDDLYILTMAAYAFQLAYSSQVQTVLNKLEQKATVKDGRKHWHQPDQPKTTNTWDYPNPTKAVDI**

BGLB036035-RA **YMESGYQRELTYKHTDSSFSAFGNSDASGSMWLTAFVTRVFKQAKAHIYIDDEVLIKAIQWMVSKQNANGSFPEPGNVIHKNMQGQAGSGVGLTLFVLISLLENKDLLVNTNAAGVLVDEARQKALAYAEQEVAKTDDLYILNMAAYAFQLANSTQVQTVLNKLEQKATVKGSYSLFTIVVNLMNTNLMSTELLNR**

1210 1220 1230 1240 1250 1260 1270 1280 1290 1300 1310 1320 1330 1340 1350 1360 1370 1380 1390 1400

....|....|....|....|....|....|....|....|....|....|....|....|....|....|....|....|....|....|....|....|....|....|....|....|....|....|....|....|....|....|....|....|....|....|....|....|....|....|....|....|

BgTEP2 **EMTSYALLTYAARGNIVAGKSIMQWLTEQRNSNGGFSSTQDTVLALNALSEFAKQTYSNNFNVQITTQLNATTSYTFNIDKTNSLLLQSRETPDVPSQVKIDATGSGMALVQVAVSFNVESEIFETTFDLTVKLIEETINNLFVETCAKWLGSGPSSAMAVQEIGIPSGFEADLESIPQLDILKRIETQNKKVILYFDQI**

BGLB030043-RB **--------------------------------------------------------------------------------------------------------------------------------------------------------------------------------------------------------**

BGLB030043-RC **--------------------------------------------------------------------------------------------------------------------------------------------------------------------------------------------------------**

BGLB030043-RA **--------------------------------------------------------------------------------------------------------------------------------------------------------------------------------------------------------**

BGLB021854-RA **EMTSYALLTYAARGNIVAGKSIMQWLTEQRNSNGGFSSTQDTVLALNALSEFAKQTYSNNFNVQITTQLNATTSYTFNIDKTNSLLLQSRETPNVPSQVKIDATGSGMALVQVAVSFNVESEIFETTFDLTVKLIEETINNLFVETCAKWLGSGPSSAMAVQEIGIPSGFEADIESIPQLDILKRIETQNKKVILYFDQI**

BGLB003612-RB **EMTSYALLTYAARGNIVAGKSIMQWLTEQRNSNGGFSSTQDTVVALNALSEFAKQTYSNNFNVQITTQLNAT-SYTFNIDKTNSLLLQSRETPDVPSQVKIDATGSGMALVQVAVSFNVESEIFETTFDLTVKLIEETINHLFVETCAK**

BGLB036035-RA

1410 1420 1430 1440 1450 1460

....|....|....|....|....|....|....|....|....|....|....|....|..

BgTEP2 **GTTPVCLNFRAVRTGLVAKSQPAAIRVYDYYEPRNQVTAFYQSQILKDST-CVVCKEECATV**

BGLB030043-RB **------------------------------------------------------------**

BGLB030043-RC **------------------------------------------------------------**

BGLB030043-RA **------------------------------------------------------------**

BGLB021854-RA **GTTPVCLNFRAVRTGLVAKSQPAAIRVYDYYEPRNQVTAFYQSQILKDSTVCDVCKECDNCV**

BGLB003612-RB

BGLB036035-RA

**I) BgTEP3**

10 20 30 40 50 60 70 80 90 100 110 120 130 140 150 160 170 180 190 200

....|....|....|....|....|....|....|....|....|....|....|....|....|....|....|....|....|....|....|....|....|....|....|....|....|....|....|....|....|....|....|....|....|....|....|....|....|....|....|....|

BgTEP3 **---------MWKLILIAVVITSGRAQNIPGRQQAASNSTQRKSTWRDCTYMIIAPSKVRANMDLSLSVHILNASSNVMLLVTLSQGQKTVVSANKVFRQGAPEIFKLKLPAD-------------LPNSIYTLKVKGSGALTFNQSTDLSYNSKEAFSVFIQINKAIFKPDDTVNFRVFGIYPDLKSYSGPMDVSIY---**

BGLB000023-RB **MDSYCKLCRMWKLILIAVVTTSGRAQNIPDRQQAASNSTQRKSTWRDCTYMIIAPSKVRINMDLSLSVHILNASSNVMLLVTLSQGQKTVVSANKVFRQGAPEIFKIKLPAD-------------LPNSIYTLKVKGSGALTFNQSTDLSYNSKEAFSTFIQINKAIFKPDDTVNFRVFGIYPDLKSYSGPMDVSIY---**

BGLB000023-RA **--------------------------------------------------MIIAPSKVRINMDLSLSVHILNASSNVMLLVTLSQGQKTVVSANKVFRQ-APSFPKDDEPKGRQNLDNLAPALTILSVCIYTLKVKGSGALTFNQSTDLSYNSKEAFSTFIQINKAIFKPDDTVNFRVFGIYPDLKSYSGPMDVSIYVVV**

BGLB028857-RA **--------------------------------------------------------------------------------------------------------------------------------------------------------------------------------------------------------**

210 220 230 240 250 260 270 280 290 300 310 320 330 340 350 360 370 380 390 400

....|....|....|....|....|....|....|....|....|....|....|....|....|....|....|....|....|....|....|....|....|....|....|....|....|....|....|....|....|....|....|....|....|....|....|....|....|....|....|....|

BgTEP3 **-------------------------------------------------------DANSNKIKQWNKVNPVNGVFTQKLVLSAQPVLGDWKIEAKANTTRAQKIFTVAKYVLPKFKVEIVMPSFALTSDNDITVTIKSRYTYDKPVKGTADVLVKLNQFENSMPIDFAQSLPVTSLQMSINGEAKVTVPKDLINAKDKDV**

BGLB000023-RB **-------------------------------------------------------DANSNKIKQWNKVNPVNGVFTQKLVLSAQPVLGDWKIEANFPCSNEQKLFTVAKYVLPKFKVEIVMPSFALTSDNDITVTIKSRYTYDKPVKGTADVLVKLYQFENSMPIDFAQSLPVTSLQMSINGEAKVTVPKDLINAKDKDV**

BGLB000023-RA **LMPKQMPAEYGSIIGIKAKLPSGESNQDSFAWFTPNVDSHSQPPIGRKRKLCLNLDANSNKIKQWNKVNPVNGVFTQKLVLSAQPVLGDWKIEAKAN------------VILPKFKVEIVMPSFALTSDNDITVTIKSRYTYDKPVKGTADVLVKLYQFENSMPIDFAQSLPVTSLQMSV--------------------**

BGLB028857-RA **--------------------------------------------------------------------------------------------------------------------------------------------------------------------------------------------------------**

410 420 430 440 450 460 470 480 490 500 510 520 530 540 550 560 570 580 590 600

....|....|....|....|....|....|....|....|....|....|....|....|....|....|....|....|....|....|....|....|....|....|....|....|....|....|....|....|....|....|....|....|....|....|....|....|....|....|....|....|

BgTEP3 **LIVIANVTESLTGNKMTAKNTVTFYDQGAQLEYPKINPTSFKPGLKYSAYLQITQPDGLPTTSITEPVRISWKVEKEKKPIDSIRVRLPKNGLVSFSVNVPLNASSLIISATFQGVTKELIVEPSYSPSSSYMQLALKTASVIRAGDVVFFEVTSTTPMTQLVYQVLSKGVIVKVGSENATSKFSHQFSVVSDSSMAPSA**

BGLB000023-RB **LIVIANVTESLTGNKMTAKNTVTFYDQGAQLEYPEINPKSFKPGLKYSAYLQITQPDGLPTTSITEPVRISWKVEKEKKPIDSIRVRLPKNGLVSFSVNVPLNASSLIISATFQGVTKELTVEPSYSPSSSYMQLALKTASVIRAGDVVFFEVTSTTPMTQLVYQVLSKGVIVKVGSENATSKFSHQFSVVSDSSMAPSA**

BGLB000023-RA **----------------------------------------WKN-LNYQASLLYIR---------------------------------------KFILKVIL--------------------------------------------------------------------------------------------------**

BGLB028857-RA **--------------------------------------------------------------------------------------------------------------------------------------------------------------------------------------------------------**

610 620 630 640 650 660 670 680 690 700 710 720 730 740 750 760 770 780 790 800

....|....|....|....|....|....|....|....|....|....|....|....|....|....|....|....|....|....|....|....|....|....|....|....|....|....|....|....|....|....|....|....|....|....|....|....|....|....|....|....|

BgTEP3 **RMVIYFYRRDGEIVIDSISFDVSGAFKNKVSFGFNSKSVEPGNNVTVTVRADPNSAAYLLAIDQSVLLIRGDNDVTSDDVFTDLKKYDTAADSADCSTCKSGLWPSWTLGGAVALEIFTKAGVVALTDAVIIQSKPSDENKDGLIRNPGAIMV---PKQIRQVFSETFLWSDLTIGVNGSASITATVPDTITSWVASAFA**

BGLB000023-RB **RIVIYFYRRDGEIVIDSISFDVSGAFKNKVSFGFNSKSVEPGNNVTVTVRADPNSAAYLLAIDQSVLLIRGDNDVSSDDVFTDLKKYDTAADSADCSTCKSGLWPSWTLGGAVALEIFTKAGVVALTDAVIIQSKPSDENEDGIMQNPGAIMVPMVPKQIRQVFTETFLWSDLTIGVNGSASITATVPDTITSWVASAFA**

BGLB000023-RA **--------------------------------------------------------------------------------------------------------------------------------------------------------------------------------------------------------**

BGLB028857-RA **--------------------------------------------------------------------------------------------------------------------------------------------------------------------------------------------------------**

810 820 830 840 850 860 870 880 890 900 910 920 930 940 950 960 970 980 990 1000

....|....|....|....|....|....|....|....|....|....|....|....|....|....|....|....|....|....|....|....|....|....|....|....|....|....|....|....|....|....|....|....|....|....|....|....|....|....|....|....|

BgTEP3 **VNSESGLGIAPSQSYLRVFRPFFVNLNLPYSVIRGEHLVVQANVFNYMTEDMQVTVTLAASDKFYNIEIDANGANGLFQQSQSVKVIMVEAGEAISVYFPIFPHELGKIDIEVKAQSTKAADAVRRQLLVEAEGIPKTKNYPILIDLTEGRTSFSQTLNLPLPSNTVKDSQRTRFSVVGDLMGPTIAGLDALLQMPTGSG**

BGLB000023-RB **VNSESGLGIAPSQSYLRVFRPFFVSLNLPYSVIRGEHLVVQANVFNYMTEDMQVTVTLAASDKFYNIEIDANGANGLFQQSQSVKVIMVEAGEAISVYFPIFPHELGKTDIEVKAQSTSAADAVRRQLLVEAEGIPKTKNYPILIDLTEGRTSFSQTLNLPLPSNTVKDSQRTRFSVVGDLMGPTIAGLDALLQMPTGSG**

BGLB000023-RA **--------------------------------------------------------------------------------------------------------------------------------------------------------------------------------------------------------**

BGLB028857-RA **--------------------------------------------------------------------------------------------------------------------------------------------------------------------------------------------------------**

1010 1020 1030 1040 1050 1060 1070 1080 1090 1100 1110 1120 1130 1140 1150 1160 1170 1180 1190 1200

....|....|....|....|....|....|....|....|....|....|....|....|....|....|....|....|....|....|....|....|....|....|....|....|....|....|....|....|....|....|....|....|....|....|....|....|....|....|....|....|

BgTEP3 **EQNMVNLAPNIYVVNYLQSVNQLSTDIKSKASNFMEKGYQRELMYRHPDGSFSNFGSNDTSGSIWLTAFVVKIFHQAQGHIYIDDNVLIEALQWIVTQQNPDGSFQLPSKGQAGSSVVLTLHVLISLFENEDVLAEDNIVEARGKALTFVESEVDKTNDLYVLSLAAYTFQLAGSTRVQAVLDKLELRATVKGGRKFWLL**

BGLB000023-RB **EQNMVNLAPNIYVVNYLQSVNQLSTDIKSKALNFMEKGYQRELMYRHPDGSFSNFGSNDTSDNIWLTAFVVKIFHQAQGHIYIDDNVLIEALQWIVTQQNPDGSFQLPSKGQAGSSVVLTLHVLISLFENEDVLAEHVSY------------------------------------------------------------**

BGLB000023-RA **--------------------------------------------------------------------------------------------------------------------------------------------------------------------------------------------------------**

BGLB028857-RA **--------------------------------------------------------------------------------------------------------------------------------------------------------------------------------------------------------**

1210 1220 1230 1240 1250 1260 1270 1280 1290 1300 1310 1320 1330 1340 1350 1360 1370 1380 1390 1400

....|....|....|....|....|....|....|....|....|....|....|....|....|....|....|....|....|....|....|....|....|....|....|....|....|....|....|....|....|....|....|....|....|....|....|....|....|....|....|....|

BgTEP3 **PEQPKKKTILNYPNQTKSIDIEITSYVLLTYAARGNLVAGKSIMWWLAEQRNSQGGFPTARCSIIALNALAVFAEKTYRNNFNMKITAKVAPQKMLQYRIDRTNALILQSGEVSDVPAQVQIEATGSGLVLAEIAVSFNVESEIFRTTFDLKVTLVEESMNYFILQTCTKWIGSESDGVMTVQEIGIPTGFEADLDSIPN**

BGLB000023-RB **--------------------------------------------------------------------------------------------------------------------------------------------------------------------------------------------------------**

BGLB000023-RA **--------------------------------------------------------------------------------------------------------------------------------------------------------------------------------------------------------**

BGLB028857-RA **-------------------------------------------MWWLAEQRNSQGGFPTARCSIIALNALAVFAEKTYRNNFNMKITAKVAPQKMLQYRIDRTNALILQSGEVSDVPAQVQIEATGSGLVLAQIAVSFNVESEIFRTTFDLKVTLVEESMNYFILQTCTK**

1410 1420 1430 1440 1450 1460 1470 1480

....|....|....|....|....|....|....|....|....|....|....|....|....|....|....|....|...

BgTEP3 **LENLKRIESQFKNLYLYIDQIDSTPVCLTMKAVRIGVVSGLQPSTVRVIDYYEPSNQVTAFYQSQILEASIICDVCKECDNCF**

BGLB000023-RB **-----------------------------------------------------------------------------------**

BGLB000023-RA **-----------------------------------------------------------------------------------**

BGLB028857-RA

**J) BgTEP4**

10 20 30 40 50 60 70 80 90 100 110 120 130 140 150 160 170 180 190 200

....|....|....|....|....|....|....|....|....|....|....|....|....|....|....|....|....|....|....|....|....|....|....|....|....|....|....|....|....|....|....|....|....|....|....|....|....|....|....|....|

BgTEP4 **MNQIWLAAAFLAAAIVHFPAQCQLVPIQDESTTPLPLKNATYWMTVSSTVRQGQPLEFRGQILVGSDPVSVTVTLLNGEGTKTLKTSPAITLSPGAVQSFKVDVPENIMDLSGEEYLYQIKVQMVGKGKTVNFKEEVLLTYESKSFFTFIQTDKAMYKPGQTVKFRVLSMTPDLKVIRDNSNDIIIEDSNKNKIRQWQGV**

BGLB000155-RB **MNQIWLAAAFLAAAIVHFPAQCQLVPIQDESTTPLPLKNATYWMTVSSTVRQGQPLEFRGQILVGSDPVSVTVTLLNGEGTKTLKTSPAITLSPGAVQSFKVDVPENIMDLTGEEYLYQIKVQMVGEGKTVNFKEEVLLTYESKSFSTFIQTDKAMYKPGQTVKFRVLSMTPDLKVIRDNSNDIIIEVSRLLSDSPKWHW**

BGLB000155-RA **MNQIWLAAAFLAAAIVHFPAQCQLVPIQDESTTPLPLKNATYWMTVSSTVRQGQPLEFRGQILVGSDPVSVTVTLLNGEGTKTLKTSPAITLSPGAVQSFKVDVPENIMDLTGEEYLYQIKVQMVGEGKTVNFKEEVLLTYESKSFSTFIQTDKAMYKPGQTGRA**

BGLB036709-RA  **MPPILRKYMLNLIHKSHLGIVKCKQRAREVMFWPGMNSDIEVTVRDCSRCAEHQNQNVSEPLMPTKKPDLPYSMVGCDLFYFEGKRV**

BGLB001162-RA

BGLB001162-RB

BGLB032760-RA

210 220 230 240 250 260 270 280 290 300 310 320 330 340 350 360 370 380 390 400

....|....|....|....|....|....|....|....|....|....|....|....|....|....|....|....|....|....|....|....|....|....|....|....|....|....|....|....|....|....|....|....|....|....|....|....|....|....|....|....|

BgTEP4 **KDPNGRGVMELSLKIAKQVVFGDWTITVKTKGTETLKTFTVQEYKLPKYEVMITTPPFGIISDPVLPITVKAIYTFGQPVSKGTVDVVITLVYSLKPEIKISGLLNKDGEFTLQVSSKQLLGLVSNGQTDLNYQSFKINANVTETDTGRNEGSSVTIIYYKTPLQLTFLGISPNNFKPGLGYTAYLEVKKKDDTLFTLAE**

BGLB000155-RB **KCSVIFFDLHG**

BGLB000155-RA

BGLB036709-RA **KDPNGRGVIELSLQLAKQVVFGDWTITVKTKGTETSKTFTVQEYKLPKYEVMITSPPFGIISDPVLPITVKAIYTFGQPVSKGTVDVVITLVYSLKPEIKISGLLNKDGEFTLQVSSKQLLGLVSNGQRDLNYQSFKINANVTETDTGRNEGSSVTIIYYKTPLQLTFLGISPNNFKPGLGYTAYLEVKKKDDTLFTLAE**

BGLB001162-RA

BGLB001162-RB

BGLB032760-RA

410 420 430 440 450 460 470 480 490 500 510 520 530 540 550 560 570 580 590 600

....|....|....|....|....|....|....|....|....|....|....|....|....|....|....|....|....|....|....|....|....|....|....|....|....|....|....|....|....|....|....|....|....|....|....|....|....|....|....|....|

BgTEP4 **ASQIRLLINVTYTVQLNKEEMAQREKELNISKSNIDNTSGTDEKQLLIRPGFIPYYDKTKTLILTINDPIRTVPDNGLIPINLDIPMEAESVSIEVNGLEPFAAEKAYKSVSKMKSPTGTYLQLKVPTETPKVGSTIKVTAVATEVITKLNFQVYSKGQLLLSEIINNPQSNTKSVEYSFTISQAMAPTLTIIAFFMKAE**

BGLB000155-RB

BGLB000155-RA

BGLB036709-RA **ASQIRLLINVTYTVQLSKEEMAQLEKELNISKSNIDNTSGTDEKQLLIRPGFIPYYDKTKTLILTINDPIRTVPDNGLIPINLDIPMEAESVSIEVNGLEPFAVEKAYKSVSKMKSPTGTYLQLKVPTETPKVGSTIKVTAVATEVITKLNFQVS**

BGLB001162-RA

BGLB001162-RB

BGLB032760-RA

610 620 630 640 650 660 670 680 690 700 710 720 730 740 750 760 770 780 790 800

....|....|....|....|....|....|....|....|....|....|....|....|....|....|....|....|....|....|....|....|....|....|....|....|....|....|....|....|....|....|....|....|....|....|....|....|....|....|....|....|

BgTEP4 **NSEFVVDSLSIGVDGLFQKPITVEFSKTQVKPGEKVDVTIKAESDSIVYLLGVDKSVQLLKSGNDITQAMVQEELMGYGTSGDYGMWRFMFFCGWPSYFGGTDAKSILSSAGVHIITDGLVYKSAFDNSFATDSGDLEMQKQPEPDEARFSGSNLVLTNVVKRRKYFSETFLWAMEIINADSNGQVTLSVTAPDTITTWV**

BGLB000155-RB

BGLB000155-RA

BGLB036709-RA

BGLB001162-RA  **MAVQTSAYVGAIQVADVVRKYFPETWLWNSDVADSNGQVTLSVTAPDTITTWV**

BGLB001162-RB  **MGYGTSGDYGMWRFMFFCGWPSYFGGTDAKSIFSSAGVHIITDGLVYKSAFDNSFATDSGDLEMQKQPEPDEARFSGSNLVLTNVVKRRKYFSETFLWAMEIINADSNGQVTLSVTAPDTITTWV**

BGLB032760-RA

810 820 830 840 850 860 870 880 890 900 910 920 930 940 950 960 970 980 990 1000

....|....|....|....|....|....|....|....|....|....|....|....|....|....|....|....|....|....|....|....|....|....|....|....|....|....|....|....|....|....|....|....|....|....|....|....|....|....|....|....|

BgTEP4 **VTAFSAHPVYGLSIVKESANLTTFRDLFVSLDLPISIIRNENFCFVATVFCYNKEEIPVLLTLDKSDNFSNIHVKVENGQVILSKESLHYSHFLGYLAERDISSVKFCFMPTALGDIPLRVSALTNVPGLSDAMEQIITVKPEGAARSTSNSYLIDMATGRWEMNVTVKFPAATVTGSETIIFNTAGNLLGPMFDNLDDL**

BGLB000155-RB

BGLB000155-RA

BGLB036709-RA

BGLB001162-RA **VTAFSAHPVYGLSIVKESANLTTFRDLFVSLDLPISIIRNENFCFVATVFCYNKEEIPVLLTLDKSDNFSNIHIKVENGQVILSKESLHYSHFLGYLAERDISSVKFCFMPTALGDIPLRVSALTNVPGLSDAMEQIITVKPEGAARSTSNSYLIDMATGRWEMNVTVKFPAATVTGSETIIFNTAGNLLGPMFDNLDDL**

BGLB001162-RB **VTAFSAHPVYGLSIVKESANLTTFRDLFVSLDLPISIIRNENFCFVATVFCYNKEEIPVLLTLDKSDNFSNIHIKVENGQVILSKESLHYSHFLGYLAERDISSVKFCFMPTALGDIPLRVSALTNVPGLSDAMEQIITVKPEGAARSTSNSYLIDMATGRWEMNVTVKFPAATVTGSETIIFNTAGNLLGPMFDNLDDL**

BGLB032760-RA

1010 1020 1030 1040 1050 1060 1070 1080 1090 1100 1110 1120 1130 1140 1150 1160 1170 1180 1190 1200

....|....|....|....|....|....|....|....|....|....|....|....|....|....|....|....|....|....|....|....|....|....|....|....|....|....|....|....|....|....|....|....|....|....|....|....|....|....|....|....|

BgTEP4 **LKKPYGCGEQNMLNFAPNIFLLEFLFSTNKNRSVAMEKAKDNMLIGYQKEITYEHSNTGGFSAFGHHEGSKDSASSWLTSFVVKCFAIAFQLDAAQGNVITIEKEIIQRSVRFMISQQNLNGSFTEKGKVFHKEMQGGSAEGEALTAYTVIALYEAQKVFASGDSIVANISQSIKLGVDFLVRRLPFLTDPYDICIVTYT**

BGLB000155-RB

BGLB000155-RA

BGLB036709-RA

BGLB001162-RA **LKKPYGCGEQNMLNFAPNIFLLEFLFSTNKNRSDAMEKAKDNMLIGYQKEITYEHSNTGGFSAFGHHEGSNDSASSWLTSFVVKCFAIAFQLDAAQGNVITIEKEIIQRSVRFMISQQNLNGSFTEKGKVFHKEMQGGSAEGEALTAYTVIALYEAQKVFASGDSIVANISQSIKLGVDFLVRRLPFLTDPYDICIVTYT**

BGLB001162-RB **LKKPYGCGEQNMLNFAPNIFLLEFLFSTNKNRSDAMEKAKDNMLIGYQKEITYEHSNTGGFSAFGHHEGSNDSASSWLTSFVVKCFAIAFQLDAAQGNVITIEKEIIQRSVRFMISQQNLNGSFTEKGKVFHKEMQGGSAEGEALTAYTVIALYEAQKVFASGDSIVANISQSIKLGVDFLVRRLPFLTDPYDICIVTYT**

BGLB032760-RA

1210 1220 1230 1240 1250 1260 1270 1280 1290 1300 1310 1320 1330 1340 1350 1360 1370 1380 1390 1400

....|....|....|....|....|....|....|....|....|....|....|....|....|....|....|....|....|....|....|....|....|....|....|....|....|....|....|....|....|....|....|....|....|....|....|....|....|....|....|....|

BgTEP4 **LHLVNDNNKETAFNKMQSIAITGDGLRYWKRATPAESNIAKYEWTASADSISIEMTSYALLVYAFREIANTEGLPIVRWITNHRGPNGGFISTQDTVIGLQALARVAAKIYSNEDIPITLAVSYESKGQLVKEIIKINKSNEMLLQSVDINYKDEQPNFVNIVATTDSGKTGPSTVIAEIVLGYNILAETSAKFYDMSHT**

BGLB000155-RB

BGLB000155-RA

BGLB036709-RA

BGLB001162-RA **LHLVNDNNKETAFNKMQSIAITGDGLRYWKRATPAESNIAKYEWTASADSISIEMTSYALLVYAFREIANTEGLPIVRWITNHRGPNGGFISTQDTVIGLQALARVAAKIYSNEDIPITLAVSFESKGQLVKEIIKINKSNEMLLQSVDINYKWKEADIYPYW**

BGLB001162-RB **LHLVNDNNKETAFNKMQSIAITGDGLRYWKRATPAESNIAKYEWTASADSISIEMTSYALLVYAFREIANTEGLPIVRWITNHRGPNGGFISTQDTVIGLQALARVAAKIYSNEDIPITLAVSFESKGQLVKEIIKINKSNEMLLQSVDINYKVSKHIFVQQLVTGICLCNNSHQNCV**

BGLB032760-RA

1410 1420 1430 1440 1450 1460 1470 1480 1490 1500 1510 1520 1530

....|....|....|....|....|....|....|....|....|....|....|....|....|....|....|....|....|....|....|....|....|....|....|....|....|....|

BgTEP4 **LDKLSAGFVLTILIKTTKDSSSMCILEVDIPPGFTPDSDALKLNKAISLSEILGDVLAIYFNTDMITTKETPVKIFMVSTGGVLTKSQPRMYRVYDYYTPDRELSKNYLLEDTDFCTAAPDVGGCQYRQK**

BGLB000155-RB

BGLB000155-RA

BGLB036709-RA

BGLB001162-RA

BGLB001162-RB

BGLB032760-RA  **MCILEVDIPPGFTPDTDALKLNKAISLSEILGDVLAIYFNTDMITTKETPVKIFMVSTGGVLTKSQPRMYRVYDYYTPDRELSKNYLLEDTDFCTAAPDVGGCQYRQK**

**K) BgCD109**

10 20 30 40 50 60 70 80 90 100 110 120 130 140 150 160 170 180 190 200

....|....|....|....|....|....|....|....|....|....|....|....|....|....|....|....|....|....|....|....|....|....|....|....|....|....|....|....|....|....|....|....|....|....|....|....|....|....|....|....|

BgCD109 **MSWTSTSALCLLCAYSTLWITCYGSFMVLTPKSVYPGIPLGVSVTAHKVVTAPVSVALSLETVQHERSIGNAETILLPGETKLLTIQVPLLNYTSPFLQLKVSATGGFRDSQTKMISINQNTSLILVQTDKAIYKPGQKVRIRVVNVDRYLKPVFNPLTVIIENAKNDKLEEYKDVNSKNGNYTYGKGVQGQCELTVHYT**

BGLB021085-RA **MSWTSTSALCLLCAYSTLWITCYGSFMVLTPKSVYPGIPLGVSVTAHKVVTAPVSVALSLETVQHEKSIGNAETILLPGETKLLTIQVPLLNYTSPFLQLKVSATGGFRDSQTKMISINQNTSLILVQTDKAIYKPGQKVRIRVVNVDRYLKPVFNPLTVIIENAKNDKLEEYKDVNSKNGNYTYGKGVQGQCELTVHYT**

BGLB022293-RC

BGLB022293-RA

BGLB022293-RD

BGLB022293-RB

BGLB022293-RE

BGLB022293-RH

BGLB022293-RF

BGLB022293-RG

BGLB021035-RA

BGLB031746-RA

210 220 230 240 250 260 270 280 290 300 310 320 330 340 350 360 370 380 390 400

....|....|....|....|....|....|....|....|....|....|....|....|....|....|....|....|....|....|....|....|....|....|....|....|....|....|....|....|....|....|....|....|....|....|....|....|....|....|....|....|

BgCD109 **ASSQEIYHKELNSDGVAVFDHLDWKKLSRNVDNITVQAAVTDETGRKEQGETTLAVYADPKRVRILDTSTTILRHGLPAHIYIEVSDHSGNPVSPVTLMMDVTHPELKGFTEVLNVPAGETIVKYTFIAIKSQEQSYYYNRGDGTLKAWLQMNDNVFDSKTFTVYRTKSPLALSILPLESQTIRVGESAIIKVNTSLPSY**

BGLB021085-RA **ASSQEIYHKELNSDGVAVFDHLDWKKLSRNVDNITVQAAVTDETGRKEQGETTLAVYADPKRVRILDTSTTILRHGLPAHIYVRLISPMFSYIVKQIHFYSTSIQVTPSWGVDADLEYCSNNFPRNFTVDVYR**

BGLB022293-RC  **MNDNVFDSKTFTVYRTKSPLALSILPLESQTIRVGESALIKVNTSLPSY**

BGLB022293-RA  **MNDNVFDSKTFTVYRTKSPLALSILPLESQTIRVGESALIKVNTSLPSY**

BGLB022293-RD  **MNDNVFDSKTFTVYRTKSPLALSILPLESQTIRVGESALIKVNTSLPSY**

BGLB022293-RB  **MNDNVFDSKTFTVYRTKSPLALSILPLESQTIRVGESALIKVNTSLPSY**

BGLB022293-RE  **MNDNVFDSKTFTVYRTKSPLALSILPLESQTIRVGESALIKVNTSLPSY**

BGLB022293-RH  **MNDNVFDSKTFTVYRTKSPLALSILPLESQTIRVGESALIKVNTSLPSY**

BGLB022293-RF  **MNDNVFDSKTFTVYRTKSPLALSILPLESQTIRVGESALIKVNTSLPSY**

BGLB022293-RG  **MNDNVFDSKTFTVYRTKSPLALSILPLESQTIRVGESALIKVNTSLPSY**

BGLB021035-RA

BGLB031746-RA

410 420 430 440 450 460 470 480 490 500 510 520 530 540 550 560 570 580 590 600

....|....|....|....|....|....|....|....|....|....|....|....|....|....|....|....|....|....|....|....|....|....|....|....|....|....|....|....|....|....|....|....|....|....|....|....|....|....|....|....|

BgCD109 **FDSTFAYLVMSQGNIVSAGQLKDNSFVITPTLEFCPLSRLLVYMIAGSESENGEVVLDAVDLTLTGCFTKEVKVEFEASETRTGTEVEMKVDVSRLDGSNEMPGQHDVFYLAVDQSIVLLQGSTDLNTDKVVSGLSSFDQVDESVTLSSAAAYFERHKLFYLTDASVWSRNQLFEKELMINRGPMLKKTSAT---ELLPD**

BGLB021085-RA

BGLB022293-RC **FDSTLAYLVMSQGNIVSAGQLKDNSFVITPTLEFCPLSRLLVYMIAGSESENGEVVLDAVDLTLTGCFTKEVRVEFEASETRTGTEVEMKVDVSRLDGSNEMPGQHDVFYLAVDQSIVLLQGSTDLNTDKVVSGLSSFDQVDESVTLSSAAAYFERHKLFYLTDASVWSRNQLFEKELMINRGQMLKKTSAT---ELLPD**

BGLB022293-RA **FDSTLAYLVMSQGNIVSAGQLKDNSFVITPTLEFCPLSRLLVYMIAGSESENGEVVLDAVDLTLTGCFTKEVRVEFEASETRTGTEVEMKVDVSRLDGSNEMPGQHDVFYLAVDQSIVLLQGSTDLNTDKVVSGLSSFDQVDESVTLSSAAAYFERYDVSYMTNLSMARYLHEFPIPVNAIFVQANDAPSPKTDRELNPY**

BGLB022293-RD **FDSTLAYLVMSQGNIVSAGQLKDNSFVITPTLEFCPLSRLLVYMIAGSESENGEVVLDAVDLTLTGCFTKEVRVEFEASETRTGTEVEMKVDVSRLDGSNEMPGQHDVFYLAVDQSIVLLQGSTDLNTDKVVSGLSSFDQVDESVTLSSAAAYFERYDVSYMTNLSMARYLHEFPIPVNAIFVQANDAPSPKN-------**

BGLB022293-RB **FDSTLAYLVMSQGNIVSAGQLKDNSFVITPTLEFCPLSRLLVYMIAGSESENGEVVLDAVDLTLTGCFTKEVRVEFEASETRTGTEVEMKVDVSRLDGSNEMPGQHDVFYLAVDQSIVLLQGSTDLNTDKVVSGLSSFDQVDESVTLSSAAAYFEHYQIKVAIDETLVVDNGQPPRFLAMASRA---------------D**

BGLB022293-RE **FDSTLAYLVMSQGNIVSAGQLKDNSFVITPTLEFCPLSRLLVYMIAGSESENGEVVLDAVDLTLTGCFTKEVRVEFEASETRTGTEVEMKVDVSRLDGSNEMPGQHDVFYLAVDQSIVLLQGSTDLNTDKVVSGLSSFDQVDESVTLSSAAAYFEHYQIKVAIDETLVVDNGQPPRFLAMASRAGN-------------D**

BGLB022293-RH **FDSTLAYLVMSQGNIVSAGQLKDNSFVITPTLEFCPLSRLLVYMIAGSESENGEVVLDAVDLTLTGCFTKEVRVEFEASETRTGTEVEMKVDVSRLDGSNEMPGQHDVFYLAVDQSIVLLQGSTDLNTDKVVSGLSSFDQVDESVTLSSAAAYFEHYQIKVAIDETLVVDNGQPPRFLAMASRAGNGEDSL--------D**

BGLB022293-RF **FDSTLAYLVMSQGNIVSAGQLKDNSFVITPTLEFCPLSRLLVYMIAGSESENGEVVLDAVDLTLTGCFTKEVRVEFEASETRTGTEVEMKVDVSRLDGSNEMPGQHDVFYLAVDQSIVLLQGSTDLNTDKVVSGLSSFDQVDESVTLSSAAAYFEHHKLLYLTDARVWSRKEFAHRKINVPNVFMEKSFVKIED-----D**

BGLB022293-RG **FDSTLAYLVMSQGNIVSAGQLKDNSFVITPTLEFCPLSRLLVYMIAGSESENGEVVLDAVDLTLTGCFTKEVRVEFEASETRTGTEVEMKVDVSRLDGSNEMPGQHDVFYLAVDQSIVLLQGSTDLNTDKVVSGLSSFDQVDESVTLSSAAAYFERYDVYYMTTLSMASDYLEVFLEKAFVAEAADGGPRLKN-------**

BGLB021035-RA

BGLB031746-RA

610 620 630 640 650 660 670 680 690 700 710 720 730 740 750 760 770 780 790 800

....|....|....|....|....|....|....|....|....|....|....|....|....|....|....|....|....|....|....|....|....|....|....|....|....|....|....|....|....|....|....|....|....|....|....|....|....|....|....|....|

BgCD109 **FDSEVDGKVPEAAYQTSARIRKDFPDTWLWGQAVTDVNGHLRSKVVLPDTITSWIVSAFAVNSEGLAVAKEPFKLTAFQLFFLSMNLPYSIKRGEVFVLRVTVFNYRSQHVQAVVSLAHSDQFMVVDETESEGWYSKSLSLEAYRASSVSYRINATTLGQITLHVTATDPADGQKDEVKRELLVKPEGVERSRAITKVMI**

BGLB021085-RA

BGLB022293-RC **FDTEVDGKVPEAAYQTSARIRKDFPDTWLWGQAVTDVNGHLRSKVVLPDTITSWIVSAFAVNSEGLAVAKEPFKLTAFQLFFLSMNLPYSIKRGEVFVLRVTVFNYRSQHVQAVVSLAHSDQFMVVDETDSEGWYSKSLSVST**

BGLB022293-RA **FDTEVDGKVPEAAYQTSARIRKDFPDTWLWGQAVTDVNGHLRSKVVLPDTITSWIVSAFAVNSEGLAVAKEPFKLTAFQLFFLSMNLPYSIKRGEVFVLRVTVFNYRSQHVQAVVSLAHSDQFMVVDETDSEGWYSKSLSVST**

BGLB022293-RD **FDTEVDGKVPEAAYQTSARIRKDFPDTWLWGQAVTDVNGHLRSKVVLPDTITSWIVSAFAVNSEGLAVAKEPFKLTAFQLFFLSMNLPYSIKRGEVFVLRVTVFNYRSQHVQAVVSLAHSDQFMVVDETDSEGWYSKSLSVST**

BGLB022293-RB **FDTEVDGKVPEAAYQTSARIRKDFPDTWLWGQAVTDVNGHLRSKVVLPDTITSWIVSAFAVNSEGLAVAKEPFKLTAFQLFFLSMNLPYSIKRGEVFVLRVTVFNYRSQHVQAVVSLAHSDQFMVVDETDSEGWYSKSLSVST**

BGLB022293-RE **FDTEVDGKVPEAAYQTSARIRKDFPDTWLWGQAVTDVNGHLRSKVVLPDTITSWIVSAFAVNSEGLAVAKEPFKLTAFQLFFLSMNLPYSIKRGEVFVLRVTVFNYRSQHVQAVVSLAHSDQFMVVDETDSEGWYSKSLSVST**

BGLB022293-RH **FDTEVDGKVPEAAYQTSARIRKDFPDTWLWGQAVTDVNGHLRSKVVLPDTITSWIVSAFAVNSEGLAVAKEPFKLTAFQLFFLSMNLPYSIKRGEVFVLRVTVFNYRSQHVQAVVSLAHSDQFMVVDETDSEGWYSKSLSVST**

BGLB022293-RF **FDTEVDGKVPEAAYQTSARIRKDFPDTWLWGQAVTDVNGHLRSKVVLPDTITSWIVSAFAVNSEGLAVAKEPFKLTAFQLFFLSMNLPYSIKRGEVFVLRVTVFNYRSQHVQAVVSLAHSDQFMVVDETDSEGWYSKSLSVST**

BGLB022293-RG **FDTEVDGKVPEAAYQTSARIRKDFPDTWLWGQAVTDVNGHLRSKVVLPDTITSWIVSAFAVNSEGLAVAKEPFKLTAFQLFFLSMNLPYSIKRGEVFVLRVTVFNYRSQHVQAVVSLAHSDQFMVVDETDSEGWYSKSLSVST**

BGLB021035-RA

BGLB031746-RA

810 820 830 840 850 860 870 880 890 900 910 920 930 940 950 960 970 980 990 1000

....|....|....|....|....|....|....|....|....|....|....|....|....|....|....|....|....|....|....|....|....|....|....|....|....|....|....|....|....|....|....|....|....|....|....|....|....|....|....|....|

BgCD109 **LNSGKSLSETFNIKWPQEKIVPDSQRVEIKVTGEVFGQALSGLENLVSIPFGCGEQNMISTVPNIFGLKYIRGTSQDGMEDLAAKLTNNMKLGYQRQVENYRHEDGSYSAWGDKFGNAESGSTWLTAFVIRSFAQASKFISVDTNVLETGIEFLKSCQDRTGKFIERGQVFHSDMQSGTGSGDGLTVYVLISMLEASQAL**

BGLB021085-RA

BGLB022293-RC

BGLB022293-RA

BGLB022293-RD

BGLB022293-RB

BGLB022293-RE

BGLB022293-RH

BGLB022293-RF

BGLB022293-RG

BGLB021035-RA  **MKLGYQRQVENYRHEDGSYSAWGDKFGNAESGSTWLTAFVIRSFAQASKFISVDNNVLETGIEFLKSCQDRTGKFIERGQVFH**

BGLB031746-RA

1010 1020 1030 1040 1050 1060 1070 1080 1090 1100 1110 1120 1130 1140 1150 1160 1170 1180 1190 1200

....|....|....|....|....|....|....|....|....|....|....|....|....|....|....|....|....|....|....|....|....|....|....|....|....|....|....|....|....|....|....|....|....|....|....|....|....|....|....|....|

BgCD109 **GETGSLSFKNQIDLALNYIRRNQDPEKLKQEKQIYLAAITAYSLSLVSNKDKDILQLIEQLLMVIKELQVPWSKVDSQDIKTLQSKQAAGDVGPPYIVKAQATRDLEIGAYVLLTLTRIENLAEGLELMKWLQSQQNSKGGFYSTQDTIMVLQALSEFGSKFRPGEVSSQLQVTHPVNLAFTLSGSRALLLQTATLPWDT**

BGLB021085-RA

BGLB022293-RC

BGLB022293-RA

BGLB022293-RD

BGLB022293-RB

BGLB022293-RE

BGLB022293-RH

BGLB022293-RF

BGLB022293-RG

BGLB021035-RA

BGLB031746-RA  **MKWLQSQQNSKGGFYSTQDTIMVLQALSEFGSKFRPGEVSSQLQVTHPVNLAFTLSGSRALLLQTATLPWDT**

1210 1220 1230 1240 1250 1260 1270 1280 1290 1300 1310 1320 1330 1340 1350 1360 1370 1380 1390

....|....|....|....|....|....|....|....|....|....|....|....|....|....|....|....|....|....|....|....|....|....|....|....|....|....|....|....|....|....|....|....|....|....|....|....|....|....|..

BgCD109 **TKVNVTLTGGTNSLAVVKVVYTYYTFAGDDDQVPTETLLFLETKSIRLGNGMHKVEACVKSSKSLKYKGMFVTTMALPSGEKPADDQSTILASNPMASRVEADEKFIHFYIDKAPSNEGYCLTANVEPHLEFEVQKPGFAQFYTYYDPDNVAEVPLSLTCHNCDTDTAVMVNMASVLLTTVVCLLASLLACM**

BGLB021085-RA

BGLB022293-RC

BGLB022293-RA

BGLB022293-RD

BGLB022293-RB

BGLB022293-RE

BGLB022293-RH

BGLB022293-RF

BGLB022293-RG

BGLB021035-RA

BGLB031746-RA **TKVNVTLTGGTNSLAVVKVVYTYYTFAGDDDQVPTETLLFLETKSIRLGNGMHKVEACVKSSKSLKYKGMFVTTMALPSGEKPADDQSTILASNPMASRVEADEKFIHFYIDKAPSNEGYCLTANVEPHLEFEVQKPGFAQFYTYYDPDNVAEVPLSLTCHNCDTDTAVMVNMASVLLTTVVCLLASLLACM**
