# Supplementary material for: A New Assessment of Thioester-Containing Proteins Diversity of the Freshwater Snail Biomphalaria glabrata
Source: Genes (Basel). 2020 Jan 7;11(1):69. doi: 10.3390/genes11010069 (PMC7016707; doi:10.3390/genes11010069)
Supplement: Supplementary file 1 [file genes-11-00069-s001.zip › Figure S5.pdf]

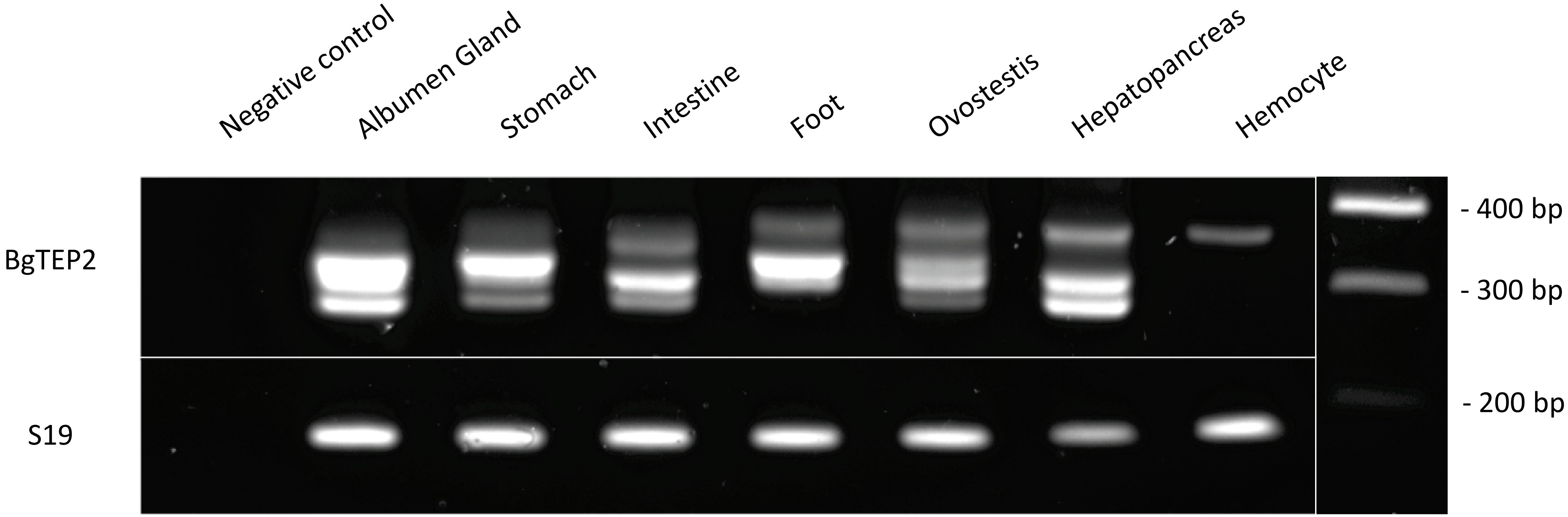
Negative control
Albumen Gland
Stomach
Intestine
Foot
Ovostestis
Hepatopancreas
Hemocyte
BgTEP2
S19
- 400 bp
- 300 bp
- 200 bp
