# Supplementary material for: A New Assessment of Thioester-Containing Proteins Diversity of the Freshwater Snail Biomphalaria glabrata
Source: Genes (Basel). 2020 Jan 7;11(1):69. doi: 10.3390/genes11010069 (PMC7016707; doi:10.3390/genes11010069)
Supplement: Supplementary file 1 [file genes-11-00069-s001.zip › File S1.docx]

**Figure S1: Alignment file of TEP protein sequences used for the phylogenetic analysis.** The file presents the protein sequence of 134 TEP from various organisms in a fasta format, after alignment with the Guidance 2 server using the MAFFT alogorithm.

**Figure S2: cDNA sequences of the eleven TEP of *Biomphalaria glabrata*.** Bold characters correspond to the protein coding sequences.

**Figure S3**: **ClustalW alignment of BgTEP protein sequences with related sequences from other organisms.** For sequences code name, see Table S2. Amino acids shared by at least 70% of the sequence are highlighted in black for identical one, in grey for similar one. Conserved protein domains are indicated by a black line. Black box is framing the thioester site. Black triangle indicates the position of the catalytic residue interacting with the thioester site. ++++ signals the protease sensitive region of complement-like factors. **A)** ClustalW alignment of BgC3-like TEP. **B)** ClustalW alignment of BgA2M. **C)** ClustalW alignment of BgMCR. **D)** ClustalW alignment of BgTEP and BgCD109.

**Figure S4**: **ClustalW alignment of each BgTEP protein sequence with corresponding protein sequences predicted from BglaB1 genome assembly.** Identical amino acid between predicted sequence from *Biomphalaria* *glabrata* genome assembly and sequence recovered from *B. glabrata* transcriptomes are highlighted in black. **A)** BgC3-1, **B)** BgC3-2, **C)** BgC3-3, **D)** BgA2M, **E)** BgMCR1, **F)** BgMCR2, **G)** BgTEP1, **H)** BgTEP2, **I)** BgTEP3, **J)** BgTEP4, **K)** BgCD109.

**Figure S5: BgTEP2 alternative splicing in snail tissues.** RT-PCR analysis of mRNA encoding BgTEP2 splice variants is performed in seven snail tissues: albumen gland, stomach, intestine, head-foot, ovotestis, hepatopancreas and hemocyte.

**Table S1: Table list of primers used to measure gene expression by quantitative PCR.** Name, nucleotide sequence, melting temperature (Tm), GC percent (GC %) and length (mer) are provided for each primer, as well as expected amplicon size for each couple of primers.

**Table S2**: **Table list of protein sequences used to construct phylogenetic tree**. For each protein sequence used in the phylogenetic tree are indicated the code name, the full name and the GenBank protein accession number of the sequence. Taxonomic data about the organism from which sequence comes are also provided, incuding the phylum, the subphylum, the class and the species name. Asterisk indicates protein for which no Genbank accession number is available, that is replaced by the corresponding Pub Med ID and DOI of the article.

**Table S3: Genome organization of BgTEP genes.** Each table column represents one BgTEP gene, each line corresponds to an exon. The position of each exon is indicated with the scaffold number from BglaB1 genome assembly and the corresponding base positions inside the scaffold. Exon encoding only 5’ or 3’ untranslated region (UTR) is highlighted in pink. Exon encoding 5’UTR-ORF or ORF-3’UTR junction is highlighted in yellow. All other ORF encoding exons are in green. The alternation of shades of green indicates a change in scaffold number from the BglaB1 genome assembly. Alternative spliced exons are written in purple. Missense or badly positioned exons from BglaB1 genome assembly are written in red. Asterisk indicates the alternative exon corresponding to the nucleotide sequence provided in Figure S1.

**Table S4: Genome organization of BgTEP2 splice variants.** For each BgTEP2 variant the table provided the name, the genome position, the nucleotide sequence and the length of the splice exon, as well as the sequences of adjacent introns (splice junctions). Transcript ID from *B. glabrata* transcriptomes expressing each of these BgTEP2 variants was also indicated. Conserved bases of the intron-exon boundaries are colored in red.

**File S1: Supplementary materials caption.**
