# Supplementary material for: A New Assessment of Thioester-Containing Proteins Diversity of the Freshwater Snail Biomphalaria glabrata
Source: Genes (Basel). 2020 Jan 7;11(1):69. doi: 10.3390/genes11010069 (PMC7016707; doi:10.3390/genes11010069)
Supplement: Supplementary file 1 [file genes-11-00069-s001.zip › Table S1.docx]

**Supplementary Table X: List primers used to measure BgTEP genes expression by Quantitative PCR**

| **Primer name** | **Sequence 5’-3’** | **Tm** | **GC %** | **mer** | **Amplicon size** |
| --- | --- | --- | --- | --- | --- |
| BgC3-1_F3 | TACTTCTTTGCCTTGAAGAATCC | 59,9 | 39,1 | 23 | 91 bp |
| BgC3-1_R3 | TCACTTTGATTGAACTGCCTGTA | 59,9 | 39.1 | 23 |  |
| BgC3-2_F1 | GTCTGTGTCAGGTTTCTAGGTA | 59,8 | 45,5 | 22 | 80 bp |
| BgC3-2_R1 | ACTGGGAGATAGCCAGTAAAT | 60 | 42,9 | 21 |  |
| BgC3-3_F2 | GATGGAGGAGCCAACAC | 59,8 | 58,8 | 17 | 83 bp |
| BgC3-3_R2 | AGTCGTTGATGTAGTTTAGGG | 60,1 | 42,9 | 21 |  |
| BgA2M_F2 | AGAGTCTTGGGCTCCTAC | 60 | 55,6 | 18 | 80 bp |
| BgA2M_R2 | TAAGCAGTTAGGGCAGTGAAT | 60 | 42,9 | 21 |  |
| BgMCR1_F1 | TTTGCTGCTACTCAGGATACTA | 59,9 | 40,9 | 22 | 91 bp |
| BgMCR1_R1 | TGCTCAAGTCAAATACATTACGAT | 60 | 33,3 | 24 |  |
| BgMCR2_F1 | AGGTATTTGATGCCTCGC | 60,2 | 50 | 18 | 83 bp |
| BgMCR2_R1 | GTAATGTGAGCCAGAAGGG | 60,2 | 52,6 | 19 |  |
| BgTEP2_F3 | GGACCAAGCAGTGCTATG | 60 | 55,6 | 18 | 87 bp |
| BgTEP2_R3 | AATGTCCAACTGTGGGATAC | 60,1 | 45 | 20 |  |
| BgTEP3_F1 | AATGGAGCAAATGGATTGTTTC | 60,1 | 36,4 | 22 | 82 bp |
| BgTEP3_R1 | AATAAACAGATATAGCCTCGCC | 60,1 | 40,9 | 22 |  |
| BgTEP4_F3 | TGTGAAAGAAATAATTAAGATCAACAAGAG | 60 | 26,7 | 30 | 164 bp |
| BgTEP4_R3 | TGTAGCCTAAGACAATTTCAGC | 59,9 | 42,9 | 21 |  |
| BgCD109_F1 | TCTGTAATTCTCAACTTGACGC | 60 | 40,9 | 22 | 105 bp |
| BgCD109_R1 | CTGAAGTACATTCGAGGCAC | 60 | 50 | 20 |  |
| S19_F | TTCTGTTGCTCGCCAC | 60 | 56,3 | 16 | 186 bp |
| S19_R | CCTGTATTTGCATCCTGTT | 60,1 | 42,1 | 19 |  |
